# Formation of trisubstituted buta-1,3-dienes and α,β-unsaturated ketones *via* the reaction of functionalized vinyl phosphates and vinyl phosphordiamidates with organometallic reagents†

**DOI:** 10.1039/d0ra07472a

**Published:** 2020-09-22

**Authors:** Petr Oeser, Jakub Koudelka, Hana Dvořáková, Tomáš Tobrman

**Affiliations:** Department of Organic Chemistry, University of Chemistry and Technology Prague, Technická 5 166 28 Prague 6 Czech Republic tomas.tobrman@vscht.cz; Laboratory of NMR Spectroscopy, University of Chemistry and Technology Prague, Technická 5 166 28 Prague 6 Czech Republic

## Abstract

We studied the reactions of vinyl phosphates and vinyl phosphordiamidates containing an ester functional group with organometallic reagents. We found that the functionalized vinyl phosphates were smoothly converted into tri- and tetrasubstituted buta-1,3-dienes *via* the reaction with aryllithium reagents. Moreover, the vinyl phosphordiamidates were converted into α,β-unsaturated ketones using Grignard reagents. Based on the performed experiments, we proposed a reaction mechanism, which was confirmed by means of the isolation of key intermediates.

## Abstract 2

We studied the reactions of vinyl phosphates and vinyl phosphordiamidates containing an ester functional group with organometallic reagents.

## Graphical abstract

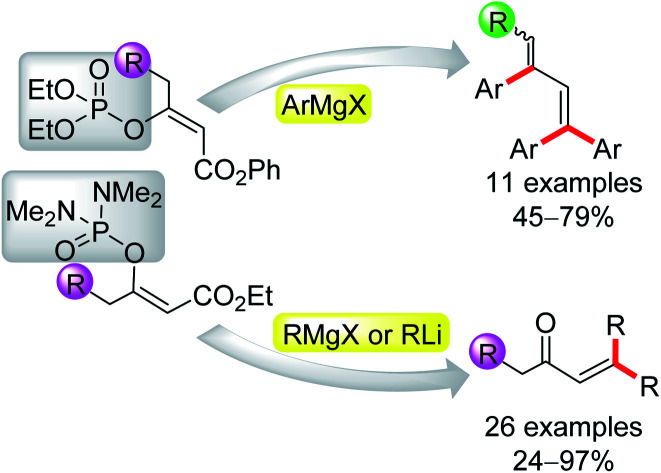

## Introduction

A Kumada–Tamao–Corriu (KTC) reaction is a representative example of the traditional cross-coupling reaction of an electrophilic template with an organometallic reagent, as represented by Grignard reagents. This reaction is frequently used for the formation of a C–C bond.^1^ Among the various electrophilic templates that are suitable for use in a KTC reaction, substrates containing an activated C–O bond are considered particularly attractive. The reason behind the popularity of such substrates is their availability, which is associated with their preparation. Recent examples of the cross-coupling reactions of ethers,^2^ tosylates,^3^ and triflates^4^ with Grignard reagents all illustrate the KTC reaction potential of compounds with an activated C–O bond. Using this approach, a wide variety of substances can be prepared, including substituted alkenes.

The stereoselective synthesis of di-, tri-,^5^ and tetrasubstituted^6^ double bonds has been the subject of significant research attention in recent decades. Vinyl phosphates also play a key role in the synthesis of substituted alkenes. Unsurprisingly, a number of KTC reactions have been described in which the reaction of the vinyl phosphates has been catalyzed by iron,^7^ nickel,^8^ or palladium^9^ catalysts in order to prepare the substituted double bond.

**Scheme 1:**
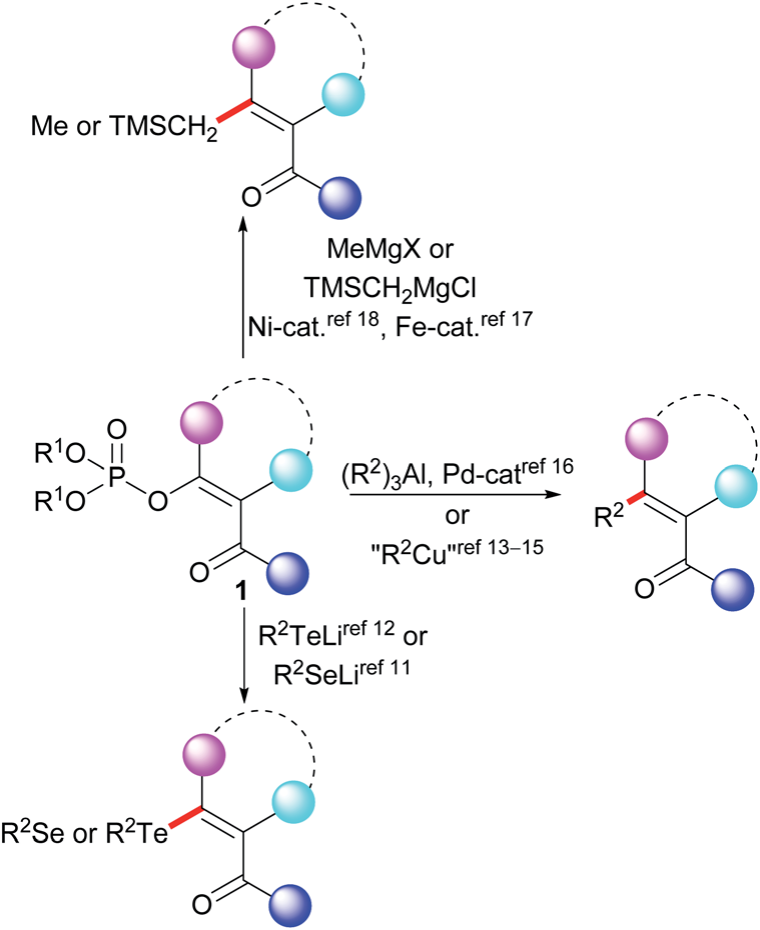
General scheme representing the replacement of the phosphate group of the functionalized vinyl phosphates 1.

Despite considerable progress having been made in relation to the preparation and application of functionalized Grignard reagents,^10^ the preparation of alkenes with functional groups that react with Grignard reagents using the KTC reaction remains difficult (Scheme 1). The vinyl phosphates 1 with a carbonyl group attached to the vinyl unit have been used in the stoichiometric synthesis of organoselenium^11^ and organotellurium compounds.^12^ A methodology for the stoichiometric cross-coupling reaction of the functionalized vinyl phosphates with dialkylcuprates has also been described.^13^ This methodology has been successfully used in the field of organic synthesis^14^ as well as in the total synthesis of naturally occurring compounds.^15^ The catalytic reactions of the functionalized vinyl phosphates represented by the general structure 1 include reactions with organoaluminum compounds,^16^ iron-catalyzed reactions with methylmagnesium halides,^17^ and nickel-catalyzed cross-coupling reactions with trimethylsilylmethylmagnesium chloride.^18^ These prior results contrast with a recent report of the KTC reaction of functionalized vinyl tosylates being performed in dry acetonitrile at 45 °C.^3*b*^ The absence of a general methodology for the KTC reaction of the functionalized vinyl phosphates 1 with Grignard reagents may indicate that this type of vinyl phosphate exhibits low stability in the presence of Grignard reagents. Our review of the literature indicated that a systematic study of the reactivity of the functionalized vinyl phosphates 1 with organometallic reagents has not previously been reported. Thus, based on our earlier studies of the cross-coupling reactions of vinyl phosphates,^19^ we decided to investigate the stability of the functionalized vinyl phosphates 2a–2c in the presence of Grignard reagents.

## Results and discussion

We began our study by synthesizing the vinyl phosphate 2a by means of the enolization of ethyl acetoacetate, followed by the reaction of the formed enolate with diethyl chlorophosphate. The prepared phosphate 2a was mixed with phenylmagnesium chloride, and then the reaction mixture was stirred for 2 hours at 23 °C. After the reaction mixture was quenched with aqueous ammonium chloride, the formation of a complex mixture of products was observed based on analysis of the ^1^H nuclear magnetic resonance (^1^H NMR) spectrum of the mixture. However, we isolated the trisubstituted diene 3a as a pure compound in an isolated yield of 27%. This finding was particularly interesting, as the synthesis of trisubstituted dienes is generally limited. The palladium-catalyzed coupling of tosylhydrazones with aryl halides,^20^ the selective arylation of vinylarenes,^21^ and diethyl-phosphite-promoted carbonyl olefination^22^ are recently reported methods for the synthesis of substituted buta-1,3-dienes. We observed that the isolated yield of the diene 3a strongly depended on the reaction workup, with comparatively higher yields of the alkene 3a being achieved with hydrochloric acid, sulfuric acid, and phosphoric acid (Table 1, entries 3–5). The isolated yield of the alkene 3a did not affect the increased equivalents of phenylmagnesium chloride or the use of the diphenylphosphate 2b (Table 1, entries 6 and 7). The best isolated yield of the diene 3a was obtained when phenyllithium was used in combination with the phenyl ester 2c (Table 1, entry 9).

**Table 1:** Optimization of the reaction of the vinyl phosphates 2a–c with phenylmagnesium chloride and phenyllithium

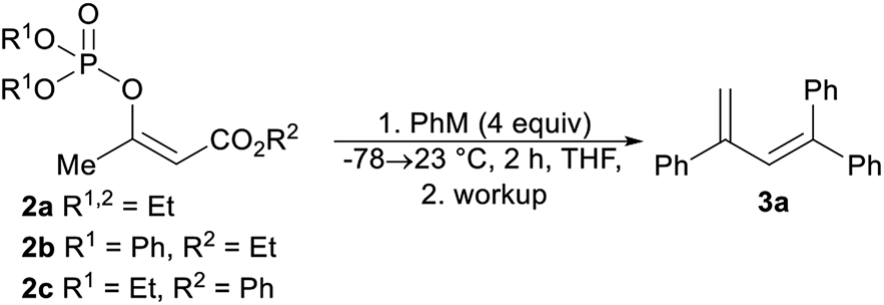				
Entry	2	M	Workup	3a^b^ (%)
1	2a	MgCl	NH_4_Cl	27
2	2a	MgCl	AcOH/MeOH	28
3	2a	MgCl	HCl	42
4	2a	MgCl	H_2_SO_4_	40
5	2a	MgCl	H_3_PO_4_	42
6	2a	MgCl	H_3_PO_4_	45^a^
7	2b	MgCl	H_3_PO_4_	40
8	2a	Li	H_3_PO_4_	51
9	2c	Li	H_3_PO_4_	65

a Six equivalents of phenylmagnesium chloride were used.

b Isolated yield.

**Scheme 2:**
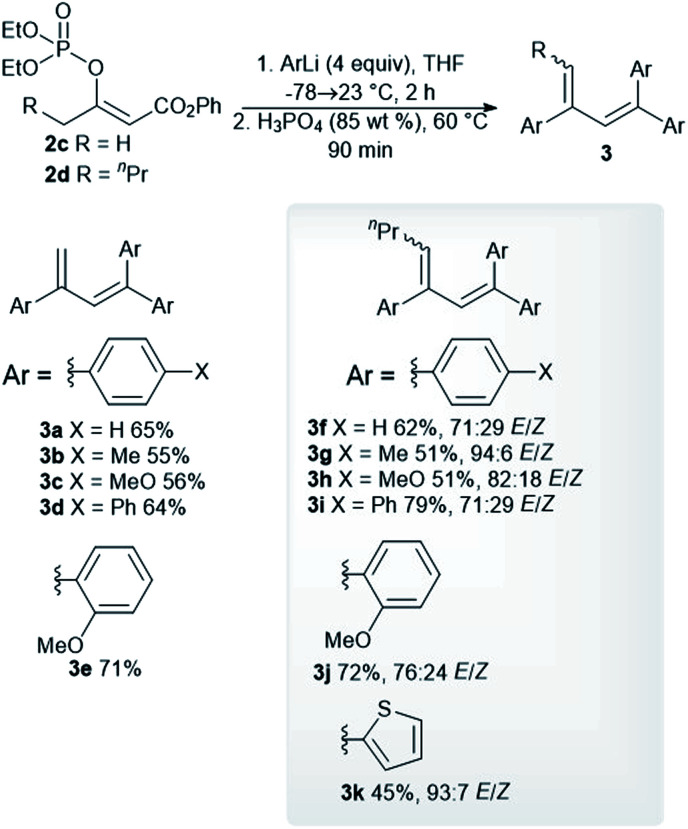
Reaction of the phosphates 2c and 2d with aryllithium reagents.

Optimized reaction conditions were used to evaluate the scope of the reaction (Scheme 2). The diethyl phosphate 2c reacted with the aryllithium reagents, thereby affording the corresponding dienes 3a–3d in a single step and in good isolated yields. 2-Methoxyphenyllithium, as an example of an *ortho*-substituted phenyl, gave the product 3e in a high isolated yield. A similar reactivity pattern was observed with regard to the diethyl phosphate with an extended alkyl chain 2d. In this case, the dienes 3f–3i were obtained in similar isolated yields to the dienes 3a–3c. The phosphate 2d was also reacted with 2-methoxyphenyllithium and 2-thienyllithium, as an example of a heteroaryl reagent, in order to give the dienes 3j and 3k. The dienes 3f–3k were obtained as an inseparable mixture of the (*E*)- and (*Z*)-stereoisomers and the double-bond geometry was determined by means of nuclear Overhauser effect (NOE) experiments. The use of an alkyllithium reagent, for example, *n*-butyllithium, failed to give the expected product. Instead, a complex reaction mixture was formed, as observed *via* the ^1^H NMR spectroscopy of the crude reaction mixture. It is worth noting that all the dienes 3a–3e were stored in a freezer, as significant decomposition was observed when they were stored at room temperature. However, the tetrasubstituted dienes 3f–3k showed excellent long-term storage stability.

**Scheme 3:**
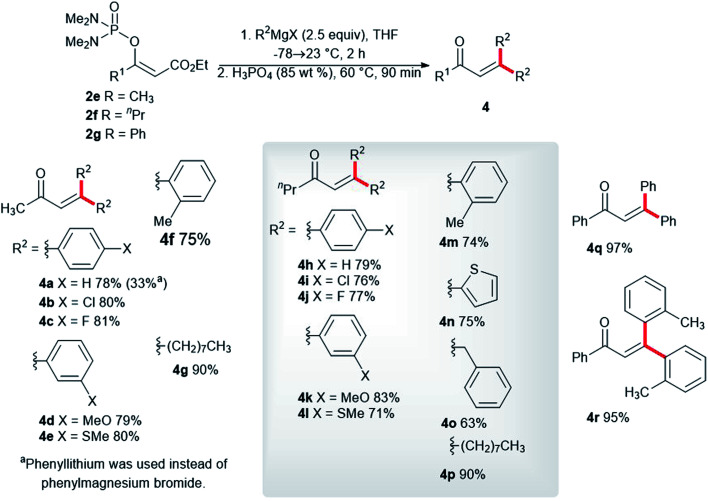
Synthesis of trisubstituted α,β-unsaturated ketones *via* the reaction of the phosphordiamidates 2e–2g with Grignard reagents.

We synthesized the vinyl phosphordiamidates 2e–2g so as to test their behavior during the developed one-pot synthesis of the substituted buta-1,3-dienes 3. Surprisingly, the vinyl phosphate 2e reacted smoothly with phenylmagnesium chloride to give the α,β-unsaturated ketone 4a in a high isolated yield (Scheme 3). α,β-Unsaturated ketones are known for their medicinal applications,^23^ and they are also valuable building blocks in relation to organic synthesis.^24^ Therefore, significant research efforts have been dedicated to identifying a novel approach to conjugated enones.^25^ Nickel-catalyzed 1,2-acyl migration,^26^ nucleophilic addition to enones,^27^ the cross-coupling reaction between acid fluorides and vinyl triflates,^28^ and carbonyl group olefinations^29^ are examples of such reactions. The conversion of the vinyl phosphordiamidate 2e into the ketone 4a observed in our study represents a hitherto undescribed reaction of phosphordiamidate en route to becoming α,β-unsaturated ketones. Further investigation in this regard showed that a significantly lower isolated yield of the ketone 4a was obtained with phenyllithium. Uniformly high yields of the corresponding ketones were obtained with the other *para*-, *meta*-, and *ortho*-substituted phenylmagnesium halides 4b–4f. Almost the same isolated yields of the ketones 4h–4m were obtained for the vinyl phosphordiamidate 2f. Additionally, the 2-thienylmagnesium bromide 4n, benzylmagnesium chloride 4o, and octylmagnesium chloride 4p reacted well with the phosphordiamidate 2f under the tested reaction conditions. The examination of the reactivity of the variously substituted vinyl phosphordiamidates was also extended to a phosphordiamidate with a phenyl substituent rather than an alkyl group, and the alkenes 4q and 4r were obtained in quantitative isolated yields.

Our attempts to prepare enones with a tetrasubstituted double bond by means of the reaction of the vinyl phosphordiamidate 2h with phenylmagnesium chloride proved to be less effective, with the formation of the ketones 5a and 6 being observed (Table 2, entry 1). Therefore, we tried to optimize the studied reaction in order to obtain only the ketone 5a. However, the use of phenylmagnesium chloride always gave a mixture of the ketones 5a and 6, regardless of the reaction conditions (Table 2, entries 2 and 3). The desired outcome was ultimately achieved through the reaction between phenyllithium and the phosphordiamidate 2h, and the ketone 5a was obtained in an isolated yield of 67% (Table 2, entry 4).

**Table 2:** Reaction of the cyclic vinyl phosphordiamidate 2h with phenyllithium and phenylmagnesium chloride

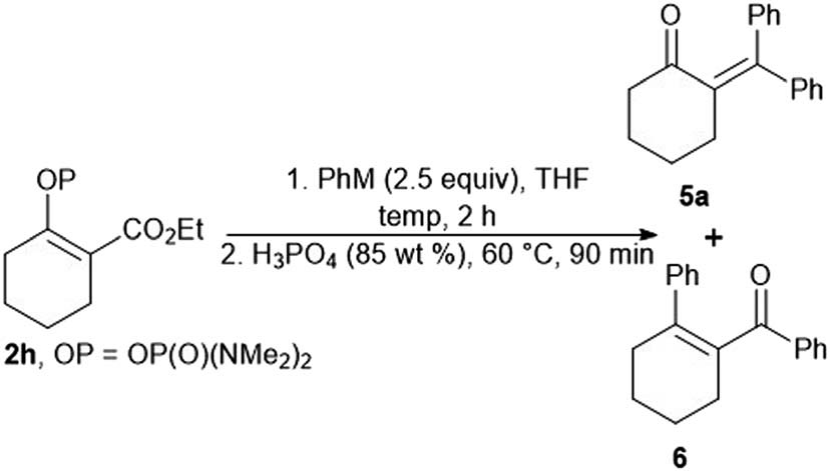				
Entry	M	Temp. [°C]	5a^a^ [%]	6^a^ [%]
1	MgCl	−78 to 23	36	33
2	MgCl	−40	24	8
3	MgCl^b^	0 to 23	25	45
4	Li	−78 to 23	67	0

a Isolated yield.

b 10 mol% of CuI was used as a catalyst.

**Scheme 4:**
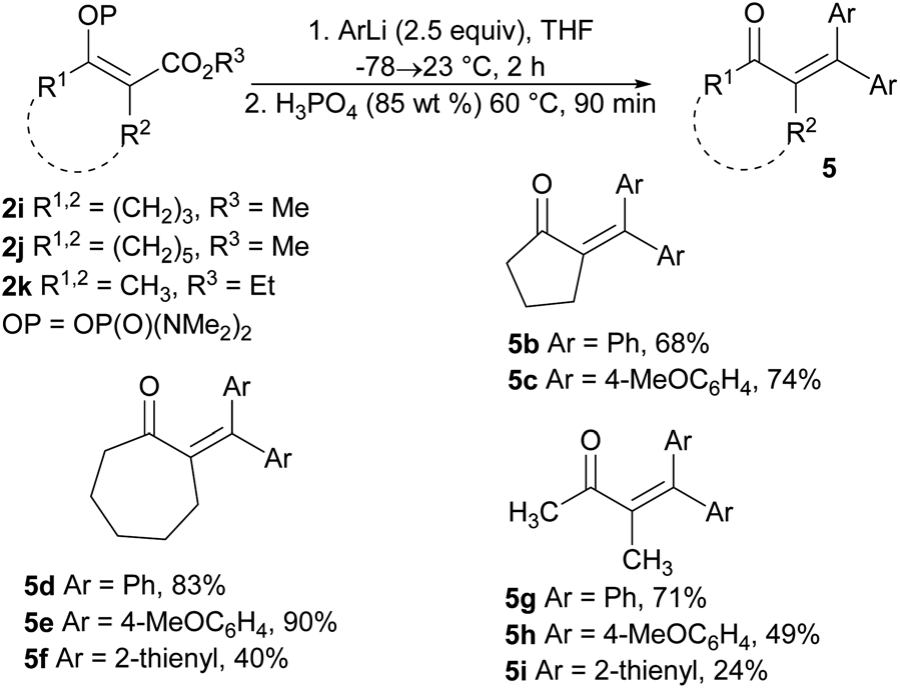
Synthesis of tetrasubstituted α,β-unsaturated ketones *via* the reaction of the phosphates 2i–2k with organolithium reagents.

The extension of the studied reaction to the cyclic vinyl phosphordiamidates 2i and 2j was successful in terms of the preparation of substituted cyclopentanones and cycloheptanones. In both cases, the expected ketones 5b, 5c, 5d, and 5e were obtained in very good isolated yields. Similar results were obtained for the acyclic phosphordiamidate 2k. Only the use of 2-thienyllithium proved less effective, and the reaction products 5f and 5i were obtained in low isolated yields (Scheme 4).

**Scheme 5:**
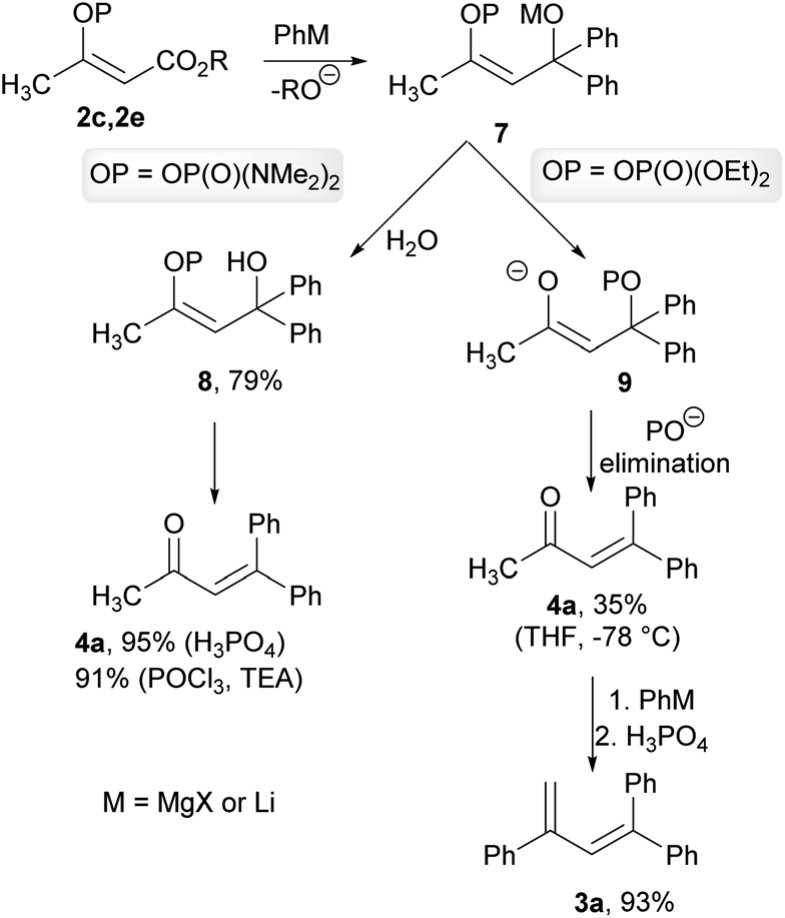
Proposed mechanism for the reaction of the vinyl phosphate 2c and the vinyl phosphordiamidate 2e with organometallic reagents.

As a result of the performed experiments, we were able to propose a mechanism for the observed transformations. We assumed that during the first step, an organometallic reagent addition to the ester functional group occurred and formed a common intermediate 7. The intermediate 7 with a phosphate group subsequently underwent *trans*-phosphorylation, with the resultant elimination forming an unsaturated ketone 4a, which was obtained in a low isolated yield of 35% when the crude reaction mixture was quenched at −78 °C. The resulting ketone 4a reacted with an organolithium reagent to form the corresponding tertiary alcohol, which was then dehydrated by phosphoric acid to give the diene 3a. This hypothesis was confirmed by the reaction of the ketone 4a with phenyllithium, with the subsequent dehydration giving the diene 3a in a 93% isolated yield. We also verified that ethyl acetoacetate did not provide the ketone 4a or the diene 3a under the studied conditions. In the case of the intermediate 7 with phosphordiamidate moiety, the *trans*-phosphorylation reaction is sluggish and the intermediate 7 was converted into the tertiary alcohol 8. The formation of this intermediate was confirmed by means of the hydrolysis of the crude reaction mixture with water, and the corresponding alcohol 8 was obtained in a 79% isolated yield. The formation of the ketone 4a was completed by the alcohol 8 dehydration. This was again confirmed by the dehydration of the independently prepared alcohol 8 into the product 4a in a quantitative isolated yield. Alternatively, the dehydration of the alcohol 8 could be performed using phosphoryl chloride in the presence of triethylamine in a similar isolated yield (Scheme 5).

## Conclusion

In conclusion, we studied the reactivity of both vinyl phosphates and vinyl phosphordiamidates. We found that vinyl phosphates can be converted into tri- and tetrasubstituted buta-1,3-dienes *via* the reaction with aryllithium reagents in dry THF at −78 °C to 23 °C. The tetrasubstituted dienes were isolated as a mixture of the (*E*)- and (*Z*)-stereoisomers. In the case of the vinyl phosphordiamidates, the reaction with Grignard reagents under the same reaction conditions resulted in the formation of unsaturated ketones, which were isolated in yields ranging from 24% to 97%. Based on the experimental results, we were able to propose a mechanism explaining the origins of both products.

## Experimental section

### Materials and methods

All reactions were performed under argon atmosphere. NMR spectra were measured on Varian MercuryPlus 300 (^1^H, 300.13 MHz; ^13^C, 75.46 MHz), Agilent 400 MR DD2 (^1^H, 400.13 MHz; ^13^C, 100.61 MHz) or Bruker Avance III 500 (^31^P, 202.45 MHz) spectrometer at 298 K. Chemical shifts of 31P NMR spectra are referenced to the signal of 85% H_3_PO_4_ that was assigned the chemical shift of 0. Mass spectra were measured on ZAB-SEQ (VG Analytical). The dry and degassed THF was prepared by PureSolv MD7. Silica gel (Merck, Silica Gel 60, 40–63 μm or Merck Silica Gel 60, 63–200 μm) was used for column chromatography. A phosphate 2a was prepared according to a published procedure.^30^*n*-BuLi (2.5 M solution in hexane), and other compounds were purchased from Sigma-Aldrich, FLuorochem and Acros Organics. Concentration of BuLi was determined by titration using menthol and 1,10-phenanthroline before use.

#### General procedure for the synthesis of starting phosphates 2a–2d (GP1)

1,3-Dicarbonyl compound was added to a suspension of sodium hydride (1.25 equiv.) in dry THF (5 mL/1 mmol) cooled to 0 °C. The resultant mixture was stirred for 30 min at 23 °C followed by addition of dialkyl chlorophosphate (1.25 equiv.). Then the reaction mixture was stirred for 2 h at 23 °C. The crude reaction mixture was quenched with saturated aqueous solution of ammonium chloride (1 mL/1 mmol), the organic layer was separated and the water layer was extracted with ether (3 × 3 mL/1 mmol). Combined organic layers were dried over MgSO_4_, the solvents were removed under reduce pressure and column chromatography (Silica gel) gave the product.

##### Ethyl (*Z*)-3-((diphenoxyphosphoryl)oxy)but-2-enoate (2b)

Prepared according to the GP1 from ethyl acetoacetate (0.260 g, 2.0 mmol), NaH (0.060 g, 2.50 mmol) and difenyl chlorophosphate (0.67 g, 2.5 mmol). Column chromatography (hexane/AcOEt 3 : 1, *R*_f_ = 0.45) gave 0.67 g (93%) of the title compound as a yellowish oil. ^1^H NMR (300 MHz, CDCl_3_) *δ* 7.39–7.16 (m, 10H), 5.43 (s, 1H), 4.11 (q, *J* = 7.1 Hz, 2H), 2.18–2.14 (m, 3H), 1.25 (t, *J* = 7.1 Hz, 3H); ^13^C NMR (75 MHz, CDCl_3_) *δ* 163.2 (d, *J* = 1.7 Hz), 156.6 (d, *J* = 7.2 Hz), 150.4 (d, *J* = 7.6 Hz), 129.7, 125.5 (d, *J* = 1.2 Hz), 120.1 (d, *J* = 4.9 Hz), 106.7 (d, *J* = 8.6 Hz), 60.0, 21.5 (d, *J* = 1.2 Hz), 14.0; HRMS (ESI) *m*/*z*: calcd for C_18_H_20_O_6_P [M + H]^+^ 363.0992; found 363.0992.

##### Phenyl (*Z*)-3-((diethoxyphosphoryl)oxy)but-2-enoate (2c)

Prepared according to the GP1 from phenyl acetoacetate (1.780 g, 10.0 mmol), NaH (0.480 g, 12.0 mmol) and diethyl chlorophosphate (2.070 g, 12.0 mmol). Column chromatography (hexane/AcOEt 2 : 1, *R*_f_ = 0.21) gave 2.61 g (83%) of the title compound as a yellowish oil. ^1^H NMR (300 MHz, CDCl_3_) *δ* 7.41–7.38 (m, 2H), 7.26–7.23 (m, 1H), 7.14–7.12 (m, 2H), 5.57 (s, 1H), 4.31–4.25 (m, 4H), 2.30 (s, 3H), 1.38–1.35 (m, 6H); ^13^C NMR (75 MHz, CDCl_3_) *δ* 161.7 (d, *J* = 1.7 Hz), 160.2 (d, *J* = 6.3 Hz), 150.4, 129.2, 125.5, 121.6, 104.3 (d, *J* = 8.5 Hz), 64.9 (d, *J* = 6.5 Hz), 21.7 (d, *J* = 1.2 Hz), 15.9 (d, *J* = 7.1 Hz); ^31^P NMR (202 MHz CDCl_3_) *δ* −8.38 (s); HRMS (ESI) *m*/*z*: calcd for C_14_H_19_O_6_P [M + Na]^+^ 337.0812; found 337.0815.

##### Phenyl (*Z*)-3-[(diethoxyphosphoryl)oxy]hept-2-enoate (2d)

Prepared according to the GP1 from phenyl 3-oxoheptanoate (3.524 g, 16.0 mmol), NaH (0.768 g, 19.20 mmol) and diethyl chlorophosphate (2.899 g, 16.80 mmol). Column chromatography (hexane/AcOEt 3 : 1, *R*_f_ = 0.26) gave 4.048 g (71%) of the title compound as a yellowish oil. ^1^H NMR (300 MHz, CDCl_3_): *δ* 7.38–7.35 (m, 2H), 7.23–7.19 (m, 1H), 7.12–7.09 (m, 2H), 5.57 (s, 1H), 4.29–4.17 (m, 4H), 2.54–2.50 (m, 2H), 1.64–1.58 (m, 2H), 1.44–1.38 (m, 2H), 1.32 (td, *J* = 7.1, 1.2 Hz, 6H), 0.95 (t, *J* = 7.3 Hz, 3H); ^13^C NMR (100 MHz, CDCl_3_) *δ* 164.1 (d, *J* = 6.9 Hz), 162.0 (d, *J* = 1.9 Hz), 150.4, 129.3, 125.6, 121.7, 104.1 (d, *J* = 7.8 Hz), 64.9 (d, *J* = 6.4 Hz), 35.0 (d, *J* = 1.0 Hz), 28.4, 22.0, 16.0 (d, *J* = 7.3 Hz), 13.7; HRMS (APCI) *m*/*z*: calcd for C_17_H_25_O_6_P [M + H]^+^ 357.1462; found 357.1463.

#### General procedure for the synthesis of phosphordiamidates 2e–2k (GP2)

1,3-Dicarbonyl compound was added to a suspension of sodium hydride (1.25 equiv.) in dry THF (5 mL/1 mmol) cooled to 0 °C. The resultant mixture was stirred for 30 min at 23 °C followed by addition of bis(*N*,*N*-dimethylamino)phosphoryl chloride (1.0 equiv.). Then the reaction mixture was stirred for 75 h at 23 °C. The crude reaction mixture was quenched with saturated aqueous solution of ammonium chloride (1 mL/1 mmol), the organic layer was separated and the water layer was extracted with AcOEt (2 × 3 mL/1 mmol). Combined organic layers were washed with brine (3 mL/1 mmol) dried over MgSO_4_, the solvents were removed under reduce pressure and column chromatography (Silica gel) gave the product.

##### Ethyl (*Z*)-3-[(bis(*N*,*N*-dimethylamino)phosphoryl)oxy]but-2-enoate (2e)

Prepared according to the GP2 from ethyl acetoacetate (13.01 g, 100.0 mmol), NaH (4.42 g, 110.0 mmol) and bis(*N*,*N*-dimethylamino)phosphoryl chloride (18.83 g, 110.0 mmol). Column chromatography (AcOEt, *R*_f_ = 0.33) gave 12.74 g (54%) of the title compound as a yellowish oil. ^1^H NMR (500 MHz, CDCl_3_) *δ* 5.16 (s, 1H), 4.07 (q, *J* = 7.1 Hz, 2H), 2.69 (d, *J* = 10.2 Hz, 12H), 2.11 (s, 3H), 1.20 (dd, *J* = 7.8, 6.5 Hz, 3H); ^13^C NMR (126 MHz, CDCl_3_) *δ* 164.0, 159.7 (d, *J* = 5.8 Hz), 103.6 (d, *J* = 7.5 Hz), 59.4, 36.4, 22.1 (d, *J* = 2.1 Hz), 14.3; ^31^P NMR (202 MHz, CDCl_3_) *δ* 15.02 (s); HRMS (ESI) *m*/*z*: calcd for C_10_H_21_N_2_O_4_P [M + Na]+ 287.1131; found 287.1135.

##### Ethyl (*Z*)-3-[(bis(*N*,*N*-dimethylamino)phosphoryl)oxy]hex-2-enoate (2f)

Prepared according to the GP2 from ethyl 3-oxohexanoate (4.746 g, 30.0 mmol), NaH (1.441 g, 36.0 mmol) and bis(*N*,*N*-dimethylamino)phosphoryl chloride (5.373 g, 31.50 mmol). Column chromatography (AcOEt, *R*_f_ = 0.32) gave 4.648 g (53%) of the title compound as a yellowish oil. ^1^H NMR (500 MHz, CDCl_3_) *δ* 5.24–5.22 (m, 1H), 4.12–4.07 (m, 2H), 2.72 (d, *J* = 10.2 Hz, 12H), 2.44–2.38 (m, 2H), 1.61–1.54 (m, 2H), 1.26–1.21 (m, 3H), 0.97–0.92 (m, 3H); ^13^C NMR (100 MHz, CDCl_3_) *δ* 164.1, 163.5 (d. *J* = 6.2 Hz), 103.1 (d, *J* = 7.2 Hz), 59.5, 37.3, 36.6 (d, *J* = 4.3 Hz), 20.0, 14.3, 13.5 (d, *J* = 1.4 Hz); HRMS (APCI) *m*/*z*: calcd for C_12_H_25_N_2_O_4_P [M + H]^+^ 293.1625; found 293.1628.

##### Ethyl (*Z*)-3-((bis(*N*,*N*-dimethylamino)phosphoryl)oxy)-3-phenylacrylate (2g)

Prepared according to the GP2 from ethyl benzoylacetate (3.840 g, 20.0 mmol), NaH (0.960 g, 24.0 mmol) and bis(*N*,*N*-dimethylamino)phosphoryl chloride (4.120 g, 24.0 mmol). Column chromatography (AcOEt, *R*_f_ = 0.38) gave 3.86 g (55%) of the title compound as a yellowish oil. ^1^H NMR (500 MHz, CDCl_3_) *δ* 7.64–7.52 (m, 2H), 7.43–7.42 (m, 3H), 5.81 (s, 1H), 4.21 (q, *J* = 7.1 Hz, 2H), 2.61 (d, *J* = 10.1 Hz, 12H), 1.34 (t, *J* = 7.1 Hz, 3H); ^13^C NMR (126 MHz, CDCl_3_) *δ* 164.3 (d, *J* = 2.1 Hz), 158.6 (d, *J* = 6.7 Hz), 136.0 (d, *J* = 1.8 Hz), 130.2, 128.3, 127.4, 107.1 (d, *J* = 6.3 Hz), 60.1, 36.6 (d, *J* = 4.6 Hz), 14.3; ^31^P NMR (202 MHz, CDCl_3_) *δ* 15.23 (s). HRMS (ESI) *m*/*z*: calcd for C_15_H_23_N_2_O_4_P [M + Na]^+^ 349.1288; found 349.1294.

##### Ethyl 2-((bis(*N*,*N*-dimethylamino)phosphoryl)oxy)cyclohex-1-ene-1-carboxylate (2h)

Prepared according to the GP2 from ethyl 2-oxocyclohexane-1-carboxylate (3.404 g, 20 mmol), NaH (0.960 g, 24 mmol) and bis(*N*,*N*-dimethylamino)phosphoryl chloride (3.582 g; 21.0 mmol). Column chromatography (AcOEt, *R*_f_ = 0.21) gave 4.578 g (75%) of the title compound as a yellowish oil. ^1^H NMR (300 MHz, CDCl_3_) *δ* 4.16 (qd, *J* = 7.2, 1.2 Hz, 2H), 2.68 (d, *J* = 10.1, 12H), 2.48–2.44 (m, 2H), 2.34–2.31 (m, 2H), 1.71–1.56 (m, 4H), 1.28–1.24 (m, 3H); ^13^C NMR (100 MHz, CDCl_3_) *δ* 153.6 (d, *J* = 6.6 Hz), 113.9 (d, *J* = 7.4 Hz), 111.6 (d, *J* = 4.7 Hz), 60.0, 36.5 (d, *J* = 4.3 Hz), 29.3 (d, *J* = 1.7 Hz), 25.6, 22.3, 21.6, 14.2; ^31^P NMR (202 MHz, CDCl_3_): *δ* 14.88; HRMS (APCI) *m*/*z*: calcd for C_13_H_25_N_2_O_4_P [M + H]^+^ 305.1625; found 305.1628.

##### Methyl 2-[(bis(*N*,*N*-dimethylamino)phosphoryl)oxy]cyclopent-1-ene-1-carboxylate (2i)

Prepared according to the GP2 from ethyl 2-oxocyclopentane-1-carboxylate (1.424 g, 10.0 mmol), NaH (0.480 g, 12.0 mmol) and bis(*N*,*N*-dimethylamino)phosphoryl chloride (1.791 g; 10.50 mmol). Column chromatography (AcOEt, *R*_f_ = 0.28) gave 2.127 g (77%) of the title compound as a yellowish oil. ^1^H NMR (400 MHz, CDCl_3_) *δ* 3.69 (s, 3H), 2.72–2.70 (m, 14H), 2.57–2.57 (m, 2H), 1.93–1.85 (m, 2H); ^13^C NMR (100 MHz, CDCl_3_) *δ* 164.6, 159.6 (d, *J* = 5.3 Hz), 112.2 (d, *J* = 7.6 Hz), 50.9, 36.4 (d, *J* = 4.4 Hz), 33.5 (d, *J* = 1.9 Hz), 28.7, 19.3; ^31^P NMR (202 MHz, CDCl_3_): *δ* 15.63; HRMS (APCI) *m*/*z*: calcd for C_11_H_21_N_2_O_4_P [M + H]^+^ = 277.1312; found 277.1315.

##### Methyl 2-[(bis(*N*,*N*-dimethylamino)phosphoryl)oxy]cyclohept-1-ene-1-carboxylate (2j)

Prepared according to the GP2 from ethyl 2-oxocycloheptane-1-carboxylate (2.558 g, 15.0 mmol), NaH (0.721 g, 18.0 mmol) and bis(*N*,*N*-dimethylamino)phosphoryl chloride (2.729 g, 16.0 mmol). Column chromatography (AcOEt, *R*_f_ = 0.25) gave 3.142 g (69%) of the title compound as a colorless liquid. ^1^H NMR (300 MHz, CDCl_3_) *δ* 3.55 (s, 3H), 2.58–2.43 (m, 14H), 2.26–2.26 (m, 2H), 1.59–1.37 (m, 6H); ^13^C NMR (100 MHz, CDCl_3_) *δ* 167.6 (d, *J* = 1.7 Hz), 158.2 (d, *J* = 7.3 Hz), 118.7 (d, *J* = 7.4 Hz), 51.1, 36.1 (d, *J* = 4.2 Hz), 33.6 (d, *J* = 1.6 Hz), 31.2, 27.4, 25.8 (d, *J* = 1.4 Hz), 23.9 (d, *J* = 0.6 Hz); ^31^P NMR (202 MHz, CDCl_3_): *δ* 14.67; HRMS (APCI) *m*/*z*: calcd for C_13_H_25_N_2_O_4_P [M + H]^+^ 305.1625; found 305.1626.

##### Ethyl (*Z*)-3-[(bis(*N*,*N*-dimethylamino)phosphoryl)oxy]-2-methylbut-2-enoate (2k)

Prepared according to the GP2 from ethyl 2-methylacetoacetate (2.88 g, 20 mmol), NaH (0.960 g, 24.0 mmol) and bis(*N*,*N*-dimethylamino)phosphoryl chloride (4.120 g, 24.0 mmol). Column chromatography (AcOEt, *R*_f_ = 0.33) gave 2.60 g (50%) of the title compound as a yellowish oil. ^1^H NMR (500 MHz, CDCl_3_) *δ* 4.17 (q, *J* = 7.1 Hz, 2H), 2.67 (d, *J* = 10.1 Hz, 12H), 2.11 (s, 3H), 1.83 (s, 3H), 1.27 (t, *J* = 7.1 Hz, 3H); ^13^C NMR (126 MHz, CDCl_3_) *δ* 167.3, 150.0 (d, *J* = 6.9 Hz), 112.5 (d, *J* = 7.8 Hz), 60.2, 36.5 (d, *J* = 3.8 Hz), 18.2 (d, *J* = 1.6 Hz), 14.7, 14.2; ^31^P NMR (202 MHz, CDCl_3_) *δ* 15.08 (s); HRMS (ESI) *m*/*z*: calcd for C_11_H_23_N_2_O_4_P [M + Na]^+^ 301.1288; found 301.1289.

#### General procedure for the synthesis of substituted buta-1,3-dienes 3 (GP3)

*n*-Butyllithium (4.0 equiv.) was added to a solution of aryl halide (4.1 equiv.) in dry THF (3 mL/1 mmol) cooled to −78 °C and the resultant mixture was stirred for 30 min at −78 °C followed by addition of enol phosphates 2a–2d (1 equiv.). Then the crude reaction mixture was stirred for 2 h at 23 °C. The reaction mixture was quenched by phosphoric acid (1 mL/1 mmol, 85 wt%) and the reaction mixture was stirred for 1 h at 60 °C. Then the reaction mixture was cooled to 23 °C, extracted with ether (2 × 4 mL mmol^−1^). The combined organic phases were washed with water (1 × 4 mL mmol^−1^), aqueous solution of saturated sodium bicarbonate (1 × 4 mL mmol^−1^) and brine (1 × 4 mL mmol^−1^). The organic layer was dried over MgSO_4_, the solvents were removed under reduce pressure and the product was obtained by column chromatography (Silica gel).

##### 1,1,3-Triphenylbuta-1,3-diene (3a)

Prepared according to the GP3 from bromobenzene (0.320 g, 2.05 mmol), ^*n*^BuLi (0.83 mL, 2.0 mmol, 2.4 M) and enol phosphate 2c (0.160 g, 0.5 mmol). Column chromatography (hexane/toluene 99 : 1, *R*_f_ = 0.47) gave 0.092 g (65%) of the title compound as a colorless oil. ^1^H NMR (300 MHz, CDCl_3_) *δ* 7.40–7.15 (m, 15H), 6.74 (d, *J* = 1.2 Hz, 1H), 5.39 (d, *J* = 1.4 Hz, 1H), 5.03 (t, *J* = 1.4 Hz, 1H), in accordance with literature.^22^

##### 1,1,3-Tris(4-methylphenyl)buta-1,3-diene (3b)

Prepared according to the GP3 from 4-bromotoluene (0.350 g, 2.05 mmol), ^*n*^BuLi (0.83 mL, 2.0 mmol, 2.4 M) and enol phosphate 2c (0.160 g, 0.5 mmol). Column chromatography (hexane, *R*_f_ = 0.34) gave 0.089 g (55%) of the title compound as a colorless oil. ^1^H NMR (400 MHz, CDCl_3_) *δ* 7.37–7.37 (m, 2H), 7.26–7.24 (m, 2H), 7.15–7.08 (m, 6H), 7.05–7.03 (m, 2H), 6.67–6.66 (m, 1H), 5.38–5.37 (m, 1H), 4.97–4.96 (m, 1H), 2.38 (s, 3H), 2.35 (s, 3H), 2.33 (s, 3H); ^13^C NMR (101 MHz, CDCl_3_) *δ* 145.0, 144.4, 140.6, 138.1, 137.4, 137.3, 137.2, 136.6, 130.0, 128.9, 128.8, 128.7, 127.9, 127.5, 126.5, 115.9, 21.3, 21.2, 21.1; HRMS (APCI) *m*/*z*: calcd for C_25_H_24_ [M + H]^+^ 325.1951 found 325.1945.

##### 1,1,3-Tris(4-methoxyphenyl)buta-1,3-diene (3c)

Prepared according to the GP3 from 4-bromoanisole (0.38 g, 2.05 mmol), ^*n*^BuLi (0.83 mL, 2.0 mmol, 2.4 M) and enol phosphate 2c (0.160 g, 0.5 mmol). Column chromatography (hexane/AcOEt 20 : 1, *R*_f_ = 0.23) gave 0.104 g (56%) of the title compound as a yellowish oil. ^1^H NMR (400 MHz, CDCl_3_) *δ* 7.36–7.33 (m, 2H), 7.29–7.26 (m, 2H), 7.09–7.06 (m, 2H), 6.87–6.84 (m, 2H), 6.80–6.72 (m, 4H), 6.59–6.57 (m, 1H), 5.31–5.31 (m, 1H), 4.95–4.93 (m, 1H), 3.83 (s, 3H), 3.79 (s, 3H), 3.77 (s, 3H); ^13^C NMR (101 MHz, CDCl_3_) *δ* 159.2, 159.1, 158.6, 144.9, 143.6, 136.1, 133.5, 132.7, 131.3, 129.2, 127.8, 126.6, 115.1, 113.5, 113.4, 113.3, 55.3, 55.3, 55.2. HRMS (APCI) *m*/*z*: calcd for C_25_H_24_O_3_ [M + H]^+^ 373.1798; found 373.1793.

##### 1,1,3-Tris[(1,1′-biphenyl)-4-yl]buta-1,3-diene (3d)

Prepared according to the GP3 from 4-bromobiphenyl (0.48 g, 2.05 mmol), ^*n*^BuLi (0.83 mL, 2.0 mmol, 2.4 M) and enol phosphate 2c (0.160 g, 0.5 mmol). Column chromatography (hexane/AcOEt 20 : 1, *R*_f_ = 0.57) gave 0.092 g (64%) of the title compound as a white solid, mp = 64.0–66.0 °C. ^1^H NMR (400 MHz, CDCl_3_) *δ* 7.65–7.25 (m, 17H), 6.89 (s 1H), 5.51 (s, 1H), 5.20 (s, 1H); ^13^C NMR (101 MHz, CDCl_3_) *δ* 145.1, 143.9, 142.0, 140.82, 140.80, 140.7, 140.5, 140.3, 139.9, 139.6, 138.9, 130.7, 128.8, 128.71, 128.69, 128.53, 128.48, 127.4, 127.29, 127.22, 127.20, 127.1, 127.04, 127.02, 126.95, 126.8, 126.6, 118.0; HRMS (APCI) *m*/*z*: calcd for C_40_H_30_ [M + H]^+^ 511.2420; found 511.2397.

##### 1,1,3-Tris(2-methoxyphenyl)buta-1,3-diene (3e)

Prepared according to the GP3 from 2-bromoanisole (0.380 g, 2.05 mmol), ^*n*^BuLi (0.83 mL, 2.0 mmol, 2.4 M) and enol phosphate 2c (0.160 g, 0.5 mmol). Column chromatography (hexane/AcOEt 20 : 1, *R*_f_ = 0.22) gave 0.132 g (71%) of the title compound as a yellowish oil. ^1^H NMR (400 MHz, CDCl_3_) *δ* 7.30–7.19 (m, 2H), 7.12–7.10 (m, 1H), 7.03–6.87 (m, 4H), 6.84–6.81 (m, 2H), 6.76–6.72 (m, 1H), 6.70–6.66 (m, 1H), 6.51–6.45 (m, 2H), 5.36–5.35 (m, 1H), 5.21–5.20 (m, 1H), 3.71 (s, 3H), 3.61 (s, 6H); ^13^C NMR (101 MHz, CDCl_3_) *δ* 157.3, 156.3, 156.0, 144.8, 136.0, 133.8, 132.2, 130.8, 130.4, 130.4, 130.1, 129.8, 128.1, 128.0, 127.7, 120.4, 120.3, 119.5, 119.1, 111.8, 109.5, 109.4, 55.8, 55.0, 54.9; HRMS (APCI) *m*/*z*: calcd for C_25_H_24_O_3_ [M + H]^+^ 373.1798; found 373.1789.

##### (*E*/*Z*)-1,1,3-Triphenylhepta-1,3-diene (3f)

Prepared according to the GP3 from bromobenzene (0.320 g, 2.05 mmol), ^*n*^BuLi (0.83 mL, 2.0 mmol, 2.4 M) and enol phosphate 2d (0.178 g, 0.5 mmol). Column chromatography (hexane, *R*_f_ = 0.35) gave 0.101 g (62%, *E*/*Z* = 79 : 21) of the title compound as a yellowish oil. ^1^H NMR (300 MHz, CDCl_3_) *δ* 7.37–7.10 (m, 15H, *E* and *Z*), 6.69–6.68 (m, 1H, *E* and *Z*), 5.77–5.73 (m, 1H, *E*^major^), 5.64–5.61 (m, 1H, *Z*^minor^), 1.99–1.95 (m, 2H, *E* and *Z*), 1.24–1.15 (m, 2H, *E* and *Z*), 0.84 (t, *J* = 6.0 Hz, 3H, *E*^major^), 0.72 (t, *J* = 6.0 Hz, 3H, *Z*^minor^); ^13^C NMR (100 MHz, CDCl_3_) *δ* 145.1, 143.6^minor^, 143.3^major^, 141.8^major^, 141.6^minor^, 140.4^minor^, 140.3^major^, 139.7^minor^, 138.2^minor^, 137.3^major^, 135.6^major^, 131.6^major^, 131.3^minor^, 130.4, 129.9, 129.2, 128.2, 128.1, 128.0, 127.9, 127.7, 127.6, 127.5, 127.1, 127.0, 126.7, 126.50, 126.47, 126.4, 126.3, 31.9^major^, 31.2^minor^, 22.7^minor^, 22.2^major^, 14.0^major^, 13.7^minor^; HRMS (APCI) *m*/*z*: calcd for C_25_H_24_ [M + H]^+^ 325.1951; found 325.1953.

##### 1,1,3-Tris(4-methylphenyl)hepta-1,3-diene (3g)

Prepared according to the GP3 from 4-bromotoluene (0.212 g, 1.23 mmol), ^*n*^BuLi (0.49 mL, 2.40 M, 1.18 mmol), and enol phosphate 2d (0.107 g, 0.30 mmol). Column chromatography (hexane, *R*_f_ = 0.28) gave the title compound 0.056 g (51%, *E*/*Z* = 83 : 17) as a yellowish oil. ^1^H NMR (300 MHz, CDCl_3_) *δ* 7.31–7.24 (m, 3H), 7.14–6.95 (m, 9H), 6.56–6.55 (m, 1H, *Z*^minor^), 6.53–6.52 (s, 1H, *E*^major^), 5.68 (t, *J* = 6.0 Hz, 1H, *E*^major^), 5.53 (t, *J* = 6.0 Hz, 1H, *Z*^minor^), 2.37 (s, 3H, *E*^major^), 2.32 (s, 3H, *Z*^minor^), 2.30 (s, 3H, *E*^major^), 2.29 (s, 3H, *Z*^minor^), 2.27 (s, 3H, *E*^major^), 1.96–1.84 (m, 2H, *E* and *Z*), 1.19–1.09 (m, 2H, *E* and *Z*), 0.80 (t, *J* = 6.0 Hz, 3H, *E*^major^), 0.69 (t, *J* = 6.0 Hz, 3H, *Z*^minor^); ^13^C NMR (75 MHz, CDCl_3_, only major peaks are reported) *δ* 144.7, 140.7, 138.9, 137.5, 137.2, 136.9, 136.5, 136.1, 130.4, 130.2, 129.7, 129.6, 129.0, 128.9, 128.8, 128.7, 128.7, 128.3, 128.1, 127.5, 126.4, 125.4, 31.9, 22.2, 21.2, 21.1, 21.0, 14.0; HRMS (APCI) *m*/*z*: calcd for C_28_H_30_ [M + H]^+^ 367.2420; found 367.2419.

##### 1,1,3-Tris(4-methoxyphenyl)hepta-1,3-diene (3h)

Prepared according to the GP3 from 4-bromoanisole (0.230 g, 1.23 mmol) a ^*n*^BuLi (0.49 mL, 2.40 M, 1.17 mmol) and enol phosphate 2d (0.107 g, 0.30 mmol). Column chromatography (hexane/EtOAc 20 : 1, *R*_f_ = 0.35) gave 0.063 g (51%, *E*/*Z* = 82 : 18) of the title compound as a yellowish oil. ^1^H NMR (300 MHz, CDCl_3_) *δ* 7.31–7.26 (m, 4H), 7.04–6.65 (m, 8H), 6.54–6.53 (m, 1H, *Z*^minor^), 6.48–6.47 (m, 1H, *E*^major^), 5.63 (t, *J* = 6.0 Hz, 1H, *E*^major^), 5.58 (t, *J* = 6.0 Hz, 1H, *Z*^minor^), 3.83 (s, 3H, *E*^major^), 3.79 (s, 3H, *Z*^minor^), 3.77 (s, 3H, *E*^major^), 3.76 (s, 3H, *Z*^minor^), 3.75 (s, 3H, *E*^major^), 1.98–1.89 (m, 2H, *E* and *Z*), 1.26–1.15 (m, 2H, *E* and *Z*), 0.83 (t, *J* = 6.0 Hz, 3H, *E*^major^), 0.74 (t, *J* = 6.0 Hz, 3H, *Z*^minor^); ^13^C NMR (100 MHz, CDCl_3_) *δ* 159.1, 158.5, 158.4, 143.9, 136.8, 136.2, 134.6, 133.0, 131.0, 129.3, 129.2, 127.6, 124.4, 113.4, 113.3, 113.1, 113.0, 55.3, 55.2, 55.1, 31.8^major^, 31.3^minor^, 22.9^minor^, 22.3^major^, 14.0^major^, 13.8^minor^; HRMS (APCI) *m*/*z*: calcd for C_28_H_30_O_3_ [M + H]^+^ 415.2268; found 415.2264.

##### 1,1,3-Tris[(1,1′-biphenyl)-4-yl]hepta-1,3-diene (3i)

Prepared according to the GP3 from 4-bromobiphenyl (0.287 g, 1.23 mmol), ^*n*^BuLi (0.49 mL, 2.40 M, 1.17 mmol) and enol phosphate 2d (0.107 g, 0.30 mmol). Column chromatography (hexane → hexane/EtOAc 30 : 1, *R*_f_ = 0.1) gave 0.119 g (72%, *E*/*Z* = 86 : 14) of the title compound as a yellowish solid, mp = 49.0–50.0 °C. ^1^H NMR (300 MHz, CDCl_3_) *δ* 7.65–7.06 (m, 27H, *E* and *Z*), 6.85 (s, 1H, *Z*^minor^), 6.82 (s, 1H, *E*^major^), 5.86–5.78 (m, 1H, *E* and *Z*), 2.10–2.01 (m, 2H, *E* and *Z*), 1.30–1.23 (m, 2H, *E* and *Z*), 0.88 (t, *J* = 7.3 Hz, 3H, *E*^major^), 0.76 (s, *J* = 7.3 Hz, 3H, *Z*^minor^); ^13^C NMR (75 MHz, CDCl_3_) *δ* 144.3, 142.4, 142.0, 141.0, 140.9, 140.8, 140.8, 140.7, 140.4, 139.8, 139.3, 139.3, 139.2, 139.0, 138.5, 138.4, 137.0, 136.6, 132.0, 131.6, 130.8, 130.4, 129.8, 128.8, 128.7, 128.6, 128.6, 128.0, 127.3, 127.2, 127.1, 127.0, 126.9, 126.9, 126.8, 126.7, 126.4, 126.2, 32.0^major^, 31.4^minor^, 22.8^minor^, 22.3^major^, 14.1^major^, 13.8^minor^; HRMS (APCI) *m*/*z*: calcd for C_43_H_36_ [M + H]^+^ 553.2890; found 553.2894.

##### 1,1,3-Tris(2-methoxyphenyl)hepta-1,3-diene (3j)

Prepared according to the GP3 from 2-bromoanisole (0.230 g, 1.23 mmol), ^*n*^BuLi (0.49 mL, 2.40 M, 1.17 mmol) and enol phosphate 2d (0.107 g, 0.30 mmol). Column chromatography (hexane/EtOAc 50 : 1, *R*_f_ = 0.14) gave 0.090 g (72%, *E*/*Z* = 71 : 29) of the title compound as a yellowish oil. ^1^H NMR (300 MHz, CDCl_3_) *δ* 7.27–7.12 (m, 3H, *E* and *Z*), 6.96–6.36 (m, 10H, *E* and *Z*), 5.80 (t, *J* = 6.0 Hz, 1H, *Z*^minor^), 5.49 (t, *J* = 6.0 Hz, 1H), 3.71 (s, 3H, *E*^major^), 3.68 (s, 3H, *Z*^minor^), 3.62 (s, 3H, *Z*^minor^), 3.60 (s, 3H, *E*^major^), 3.59 (s, 3H, *Z*^minor^), 3.44 (s, 3H, *E*^major^), 2.24 (q, *J* = 7.4 Hz, 2H, *E*^major^), 1.78 (q, *J* = 7.4 Hz, 2H, *Z*^minor^), 1.46–1.40 (m, 2H, *E*^major^), 1.25–1.22 (m, 2H, *Z*^minor^), 0.95 (t, *J* = 7.3 Hz, 3H, *E*^major^), 0.95 (t, *J* = 7.3 Hz, 3H, *Z*^minor^); ^13^C NMR (100 MHz, CDCl_3_) *δ* 157.4, 156.3, 156.1, 136.4, 136.1, 134.8, 134.0, 133.9, 132.3, 130.9, 130.8, 130.3, 129.7, 127.7, 127.4, 127.0, 120.3, 119.5, 119.0, 112.1, 109.6, 109.4, 55.9, 55.1, 54.7, 31.3^minor^, 31.1^major^, 22.7^major^, 22.6^minor^, 14.0^major^, 13.9^minor^; HRMS (APCI) *m*/*z*: calcd for C_28_H_30_O_3_ [M + H]^+^ 415.2268; found 415.2277.

##### 1,1,3-Tris(2-thienyl)hepta-1,3-diene (3k)

Prepared according to the GP3 from thiophene (0.108 g, 1.28 mmol), ^*n*^BuLi (0.51 mL, 2.40 M, 1.22 mmol), dry THF (3 mL). The reaction mixture was stirred for 30 min at 0 °C followed by addition of enol phosphate 2d (0.110 g, 0.31 mmol). Column chromatography (hexane, *R*_f_ = 0.14) gave 0.048 g (45%, *E*/*Z* = 93 : 7) of the title compound as a greenish oil. ^1^H NMR (300 MHz, CDCl_3_) *δ* 7.28–7.26 (m, 1H), 7.23–7.21 (m, 1H), 7.13–7.12 (m, 1H), 7.09–7.02 (m, 3H), 6.97–6.87 (m, 3H), 6.69 (d, *J* = 1.2 Hz, 1H, *E*^major^), 5.92 (td, *J* = 7.4, 1.3 Hz, 1H, *E*^major^), 5.92 (td, *J* = 7.4, 1.3 Hz, 1H, *Z*^minor^), 2.24–2.18 (m, 2H, *Z*^minor^), 2.07–2.01 (m, 2H, *E*^major^), 1.33–1.23 (m, 2H), 0.87 (t, *J* = 7.4 Hz, 3H, *E*^major^), 0.79 (t, *J* = 7.4 Hz, 3H, *Z*^minor^); ^13^C NMR (100 MHz, CDCl_3_) *δ* 146.9^minor^, 145.9, 145.3, 141.2^minor^, 140.2^minor^, 140.1, 136.2, 131.3, 130.6, 128.5, 127.3, 127.1, 126.5, 126.4, 126.2, 125.1, 125.1, 123.7, 123.3, 31.7^major^, 31.6^minor^, 22.6^minor^, 22.2^major^, 14.0^major^, 13.8^minor^; HRMS (APCI) *m*/*z*: calcd for C_19_H_18_S_3_ [M + H]^+^ 343.0643; found 343.0649.

#### General procedure for conversion of phosphorodiamidates 2e–2k to α,β-ketones 4,5 (GP4)

Organolithium or Grignard reagent (2.5 equiv.) was added to a solution of phosphordiamidate 2e–2k in dry THF (10 mL/1 mmol) cooled to −78 °C. The resultant mixture was warmed to 23 °C and stirred for 2 h at 23 °C. Then the reaction mixture was quenched by phosphoric acid (1 mL/1 mmol, 85 wt%) and the mixture was stirred for 1 h at 60 °C. Then the reaction mixture was cooled to 23 °C and the mixture was extracted with ether (2 × 4 mL mmol^−1^). The combined organic phases were washed with water (1 × 4 mL mmol^−1^), aqueous solution of saturated sodium bicarbonate (1 × 4 mL mmol^−1^) and brine (1 × 4 mL mmol^−1^). The organic layer was dried over MgSO_4_, the solvents were removed under reduce pressure and column chromatography (Silica gel) gave the product.

##### 4,4-Diphenylbut-3-en-2-one (4a)

Prepared according to the GP4 from phosphordiamidate 2e (0.130 g, 0.5 mmol) and phenylmagnesium chloride (0.70 mL, 1.3 mmol). Column chromatography (hexane/AcOEt 9 : 1, *R*_f_ = 0.42) gave 0.087 g (78%) of the title compound as a yellowish oil. ^1^H NMR (300 MHz, CDCl_3_) *δ* 7.43–7.39 (m, 3H), 7.36–7.28 (m, 5H), 7.25–7.20 (m, 2H), 6.58 (s, 1H), 1.88 (s, 3H), in accordance with literature.^31^

##### 4,4-Bis(4-chlorophenyl)but-3-en-2-one (4b)

Prepared according to the GP4 from phosphordiamidate 2e (0.130 g, 0.5 mmol) and 4-chlorophenylmagnesium chloride (1.30 mL, 1.3 mmol). Column chromatography (hexane/AcOEt 9 : 1, *R*_f_ = 0.24) gave 0.116 g (80%) of the title compound as a yellowish oil. ^1^H NMR (400 MHz, CDCl_3_) *δ* 7.40–7.38 (m, 2H), 7.32–7.30 (m, 2H), 7.21–7.19 (m, 2H), 7.15–7.12 (m, 2H), 6.56 (s, 1H), 1.99 (s, 3H); ^13^C NMR (101 MHz, CDCl_3_) *δ* 199.3, 151.5, 139.1, 137.0, 136.0, 135.2, 131.1, 129.8, 129.0, 128.9, 127.7, 31.0; HRMS (APCI) *m*/*z*: calcd for C_16_H_12_Cl_2_O_1_ [M + H]^+^ 291.0338; found 291.0329.

##### 4,4-Bis(4-fluorophenyl)but-3-en-2-one (4c)

Prepared according to the GP4 from phosphordiamidate 2e (0.130 g, 0.5 mmol) and 4-fluorophenylmagnesium chloride (1.30 mL, 1.3 mmol). Column chromatography (hexane/AcOEt 9 : 1, *R*_f_ = 0.33) gave 0.105 g (81%) of the title compound as a yellowish oil. ^1^H NMR (400 MHz, CDCl_3_) *δ* 7.28–7.24 (m, 2H), 7.20–7.17 (m, 2H), 7.13–7.09 (m, 2H), 7.05–7.01 (m, 2H), 6.53 (s, 1H), 1.96 (s, 3H); ^13^C NMR (101 MHz, CDCl_3_) *δ* 199.5, 163.6 (d, *J* = 251.5 Hz), 163.1 (d, *J* = 250.5 Hz), 151.8, 136.8 (d, *J* = 3.1 Hz), 134.6 (d, *J* = 3.5 Hz), 131.5 (d, *J* = 8.1 Hz), 130.4 (d, *J* = 9.1 Hz), 127.2 (d, *J* = 6.1 Hz), 115.7 (d, *J* = 5.1 Hz), 115.5 (d, *J* = 6.1 Hz), 30.6; HRMS (APCI) *m*/*z*: calcd for C_16_H_12_F_2_O_1_ [M + H]^+^ 259.0929; found 259.0929.

##### 4,4-Bis(3-methoxyphenyl)but-3-en-2-one (4d)

Prepared according to the GP4 from phosphordiamidate 2e (0.130 g, 0.5 mmol) and 3-methoxyphenylmagnesium bromide (1.30 mL, 1.3 mmol). Column chromatography (hexane/AcOEt 6 : 1, *R*_f_ = 0.32) gave 0.112 g (79%) of the title compound as a yellowish oil. ^1^H NMR (400 MHz, CDCl_3_) *δ* 7.34–7.30 (m, 1H), 7.26–7.22 (m, 1H), 6.97–6.80 (m, 5H), 6.75–6.74 (m, 1H), 6.56 (s, 1H), 3.80 (s, 3H), 3.78 (s, 3H), 1.89 (s, 3H); ^13^C NMR (101 MHz, CDCl_3_) *δ* 200.4, 159.6, 159.5, 153.5, 142.0, 140.2, 129.5, 129.4, 128.0, 122.1, 121.0, 115.0, 114.9, 114.4, 114.0, 55.3, 30.2; HRMS (APCI) *m*/*z*: calcd for C_18_H_18_O_3_ [M + H]^+^ 283.1329; found 283.1324.

##### 4,4-Bis(3-(methylthio)phenyl)but-3-en-2-one (4e)

Prepared according to the GP4 from phosphordiamidate 2e (0.130 g, 0.5 mmol) and (3-(methylthio)phenyl)magnesium bromide (2.60 mL, 1.3 mmol). Column chromatography (hexane/AcOEt 6 : 1, *R*_f_ = 0.34) gave 0.126 g (80%) of the title compound as a yellowish oil. ^1^H NMR (400 MHz, CDCl_3_) *δ* 7.35–7.24 (m, 3H), 7.19–7.18 (m, 1H), 7.07–7.06 (m, 1H), 7.03–6.96 (m, 3H), 6.55 (s, 1H), 2.47 (s, 3H), 2.45 (s, 3H), 1.92 (s, 3H); ^13^C NMR (101 MHz, CDCl_3_) *δ* 200.1, 152.9, 141.1, 139.2, 139.2, 139.1, 128.9, 128.8, 128.2, 127.4, 127.1, 126.7, 126.2, 126.1, 125.3, 30.4, 15.7, 15.6; HRMS (APCI) *m*/*z*: calcd for C_18_H_18_OS_2_ [M + H]^+^ 315.0872; found 315.0879.

##### 4,4-Di-*o*-tolylbut-3-en-2-one (4f)

Prepared according to the GP4 from phosphordiamidate 2e (0.130 g, 0.5 mmol) and *o*-tolylmagnesium chloride (1.30 mL, 1.3 mmol). Column chromatography (hexane/AcOEt 6 : 1, *R*_f_ = 0.49) gave 0.094 g (75%) of the title compound as a yellowish solid, mp = 62.0–65.0 °C. ^1^H NMR (400 MHz, CDCl_3_) *δ* 7.28–7.03 (m, 8H), 6.31 (s, 1H), 2.35 (s, 3H), 2.10 (s, 3H), 1.85 (s, 3H); ^13^C NMR (101 MHz, CDCl_3_) *δ* 200.2, 153.8, 140.3, 139.4, 135.7, 135.6, 132.3, 131.3, 130.6, 130.1, 129.9, 128.7, 128.4, 125.8, 125.8, 29.9, 21.0, 19.9; HRMS (APCI) *m*/*z*: calcd for C_18_H_18_O [M + H]^+^ 251.1430; found 251.1432.

##### 4-Octyldodec-3-en-2-one (4g)

Prepared according to the GP4 from phosphordiamidate 2e (0.13 g, 0.5 mmol) and octylmagnesium chloride (0.6 mL, 1.3 mmol). Column chromatography (hexane/AcOEt 6 : 1, *R*_f_ = 0.41) gave 0.124 g (84%) of the title compound as a yellowish oil. ^1^H NMR (400 MHz, CDCl_3_) *δ* 6.01 (s, 1H), 2.55–2.51 (m, 2H), 2.16 (s, 3H), 2.12–2.08 (m, 2H), 1.46–1.25 (m, 24H), 0.90–0.85 (m, 6H); ^13^C NMR (101 MHz, CDCl_3_) *δ* 198.4, 163.8, 122.9, 38.7, 32.4, 31.92, 31.88, 31.85, 30.0, 29.44, 29.42, 29.3, 29.2, 28.6, 27.8, 22.67, 22.66, 14.12, 14.11; HRMS (APCI) *m*/*z*: calcd for C_20_H_38_O [M + H]^+^ 295.2995; found 295.2984.

##### 1,1-Diphenylhex-1-en-3-one (4h)

Prepared according to the GP4 from phosphordiamidate 2f (0.149 g; 0.51 mmol) and phenylmagnesium chloride (0.65 mL, 1.28 mmol). Column chromatography (hexane/AcOEt 20 : 1, *R*_f_ = 0.23) gave 0.101 g (79%) of the title compound as a yellowish oil. ^1^H NMR (300 MHz, CDCl_3_) *δ* 7.41–7.29 (m, 8H), 7.21–7.18 (m, 2H), 6.58 (s, 1H), 2.22 (t, *J* = 7.3 Hz, 2H), 1.59–1.46 (m, 2H), 0.79 (t, *J* = 7.4 Hz, 3H), in accordance with literature.^32^

##### 1,1-Bis(4-chlorophenyl)hex-1-en-3-one (4i)

Prepared according to the GP4 from phosphordiamidate 2f (0.146 g; 0.50 mmol) and 4-chlorophenylmagnesium chloride (1.30 mL, 1.25 mmol). Column chromatography (hexane/AcOEt 20 : 1, *R*_f_ = 0.31) gave 0.121 g (76%) of the title compound as a yellowish oil. ^1^H NMR (300 MHz, CDCl_3_) *δ* 7.37–7.35 (m, 2H), 7.32–7.29 (m, 2H), 7.21–7.19 (m, 2H), 7.12–7.10 (m, 2H), 6.57 (s, 1H), 2.33 (t, *J* = 7.3 Hz, 2H), 1.61–1.51 (m, 2H), 0.85 (t, *J* = 7.4 Hz, 3H); ^13^C NMR (100 MHz, CDCl_3_) *δ* 201.2, 150.8, 139.1, 136.9, 135.6, 134.7, 130.8, 129.6, 128.7, 128.6, 126.4, 45.6, 17.6, 13.7; HRMS (APCI) *m*/*z*: calcd for C_18_H_16_Cl_2_O [M + H]^+^ 319.0651; found 319.0650.

##### 1,1-Bis(4-fluorophenyl)hex-1-en-3-one (4j)

Prepared according to the GP4 from phosphordiamidate 2f (0.146 g; 0.50 mmol) and 4-fluorophenylmagnesium bromide (1.30 mL, 1.25 mmol). Column chromatography (hexane/AcOEt 20 : 1, *R*_f_ = 0.24) gave 0.110 g (77%) of the title compound as a yellowish oil. ^1^H NMR (300 MHz, CDCl_3_) *δ* 7.27–7.24 (m, 2H), 7.18–7.14 (m, 2H), 7.10–7.00 (m, 4H), 6.53 (s, 1H), 2.30 (t, *J* = 7.3 Hz, 2H), 1.60–1.51 (m, 2H), 0.84 (t, *J* = 7.3 Hz, 3H); ^13^C NMR (100 MHz, CDCl_3_) *δ* 201.5, 163.5 (d, *J* = 250.4 Hz), 162.9 (d, *J* = 248.6 Hz) 151.2, 137.1 (d, *J* = 3.5 Hz), 134.6 (d, *J* = 3.5 Hz), 131.3 (d, *J* = 8.3 Hz), 130.2 (d, *J* = 8.4 Hz), 126.1, 115.5 (d, *J* = 10.9 Hz), 115.3 (d, *J* = 10.8 Hz), 45.5, 17.6, 13.7; HRMS (APCI) *m*/*z*: calcd for C_18_H_16_F_2_O [M + H]^+^ 287.1242; found 287.1243.

##### 1,1-Bis(3-methoxyphenyl)hex-1-en-3-one (4k)

Prepared according to the GP4 from phosphordiamidate 2f (0.146 g; 0.50 mmol) and 3-methoxyphenylmagnesium bromide (1.30 mL, 1.25 mmol). Column chromatography (hexane/AcOEt 20 : 1, *R*_f_ = 0.18) gave 0.129 g (83%) of the title compound as a yellowish oil. ^1^H NMR (300 MHz, CDCl_3_) *δ* 7.32–7.22 (m, 2H), 6.95–6.71 (m, 6H), 6.55 (s, 1H), 3.78 (s, 3H), 3.77 (s, 3H), 2.21 (t, *J* = 7.3 Hz, 2H), 1.56–1.47 (m, 2H), 0.79 (t, *J* = 7.3 Hz, 3H); ^13^C NMR (75 MHz, CDCl_3_) *δ* 202.6, 159.4, 159.4, 152.4, 142.2, 140.2, 129.3, 127.0, 121.9, 120.9, 114.9, 114.5, 114.1, 114.0, 55.3, 55.2, 44.9, 17.8, 13.7; HRMS (APCI) *m*/*z*: calcd for C_20_H_22_O_3_ [M + H]^+^ 311.1642; found 311.1645.

##### 1,1-Bis(3-(methylthio)phenyl)hex-1-en-3-one (4l)

Prepared according to the GP4 from phosphordiamidate 2f (0.146 g; 0.50 mmol) and (3-(methylthio)phenyl)magnesium bromide (1.40 mL, 1.25 mmol). Column chromatography (hexane/AcOEt 30 : 1, *R*_f_ = 0.11) gave 0.122 g (71%) of the title compound as a yellowish oil. ^1^H NMR (300 MHz, CDCl_3_) *δ* 7.30–7.18 (m, 5H), 7.05–6.94 (m, 3H), 6.54 (s, 1H), 2.46 (s, 3H), 2.46 (s, 3H), 2.45 (s, 3H), 2.24 (t, *J* = 7.3 Hz, 2H), 1.57–1.48 (m, 2H), 0.81 (t, *J* = 7.4 Hz, 3H); ^13^C NMR (75 MHz, CDCl_3_) *δ* 202.2, 151.9, 141.4, 139.3, 138.9, 138.8, 128.8, 128.6, 127.2, 127.16, 127.13, 126.5, 126.2, 126.1, 125.2, 45.1, 17.7, 15.7, 15.7, 13.7; HRMS (APCI) *m*/*z*: calcd for C_20_H_22_OS_2_ [M + H]^+^ 343.1185; found 343.1188.

##### 1,1-Di-*o*-tolylhex-1-en-3-one (4m)

Prepared according to the GP4 from phosphordiamidate 2f (0.146 g; 0.50 mmol) and *o*-tolylmagnesium chloride (1.30 mL, 1.25 mmol). Column chromatography (hexane/AcOEt 30 : 1, *R*_f_ = 0.28) gave 0.103 g (74%) of the title compound as a yellowish oil. ^1^H NMR (300 MHz, CDCl_3_) *δ* 7.26–7.06 (m, 8H), 6.33 (s, 1H), 2.33 (s, 3H), 2.19 (t, *J* = 7.3 Hz, 2H), 2.10 (s, 3H), 1.54–1.49 (m, 2H), 0.79 (t, *J* = 7.4 Hz, 3H); ^13^C NMR (75 MHz, CDCl_3_) *δ* 202.3, 152.7, 140.7, 139.5, 135.7, 135.6, 131.2, 131.1, 130.4, 129.8, 129.8, 128.3, 128.2, 125.7, 125.6, 44.8, 20.9, 19.9, 17.7, 13.7; HRMS (APCI) *m*/*z*: calcd for C_20_H_22_O [M + H]^+^ 279.1743; found 279.1746.

##### 1,1-Di(thiophen-2-yl)hex-1-en-3-one (4n)

Prepared according to the GP4 from phosphordiamidate 2f (0.146 g; 0.50 mmol) and 2-thienylmagnesium bromide (1.30 mL, 1.26 mmol). Column chromatography (hexane/AcOEt 20 : 1, *R*_f_ = 0.38) gave 0.098 g (75%) of the title compound as a brownish oil. ^1^H NMR (300 MHz, CDCl_3_) *δ* 7.48–7.46 (m, 1H), 7.38–7.36 (m, 1H), 7.15–7.12 (m, 1H), 7.10–7.08 (m, 2H), 7.03–7.00 (m, 1H), 6.61 (d, *J* = 1.7 Hz, 1H), 2.28 (td, *J* = 7.3, 1.8 Hz, 2H), 1.62–1.49 (m, 2H), 0.83 (td, *J* = 7.4, 1.8 Hz, 3H); ^13^C NMR (75 MHz, CDCl_3_) *δ* 201.2, 145.0, 138.6, 138.2, 129.9, 129.6, 128.1, 127.9, 127.7, 126.9, 125.5, 44.9, 17.9, 13.7; HRMS (APCI) *m*/*z*: calcd for C_14_H_14_OS_2_ [M + H]^+^ 263.0559; found 263.0551.

##### 2-Benzyl-1-phenylhept-2-en-4-one (4o)

Prepared according to the GP4 from phosphordiamidate 2f (0.146 g; 0.50 mmol) and benzylmagnesium chloride (1.30 mL, 1.26 mmol). Column chromatography (hexane/AcOEt 50 : 1, *R*_f_ = 0.28) gave 0.088 g (63%) of the title compound as a yellowish oil. ^1^H NMR (300 MHz, CDCl_3_) *δ* 7.33–7.22 (m, 8H), 7.12–7.09 (m, 2H), 6.09 (s, 1H), 3.94 (s, 2H), 3.33 (s, 2H), 2.46 (t, *J* = 7.3 Hz, 2H), 1.68–1.62 (m, 2H), 0.94 (t, *J* = 7.4 Hz, 3H); ^13^C NMR (100 MHz, CDCl_3_) *δ* 201.3, 157.6, 138.9, 137.8, 129.3, 129.1, 128.5, 128.4, 126.6, 126.2, 125.6 46.5, 43.5, 36.8, 17.5, 13.8; HRMS (APCI) *m*/*z*: calcd for C_20_H_22_O [M + H]^+^ 279.1743; found 279.1738.

##### 6-Octyltetradec-5-en-4-one (4p)

Prepared according to the GP4 from phosphordiamidate 2f (0.148 g; 0.51 mmol) and octylmagnesium chloride (0.63 mL, 1.26 mmol). Column chromatography (hexane/AcOEt 30 : 1, *R*_f_ = 0.44) gave 0.147 g (90%) of the title compound as a colorless oil. ^1^H NMR (300 MHz, CDCl_3_) *δ* 5.99 (s, 1H), 2.55–2.51 (m, 2H), 2.39 (t, *J* = 7.3 Hz, 2H), 2.12–2.08 (m, 2H), 1.64–1.58 (m, 2H), 1.44–1.25 (m, 24H), 0.94–0.86 (m, 9H); ^13^C NMR (75 MHz, CDCl_3_) *δ* 200.9, 163.3, 122.6, 46.4, 38.7, 32.5, 31.9, 31.8, 30.0, 29.5, 29.4, 29.4, 29.3, 29.2, 28.7, 27.8, 22.7, 22.6, 17.7, 14.1, 13.8; HRMS (APCI) *m*/*z*: calcd for C_22_H_42_O [M + H]^+^ 323.3308; found 323.3310.

##### 1,3,3-Triphenylprop-2-en-1-one (4q)

Prepared according to the GP4 from phosphordiamidate 2g (0.160 g; 0.50 mmol) and phenylmagnesium chloride (0.70 mL, 1.30 mmol). Column chromatography (hexane/AcOEt 9 : 1, *R*_f_ = 0.32) gave 0.129 g (97%) of the title compound as a yellow oil. ^1^H NMR (400 MHz, CDCl_3_) *δ* 7.92–7.90 (m, 2H), 7.48–7.46 (m, 1H), 7.41–7.36 (m, 7H), 7.28–7.26 (m, 3H), 7.19–7.12 (m, 2H), 7.12 (s, 1H), in accordance with literature.^33^

##### 1-Phenyl-3,3-di-*o*-tolylprop-2-en-1-one (4r)

Prepared according to the GP4 from phosphordiamidate 2g (0.160 g; 0.50 mmol) and 2-tolylmagnesium chloride (1.30 mL, 1.30 mmol). Column chromatography (hexane/AcOEt 9 : 1, *R*_f_ = 0.35) gave 0.144 g (95%) of the title compound as a yellow oil. ^1^H NMR (400 MHz, CDCl_3_) *δ* 7.90–7.88 (m, 2H), 7.50–7.46 (m, 1H), 7.40–7.36 (m, 2H), 7.25–7.06 (m, 8H), 6.98 (s, 1H), 2.36 (s, 3H), 2.09 (s, 3H); ^13^C NMR (101 MHz, CDCl_3_) *δ* 192.0, 154.9, 141.1, 139.6, 138.3, 135.8, 135.7, 132.6, 131.2, 130.3, 129.9, 129.6, 128.5, 128.4, 128.3, 128.0, 128.0, 125.9, 125.5, 21.0, 20.1; HRMS (APCI) *m*/*z*: calcd for C_23_H_20_O [M + H]^+^ 313.1587; found 313.1591.

##### 2-(Diphenylmethylene)cyclohexan-1-one (5a)

Prepared according to the GP4 from bromobenzene (0.196 g, 1.25 mmol), ^*n*^BuLi (0.50 mL, 1.20 mmol), phosphordiamidate 2h (0.152 g, 0.50 mmol). Column chromatography (hexane/AcOEt 20 : 1, *R*_f_ = 0.17) gave 0.088 g, (67%) of the title compound as a white solid, mp = 130.0–131.0 °C. ^1^H NMR (400 MHz, CDCl_3_) *δ* 7.34–7.22 (m, 6H), 7.14–7.11 (m, 2H), 7.06–7.04 (m, 2H), 2.65–2.59 (m, 4H), 2.01–1.96 (m, 2H), 1.83–1.78 (m, 2H); ^13^C NMR (100 MHz, CDCl_3_) *δ* 206.9, 144.2, 142.2, 140.8, 138.9, 129.9, 129.0, 128.0, 127.7, 127.3, 45.0, 34.1, 26.6, 26.3; HRMS (APCI) *m*/*z*: calcd for C_19_H_18_O [M + H]^+^ 263.1430; found 263.1434.

##### 2-(Diphenylmethylene)cyclopentan-1-one (5b)

Prepared according to the GP4 from bromobenzene (0.198 g, 1.26 mmol), ^*n*^BuLi (0.50 mL, 1.20 mmol), phosphordiamidate 2i (0.138 g, 0.50 mmol). Column chromatography (hexane/AcOEt 20 : 1, *R*_f_ = 0.08) gave 0.085 g (68%) of the title compound as a yellow oil. ^1^H NMR (300 MHz, CDCl_3_) *δ* 7.34–7.31 (m, 6H), 7.21–7.17 (m, 2H), 7.14–7.10 (m,2H), 2.82 (t, *J* = 7.0 Hz, 2H), 2.38 (t, *J* = 7.7 Hz, 2H), 1.98–1.88 (m, 2H), in accordance with literature.^34^

##### 2-(Bis(4-methoxyphenyl)methylene)cyclopentan-1-one (5c)

Prepared according to the GP4 from 4-bromoanisole (0.237 g, 1.26 mmol), ^*n*^BuLi (0.50 mL, 1.20 mmol), phosphordiamidate 2i (0.140 g, 0.51 mmol). Column chromatography (hexane/AcOEt 9 : 1, *R*_f_ = 0.15) gave 0.115 g (74%) of the title compound as a yellow oil. ^1^H NMR (400 MHz, CDCl_3_) *δ* 7.14–7.12 (m, 2H), 7.06–7.04 (m, 2H), 6.86–6.83 (m, 4H), 3.82 (s, 6H), 2.81 (t, *J* = 7.0 Hz, 2H), 2.37 (t, *J* = 7.7 Hz, 2H), 1.91 (p, *J* = 7.3 Hz, 2H); ^13^C NMR (100 MHz, CDCl_3_) *δ* 206.6, 159.7, 159.4, 148.2, 134.6, 132.6, 132.5, 131.6, 131.3, 113.2, 113.1, 55.3, 55.1, 40.0, 33.5, 20.7; HRMS (APCI) *m*/*z*: calcd for C_20_H_20_O_3_ [M + H]^+^ 309.1485; found 309.1489.

##### 2-(Diphenylmethylene)cycloheptan-1-one (5d)

Prepared according to the GP4 from bromobenzene (0.200 g, 1.28 mmol), ^*n*^BuLi (0.51 mL, 1.22 mmol), phosphordiamidate 2j (0.160 g, 0.52 mmol). Column chromatography (hexane/AcOEt 20 : 1, *R*_f_ = 0.15) gave 0.120 g (83%) of the title compound as a white solid, mp = 79.5–81.5 °C. ^1^H NMR (400 MHz, CDCl_3_) *δ* 7.37–7.19 (m, 8H), 7.11–7.08 (m, 2H), 2.50–2.47 (m, 2H), 2.39–2.36 (m, 2H), 1.92–1.88 (m, 2H), 1.72–1.61 (m, 4H); ^13^C NMR (100 MHz, CDCl_3_) *δ* 211.2, 142.5, 141.8, 141.5, 140.9, 128.9, 128.6, 128.3, 128.1, 127.22, 127.20, 43.4, 30.3, 29.3, 29.2, 24.2; HRMS (APCI) *m*/*z*: calcd for C_20_H_20_O [M + H]^+^ 277.1587; found 277.1586.

##### 2-(Bis(4-methoxyphenyl)methylene)cycloheptan-1-one (5e)

Prepared according to the GP4 from 4-bromoanisole (0.240 g, 1.28 mmol), ^*n*^BuLi (0.51 mL, 1.22 mmol), phosphordiamidate 2j (0.152 g, 0.50 mmol). Column chromatography (hexane/AcOEt 9 : 1, *R*_f_ = 0.24) gave 0.152 g (90%) of the title compound as a yellow oil. ^1^H NMR (400 MHz, CDCl_3_) *δ* 7.12–7.10 (m, 2H), 7.00–6.98 (m, 2H), 6.88–6.86 (m, 2H), 6.77–6.74 (m, 2H), 3.81 (s, 3H), 3.76 (s, 3H), 2.50–2.41 (m, 2H), 2.40–2.38 (m, 2H), 1.90–1.86 (m, 2H), 1.71–1.60 (m, 4H); ^13^C NMR (100 MHz, CDCl_3_) *δ* 211.7, 158.7, 158.7, 141.4, 141.4, 134.4, 133.6, 130.4, 13.99, 113.0, 113.4, 55.19, 55.1, 43.4, 30.5, 29.4, 29.3, 24.3; HRMS (APCI) *m*/*z*: calcd for C_22_H_24_O_3_ [M + H]^+^ 337.1798; found 337.1802.

##### 2-(Di(thiophen-2-yl)methylene)cycloheptan-1-one (5f)

Prepared according to the GP4 from thiophene (0.104 g, 1.23 mmol), ^*n*^BuLi (0.49 mL, 1.17 mmol), phosphordiamidate 2j (0.156 g, 0.51 mmol). Column chromatography (hexane/AcOEt 20 : 1, *R*_f_ = 0.28) gave 0.059 g (40%) of the title compound as an orange oil. ^1^H NMR (400 MHz, CDCl_3_) *δ* 7.36–7.35 (m, 1H), 7.26–7.24 (m, 1H), 7.04–6.99 (m, 2H), 6.93–6.88 (m, 2H), 2.53–2.46 (m, 4H), 1.86–1.67 (m, 6H); ^13^C NMR (100 MHz, CDCl_3_) *δ* 211.4, 145.1, 142.4, 141.6, 128.0, 127.8, 126.8, 126.7, 126.4, 126.3, 124.7, 42.7, 30.9, 28.4, 28.3, 23.7; HRMS (APCI) *m*/*z*: calcd for C_16_H_16_OS_2_ [M + H]^+^ 289.0715; found 289.0719.

##### 3-Methyl-4,4-diphenylbut-3-en-2-one (5g)

Prepared according to the GP4 from bromobenzene (0.20 g, 1.3 mmol), ^*n*^BuLi (0.52 mL, 1.25 mmol), phosphordiamidate 2k (0.140 g, 0.50 mmol). Column chromatography (hexane/AcOEt 20 : 1, *R*_f_ = 0.37) gave 0.084 g (71%) of the title compound as an yellowish oil. ^1^H NMR (400 MHz, CDCl_3_) *δ* 7.37–7.26 (m, 6H), 7.18–7.10 (m, 4H), 1.98 (s, 3H), 1.82 (s, 3H); ^13^C NMR (101 MHz, CDCl_3_) *δ* 207.9, 145.1, 141.7, 141.1, 137.7, 129.8, 129.7, 128.4, 128.3, 128.1, 127.7, 30.2, 18.7; HRMS (APCI) *m*/*z*: calcd for C_17_H_16_O [M + H]^+^ 237.1274; found 237.1266.

##### 4,4-Bis(4-methoxyphenyl)-3-methylbut-3-en-2-one (5h)

Prepared according to the GP4 from 4-bromoanisole (0.240 g, 1.3 mmol), ^*n*^BuLi (0.52 mL, 1.25 mmol), phosphordiamidate 2k (0.140 g, 0.50 mmol). Column chromatography (hexane/AcOEt 6 : 1, *R*_f_ = 0.55) gave 0.072 g (49%) of the title compound as a white solid, mp = 58.0–61.0 °C. ^1^H NMR (400 MHz, CDCl_3_) *δ* 7.10–7.07 (m, 2H), 7.02–7.00 (m, 2H), 6.88–6.80 (m, 4H), 3.82 (s, 3H), 3.81 (s, 3H), 2.00 (s, 3H), 1.80 (s, 3H); ^13^C NMR (101 MHz, CDCl_3_) *δ* 207.9, 160.0, 159.1, 145.6, 136.3, 134.6, 133.8, 131.4, 131.4, 113.7, 113.4, 55.3, 30.2, 19.0; HRMS (APCI) *m*/*z*: calcd for C_19_H_20_O_3_ [M + H]^+^ 297.1485; found 297.1489.

##### 3-Methyl-4,4-di(thiophen-2-yl)but-3-en-2-one (5i)

Prepared according to the GP4 from thiophene (0.110 g, 1.30 mmol), ^*n*^BuLi (0.52 mL, 1.25 mmol), phosphordiamidate 2k (0.140 g, 0.50 mmol). Column chromatography (hexane/AcOEt 9 : 1, *R*_f_ = 0.52) gave 0.029 g (24%) of the title compound as a yellow oil. ^1^H NMR (400 MHz, CDCl_3_) *δ* 7.41–7.39 (m, 2H), 7.06–7.05 (m, 2H), 6.99–6.98 (m, 2H), 2.17 (s, 3H), 1.91 (s, 3H); ^13^C NMR (101 MHz, CDCl_3_) *δ* 207.2, 143.2, 142.7, 139.8, 130.3, 129.9, 129.5, 128.5, 127.6, 127.2, 127.0, 29.5, 19.3; HRMS (APCI) *m*/*z*: calcd for C_13_H_12_OS_2_ [M + H]^+^ 249.0402; found 249.0406.

#### Phenyl(3,4,5,6-tetrahydro-[1,1′-biphenyl]-2-yl)methanone (6)

Phenylmagnesium chloride (0.78 mL, 1.53 mmol) was added to a mixture of CuI (0.029 g, 10 mol%) in dry ether (5 mL) cooled to 0 °C. The resultant mixture was stirred for 1 h at 0 °C followed by addition of phosporodiamidate 2h (0.156 g, 0.51 mmol). Then the mixture was stirred for 3 h at 23 °C. The crude reaction mixture was quenched with saturated aqueous solution of ammonium chloride (2 mL), diluted with ether (30 mL) and the organic layer was washed with water (30 mL) and brine (30 mL). The organic phase was dried over MgSO_4_ and the solvents were removed under reduce pressure. Column chromatography (Silica gel, hexane/AcOEt 20 : 1, *R*_f_ = 0.28) gave 0.062 g (46%) of the title compound as a white solid. ^1^H NMR (300 MHz, CDCl_3_) *δ* 7.69–7.66 (m, 2H), 7.32–7.31 (m, 1H), 7.23–7.19 (m, 2H), 7.09–6.99 (m, 5H), 2.53–2.45 (m, 4H), 1.91–1.82 (m, 4H), in accordance with literature.^35^

#### (*Z*)-4-Hydroxy-4,4-diphenylbut-2-en-2-yl bis(*N*,*N*-dimethylamino)phosphordiamidate (8)

Phenylmagnesium chloride (6.58 mL, 12.50 mmol) was added to a solution of phosphordiamidated 2e (1.320 g, 5.0 mmol) cooled to −78 °C. The resultant reaction mixture was stirred for 2 h at 23 °C. Then the reaction mixture was quenched with water (20 mL), the organic layer was separated and the water layer was extracted with ether (2 × 40 mL). Combined organic layers were washed with water (30 mL), brine (10 mL), dried over MgSO_4_ and concentrated under reduce pressure. Column chromatography (Silica gel, hexane/AcOEt 20 : 1, *R*_f_ = 0.53) gave 1.480 g (79%) of the title compound as a white solid, mp = 74.0–77.0 °C. ^1^H NMR (500 MHz, CDCl_3_) *δ* 7.59–7.57 (m, 4H), 7.30–7.27 (m, 4H), 7.18–7.15 (m, 2H), 6.22 (s, 1H), 5.74 (s, 1H), 2.42 (d, *J* = 10.1 Hz, 12H), 2.05 (s, 3H); ^13^C NMR (126 MHz, CDCl_3_) *δ* 148.8, 145.5 (d, *J* = 7.6 Hz), 128.1, 126.4, 126.2, 122.7 (d, *J* = 6.6 Hz), 75.8, 36.2 (d, *J* = 4.7 Hz), 21.8; ^31^P NMR (202 MHz, CDCl_3_) *δ* 15.12 (s); HRMS (ESI) *m*/*z*: calcd for C_20_H_27_N_2_O_3_P [M + Na]^+^ 397.1652; found 397.1660.

## Conflicts of interest

There are no conflicts to declare.

## Supplementary Material

RA-010-D0RA07472A-s001
