# Supplementary material for: Formation of trisubstituted buta-1,3-dienes and α,β-unsaturated ketones via the reaction of functionalized vinyl phosphates and vinyl phosphordiamidates with organometallic reagents
Source: RSC Adv. 2020 Sep 22;10(58):35109–20. doi: 10.1039/d0ra07472a (PMC9056830; doi:10.1039/d0ra07472a)
Supplement: RA-010-D0RA07472A-s001 [file RA-010-D0RA07472A-s001.pdf]

Electronic Supplementary Material (ESI) for RSC Advances.
This journal is © The Royal Society of Chemistry 2020

Formation of Trisubstituted Buta-1,3-dienes and α,β-Unsaturated Ketones via the Reaction of Functionalized Vinyl Phosphates and Vinyl Phosphordiamidates with Organometallic Reagents.

Petr Oeser, [a] Jakub Koudelka,[a] Hana Dvořáková[b] and Tomáš Tobrman*[c]

[a] Department of Organic Chemistry, University of Chemistry and Technology, Prague, Technická 5, 166 28 Prague 6, Czech Republic. Email: tomas.tobrman@vscht.cz
[b] Laboratory of NMR spectroscopy, University of Chemistry and Technology, Prague, Technická 5, 166 28 Prague 6, Czech Republic.

Supporting Information

Copies of ^{1}H and ^{13}C NMR spectra **S2-S63**

Copies of ^{1}H and ^{13}C NMR spectra

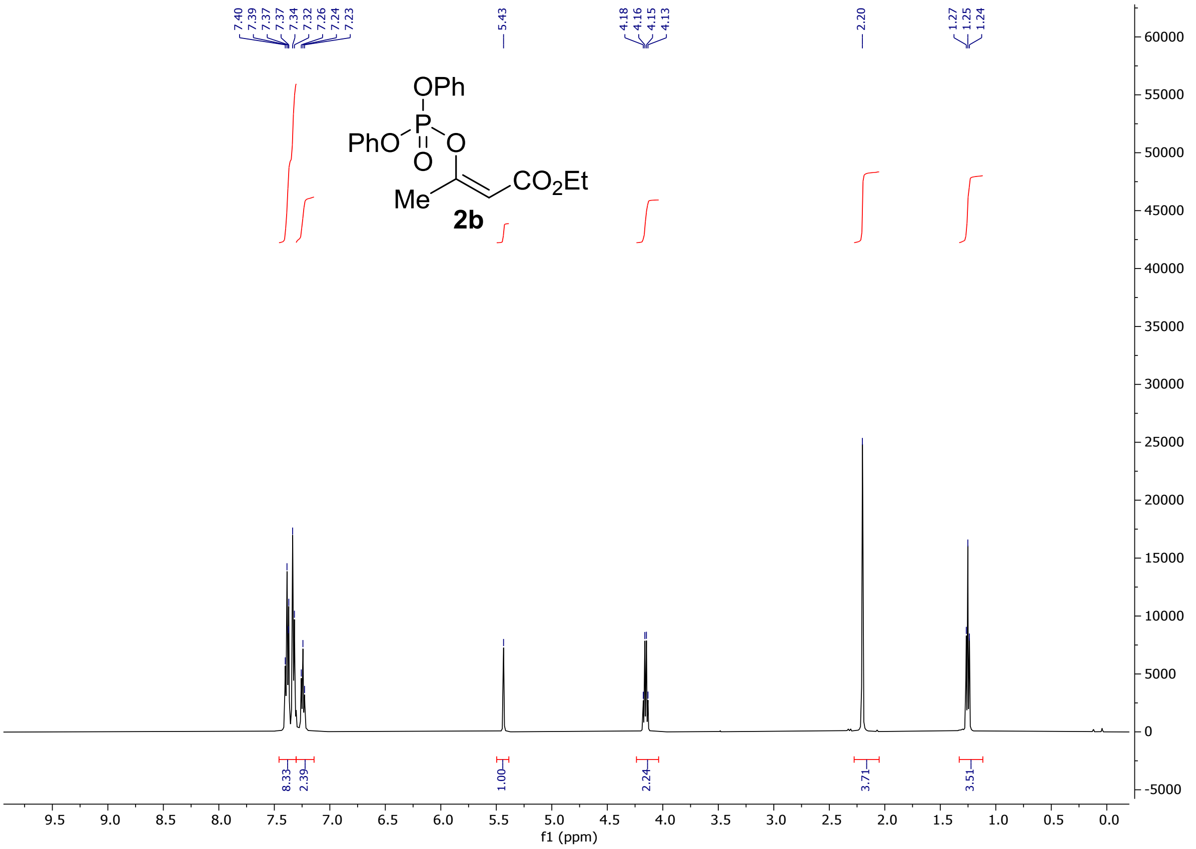

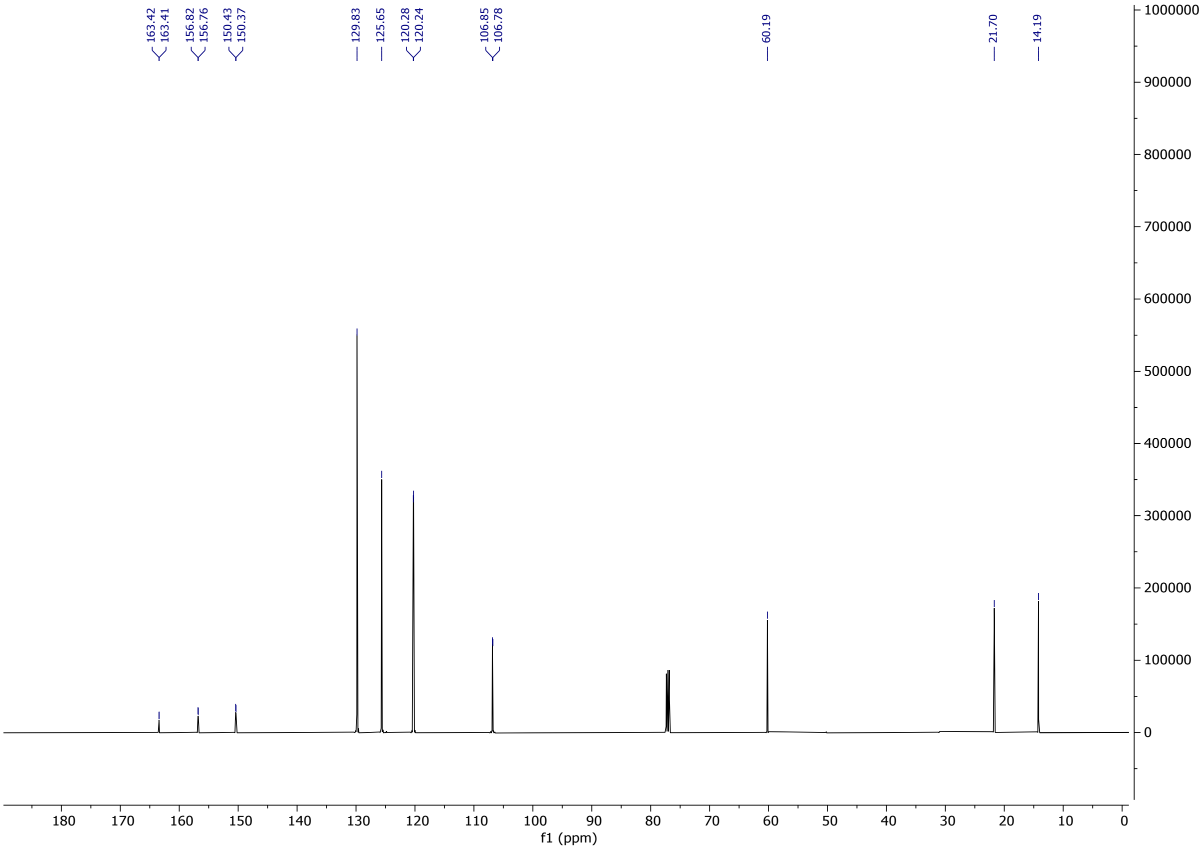

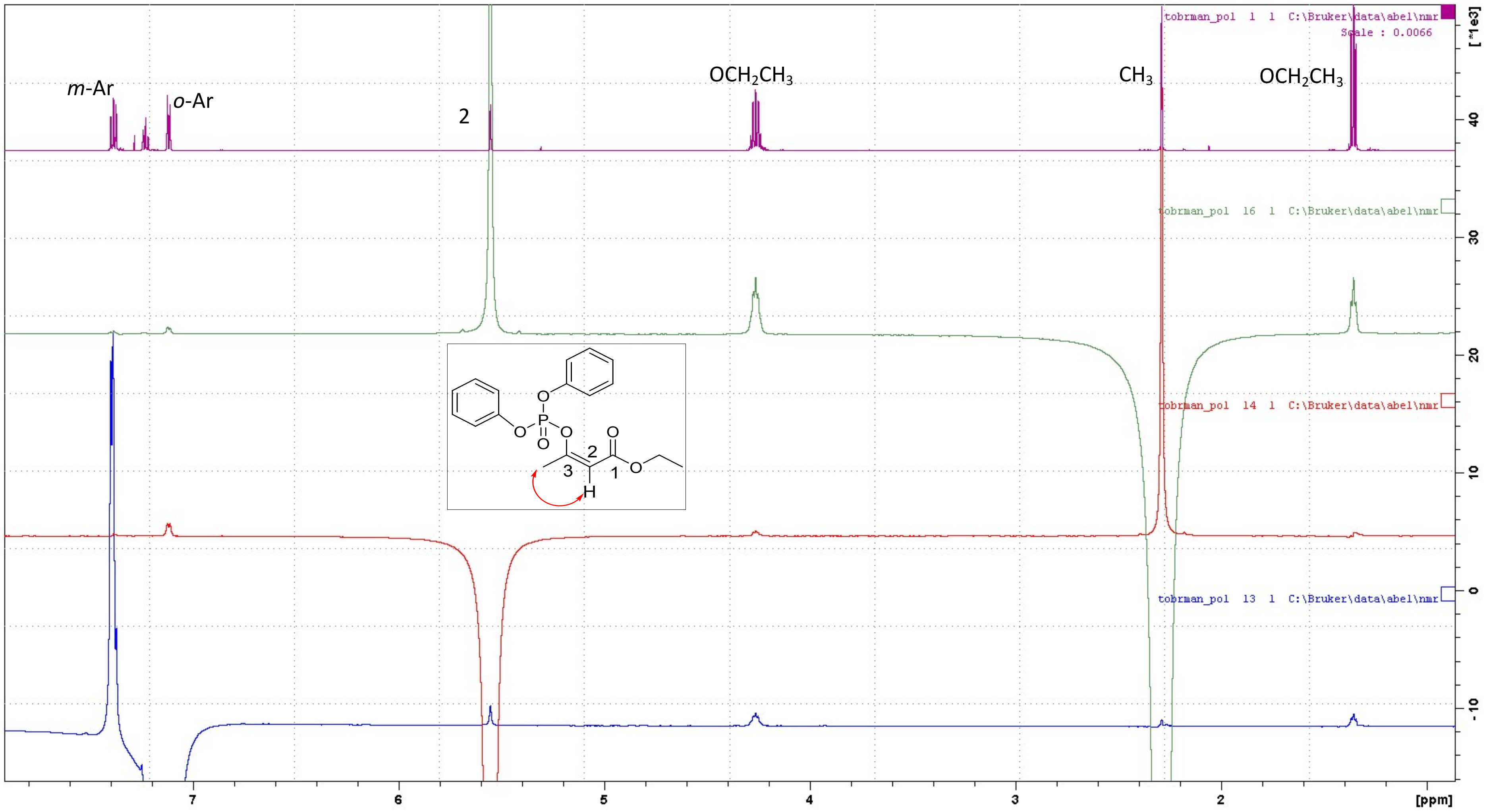
m-Ar
o-Ar
2
OCH2CH3
CH3
OCH2CH3
tobrman_pol 1 1 C:\Bruker\data\abel\nmr
Scale : 0.0066
tobrman_pol 16 1 C:\Bruker\data\abel\nmr
tobrman_pol 14 1 C:\Bruker\data\abel\nmr
tobrman_pol 13 1 C:\Bruker\data\abel\nmr
[*1e3]
[ppm]

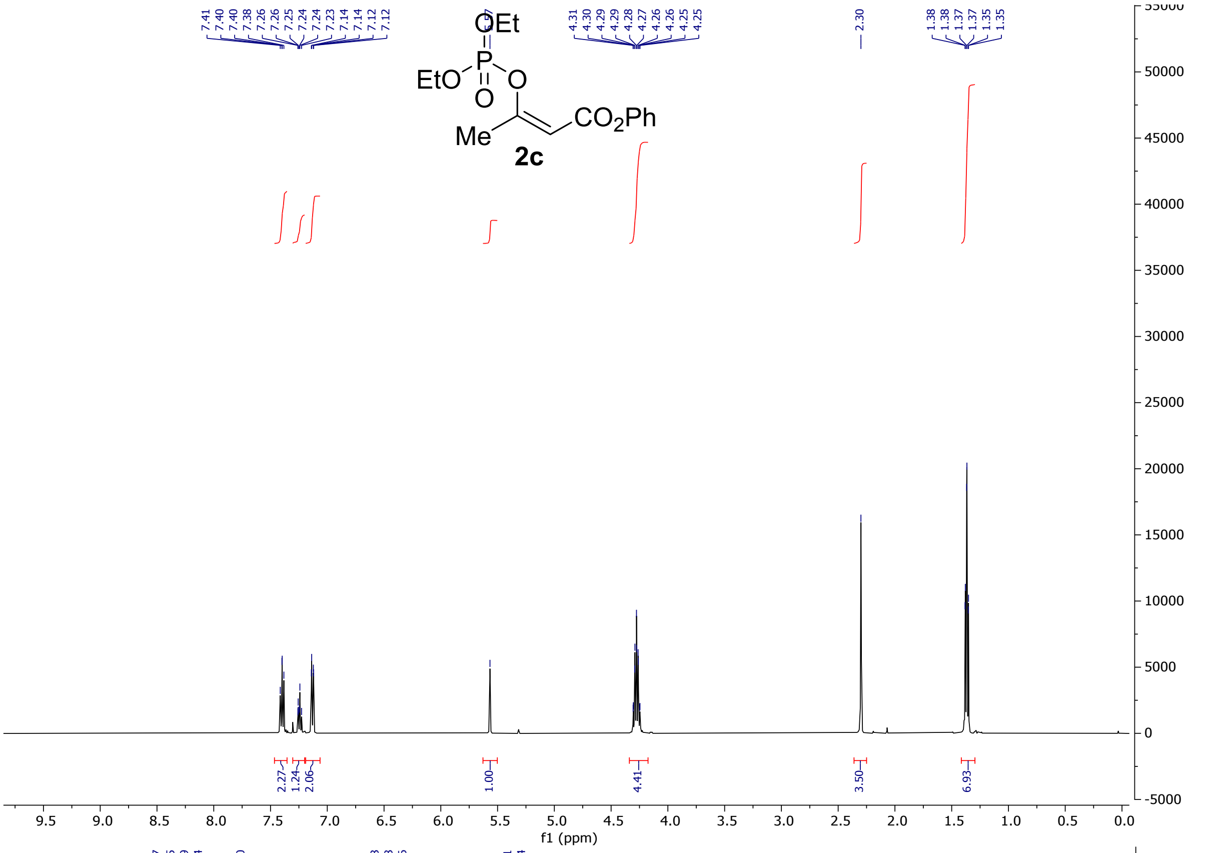
OEt
EtO
P
O
O
CO2Ph
Me
2c
f1 (ppm)

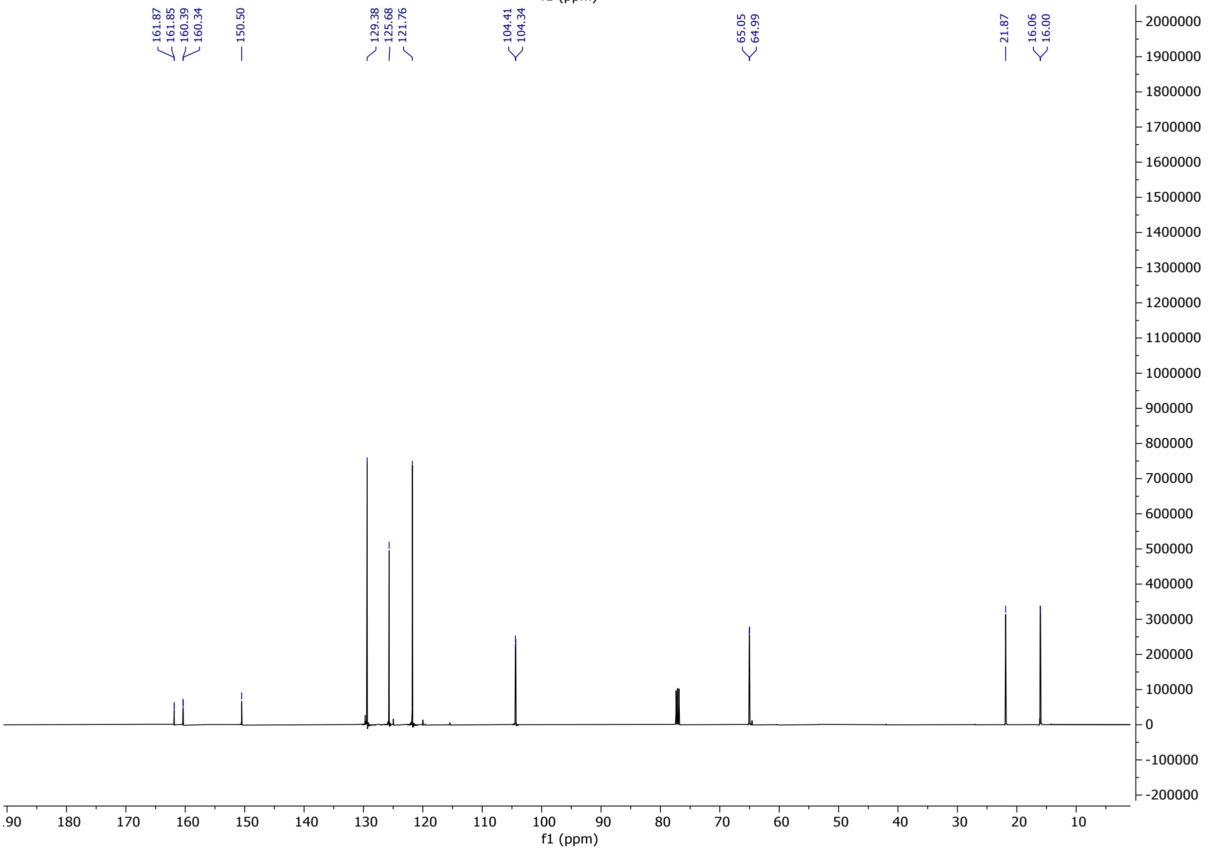
161.87
161.85
160.39
160.34
150.50
129.38
125.68
121.76
104.41
104.34
65.05
64.99
21.87
16.06
16.00
f1 (ppm)

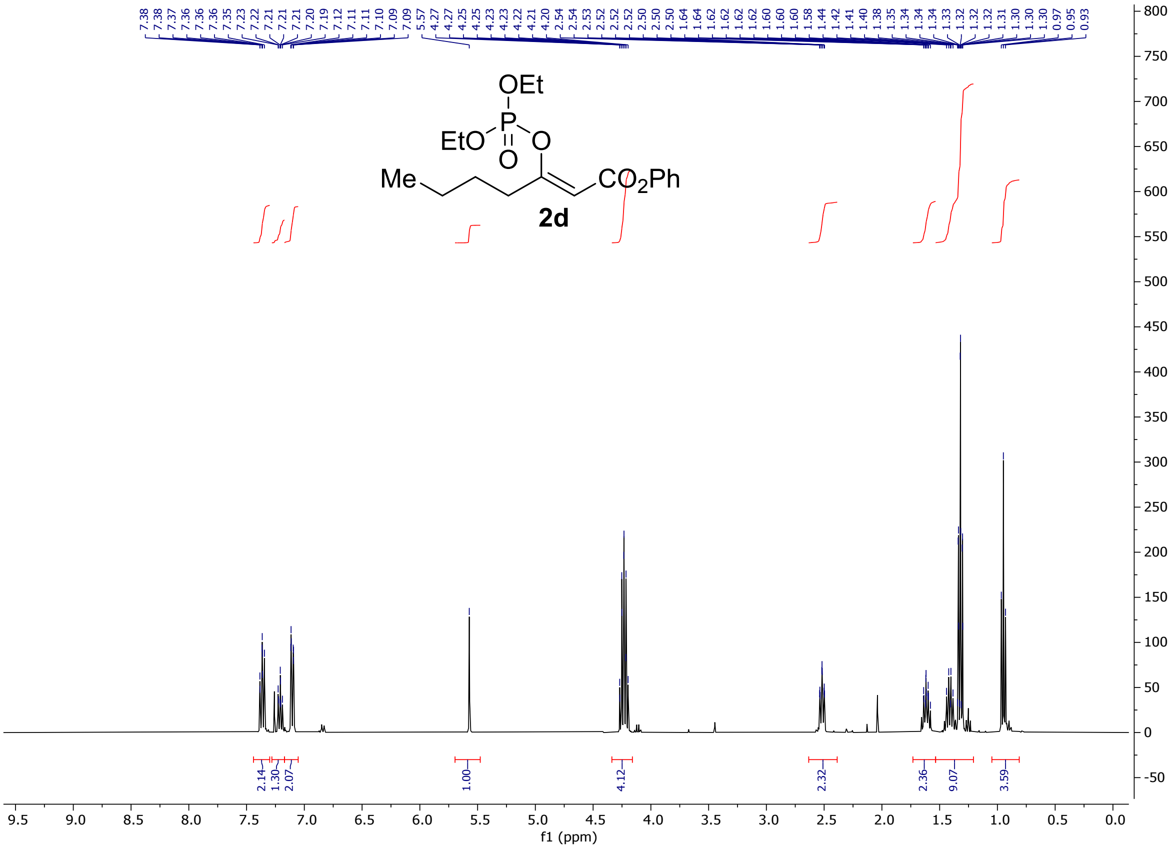
OEt
EtO
P
O
O
Me
CO2Ph
2d

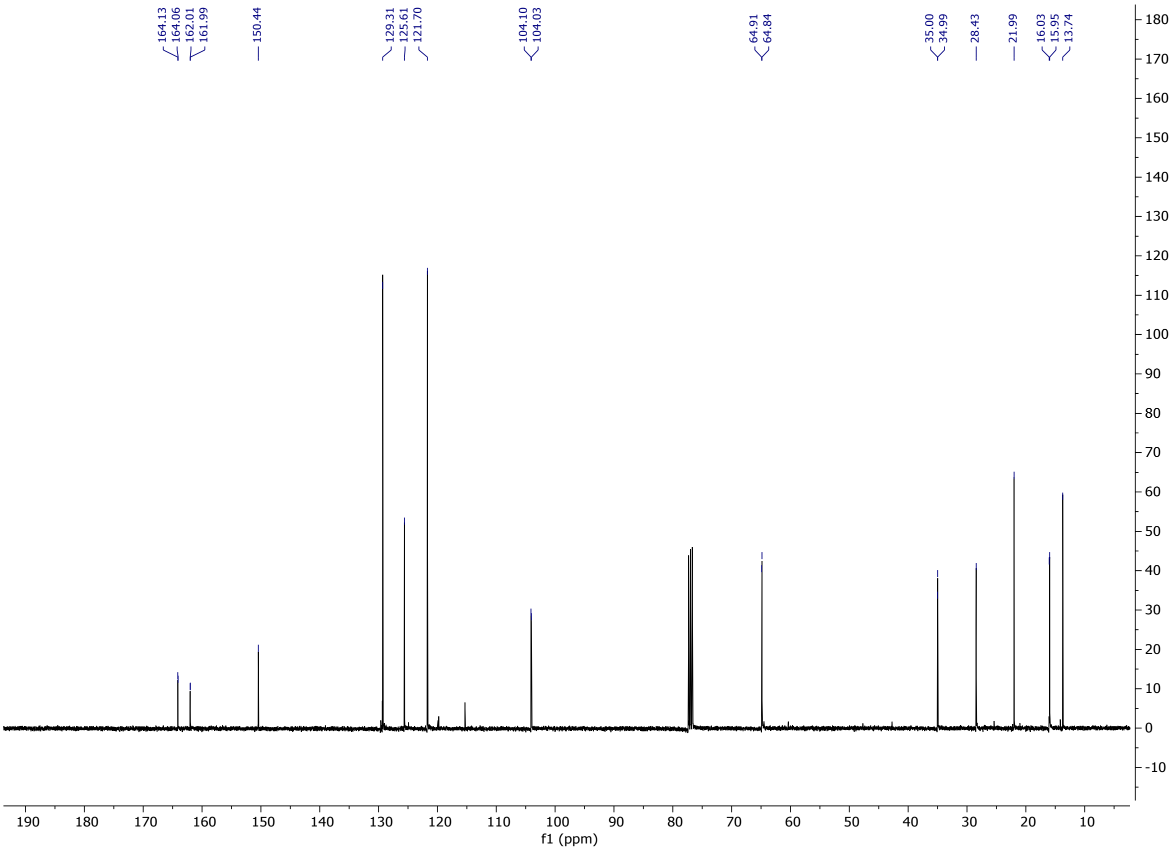

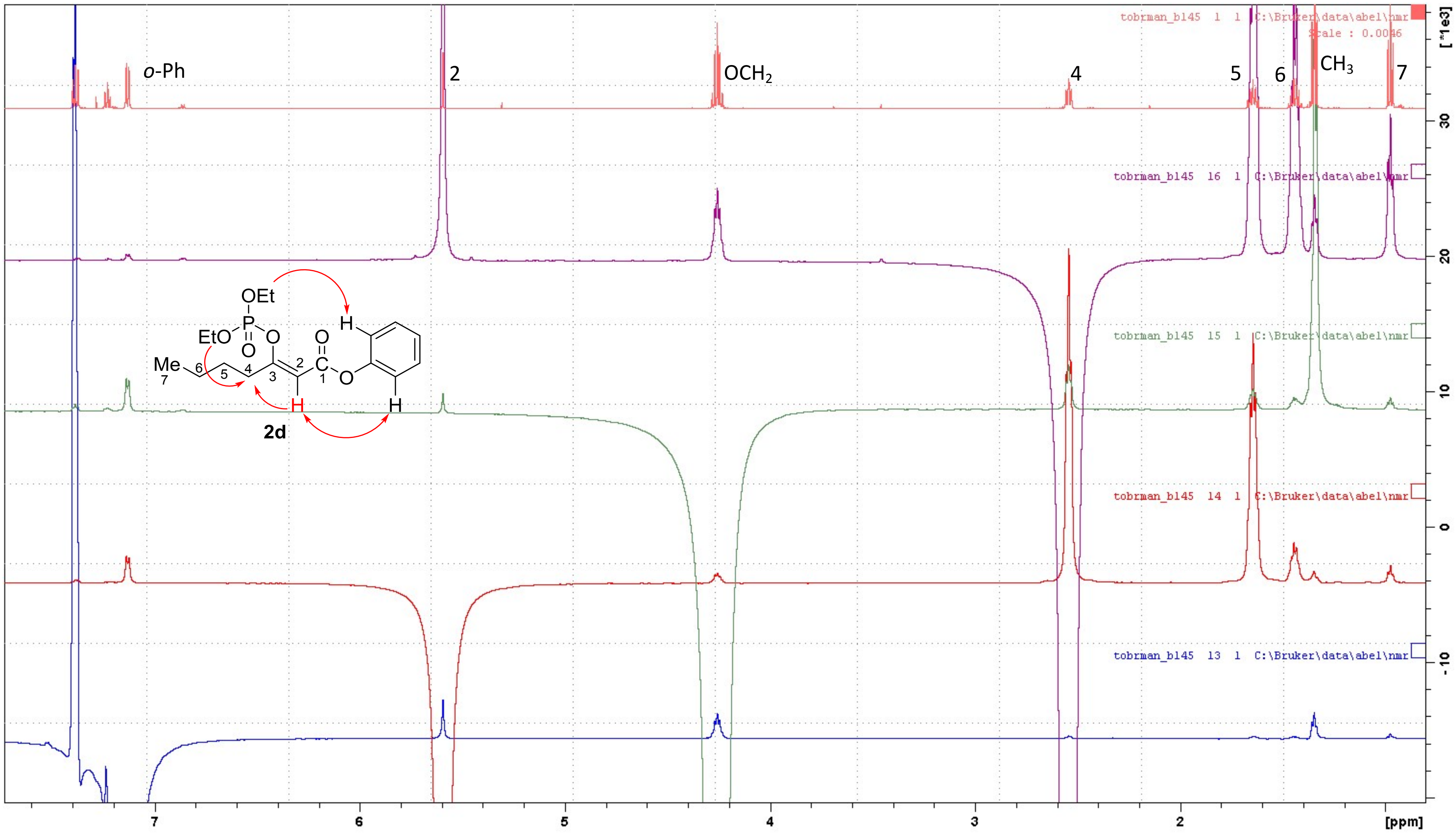

o-Ph
2
OCH2
4
5
6
CH3
7
tobrman_b145 1 1 C:\Bruker\data\abel\nmr
Scale : 0.0046
tobrman_b145 16 1 C:\Bruker\data\abel\nmr
tobrman_b145 15 1 C:\Bruker\data\abel\nmr
tobrman_b145 14 1 C:\Bruker\data\abel\nmr
tobrman_b145 13 1 C:\Bruker\data\abel\nmr
[*1e3]
[ppm]
OEt
EtO
Me
2d

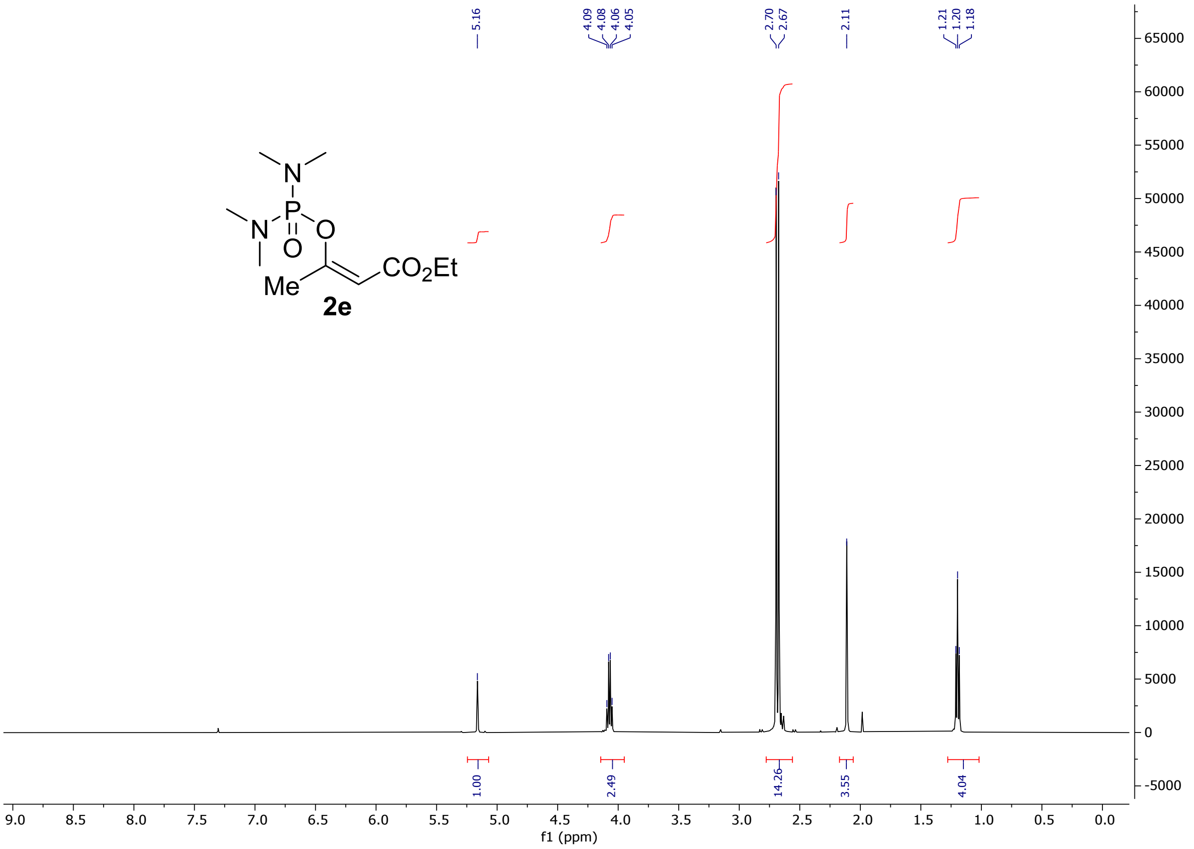
5.16
4.09
4.08
4.06
4.05
2.70
2.67
2.11
1.21
1.20
1.18
CO2Et
Me
2e
1.00
2.49
14.26
3.55
4.04
f1 (ppm)

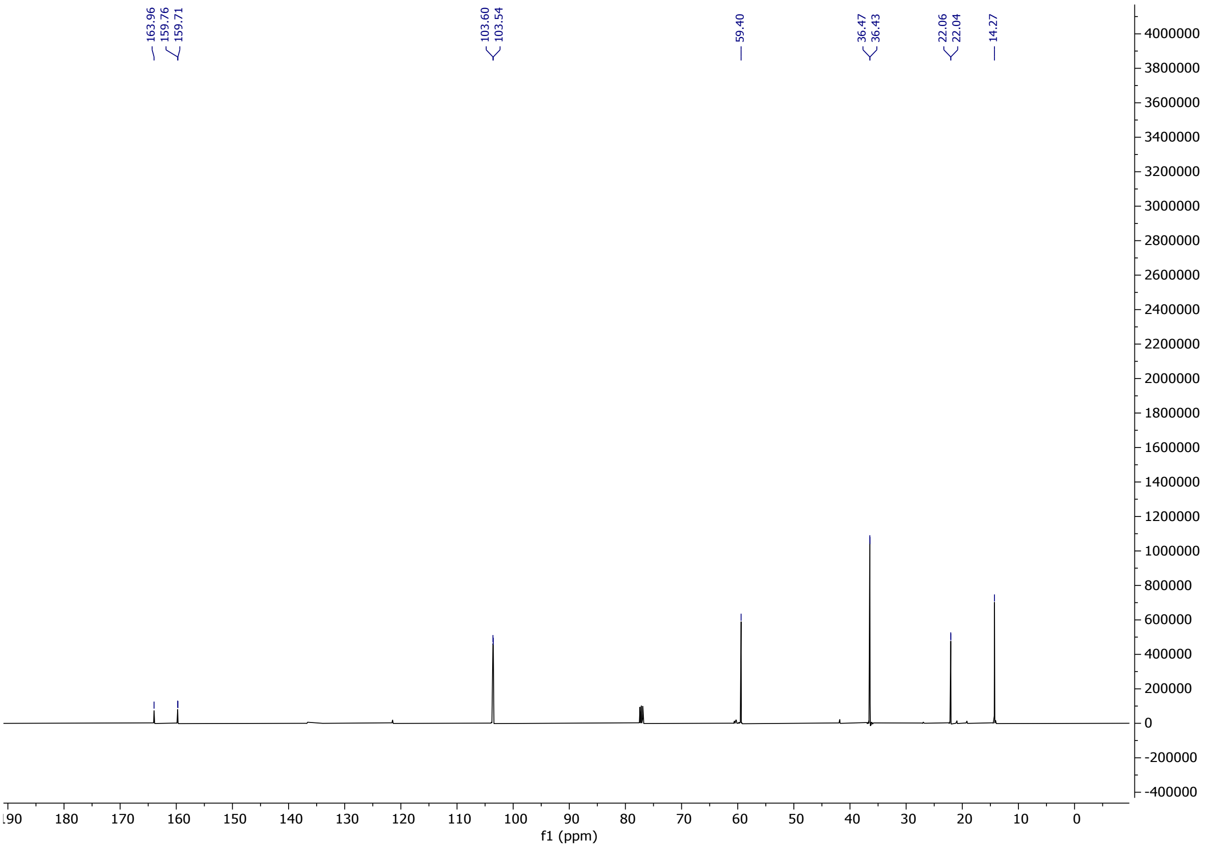
163.96
159.76
159.71
103.60
103.54
59.40
36.47
36.43
22.06
22.04
14.27
f1 (ppm)

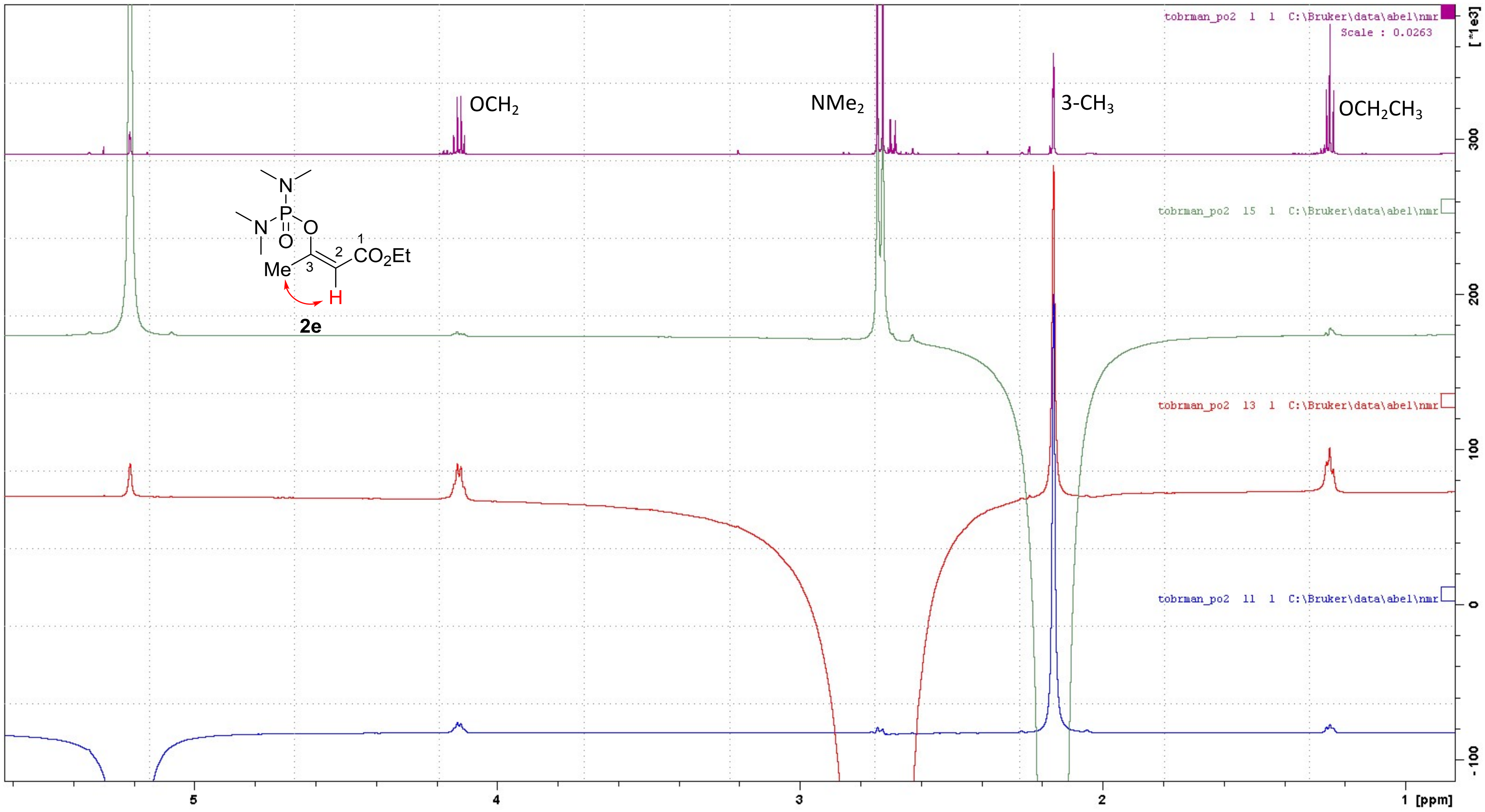

tobrman_po2 1 1 C:\Bruker\data\abel\nmr
Scale : 0.0263
OCH2
NMe2
3-CH3
OCH2CH3
tobrman_po2 15 1 C:\Bruker\data\abel\nmr
tobrman_po2 13 1 C:\Bruker\data\abel\nmr
tobrman_po2 11 1 C:\Bruker\data\abel\nmr
2e
[*1e3]
300
200
100
0
-100
5
4
3
2
1 [ppm]

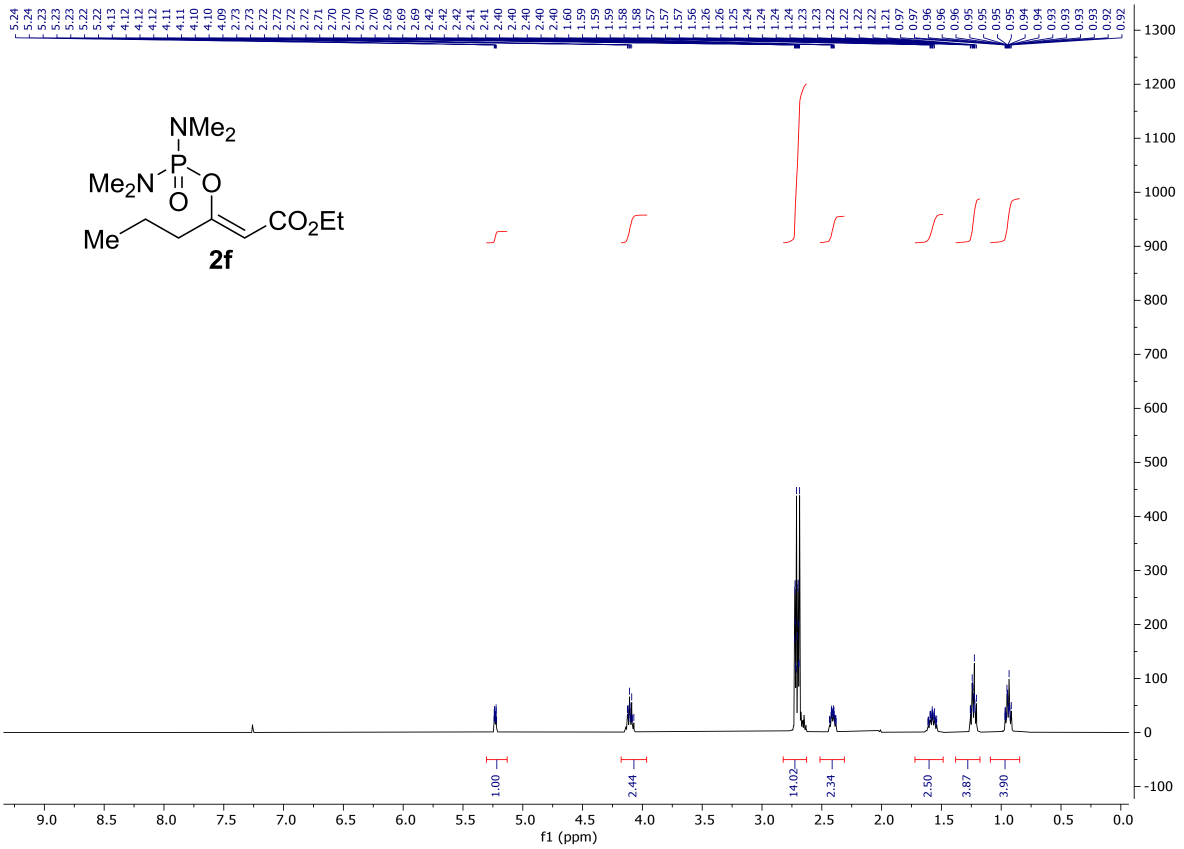

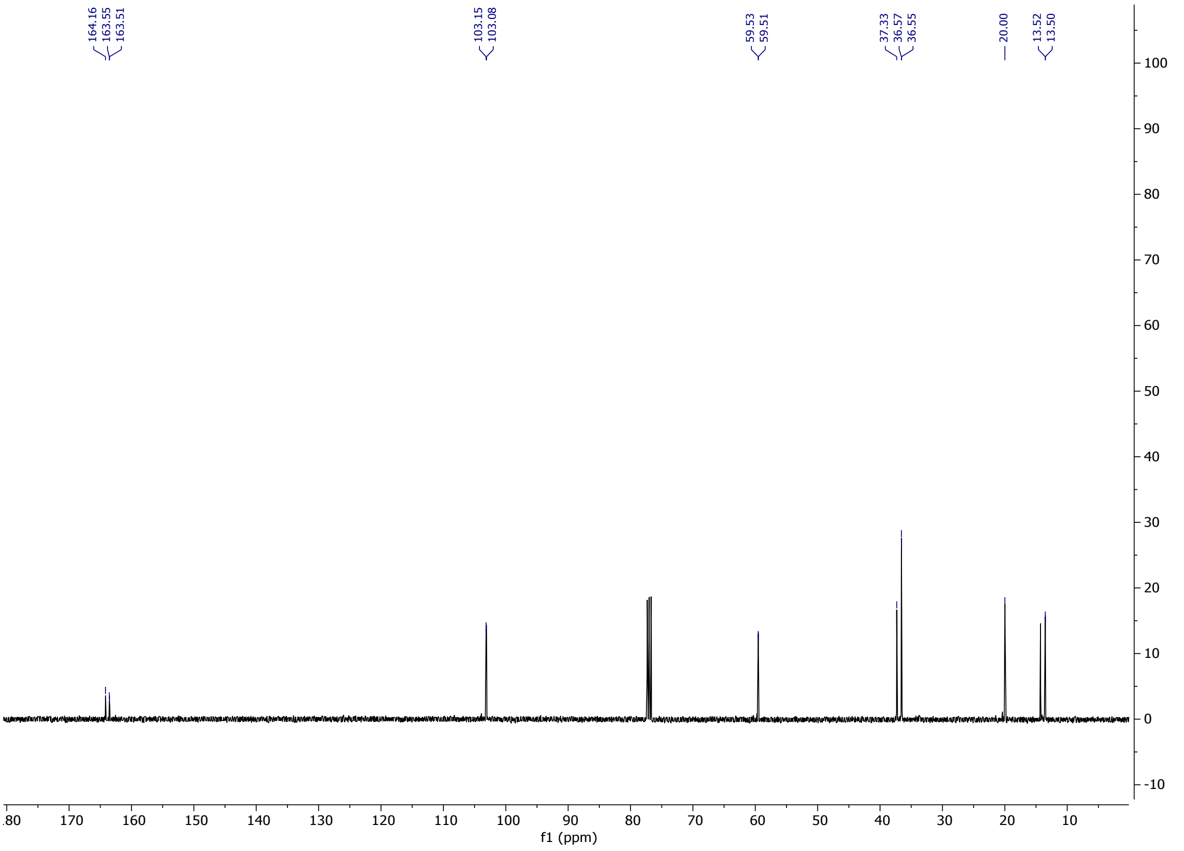

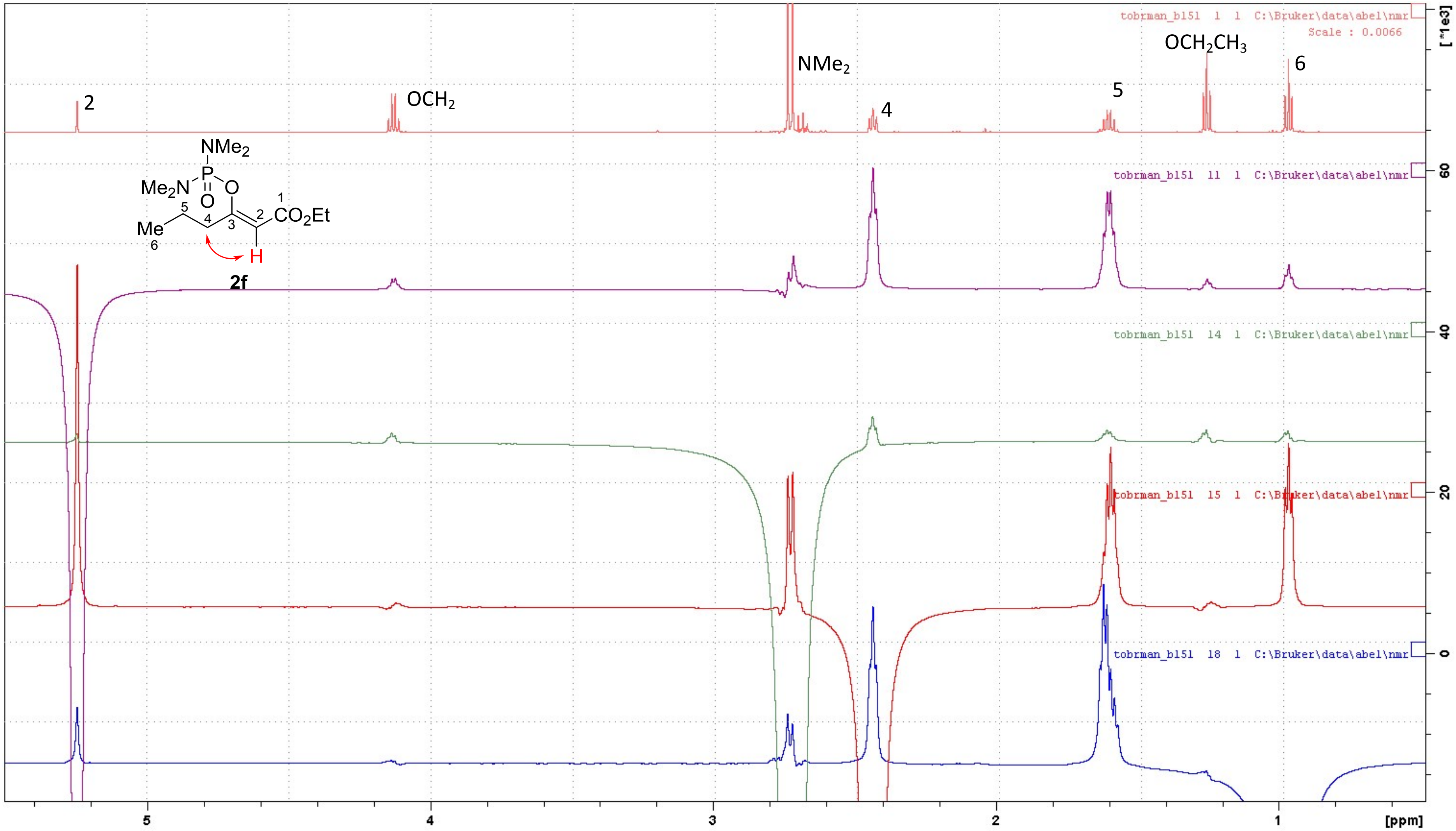

tobrman_b151 1 1 C:\Bruker\data\abel\nmr
Scale : 0.0066
tobrman_b151 11 1 C:\Bruker\data\abel\nmr
tobrman_b151 14 1 C:\Bruker\data\abel\nmr
tobrman_b151 15 1 C:\Bruker\data\abel\nmr
tobrman_b151 18 1 C:\Bruker\data\abel\nmr
[*1e3]
2
OCH2
NMe2
4
5
OCH2CH3
6
NMe2
Me2N
P
O
O
1
CO2Et
2
3
4
5
Me
6
H
2f
[ppm]

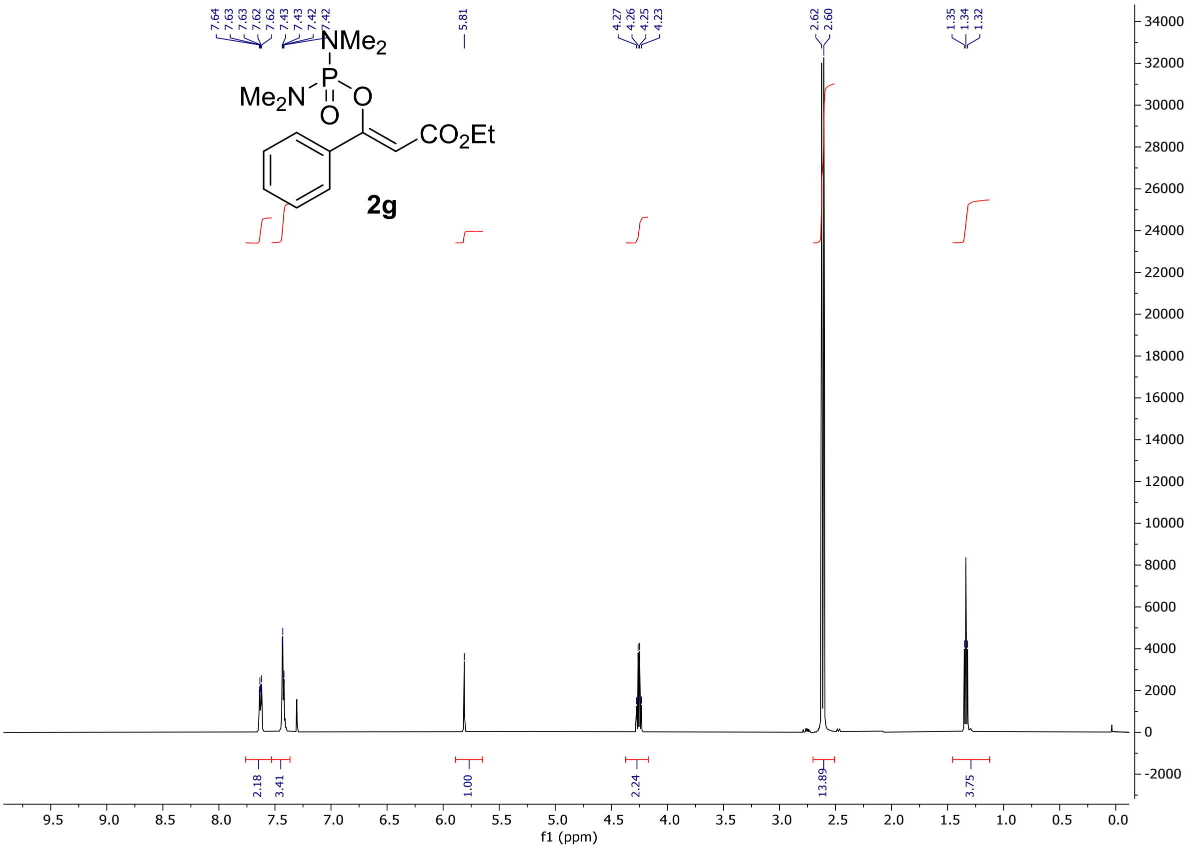

7.64
7.63
7.63
7.62
7.62
7.43
7.43
7.42
7.42
5.81
4.27
4.26
4.25
4.23
2.62
2.60
1.35
1.34
1.32
NMe2
Me2N
P
O
O
CO2Et
2g
2.18
3.41
1.00
2.24
13.89
3.75
f1 (ppm)

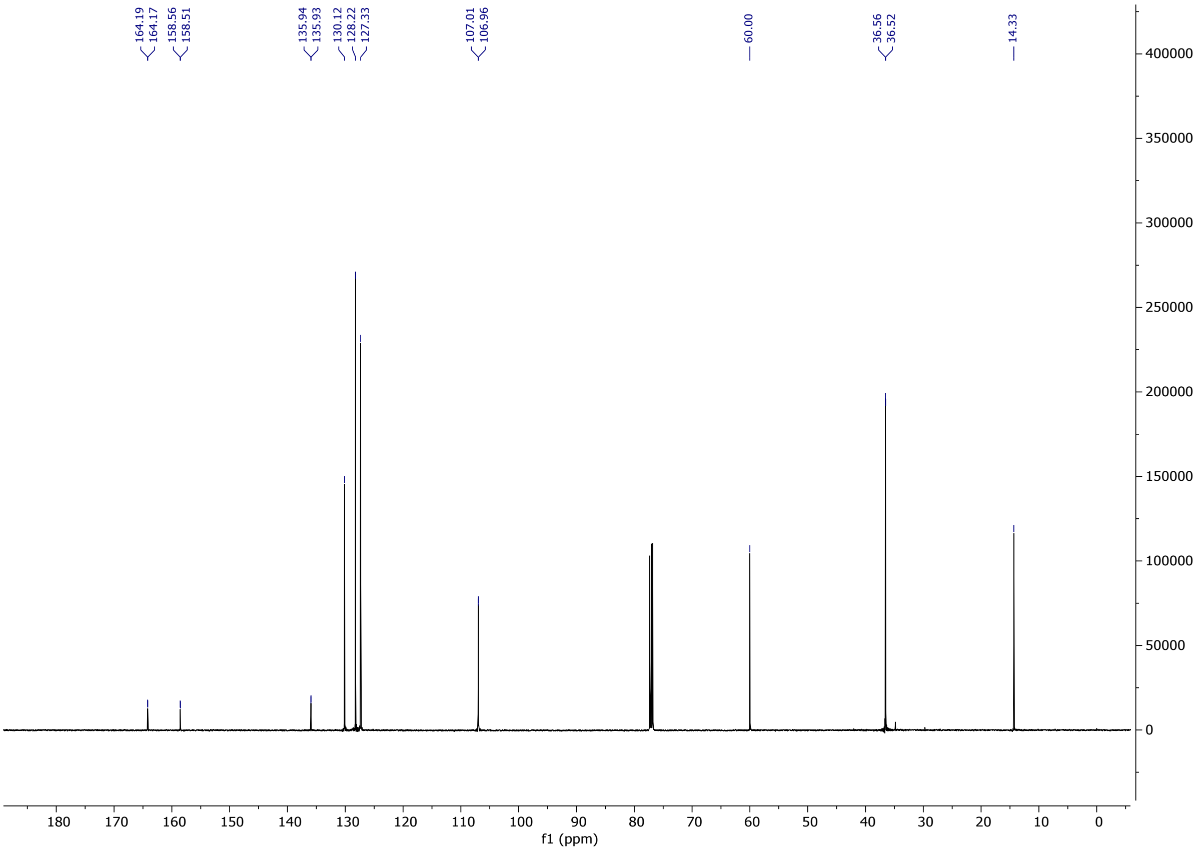

164.19
164.17
158.56
158.51
135.94
135.93
130.12
128.22
127.33
107.01
106.96
60.00
36.56
36.52
14.33
f1 (ppm)

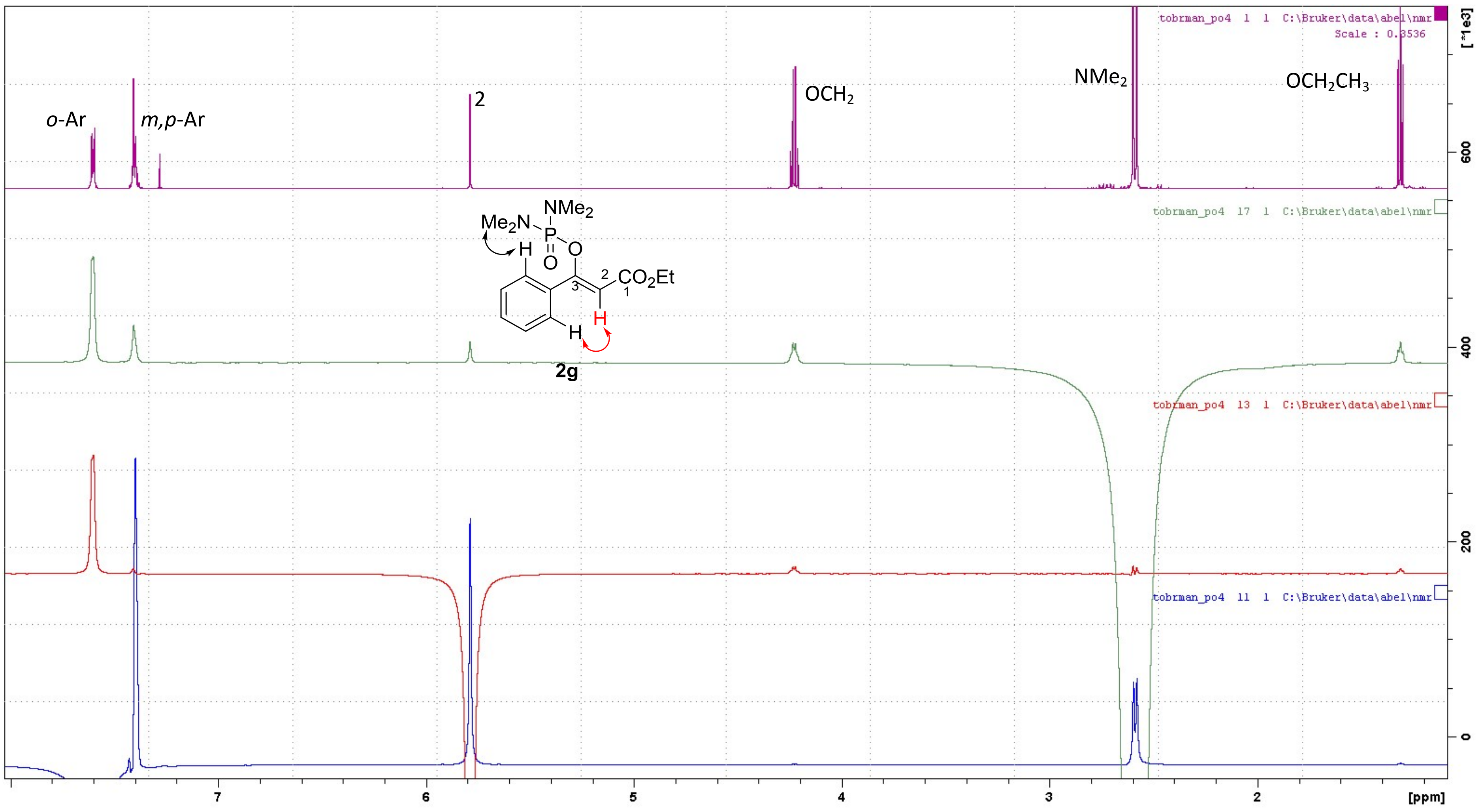

o-Ar
m,p-Ar
2
OCH2
NMe2
OCH2CH3
tobrman_po4 1 1 C:\Bruker\data\abel\nmr
Scale : 0.3536
tobrman_po4 17 1 C:\Bruker\data\abel\nmr
tobrman_po4 13 1 C:\Bruker\data\abel\nmr
tobrman_po4 11 1 C:\Bruker\data\abel\nmr
[*1e3]
600
400
200
0
7
6
5
4
3
2
[ppm]
Me2N
NMe2
P
O
O
H
H
H
CO2Et
1
2
3
2g

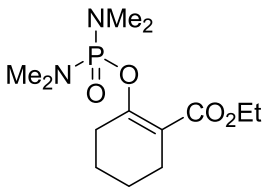

2h

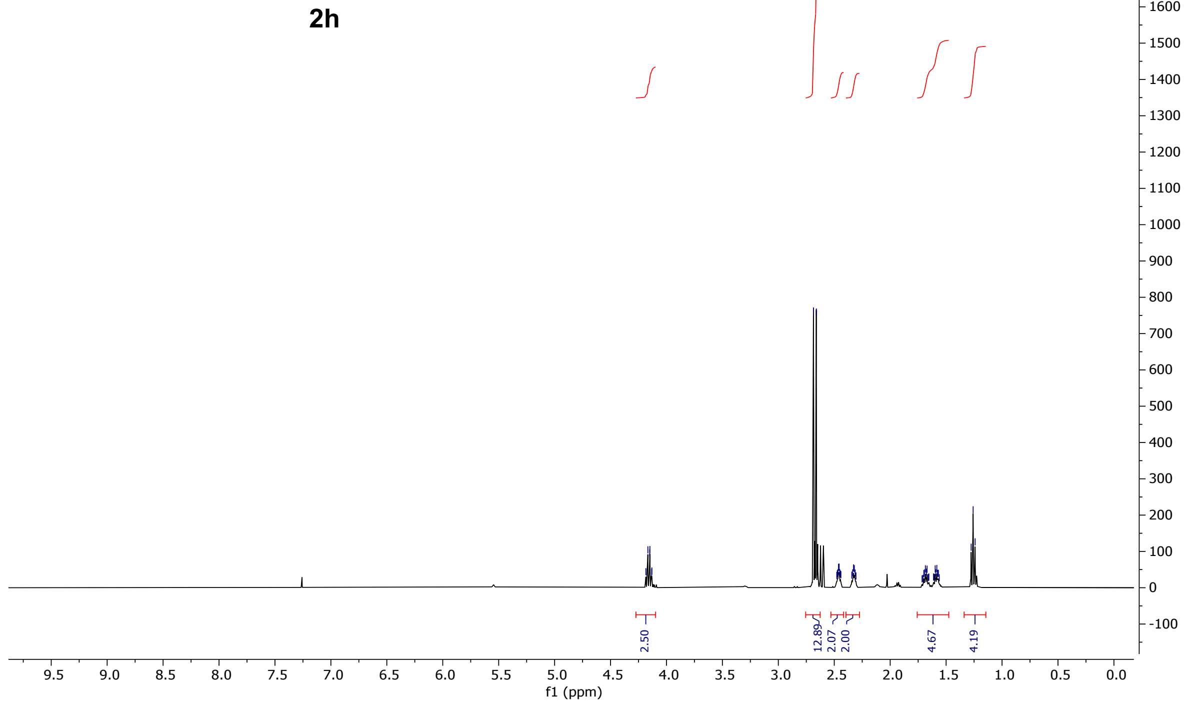

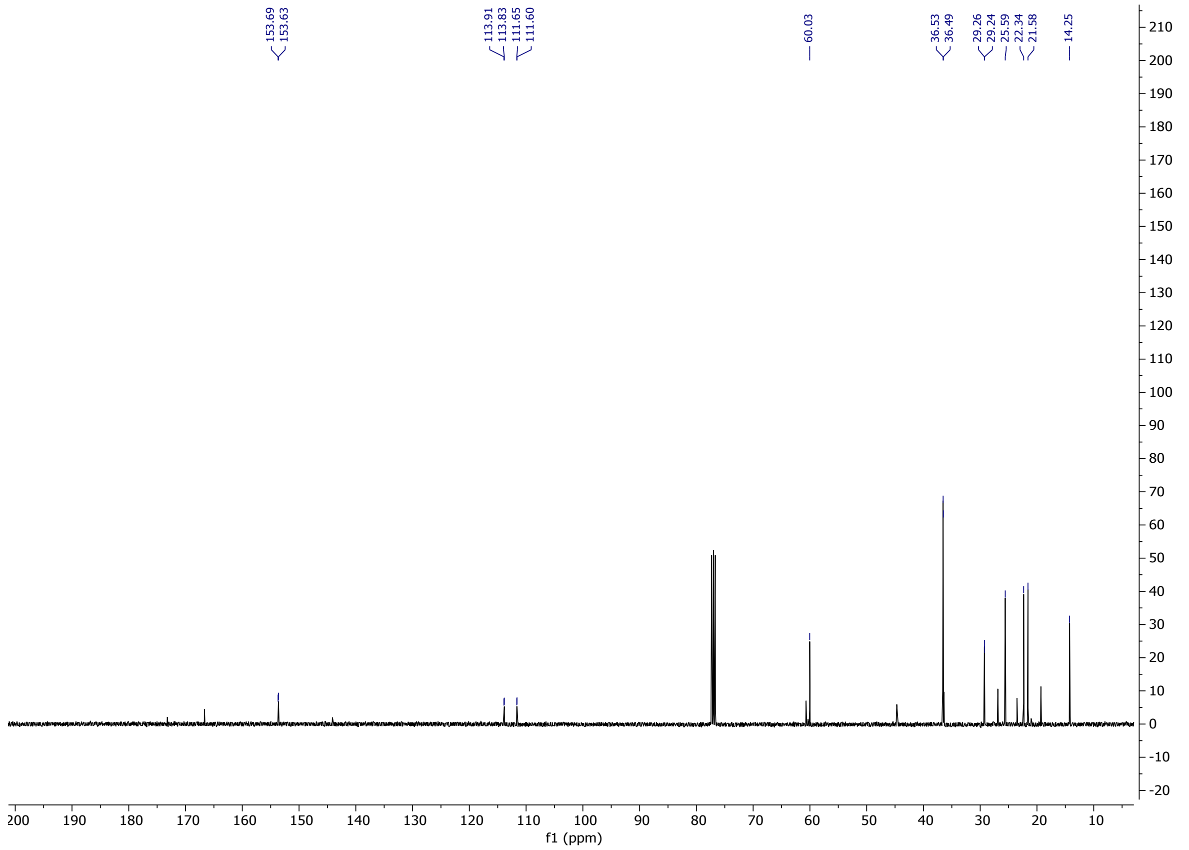

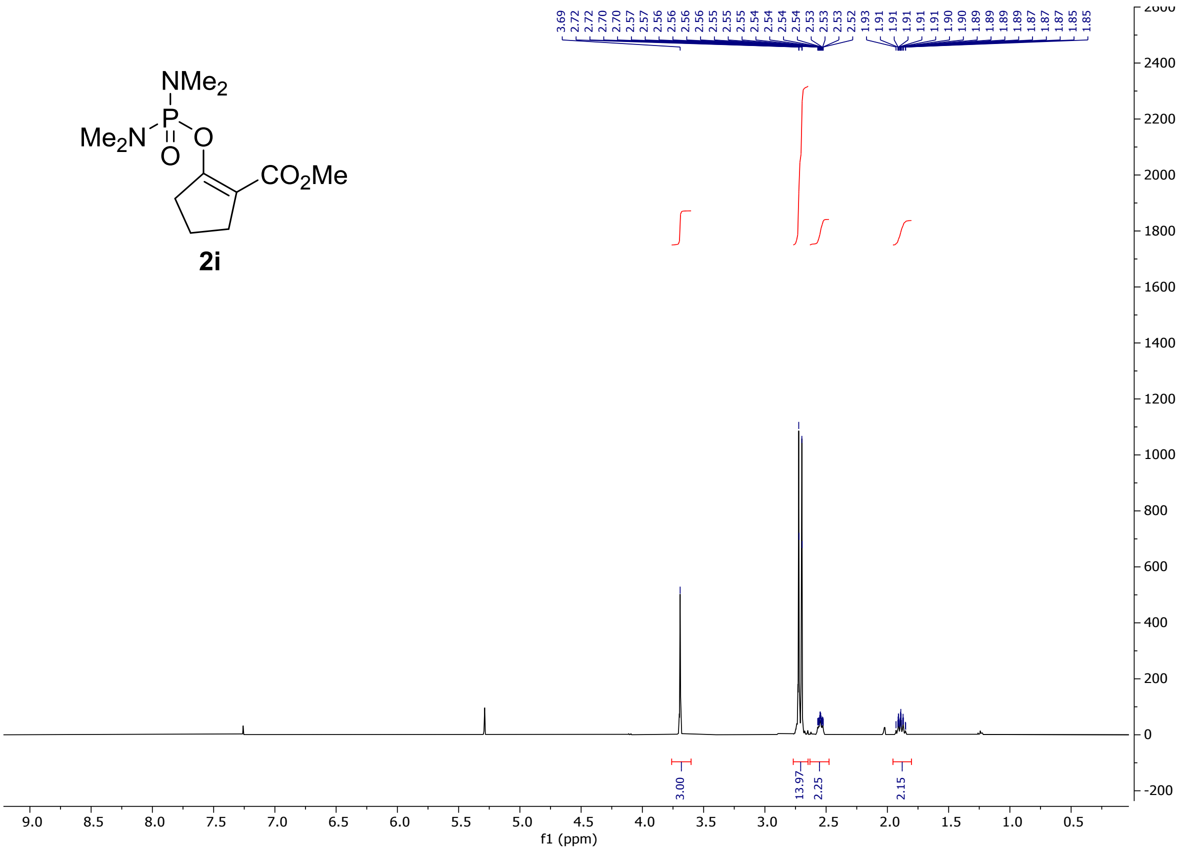
NMe2
Me2N
P
O
O
CO2Me
2i
3.69
2.72
2.72
2.70
2.70
2.57
2.57
2.56
2.56
2.56
2.55
2.55
2.55
2.54
2.54
2.54
2.53
2.53
2.53
2.52
1.93
1.91
1.91
1.91
1.91
1.90
1.90
1.89
1.89
1.89
1.87
1.87
1.85
1.85
3.00
13.97
2.25
2.15
f1 (ppm)

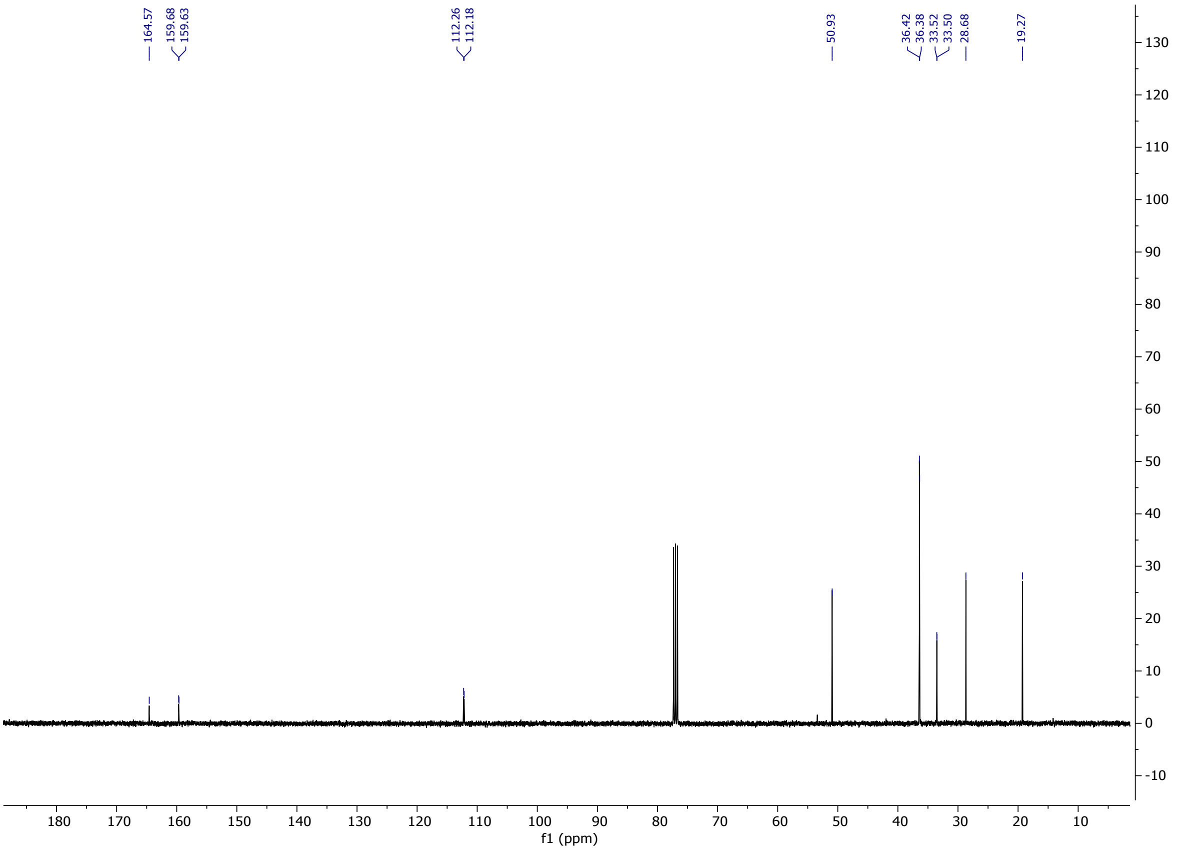
164.57
159.68
159.63
112.26
112.18
50.93
36.42
36.38
33.52
33.50
28.68
19.27
f1 (ppm)

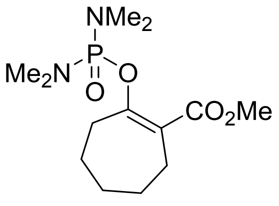

2j

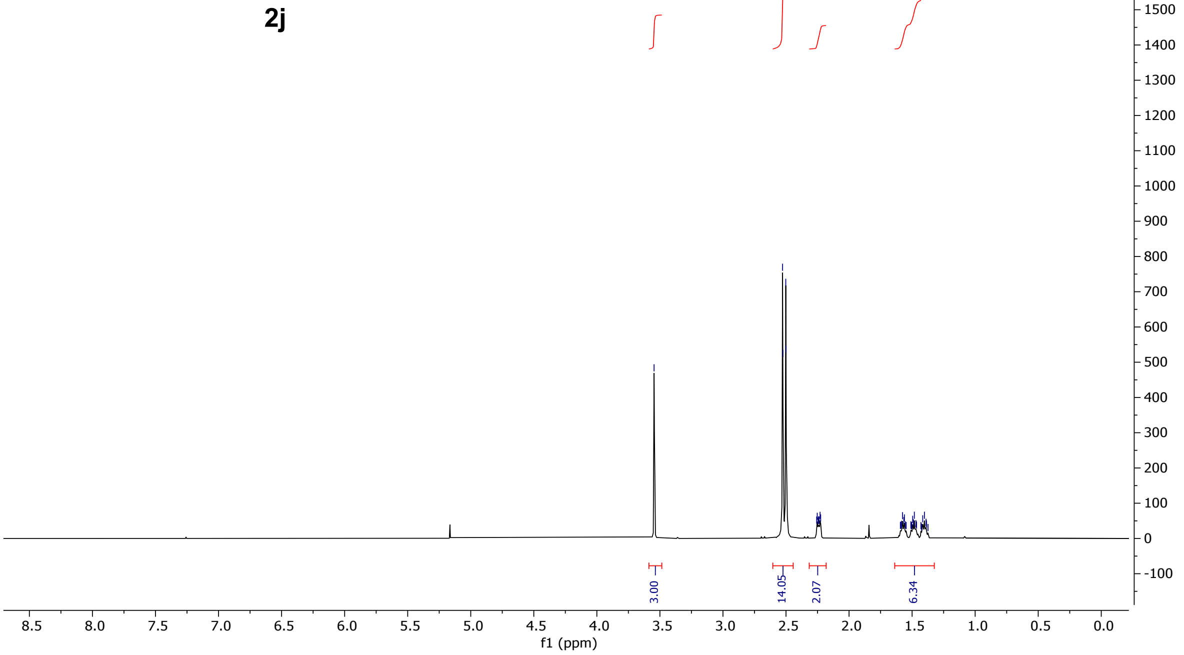
3.55
2.53
2.52
2.50
2.50
2.26
2.25
2.25
2.24
2.24
2.24
2.23
2.23
1.59
1.59
1.58
1.58
1.57
1.56
1.55
1.51
1.51
1.50
1.50
1.49
1.48
1.47
1.46
1.42
1.40
1.39
1.39
3.00
14.05
2.07
6.34
f1 (ppm)

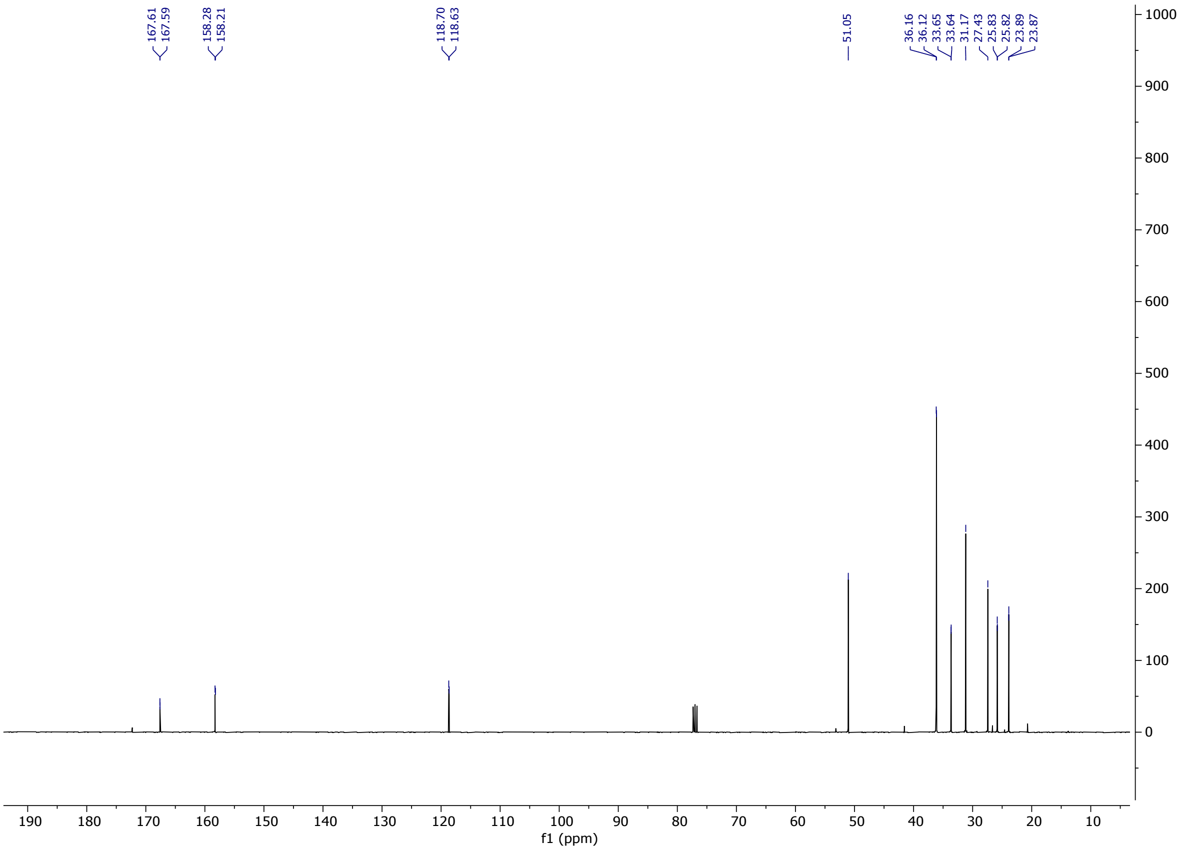
167.61
167.59
158.28
158.21
118.70
118.63
51.05
36.16
36.12
33.65
33.64
31.17
27.43
25.83
25.82
23.89
23.87
f1 (ppm)

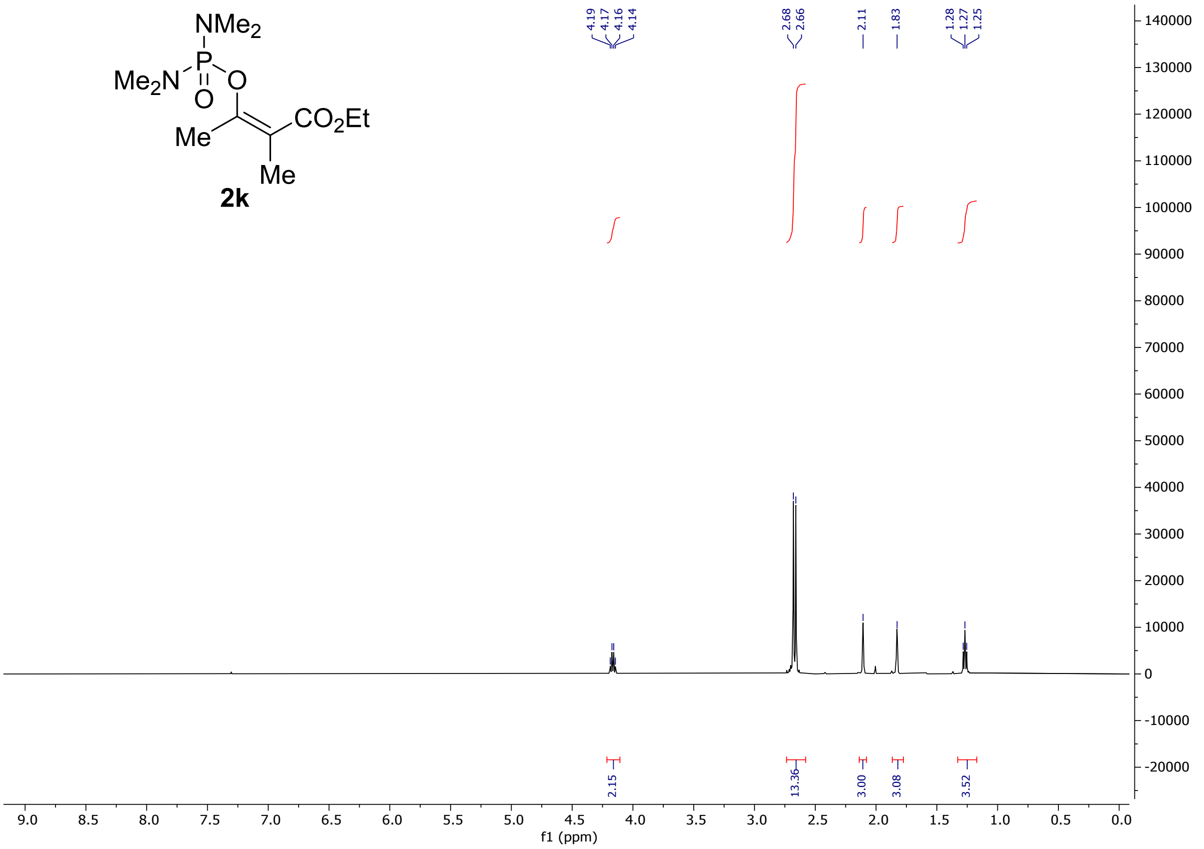
NMe2
Me2N
P
O
O
CO2Et
Me
Me
2k
4.19
4.17
4.16
4.14
2.68
2.66
2.11
1.83
1.28
1.27
1.25
2.15
13.36
3.00
3.08
3.52
f1 (ppm)

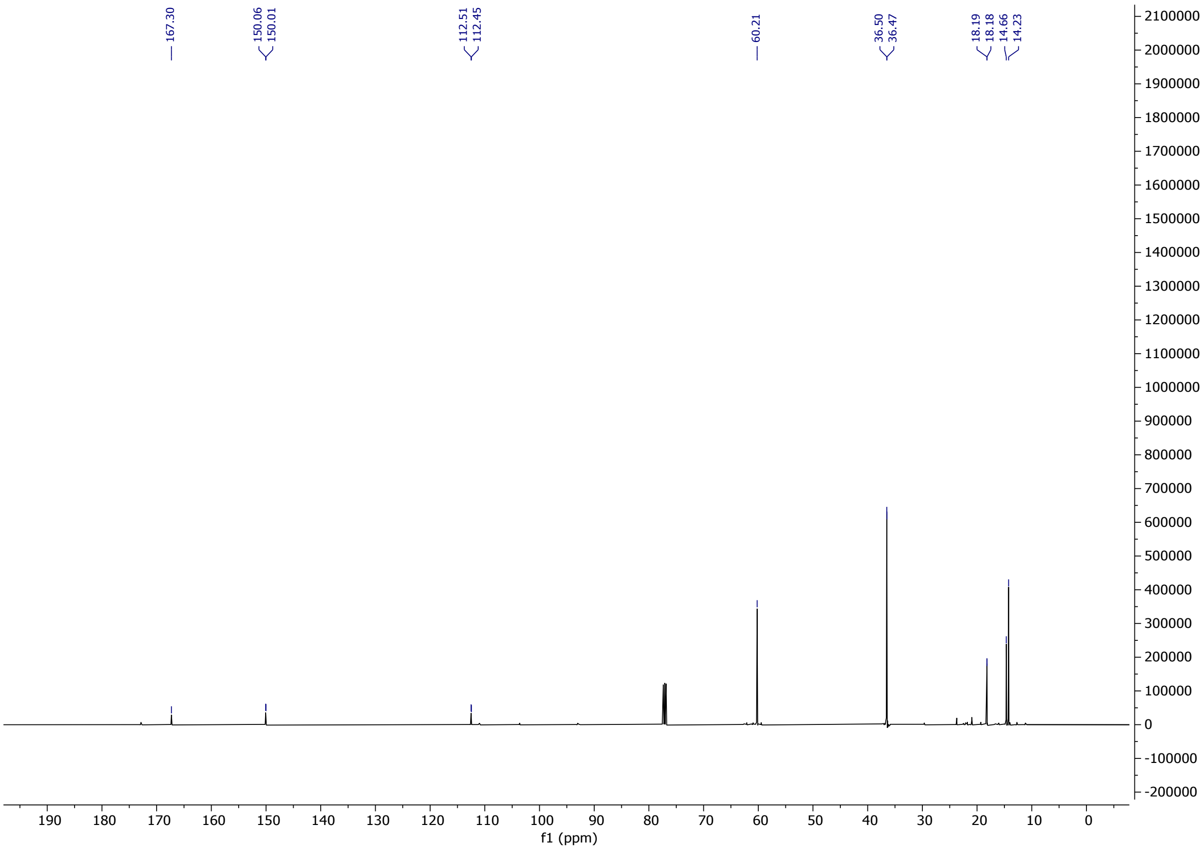
167.30
150.06
150.01
112.51
112.45
60.21
36.50
36.47
18.19
18.18
14.66
14.23
f1 (ppm)

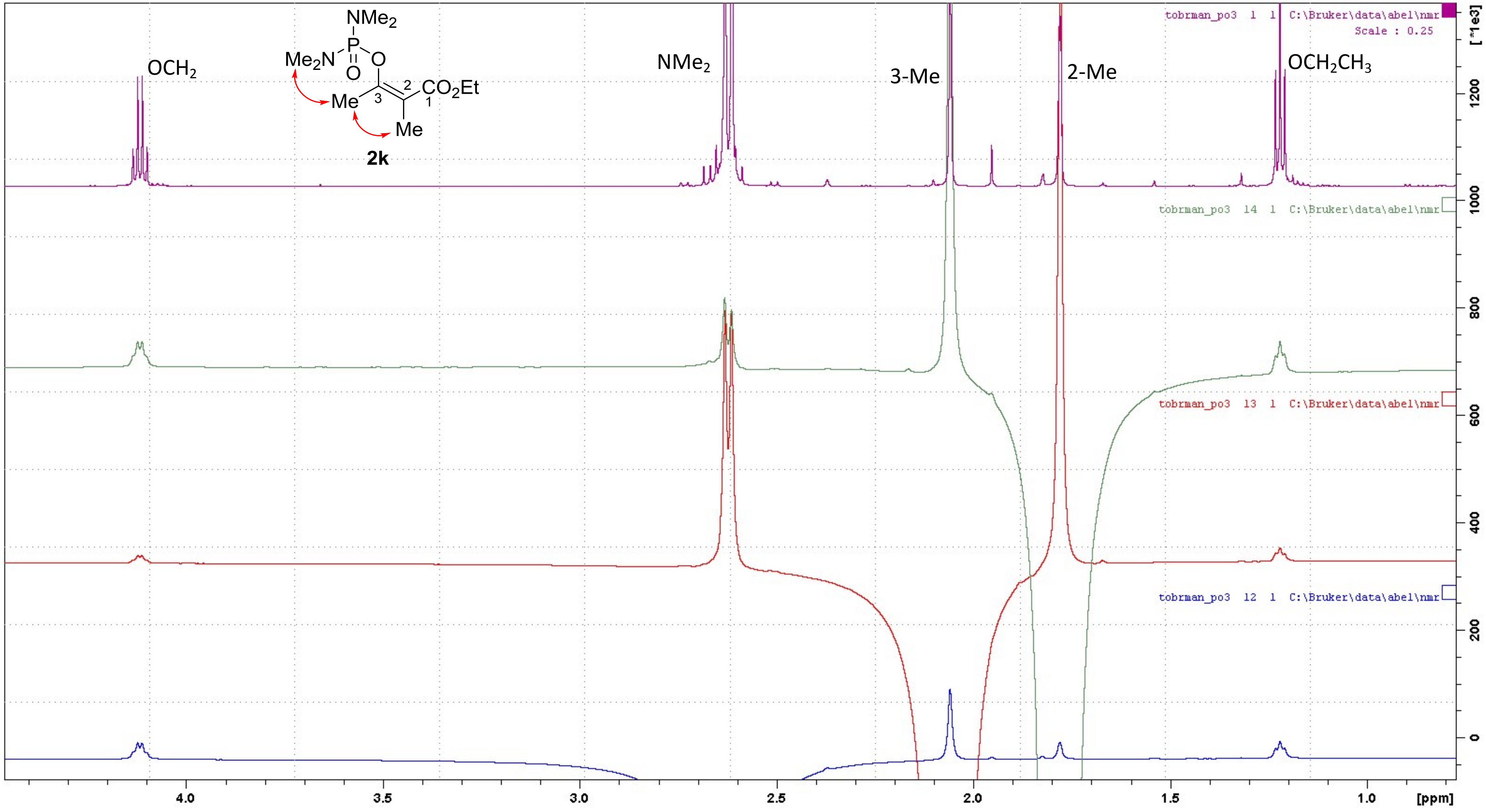

OCH2
NMe2
Me2N
P
O
O
Me
3
2
CO2Et
1
Me
2k
NMe2
3-Me
2-Me
OCH2CH3
tobrman_po3 1 1 C:\Bruker\data\abel\nmr
Scale : 0.25
tobrman_po3 14 1 C:\Bruker\data\abel\nmr
tobrman_po3 13 1 C:\Bruker\data\abel\nmr
tobrman_po3 12 1 C:\Bruker\data\abel\nmr
[*1e3]
1200
1000
800
600
400
200
0
4.0
3.5
3.0
2.5
2.0
1.5
1.0
[ppm]

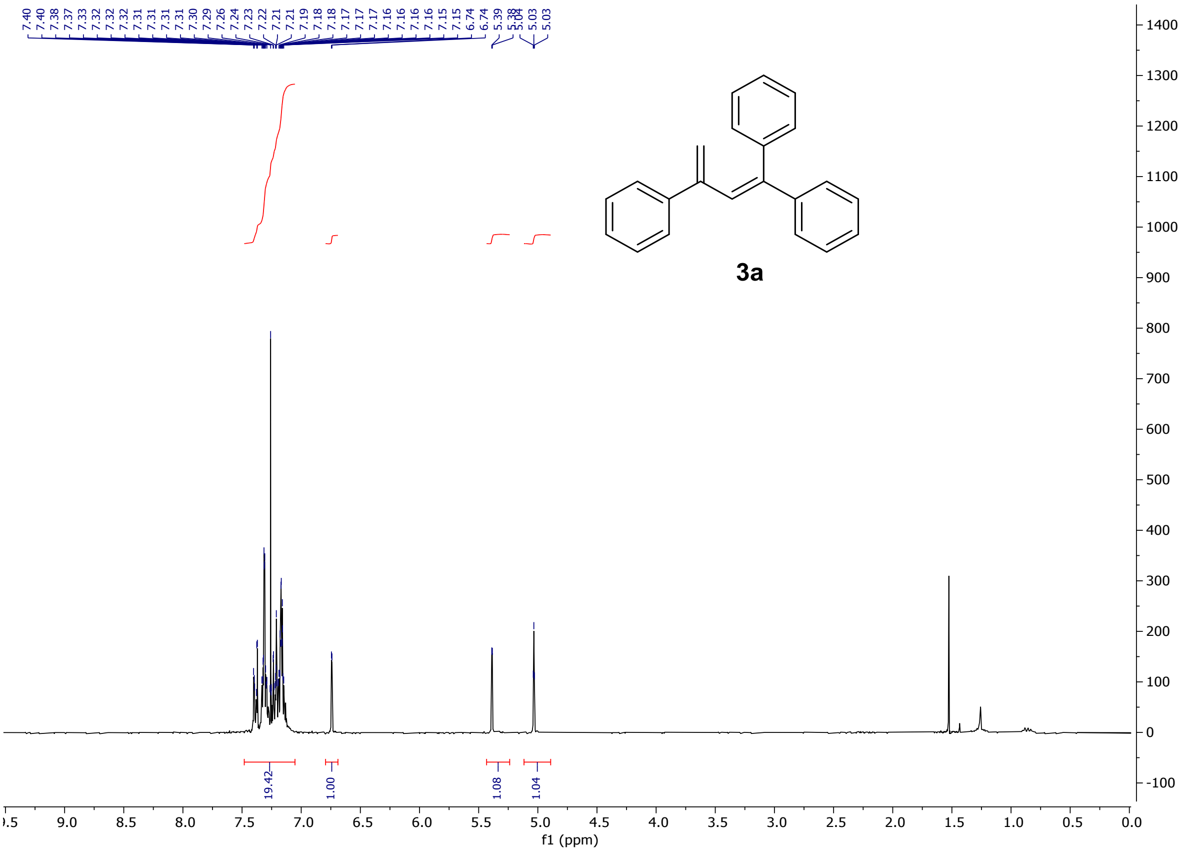
3a
f1 (ppm)

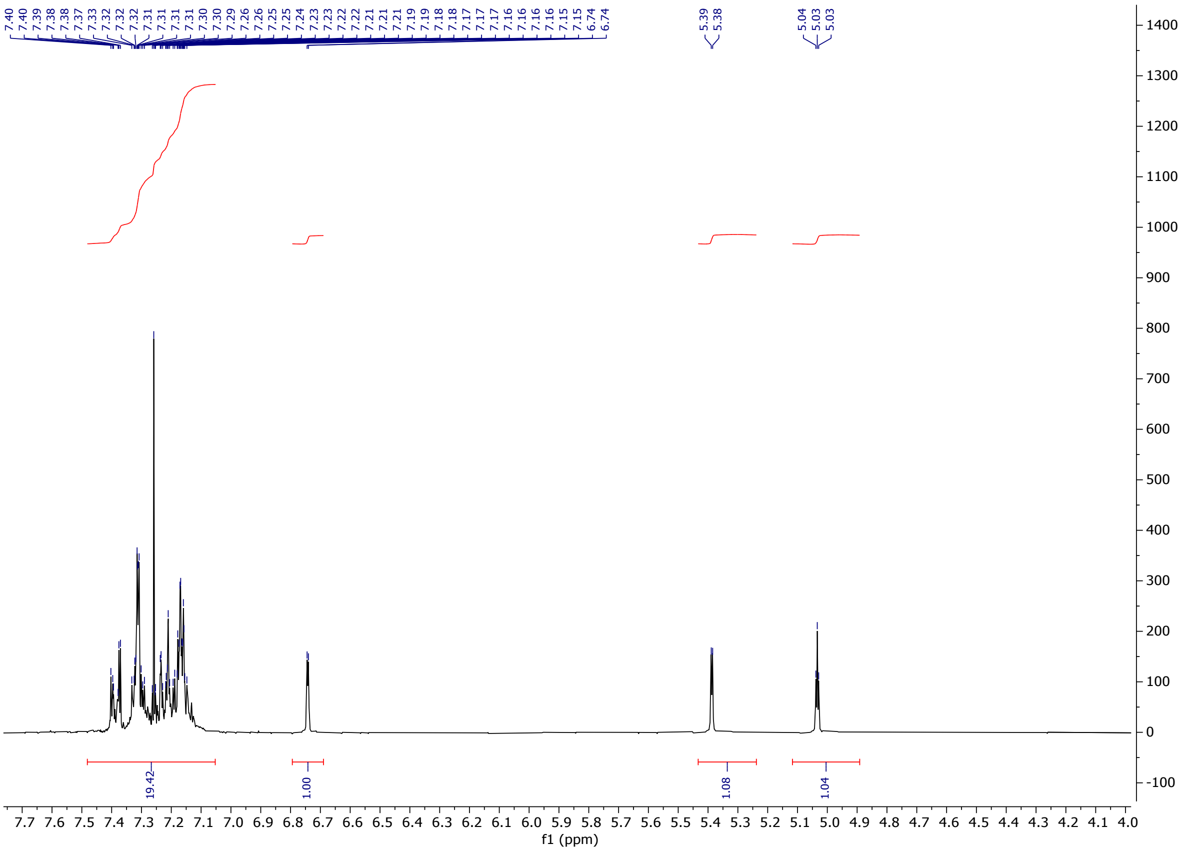
f1 (ppm)

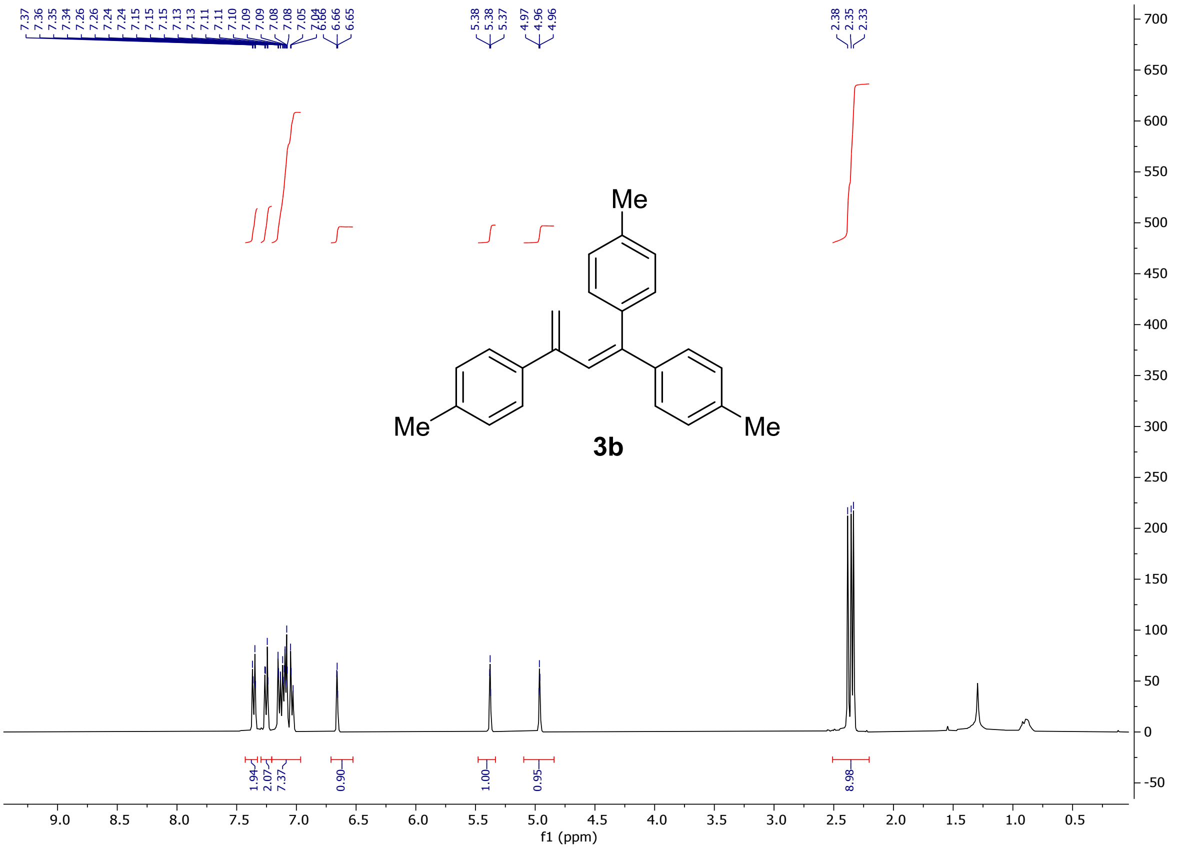
7.37 7.36 7.35 7.34 7.26 7.26 7.24 7.24 7.15 7.15 7.15 7.13 7.13 7.11 7.11 7.10 7.09 7.09 7.08 7.08 7.05 7.04 6.66 6.66 6.65
5.38 5.38 5.37 4.97 4.96 4.96
2.38 2.35 2.33
Me
Me
Me
3b
1.94 2.07 7.37 0.90 1.00 0.95 8.98
f1 (ppm)

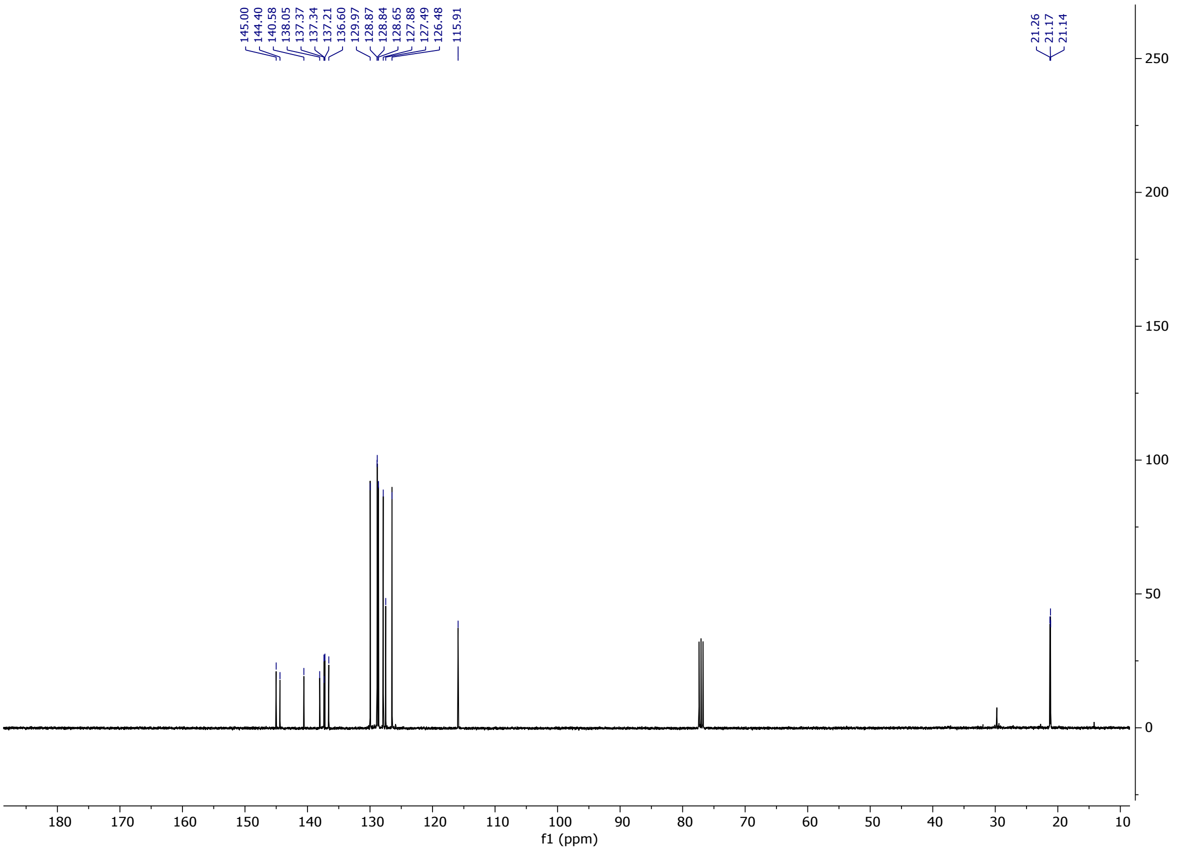
145.00 144.40 140.58 138.05 137.37 137.34 137.21 136.60 129.97 128.87 128.84 128.65 127.88 127.49 126.48 115.91
21.26 21.17 21.14
f1 (ppm)

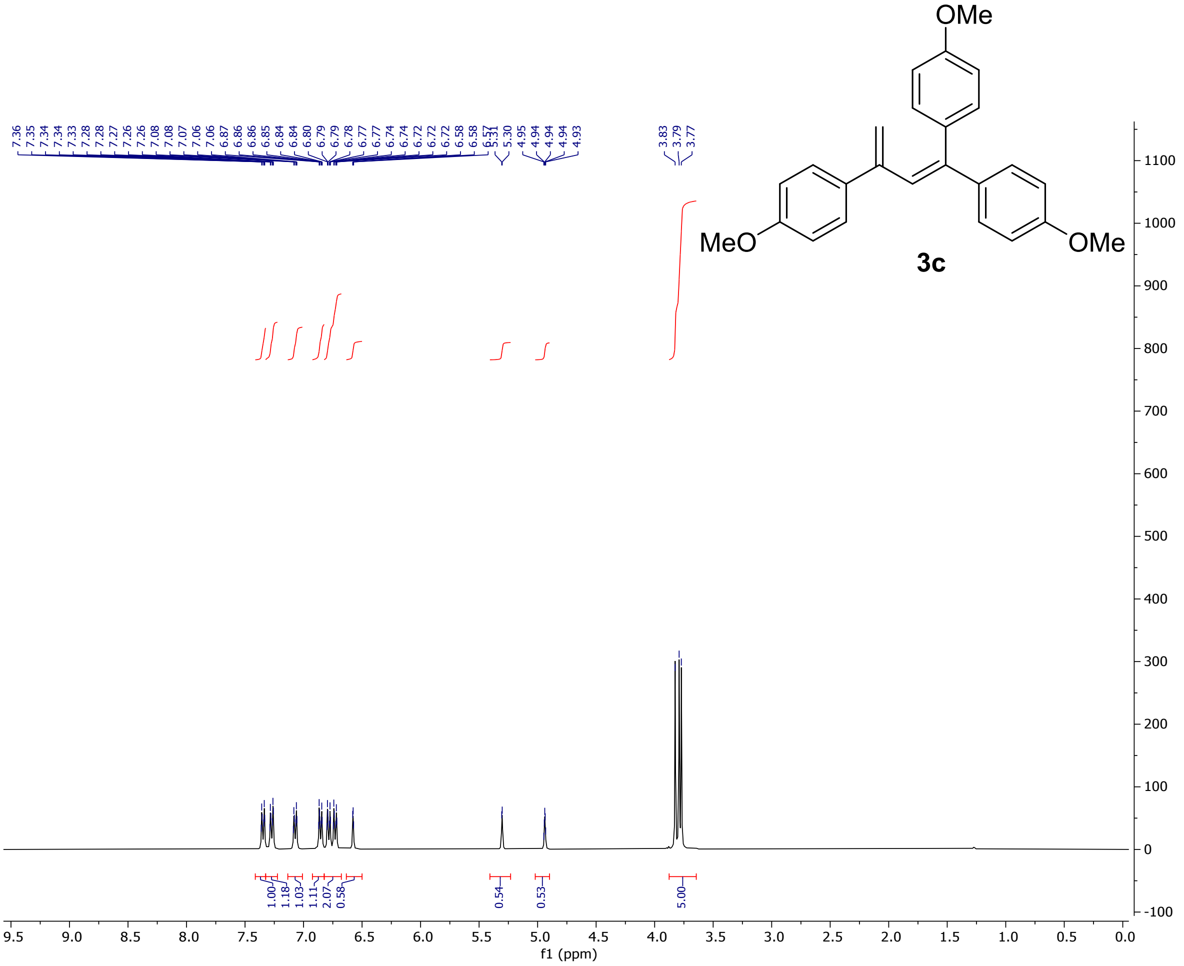

OMe
MeO
OMe
3c
7.36 7.35 7.34 7.34 7.33 7.28 7.28 7.27 7.26 7.26 7.08 7.08 7.07 7.06 7.06 6.87 6.86 6.86 6.85 6.84 6.84 6.80 6.79 6.79 6.78 6.77 6.77 6.74 6.74 6.72 6.72 6.72 6.58 6.57 5.31 5.30 4.95 4.94 4.94 4.94 4.93
3.83 3.79 3.77
1.00 1.18 1.03 1.11 2.07 0.58 0.54 0.53 5.00
f1 (ppm)

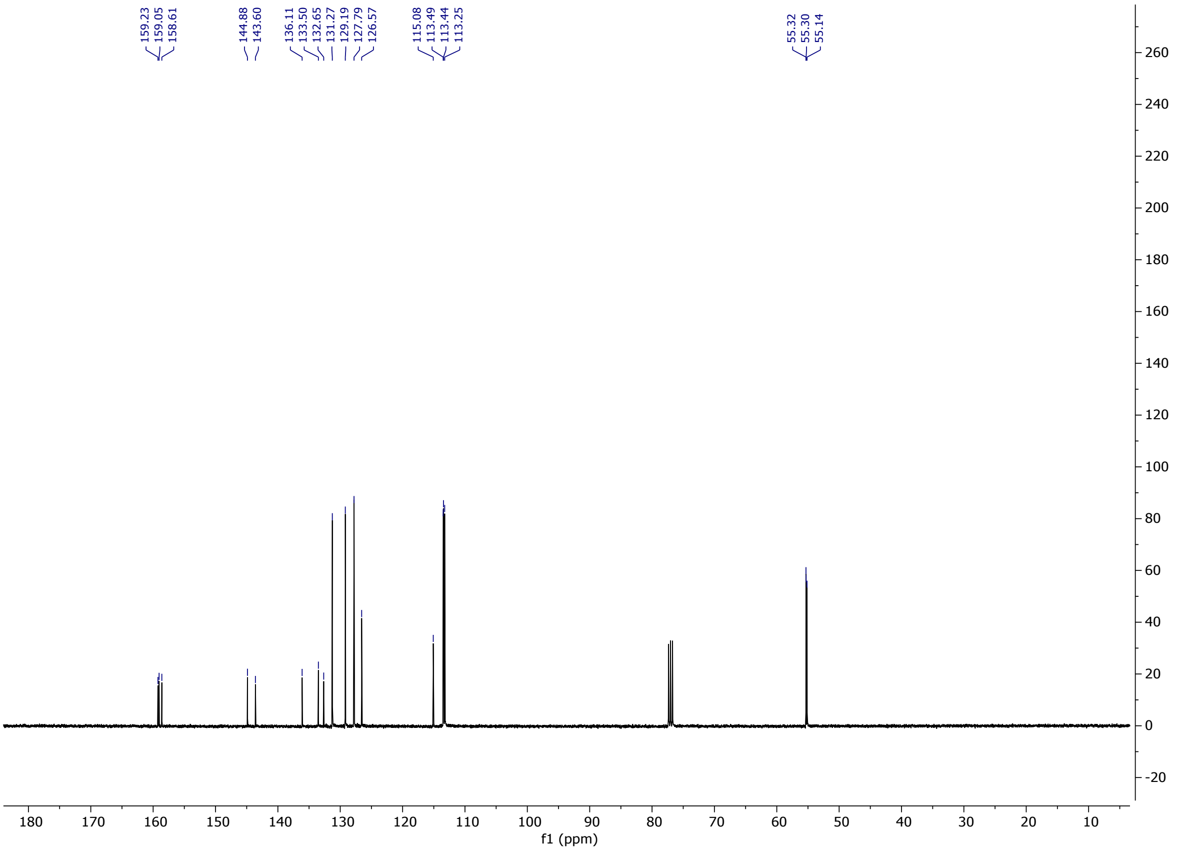

159.23 159.05 158.61
144.88 143.60
136.11 133.50 132.65 131.27 129.19 127.79 126.57
115.08 113.49 113.44 113.25
55.32 55.30 55.14
f1 (ppm)

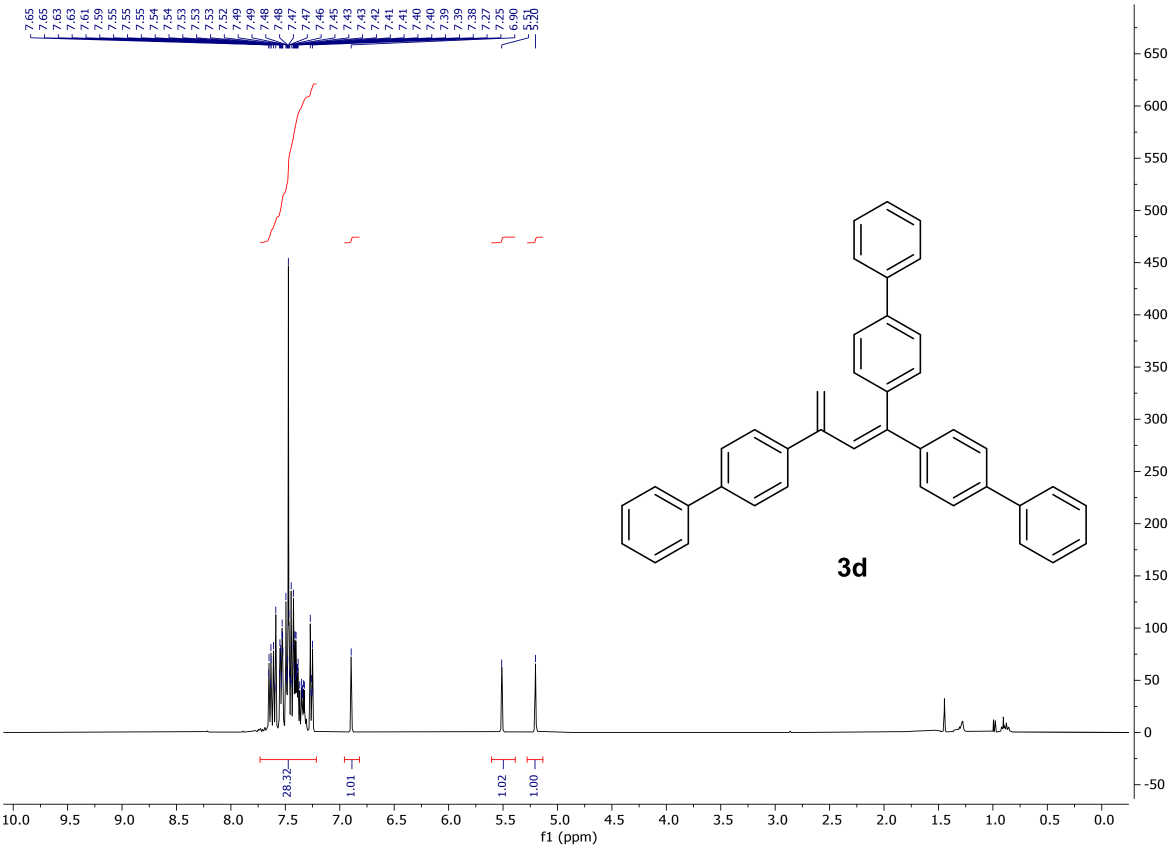
3d
f1 (ppm)

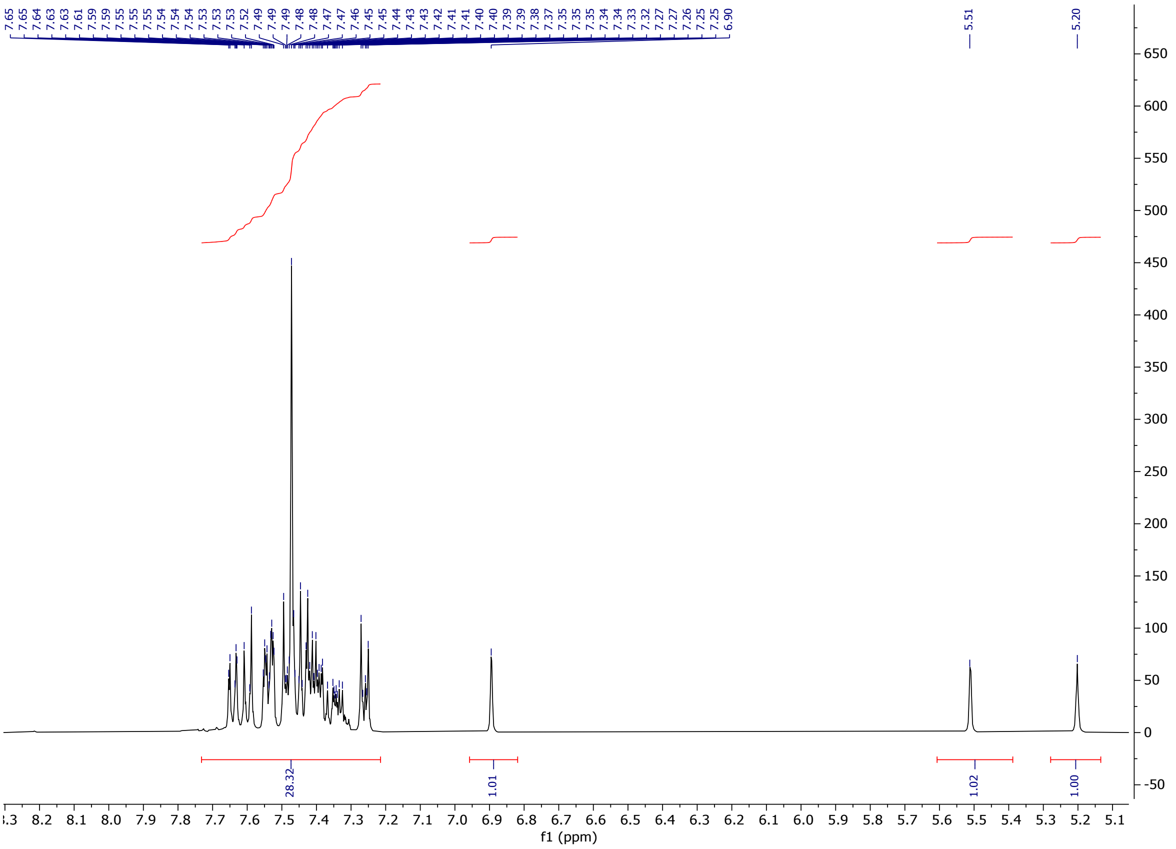
f1 (ppm)

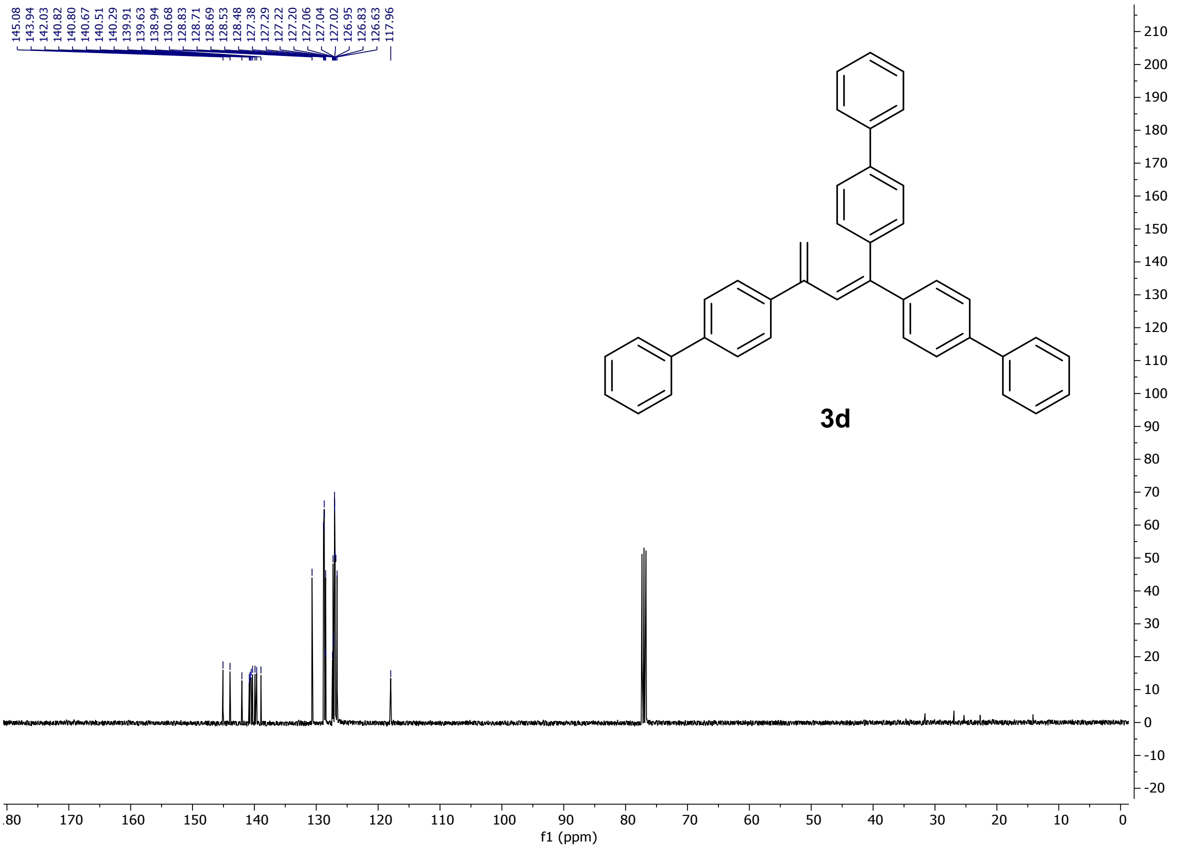

3d

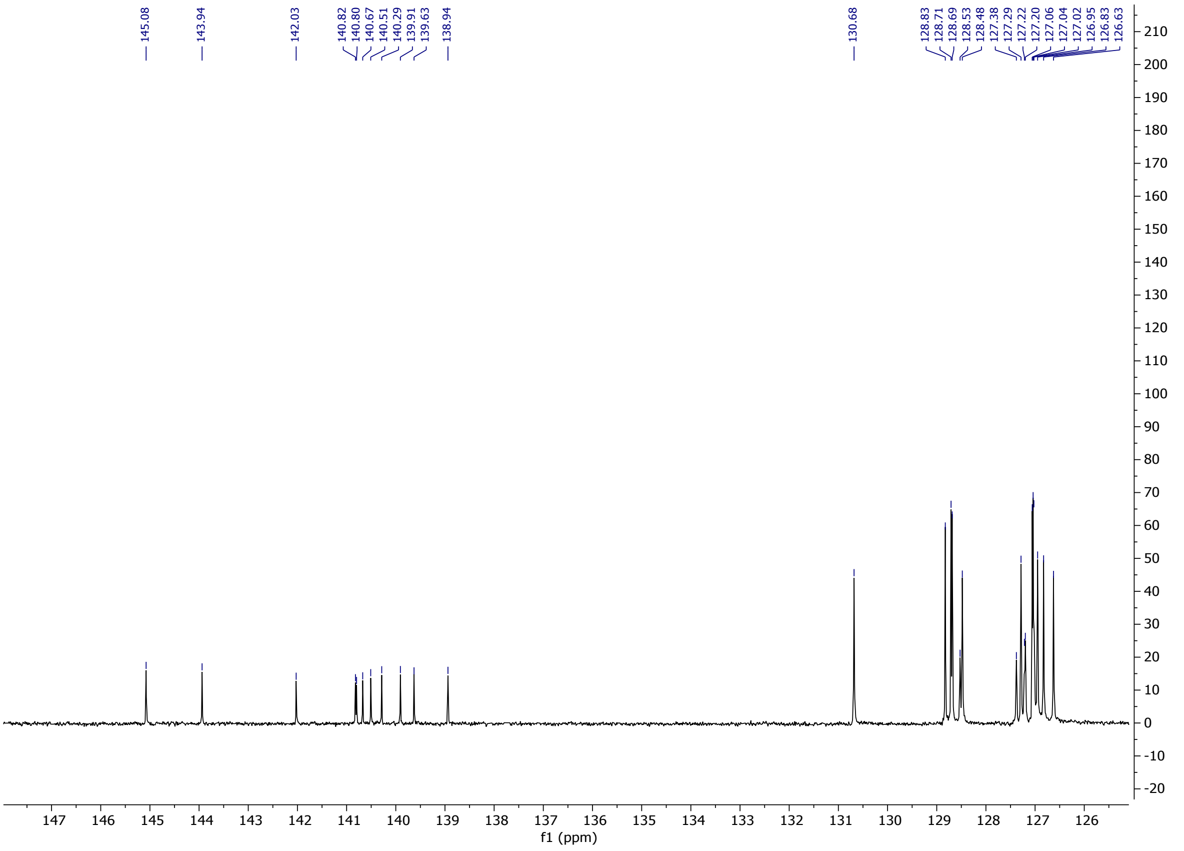

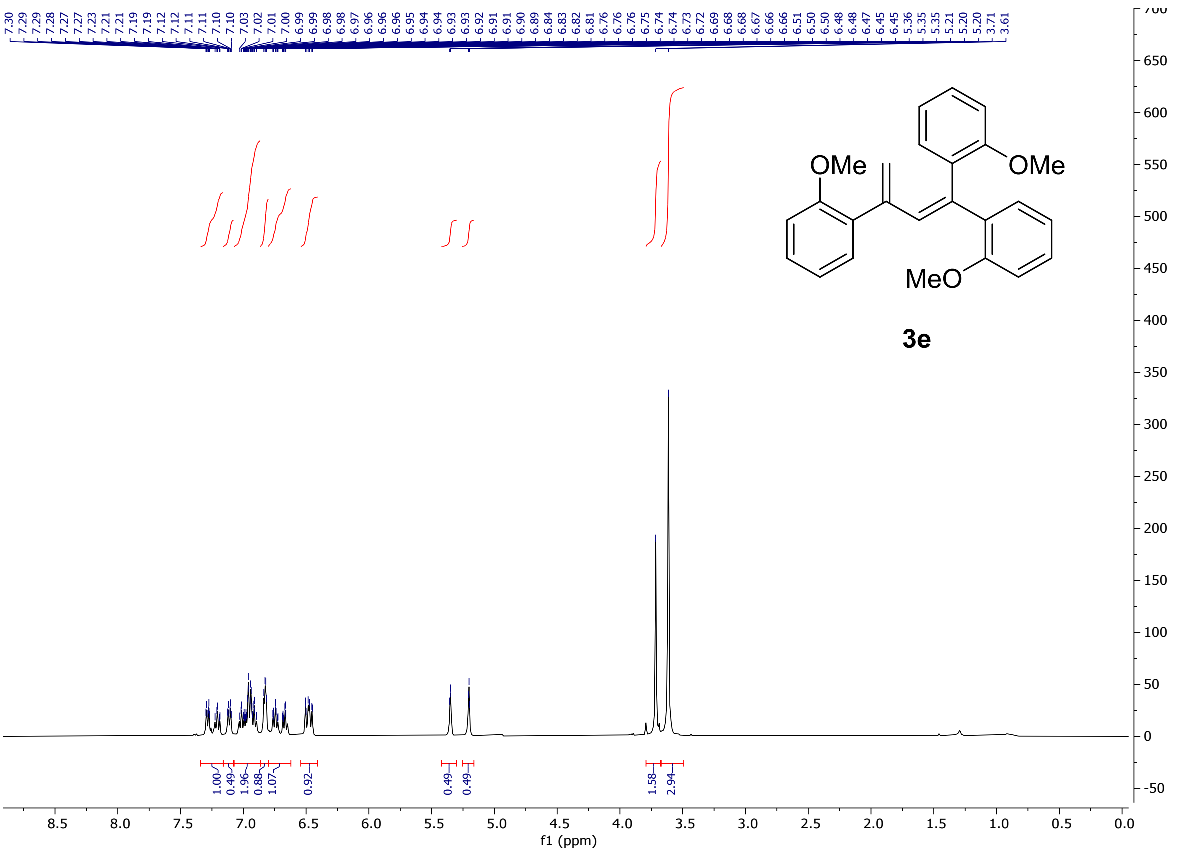

7.30 7.29 7.29 7.28 7.27 7.27 7.23 7.21 7.21 7.19 7.19 7.12 7.12 7.11 7.11 7.10 7.10 7.03 7.02 7.01 7.00 6.99 6.99 6.98 6.98 6.97 6.96 6.96 6.96 6.95 6.94 6.94 6.93 6.93 6.92 6.91 6.91 6.90 6.89 6.84 6.83 6.82 6.81 6.76 6.76 6.76 6.75 6.74 6.74 6.73 6.72 6.69 6.68 6.68 6.67 6.66 6.66 6.51 6.50 6.50 6.48 6.48 6.47 6.45 6.45 5.36 5.35 5.35 5.21 5.20 5.20 3.71 3.61
OMe
OMe
MeO
3e
1.00 0.49 1.96 0.88 1.07 0.92 0.49 0.49 1.58 2.94
f1 (ppm)

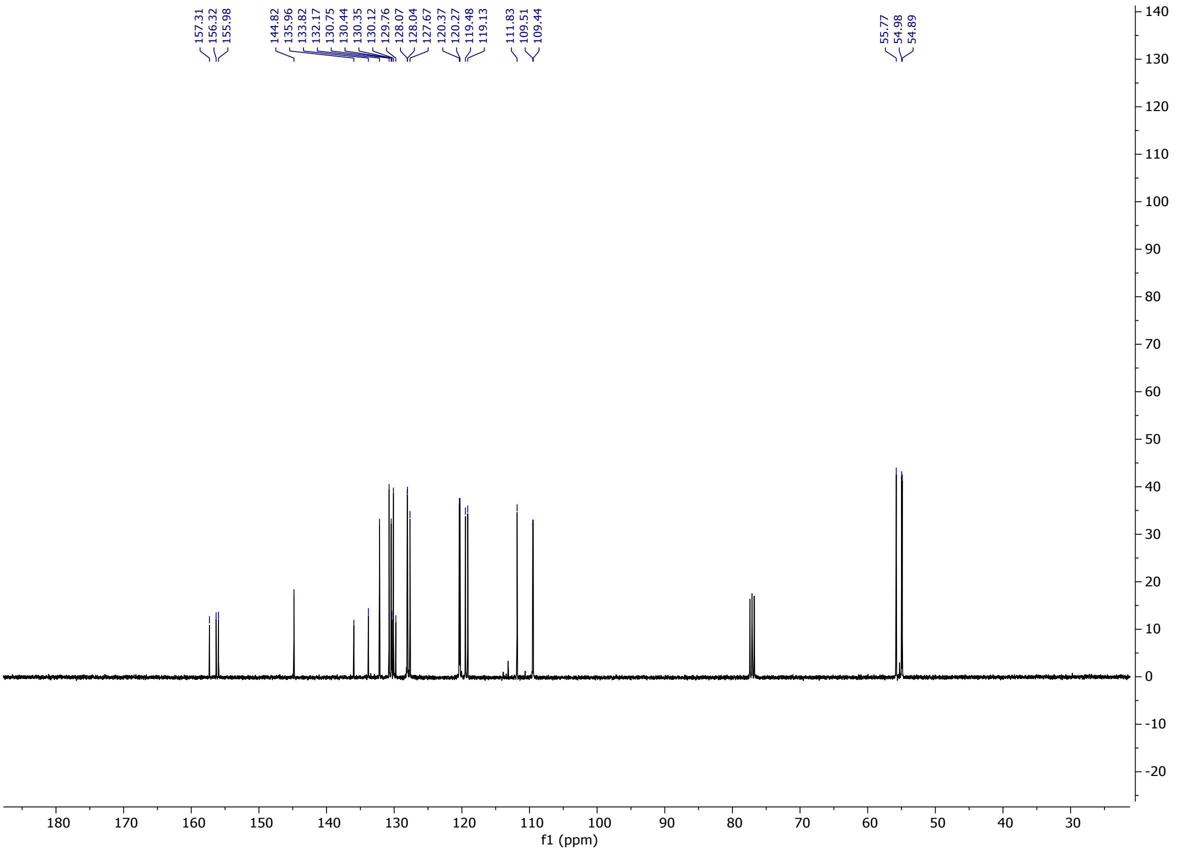

157.31 156.32 155.98 144.82 135.96 133.82 132.17 130.75 130.44 130.35 130.12 129.76 128.07 128.04 127.67 120.37 120.27 119.48 119.13 111.83 109.51 109.44 55.77 54.98 54.89
f1 (ppm)

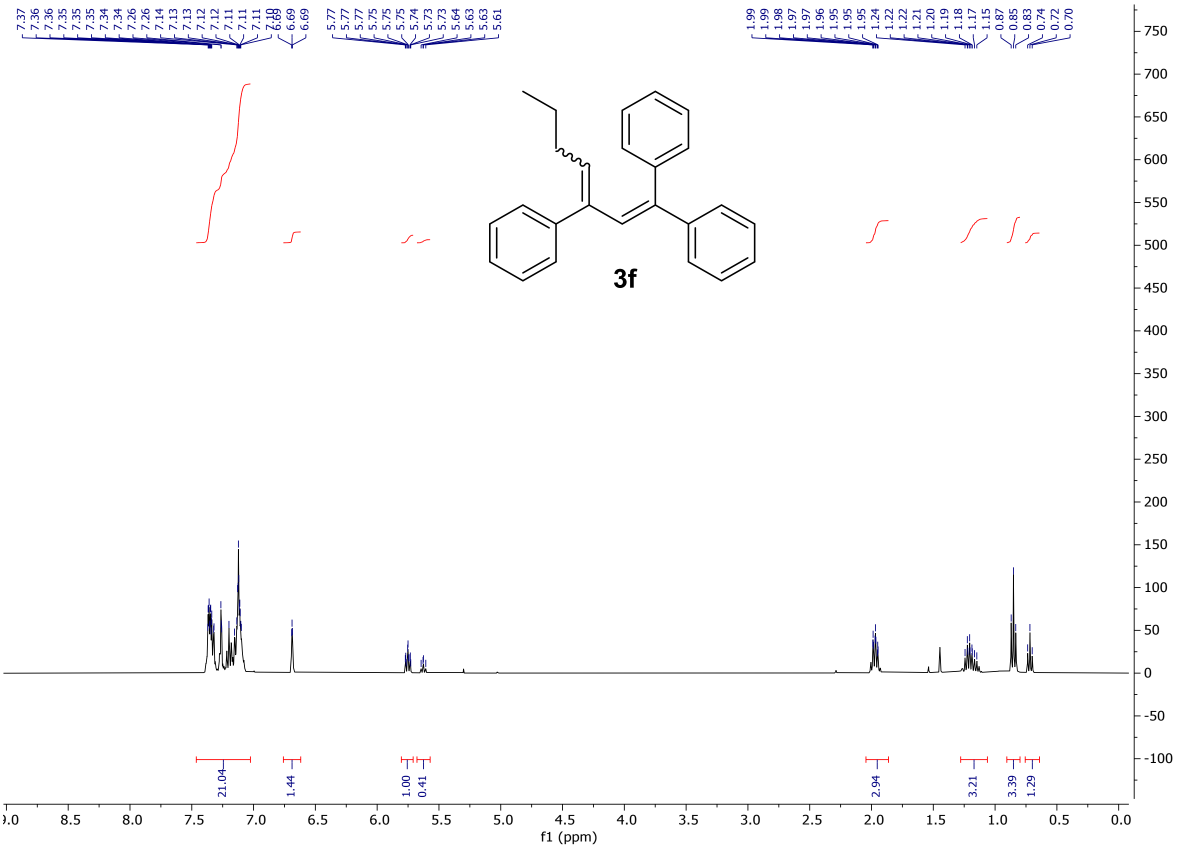
3f
f1 (ppm)

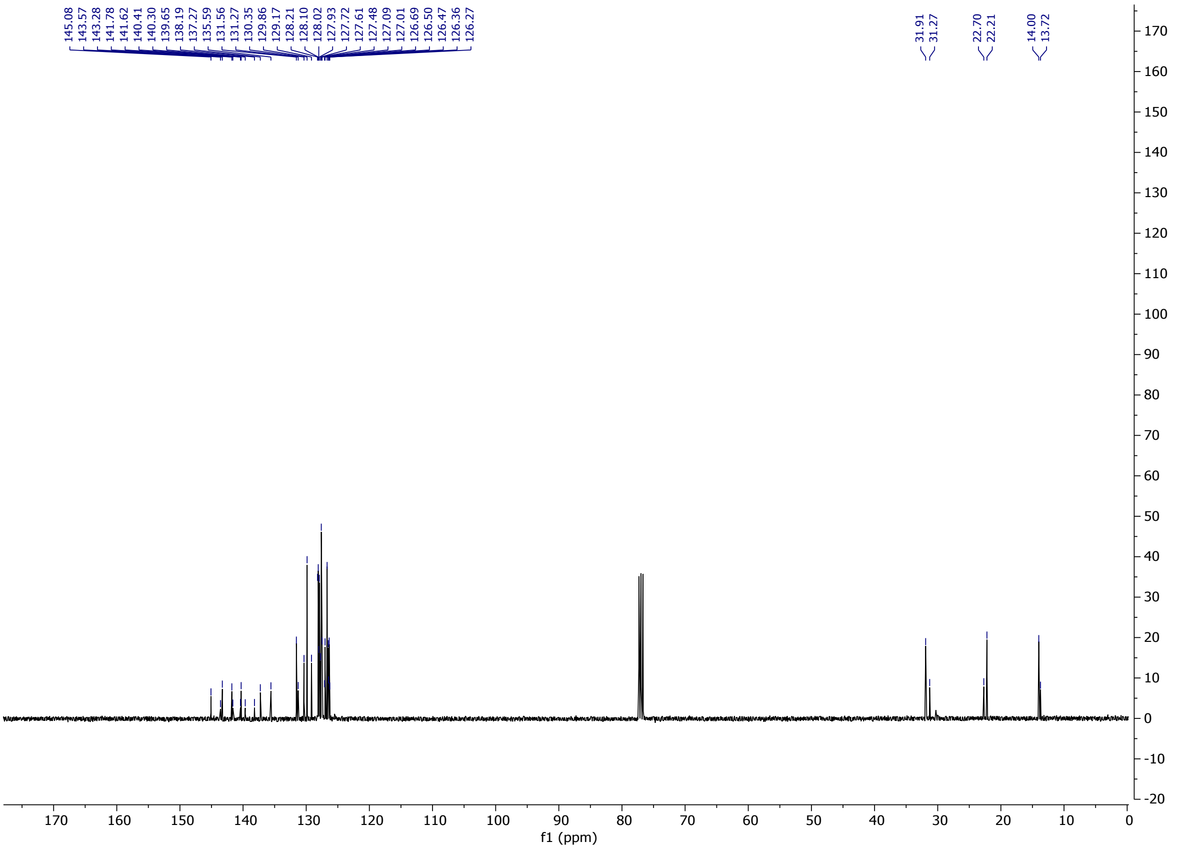
f1 (ppm)

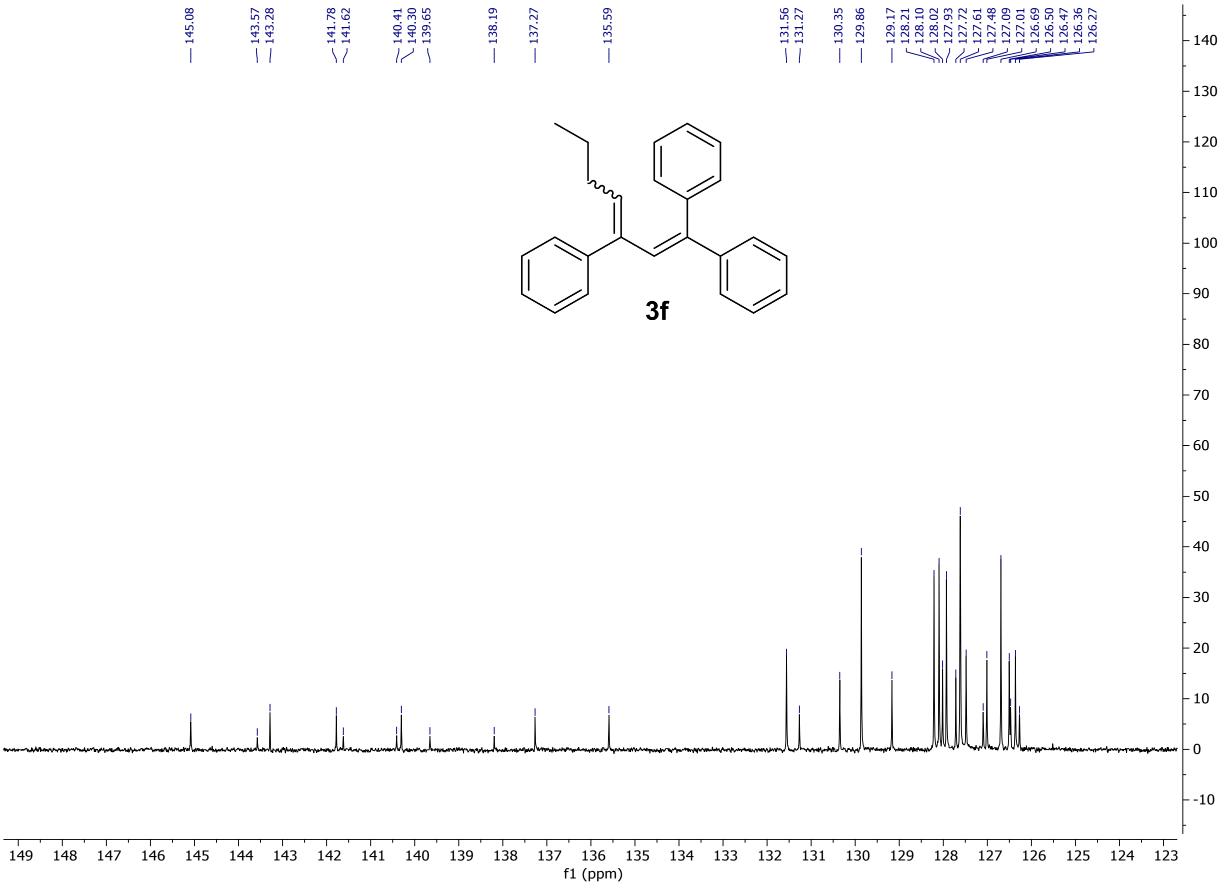

145.08
143.57
143.28
141.78
141.62
140.41
140.30
139.65
138.19
137.27
135.59
131.56
131.27
130.35
129.86
129.17
128.21
128.10
128.02
127.93
127.72
127.61
127.48
127.09
127.01
126.69
126.50
126.47
126.36
126.27
3f
f1 (ppm)

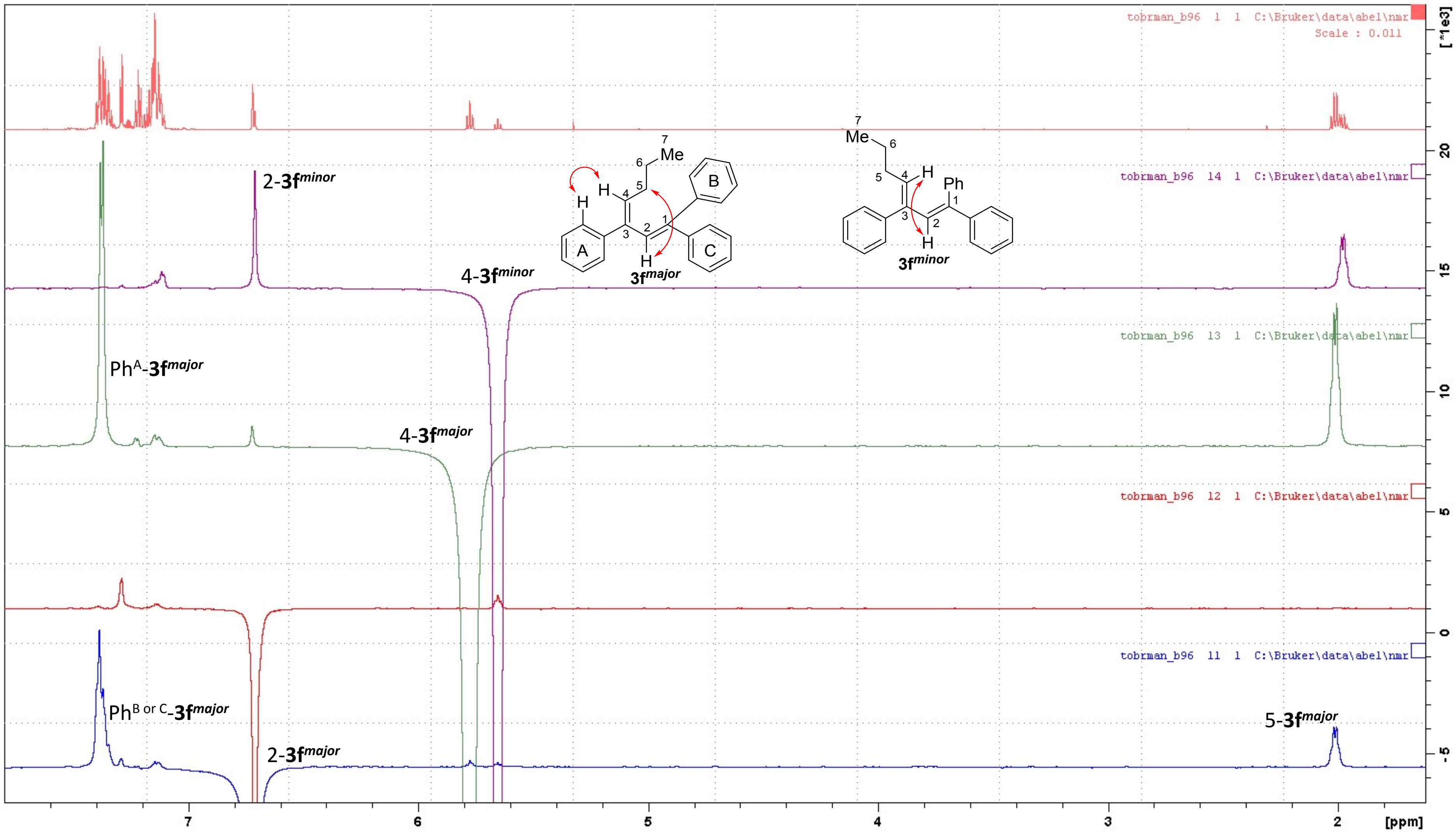
tobrman_b96 1 1 C:\Bruker\data\abel\nmr
Scale : 0.011
tobrman_b96 14 1 C:\Bruker\data\abel\nmr
tobrman_b96 13 1 C:\Bruker\data\abel\nmr
tobrman_b96 12 1 C:\Bruker\data\abel\nmr
tobrman_b96 11 1 C:\Bruker\data\abel\nmr
2-3f minor
4-3f minor
3f major
3f minor
PhA-3f major
4-3f major
PhB or C-3f major
2-3f major
5-3f major
[ppm]
[*1e3]

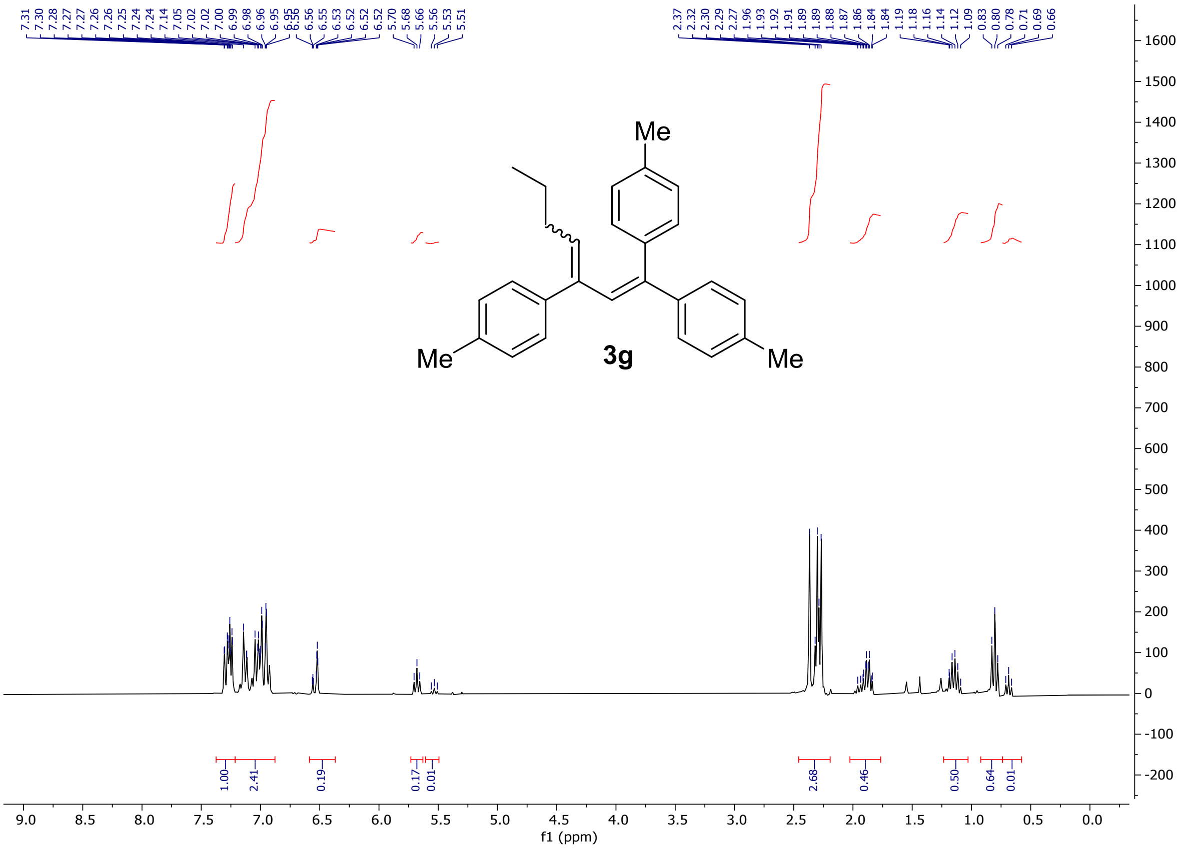
Me
Me
Me
3g
f1 (ppm)

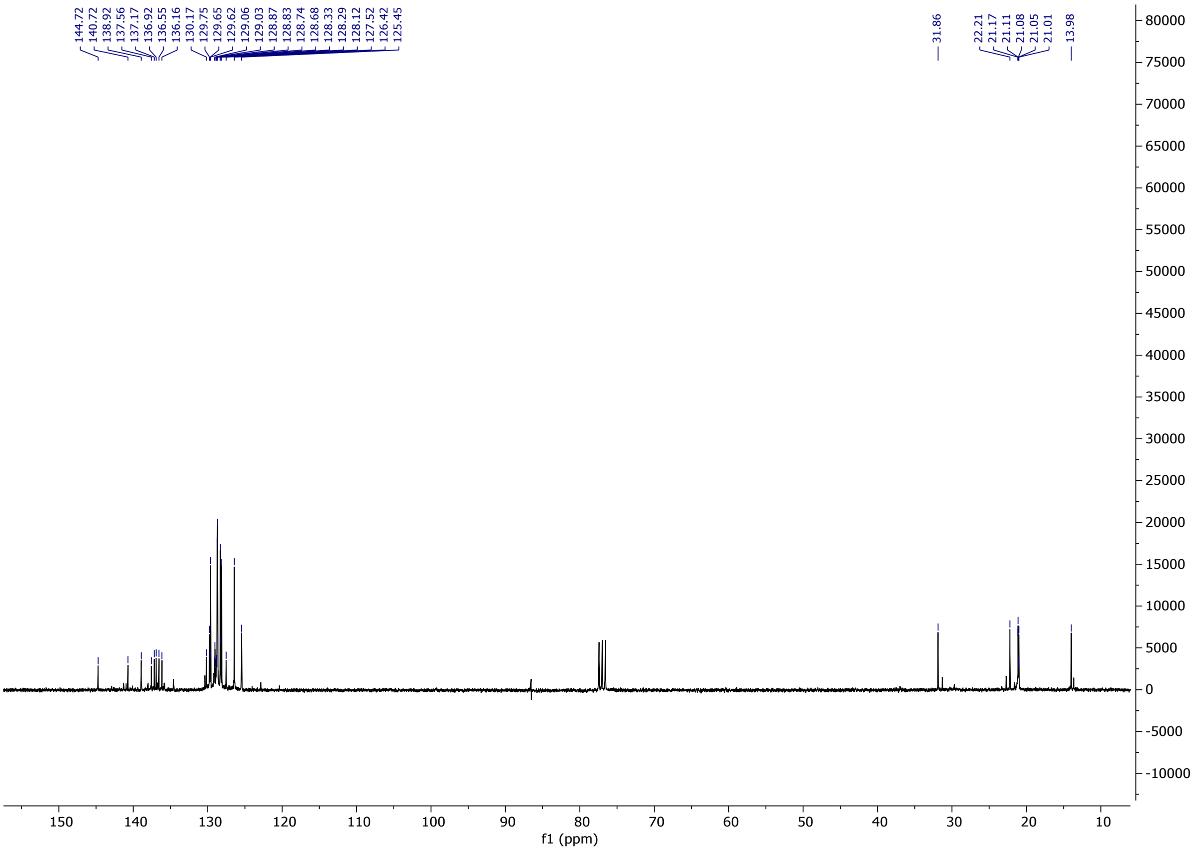
f1 (ppm)

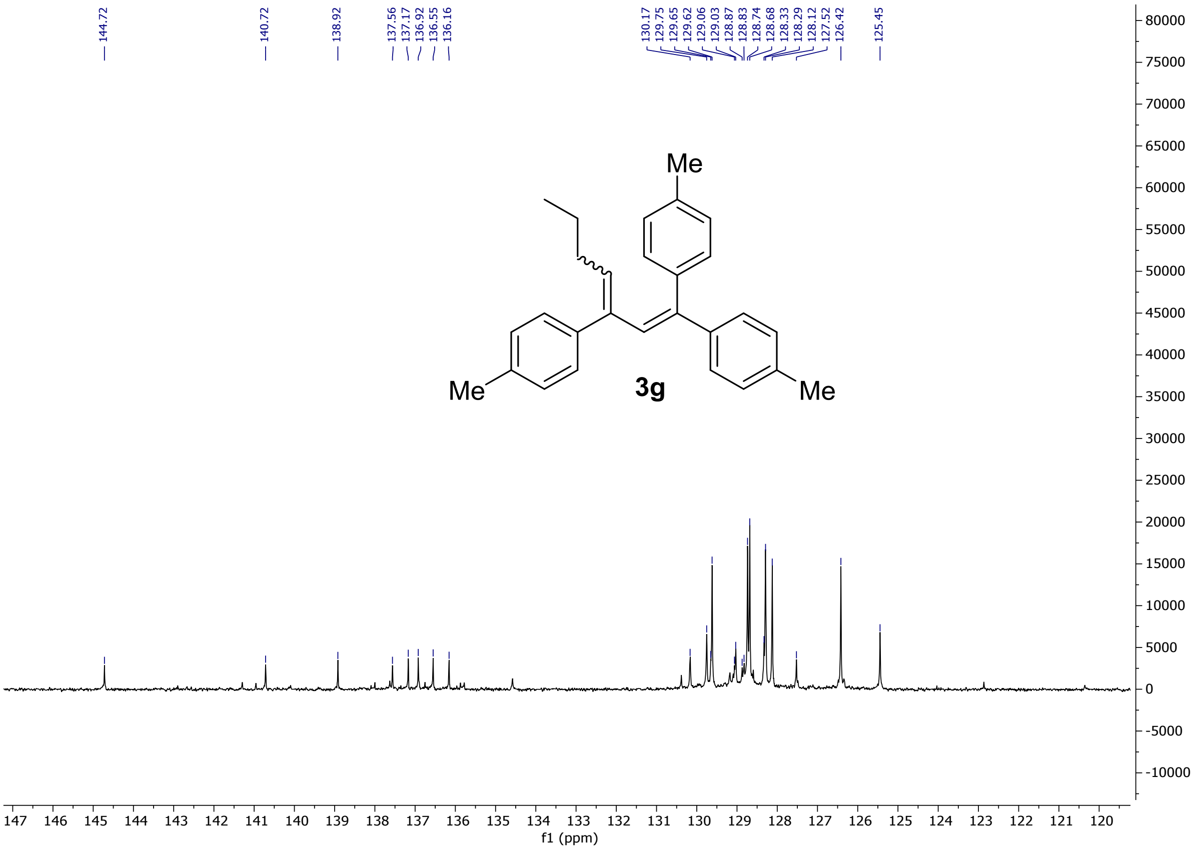
144.72
140.72
138.92
137.56
137.17
136.92
136.55
136.16
130.17
129.75
129.65
129.62
129.06
129.03
128.87
128.83
128.74
128.68
128.33
128.29
128.12
127.52
126.42
125.45
Me
Me
Me
3g
f1 (ppm)

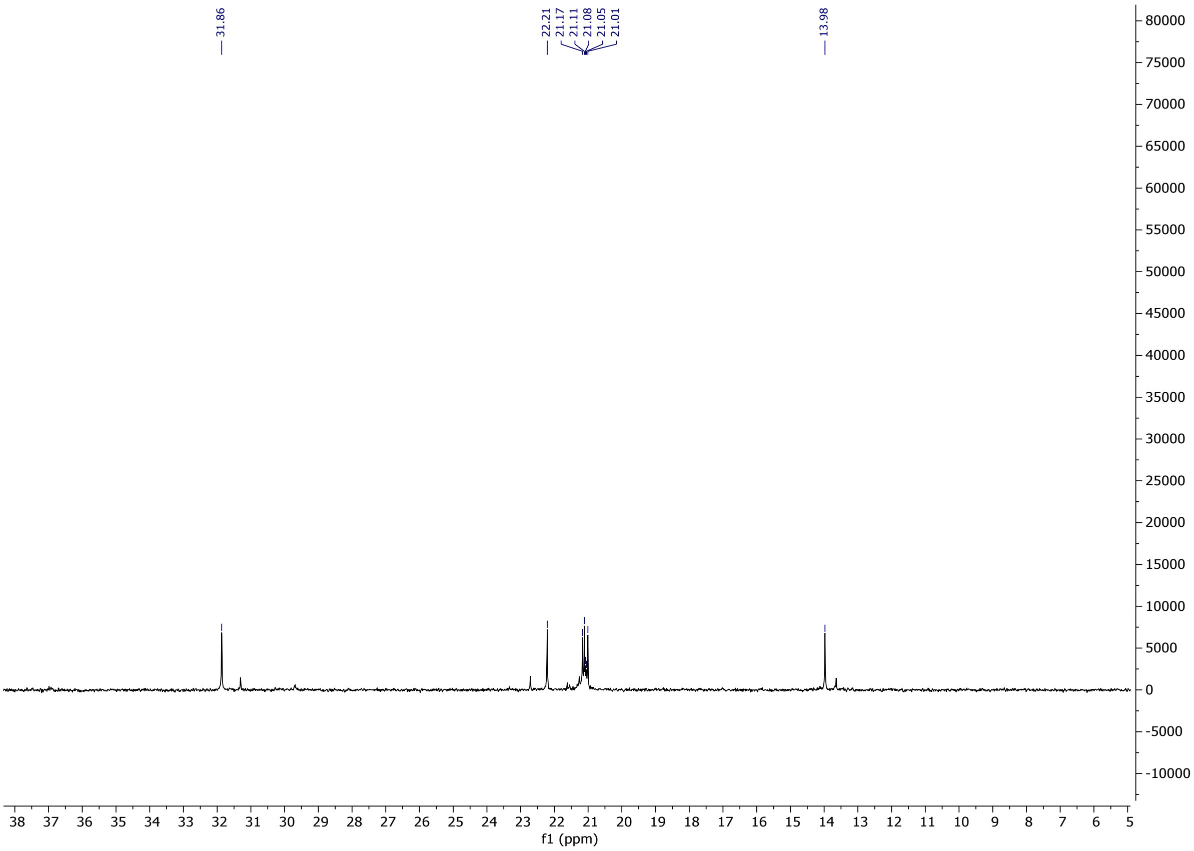
31.86
22.21
21.17
21.11
21.08
21.05
21.01
13.98
f1 (ppm)

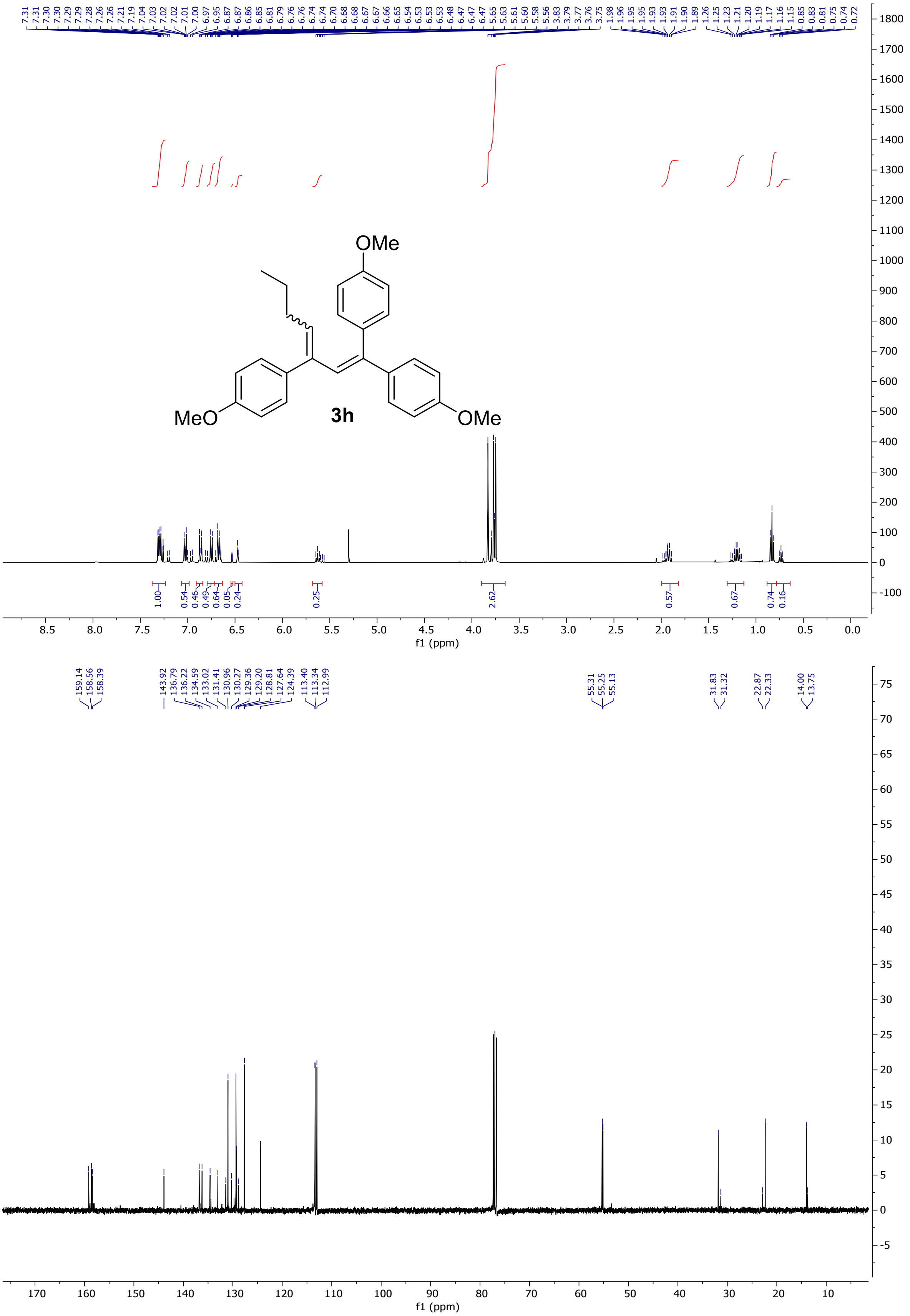

OMe
MeO
3h
OMe
f1 (ppm)
f1 (ppm)

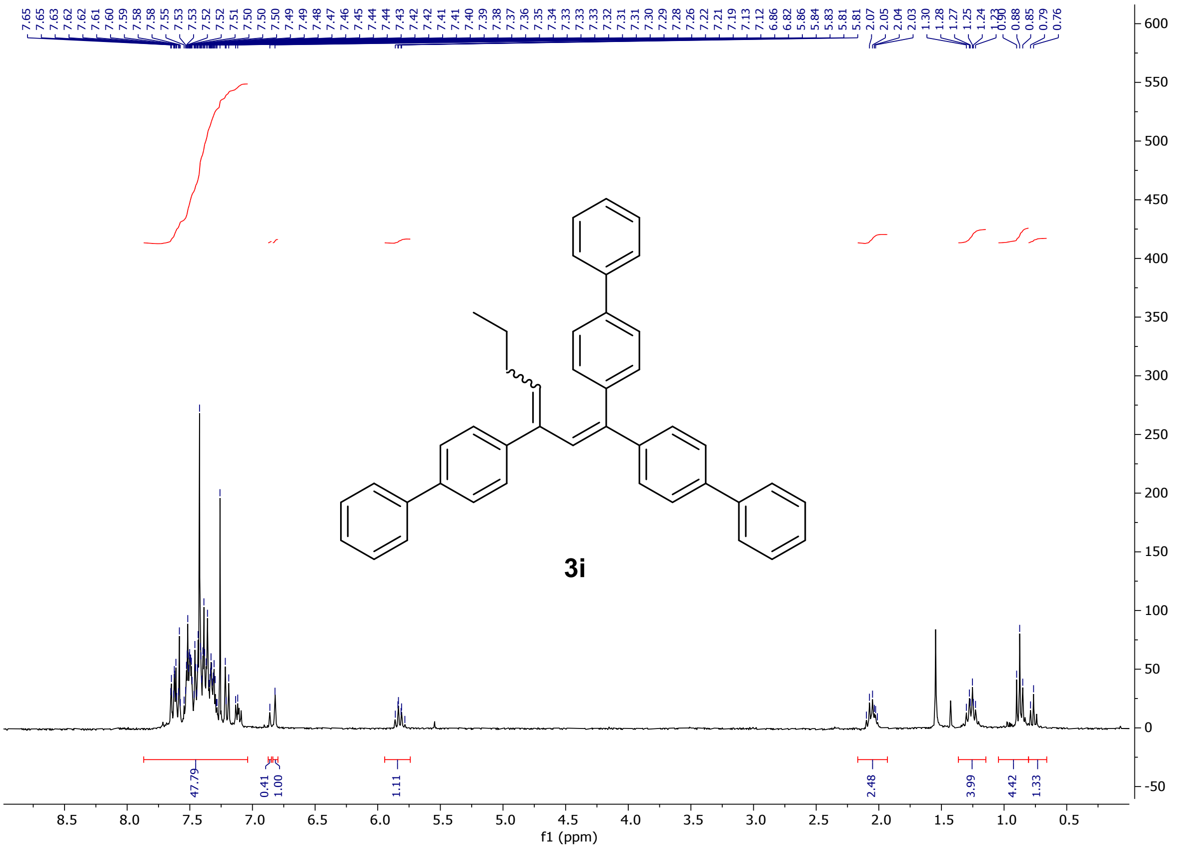

3i
f1 (ppm)
47.79
0.41
1.00
1.11
2.48
3.99
4.42
1.33

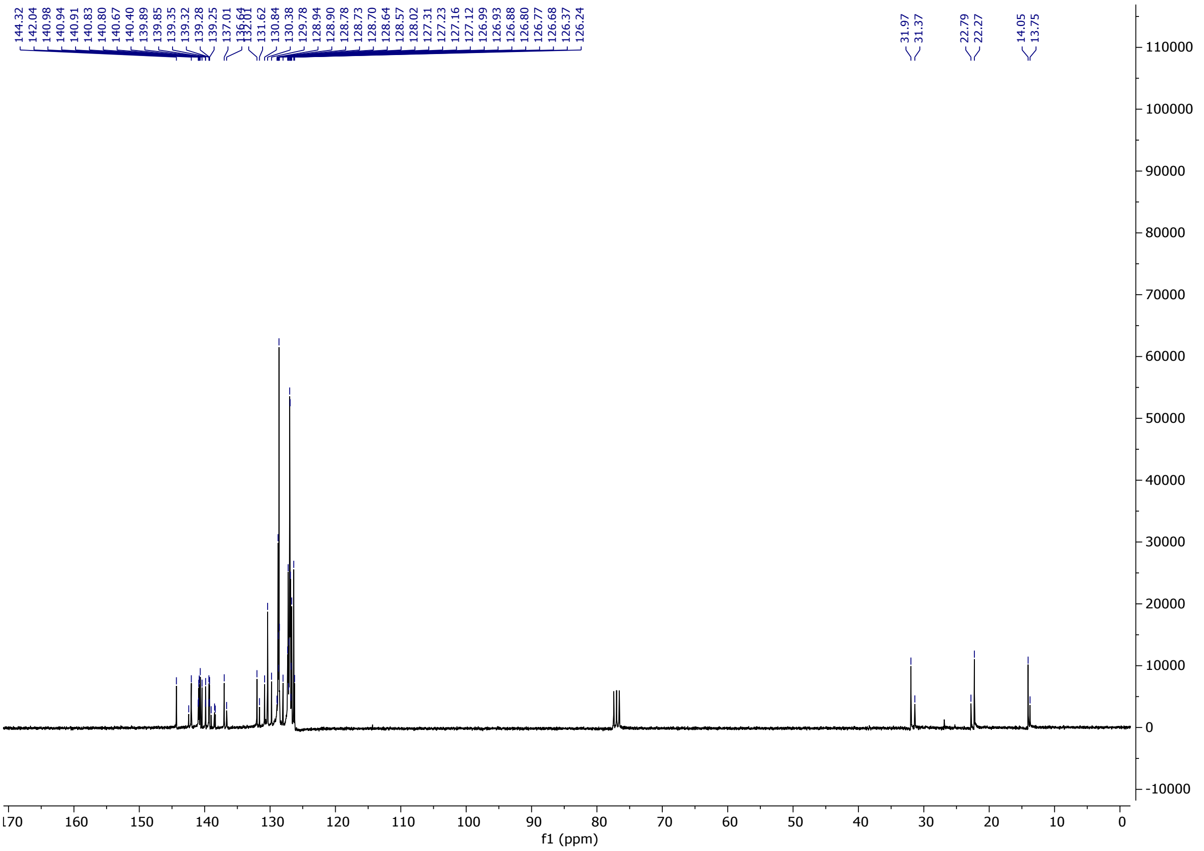

144.32
142.04
140.98
140.94
140.91
140.83
140.80
140.67
140.40
139.89
139.85
139.35
139.32
139.28
139.25
137.01
131.62
130.84
130.38
129.78
128.94
128.90
128.78
128.73
128.70
128.64
128.57
128.02
127.31
127.23
127.16
127.12
126.99
126.93
126.88
126.80
126.77
126.68
126.37
126.24
31.97
31.37
22.79
22.27
14.05
13.75
f1 (ppm)

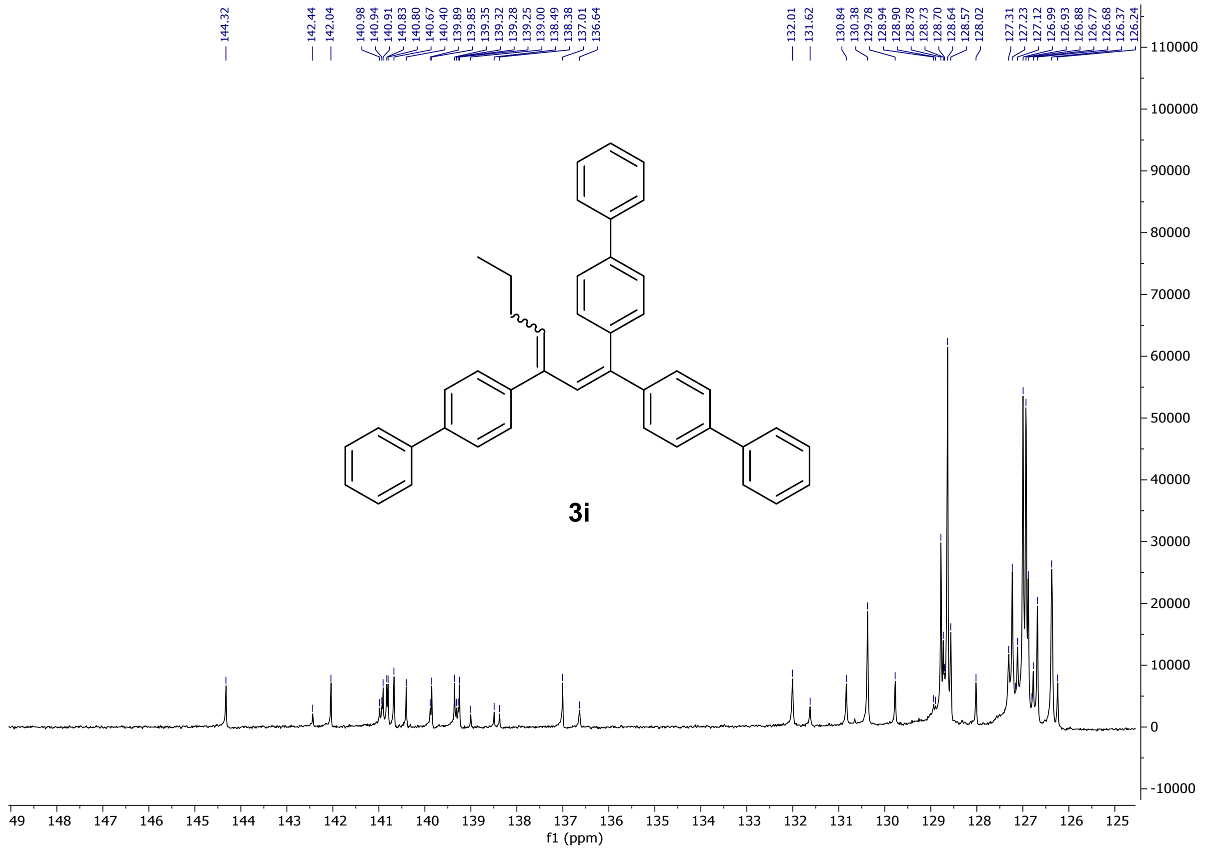
144.32
142.44
142.04
140.98
140.94
140.91
140.83
140.80
140.67
140.40
139.89
139.85
139.35
139.32
139.28
139.25
139.00
138.49
138.38
137.01
136.64
132.01
131.62
130.84
130.38
129.78
128.94
128.90
128.78
128.73
128.70
128.64
128.57
128.02
127.31
127.23
127.12
126.99
126.93
126.88
126.77
126.68
126.37
126.24
3i
f1 (ppm)

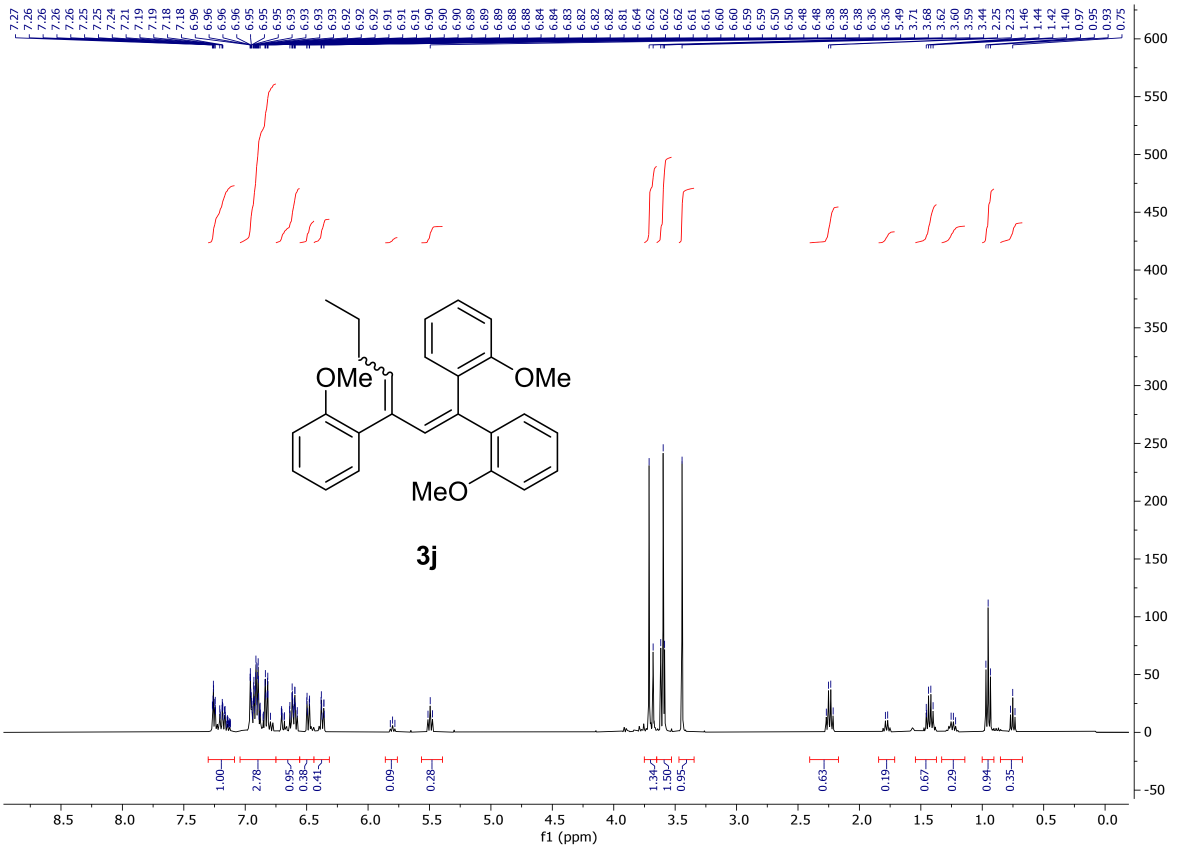
OMe
OMe
MeO
3j
f1 (ppm)

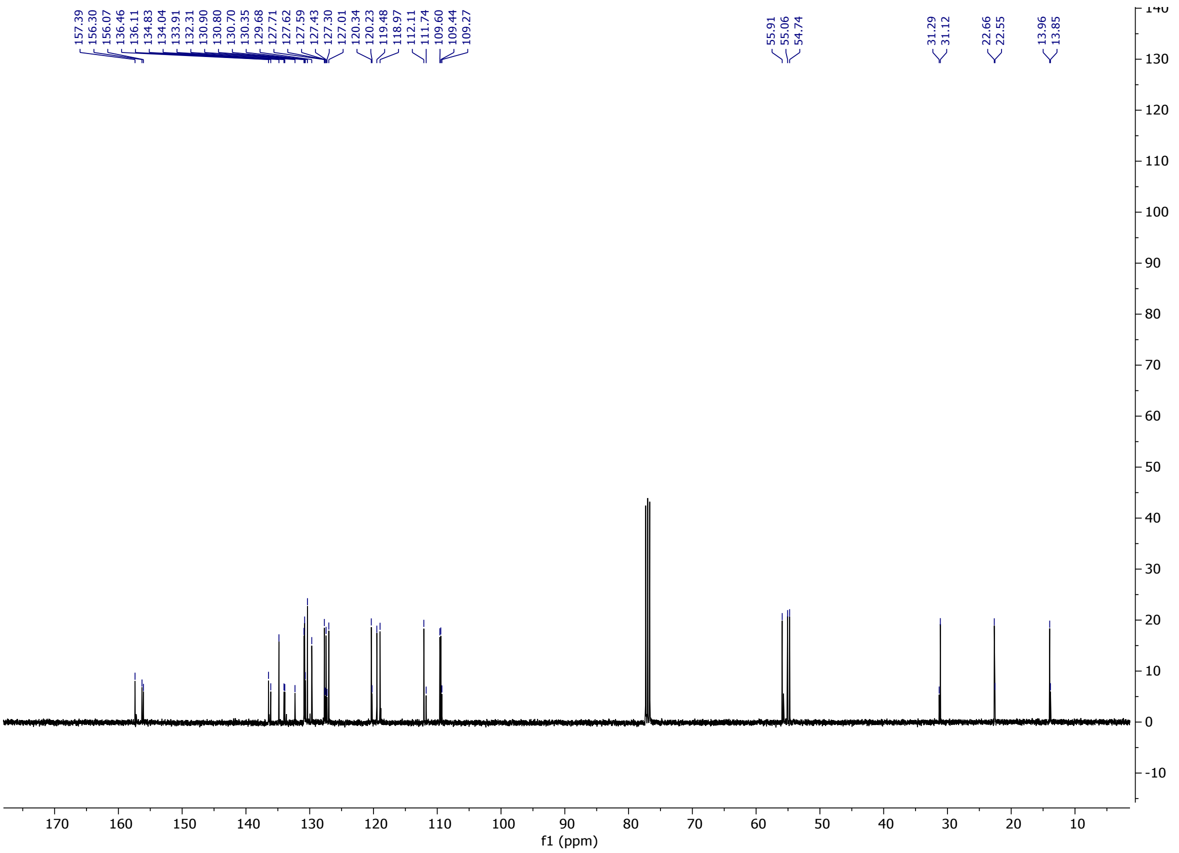
f1 (ppm)

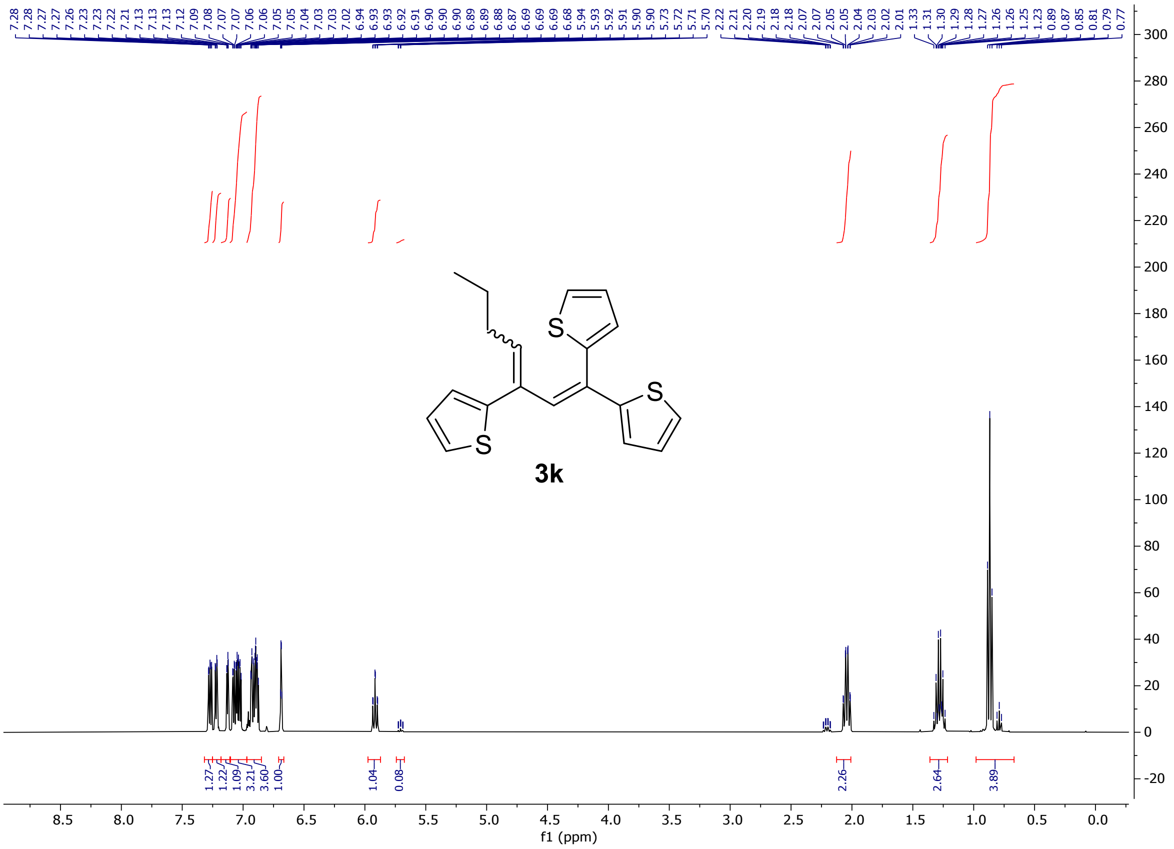
7.28 7.28 7.27 7.26 7.23 7.23 7.22 7.21 7.13 7.13 7.13 7.12 7.09 7.08 7.07 7.07 7.06 7.06 7.05 7.05 7.04 7.03 7.03 7.02 6.94 6.93 6.93 6.92 6.91 6.90 6.90 6.90 6.89 6.89 6.88 6.87 6.69 6.69 6.69 6.68 5.94 5.93 5.92 5.91 5.90 5.90 5.73 5.72 5.71 5.70 2.22 2.21 2.20 2.19 2.18 2.18 2.07 2.07 2.05 2.05 2.04 2.03 2.02 2.01 1.33 1.31 1.30 1.29 1.28 1.27 1.26 1.26 1.25 1.23 0.89 0.87 0.85 0.81 0.79 0.77
S
S
S
3k
1.27 1.22 1.09 3.21 3.60 1.00 1.04 0.08 2.26 2.64 3.89
f1 (ppm)

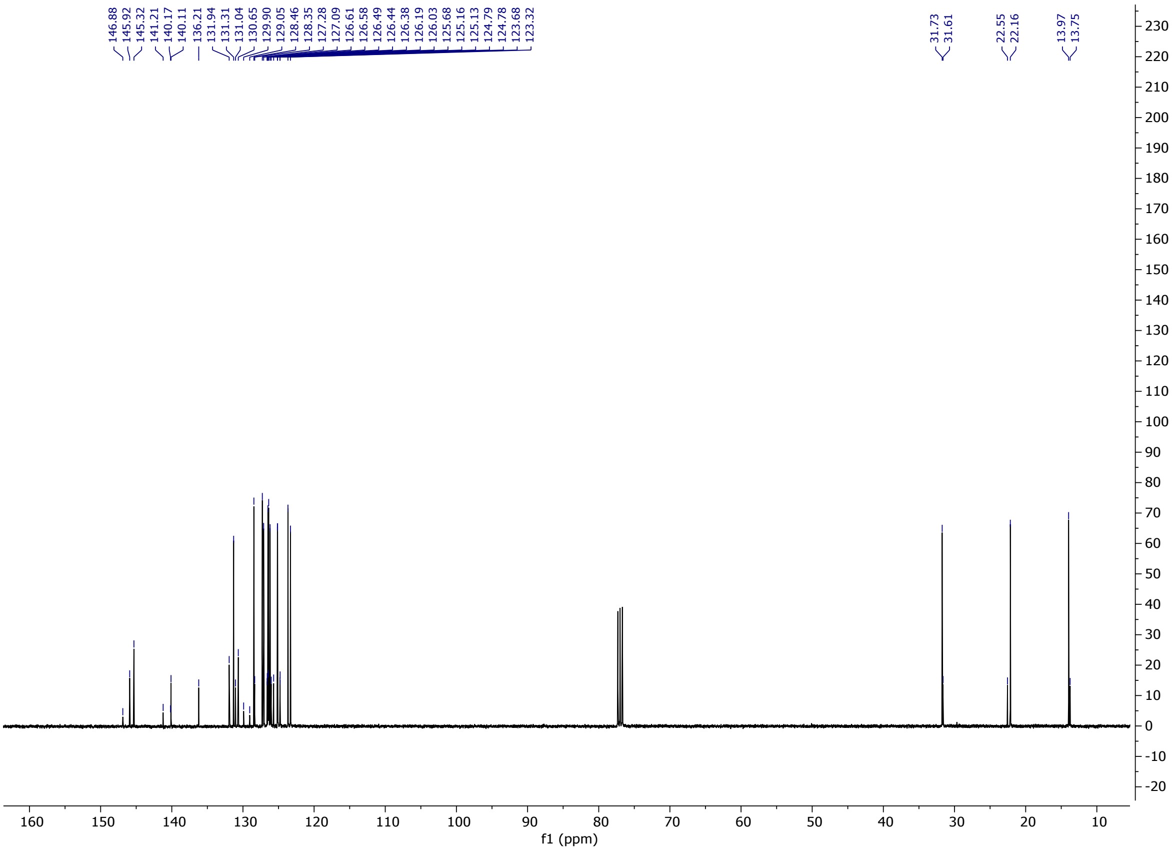
146.88 145.92 145.32 141.21 140.17 140.11 136.21 131.94 131.31 131.04 130.65 129.90 129.05 128.46 128.35 127.28 127.09 126.61 126.58 126.49 126.44 126.38 126.19 126.03 125.68 125.16 125.13 124.79 124.78 123.68 123.32
31.73 31.61 22.55 22.16 13.97 13.75
f1 (ppm)

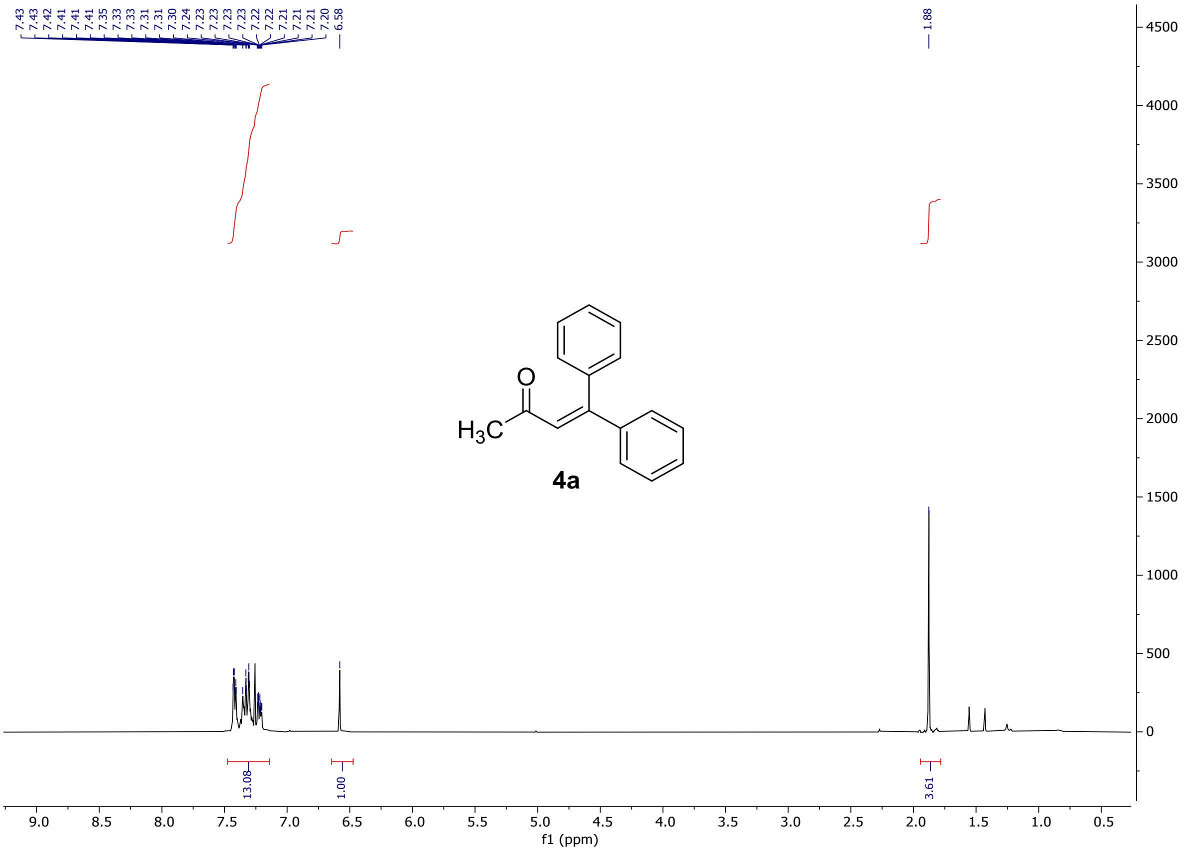
7.43
7.43
7.42
7.41
7.41
7.41
7.35
7.33
7.33
7.31
7.31
7.30
7.24
7.23
7.23
7.23
7.23
7.22
7.22
7.21
7.21
7.21
7.20
6.58
1.88
O
H3C
4a
13.08
1.00
3.61
4500
4000
3500
3000
2500
2000
1500
1000
500
0
9.0
8.5
8.0
7.5
7.0
6.5
6.0
5.5
5.0
4.5
4.0
3.5
3.0
2.5
2.0
1.5
1.0
0.5
f1 (ppm)

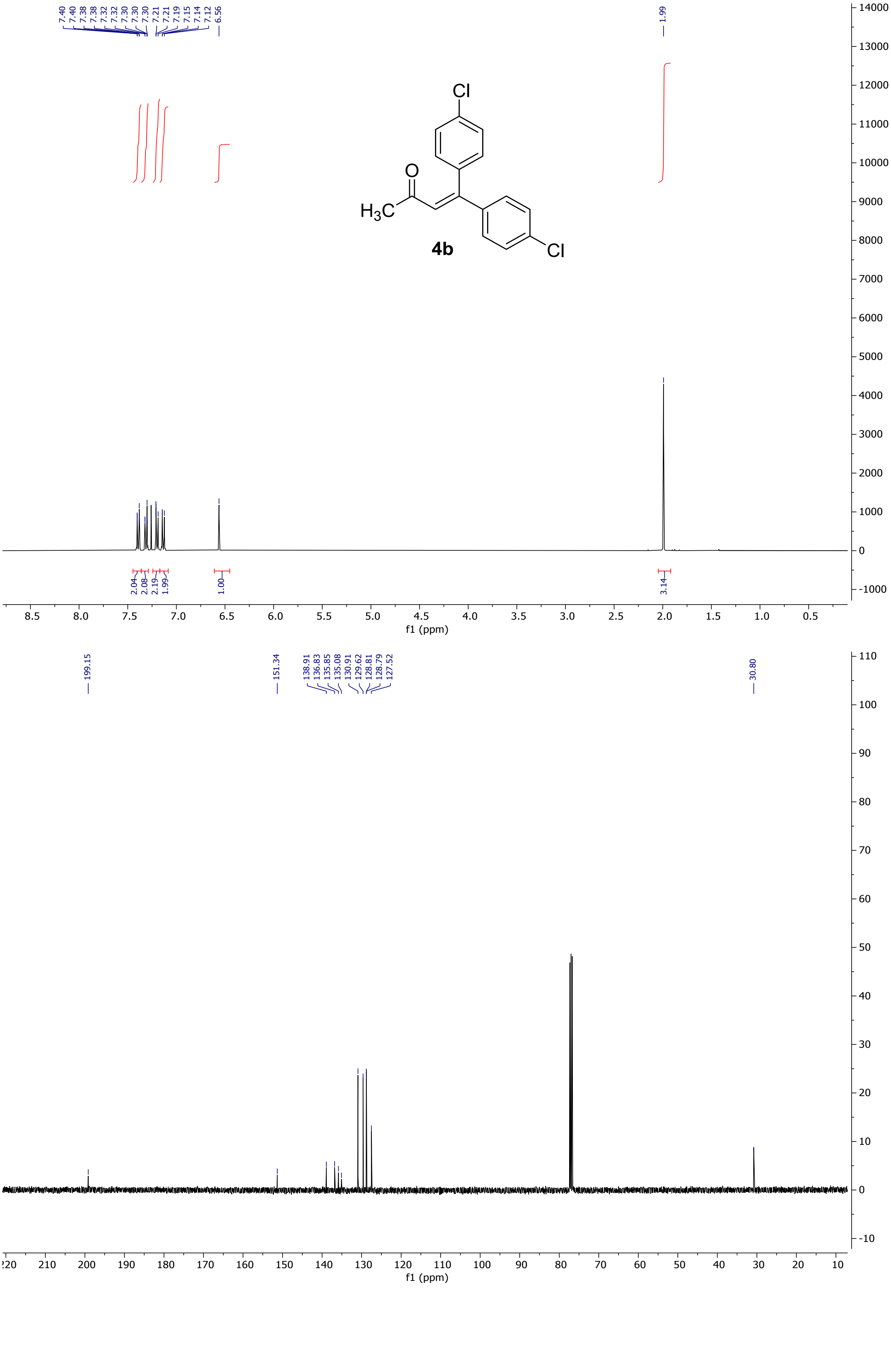
7.40
7.40
7.38
7.38
7.32
7.32
7.30
7.30
7.30
7.21
7.21
7.19
7.15
7.14
7.12
6.56
1.99
Cl
O
H_3C
Cl
2.04
2.08
2.19
1.99
1.00
3.14
f1 (ppm)
199.15
151.34
138.91
136.83
135.85
135.08
130.91
129.62
128.81
128.79
127.52
30.80
f1 (ppm)

4b

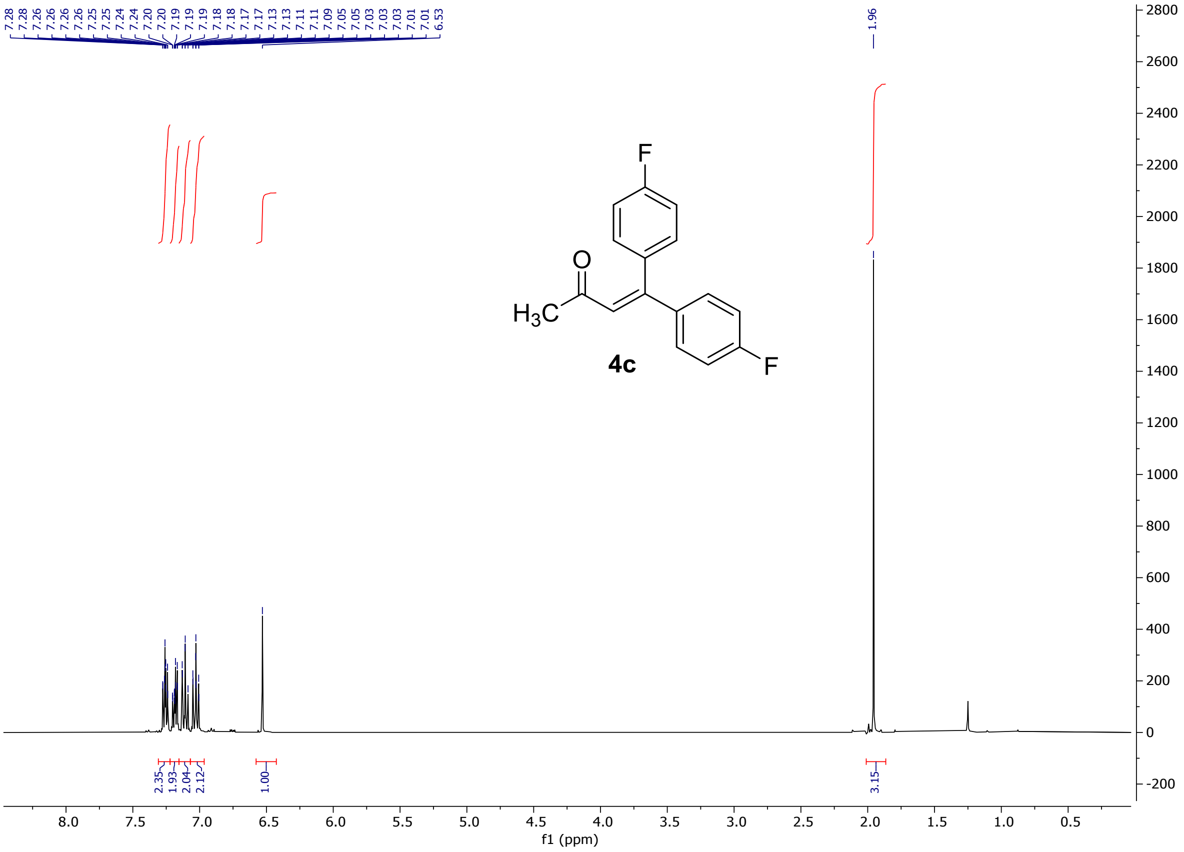
7.28
7.28
7.26
7.26
7.26
7.26
7.25
7.25
7.24
7.24
7.20
7.20
7.19
7.19
7.19
7.19
7.18
7.18
7.17
7.17
7.13
7.13
7.11
7.11
7.09
7.05
7.05
7.03
7.03
7.03
7.01
7.01
6.53
1.96
2.35
1.93
2.04
2.12
1.00
3.15
f1 (ppm)
F
O
H3C
F

4c

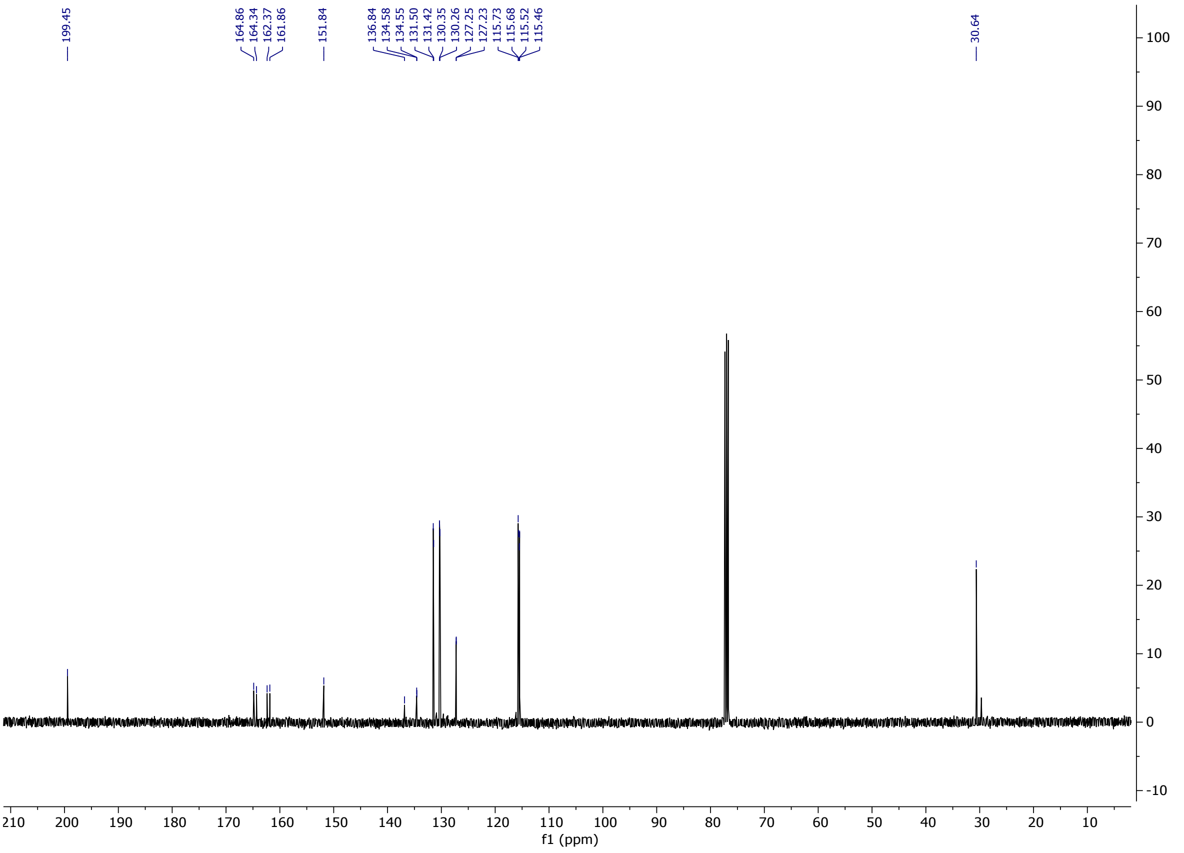
199.45
164.86
164.34
162.37
161.86
151.84
136.84
134.58
134.55
131.50
131.42
130.35
130.26
127.25
127.23
115.73
115.68
115.52
115.46
30.64
f1 (ppm)

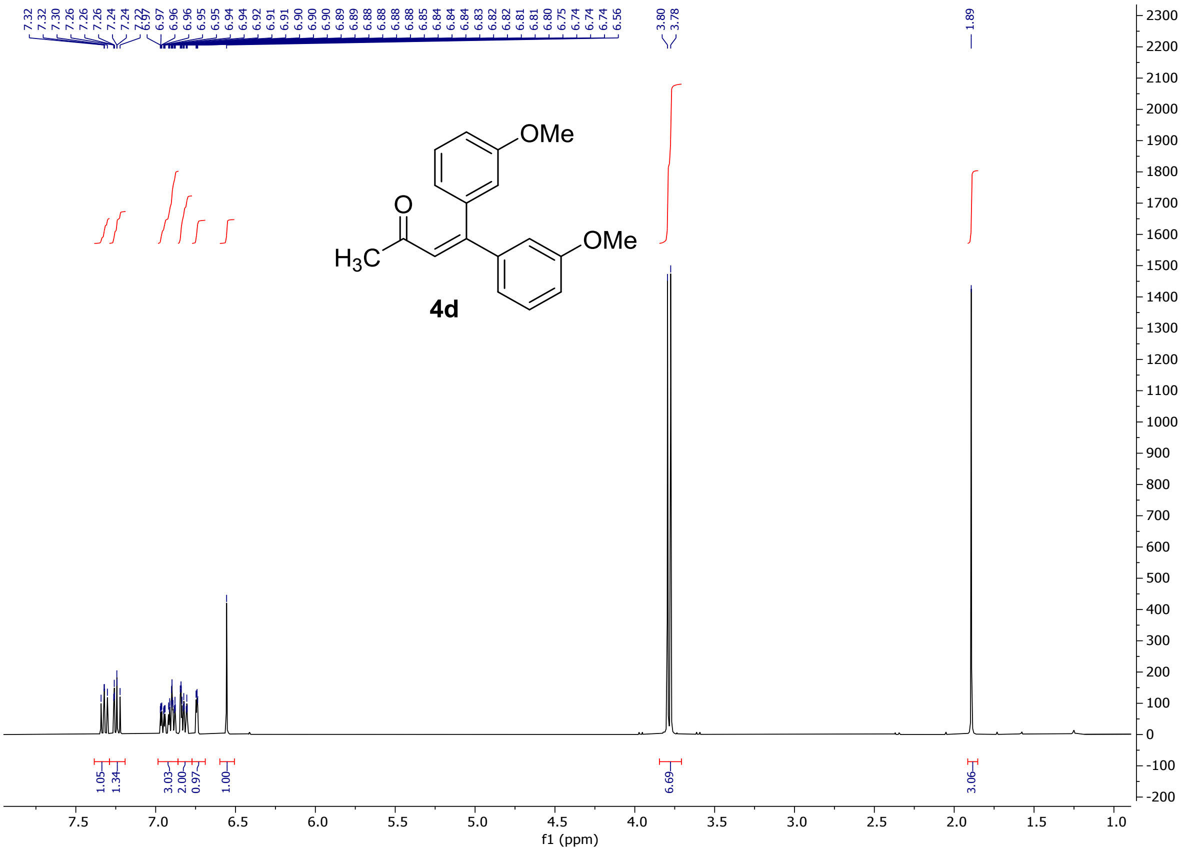

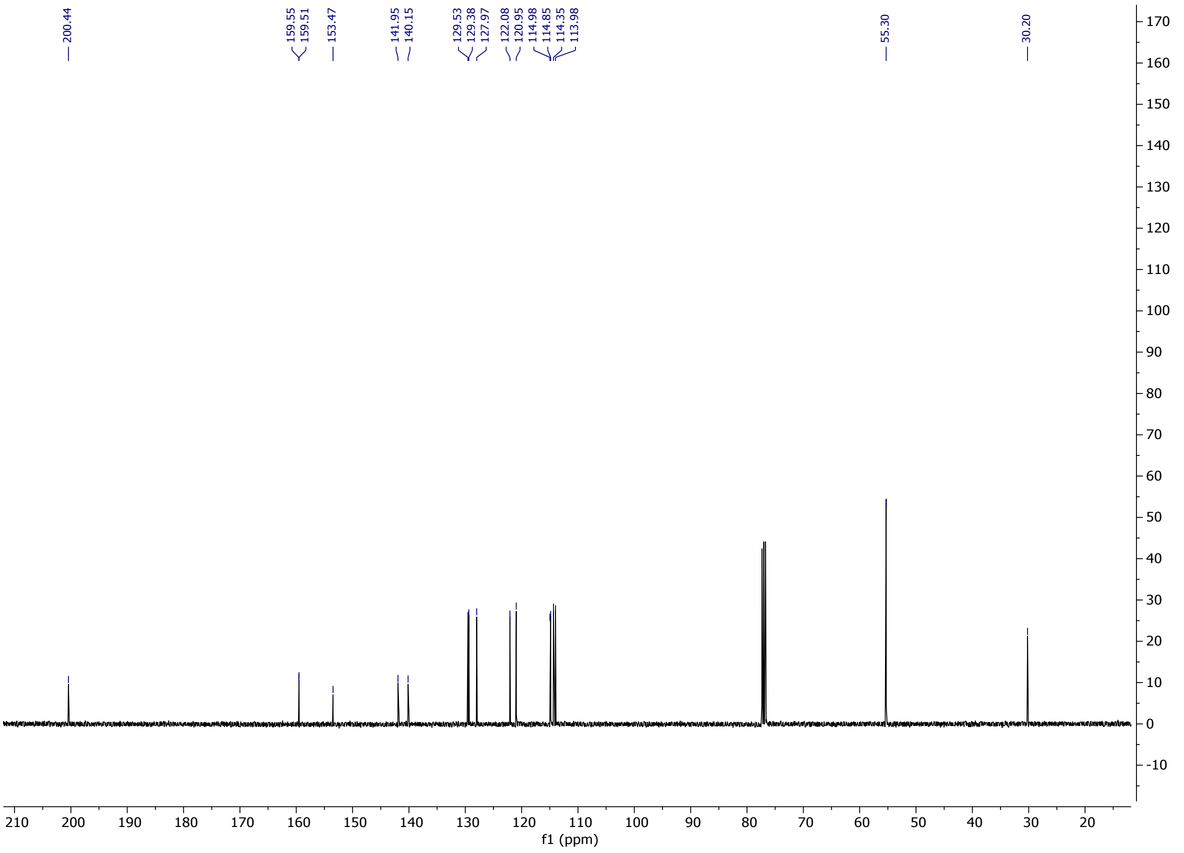

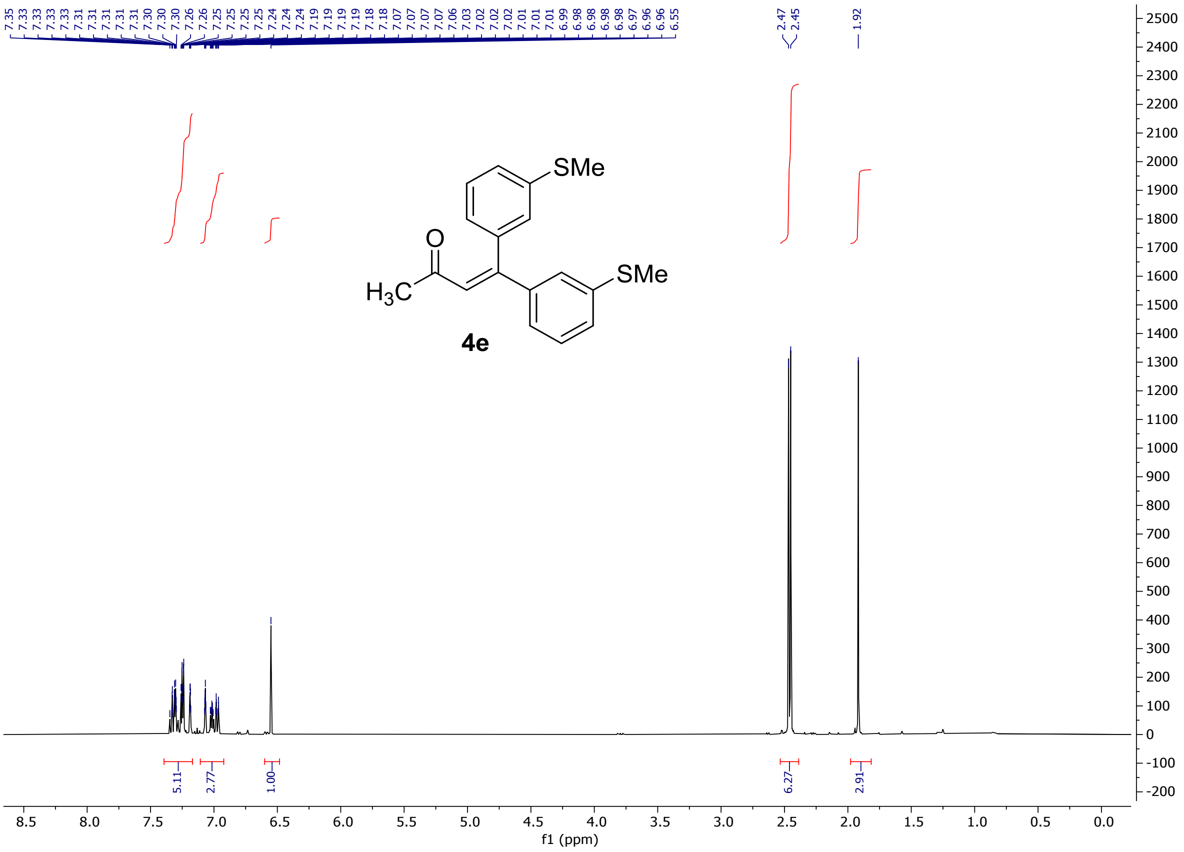
7.35 7.33 7.33 7.33 7.33 7.31 7.31 7.31 7.31 7.31 7.30 7.30 7.30 7.26 7.26 7.25 7.25 7.25 7.25 7.24 7.24 7.24 7.19 7.19 7.19 7.19 7.18 7.18 7.07 7.07 7.07 7.07 7.06 7.03 7.02 7.02 7.02 7.01 7.01 7.01 6.99 6.98 6.98 6.98 6.98 6.97 6.96 6.96 6.55
2.47 2.45
1.92
SMe
SMe
O
H3C
4e
5.11
2.77
1.00
6.27
2.91
f1 (ppm)

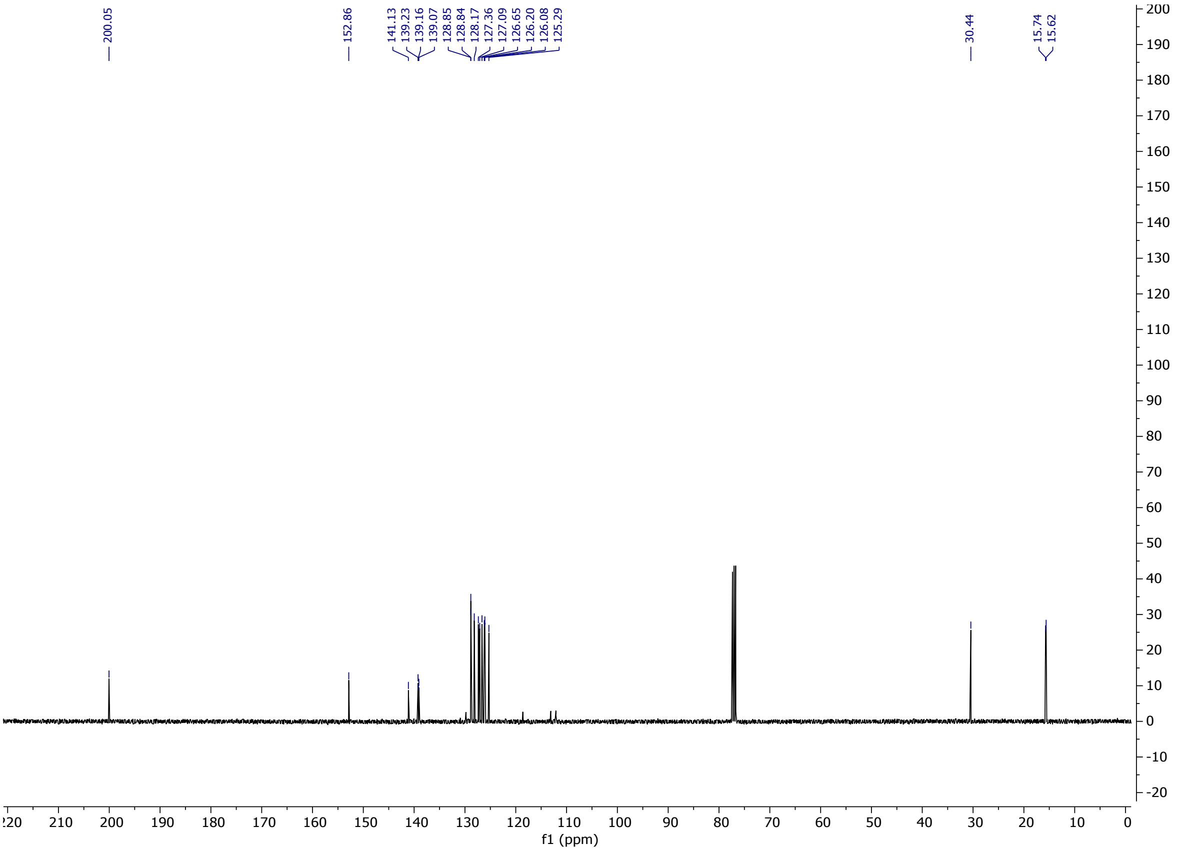
200.05
152.86
141.13 139.23 139.16 139.07 128.85 128.84 128.17 127.36 127.09 126.65 126.20 126.08 125.29
30.44
15.74 15.62
f1 (ppm)

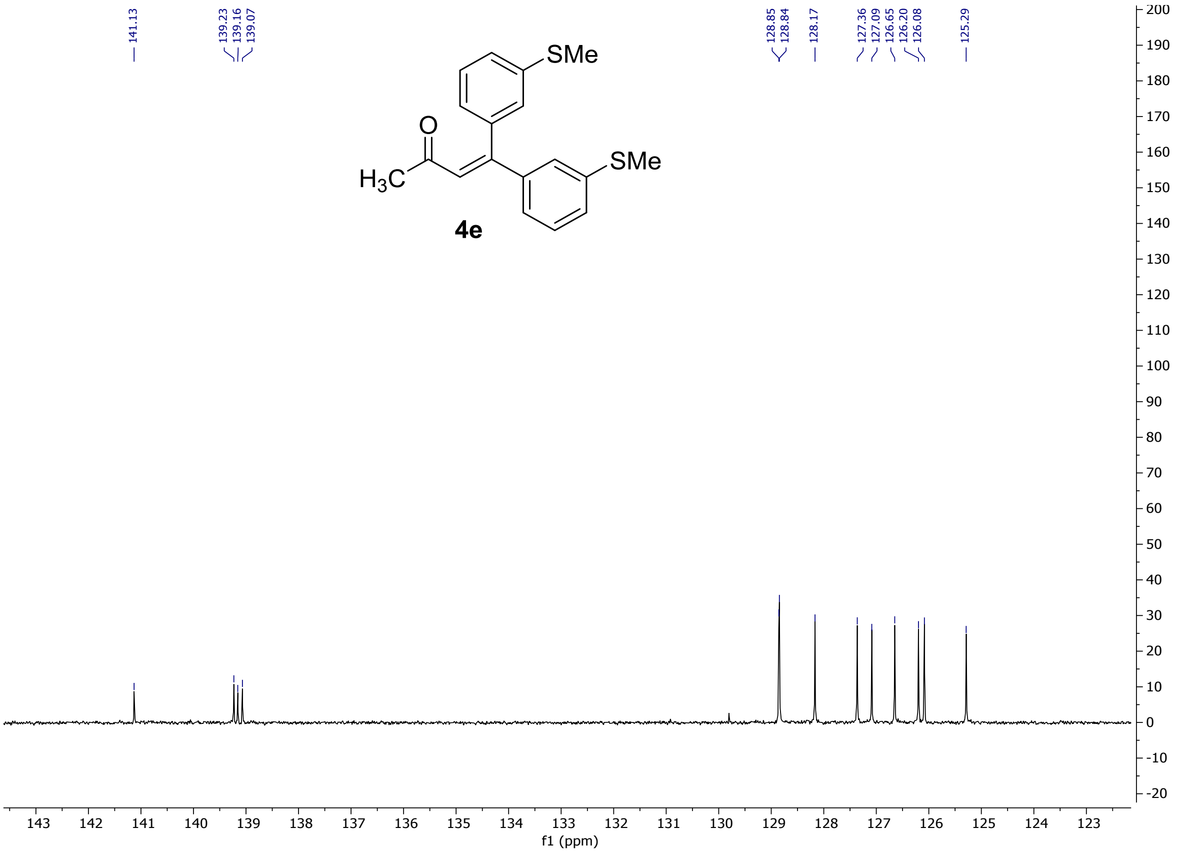

141.13
139.23
139.16
139.07
128.85
128.84
128.17
127.36
127.09
126.65
126.20
126.08
125.29
SMe
SMe
O
H_3C
4e
f1 (ppm)

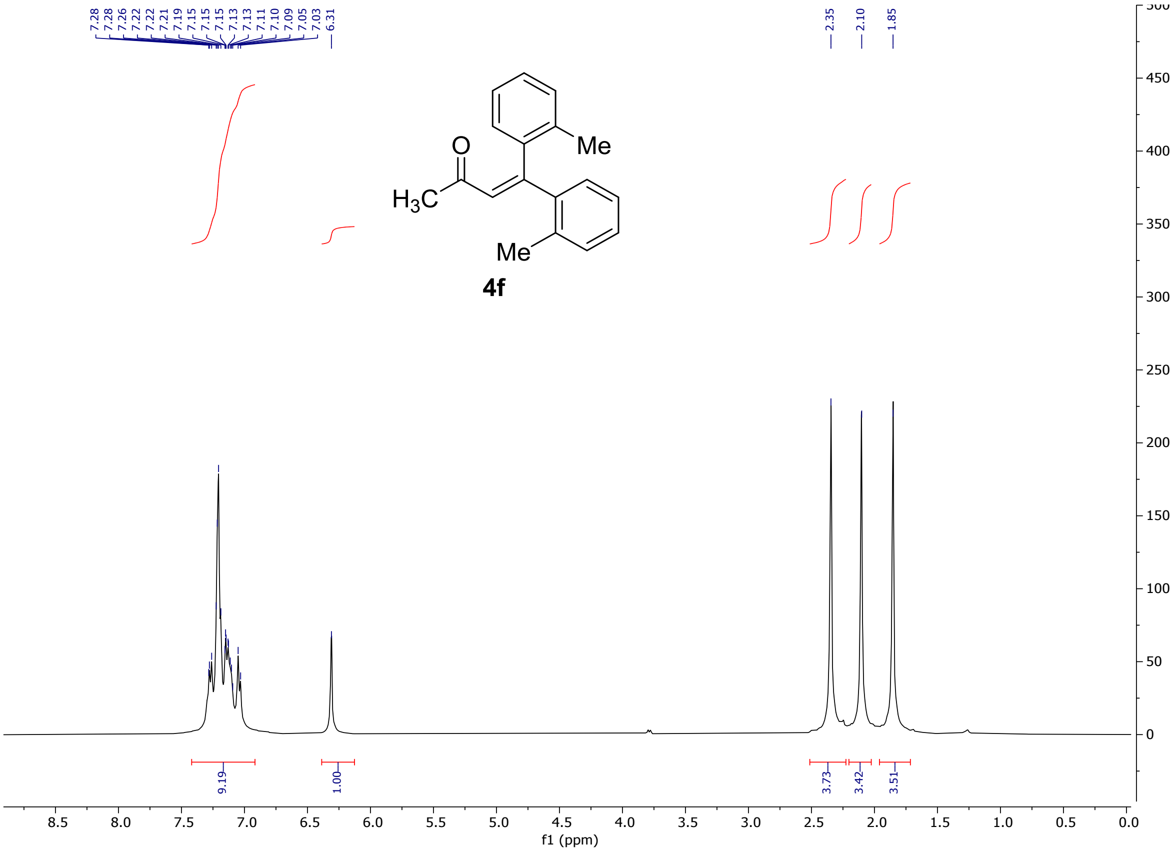

4f

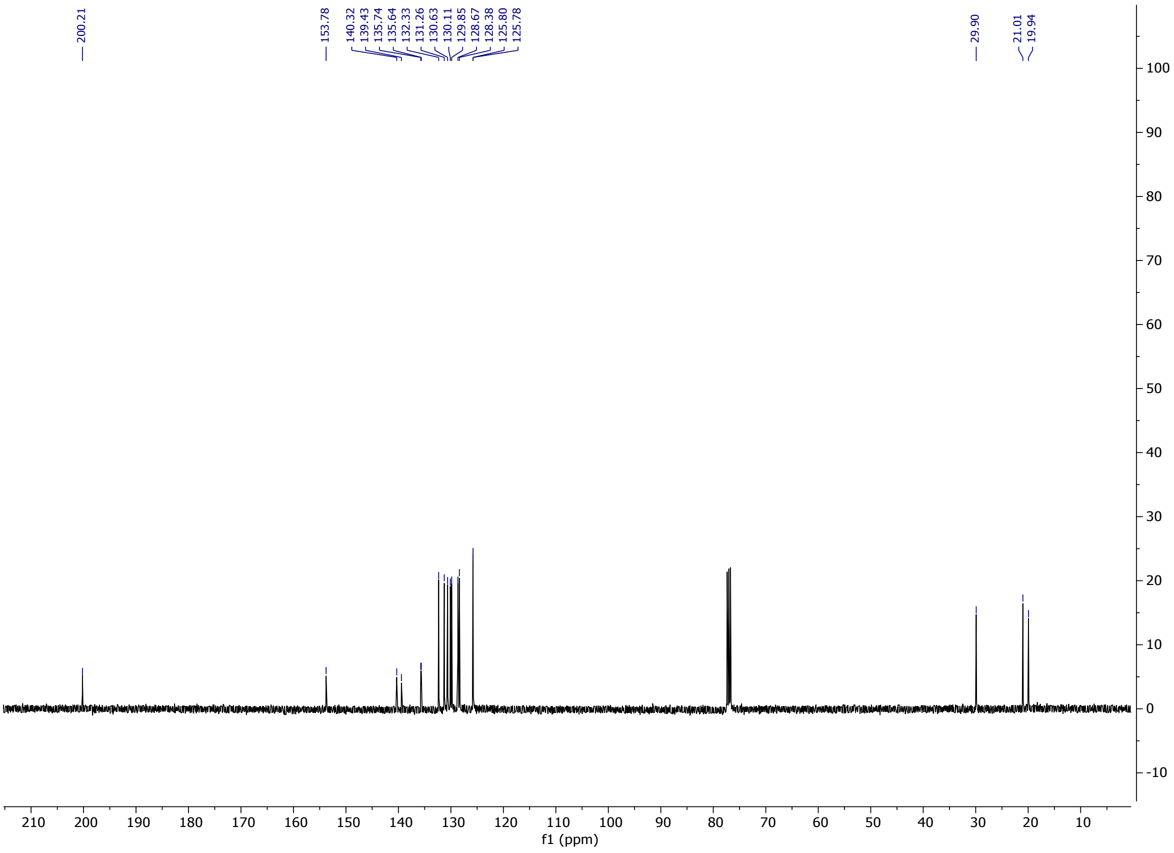

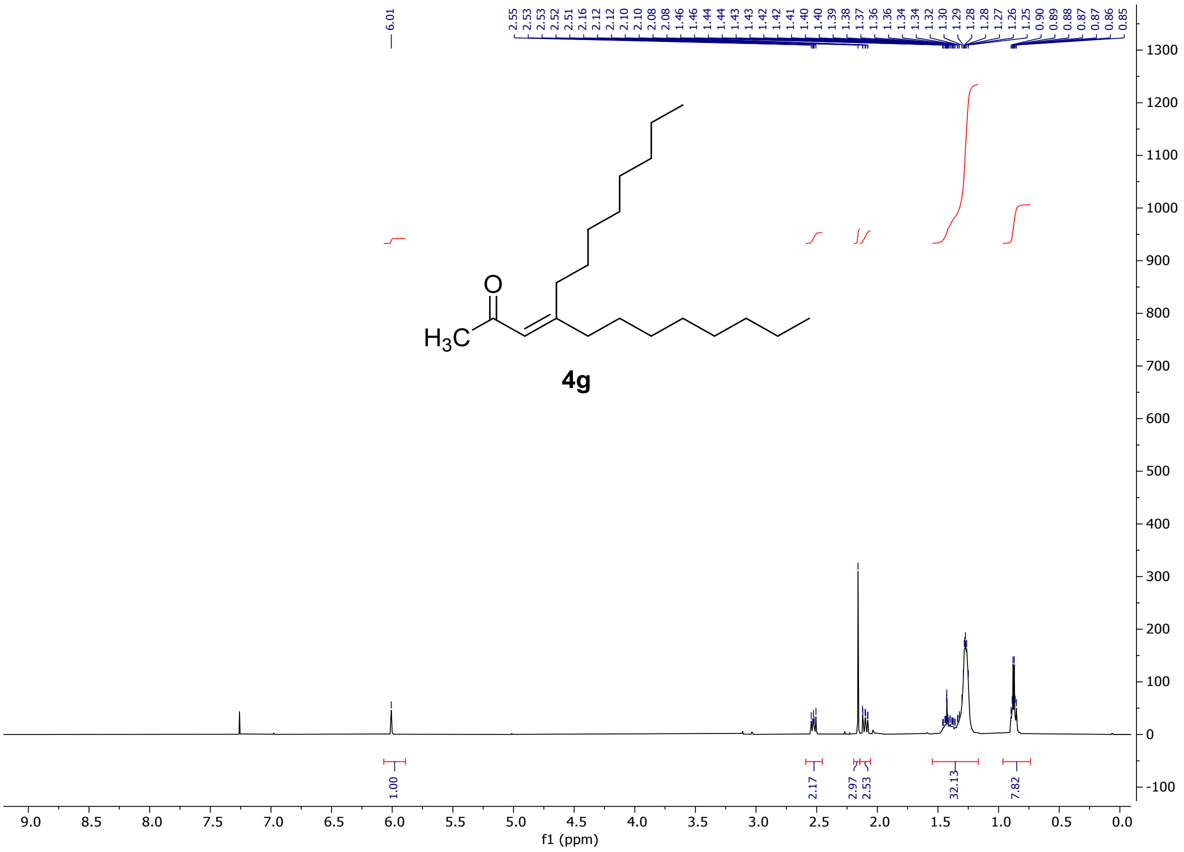

4g

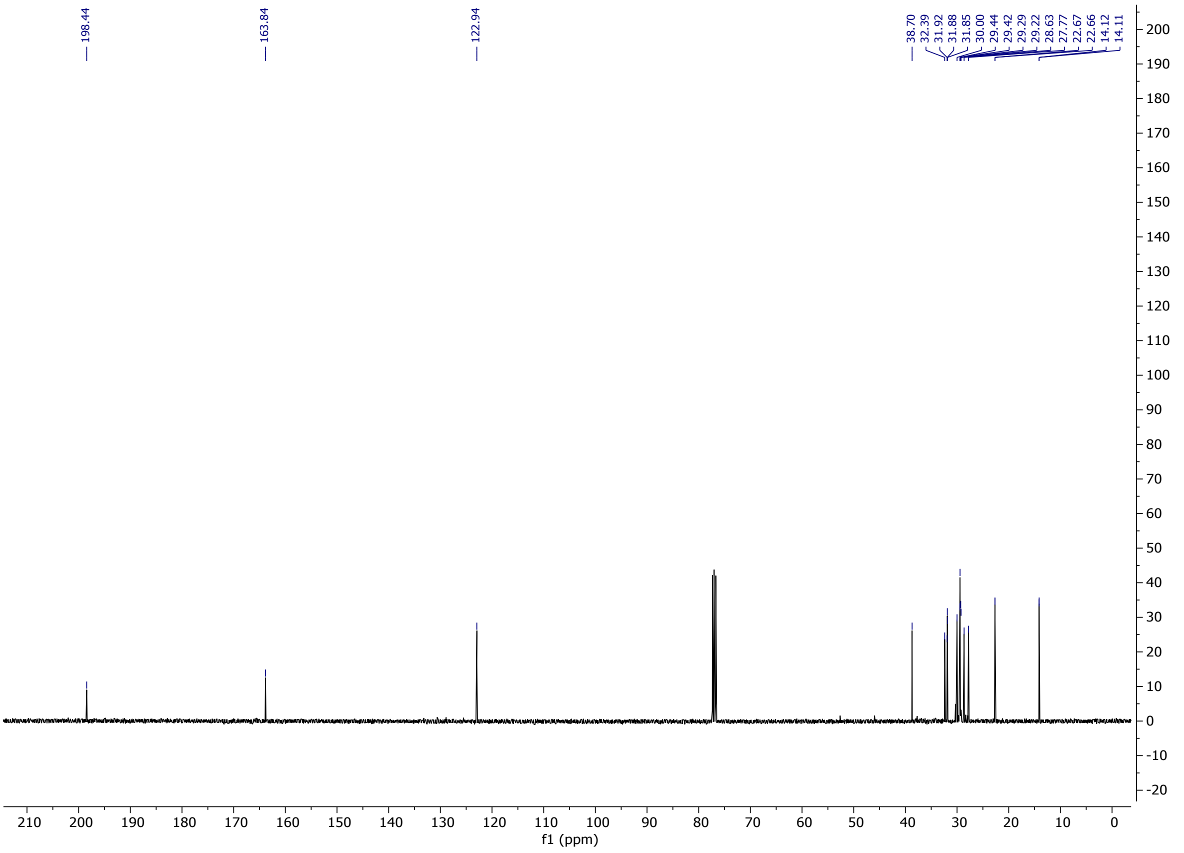

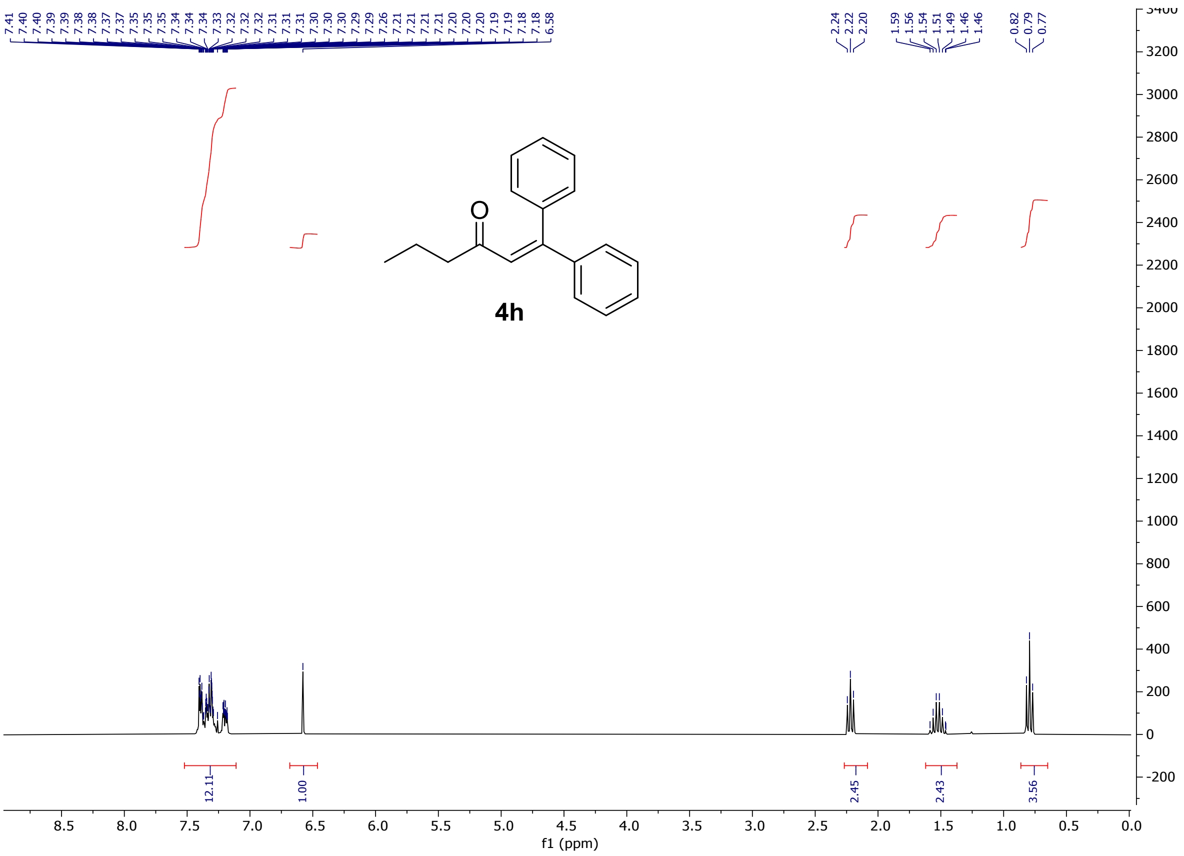

4h
f1 (ppm)

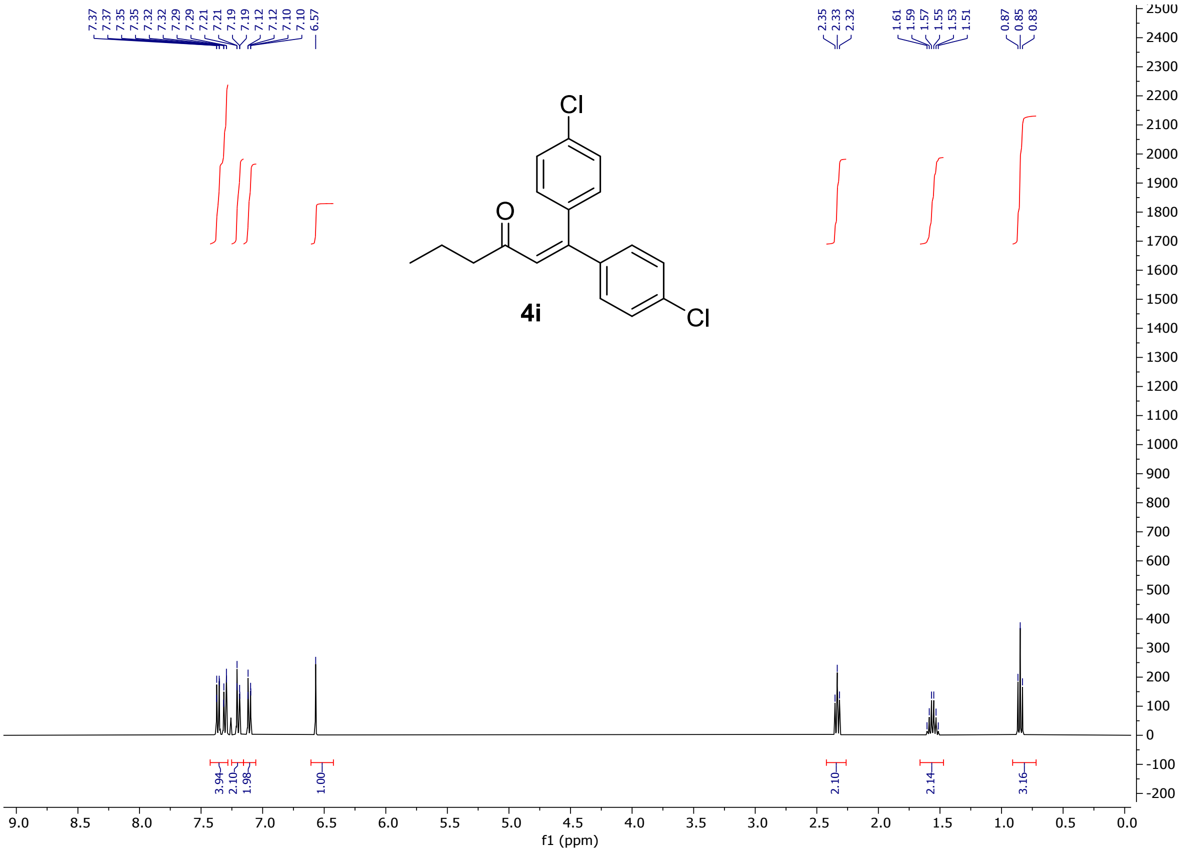
7.37
7.37
7.35
7.35
7.32
7.32
7.29
7.29
7.21
7.21
7.19
7.19
7.12
7.12
7.10
7.10
6.57
2.35
2.33
2.32
1.61
1.59
1.57
1.55
1.53
1.51
0.87
0.85
0.83
Cl
O
4i
Cl
3.94
2.10
1.98
1.00
2.10
2.14
3.16
f1 (ppm)

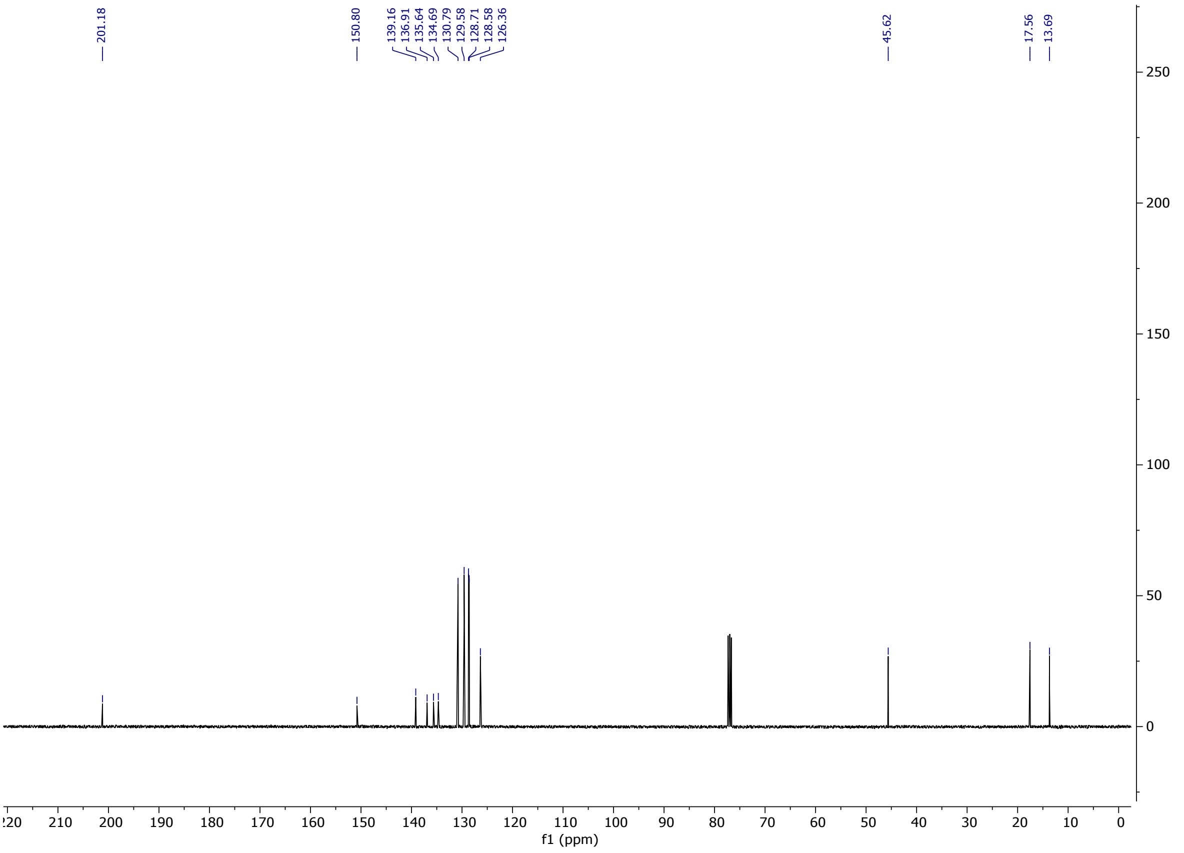
201.18
150.80
139.16
136.91
135.64
134.69
130.79
129.58
128.71
128.58
126.36
45.62
17.56
13.69
f1 (ppm)

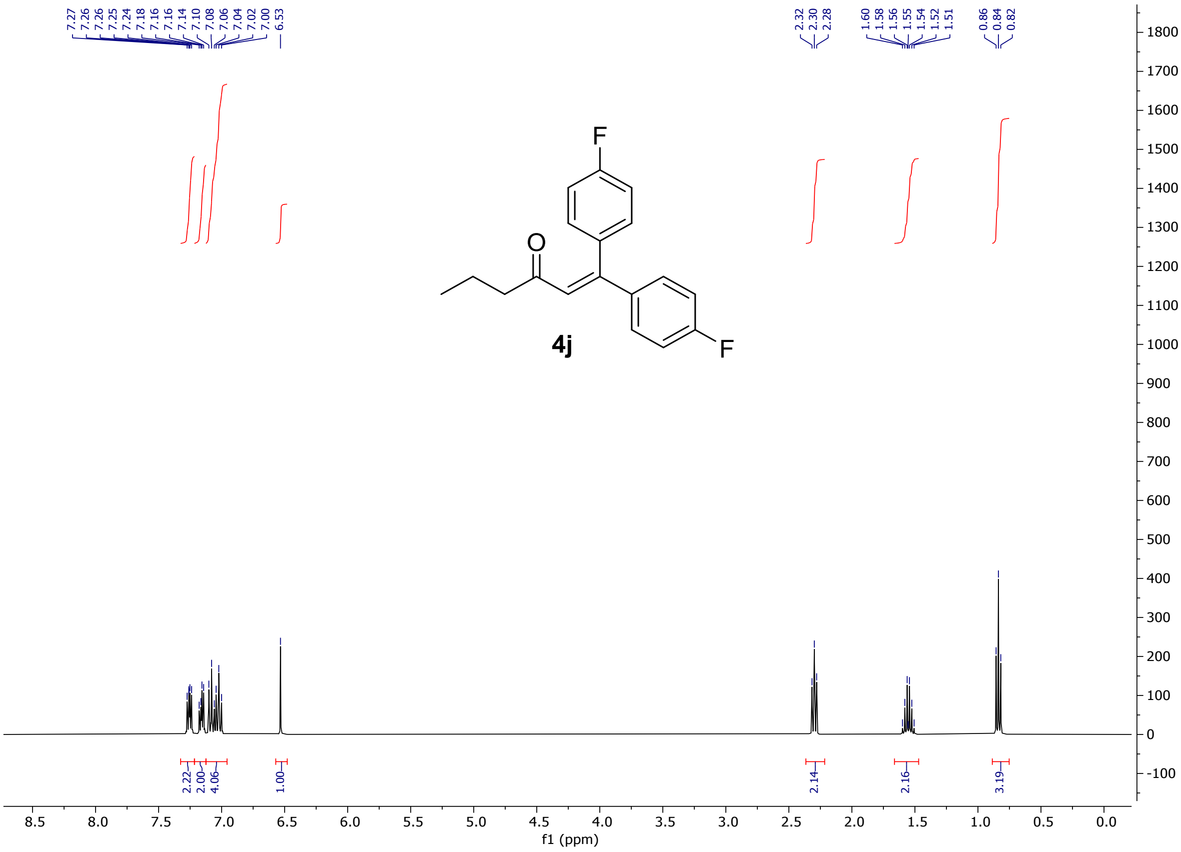

7.27
7.26
7.26
7.25
7.24
7.18
7.16
7.16
7.14
7.10
7.08
7.06
7.04
7.02
7.00
6.53
2.32
2.30
2.28
1.60
1.58
1.56
1.55
1.54
1.52
1.51
0.86
0.84
0.82
F
O
F
4j
2.22
2.00
4.06
1.00
2.14
2.16
3.19
f1 (ppm)

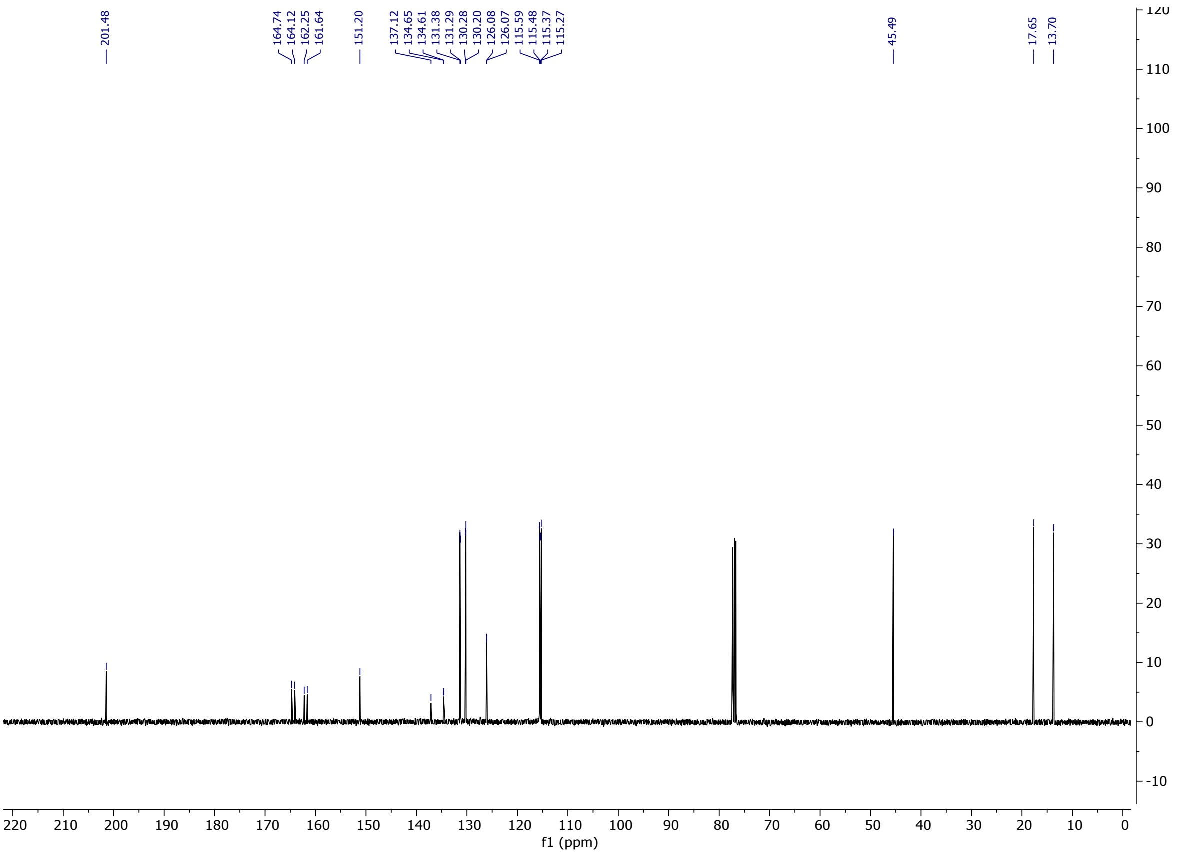

201.48
164.74
164.12
162.25
161.64
151.20
137.12
134.65
134.61
131.38
131.29
130.28
130.20
126.08
126.07
115.59
115.48
115.37
115.27
45.49
17.65
13.70
f1 (ppm)

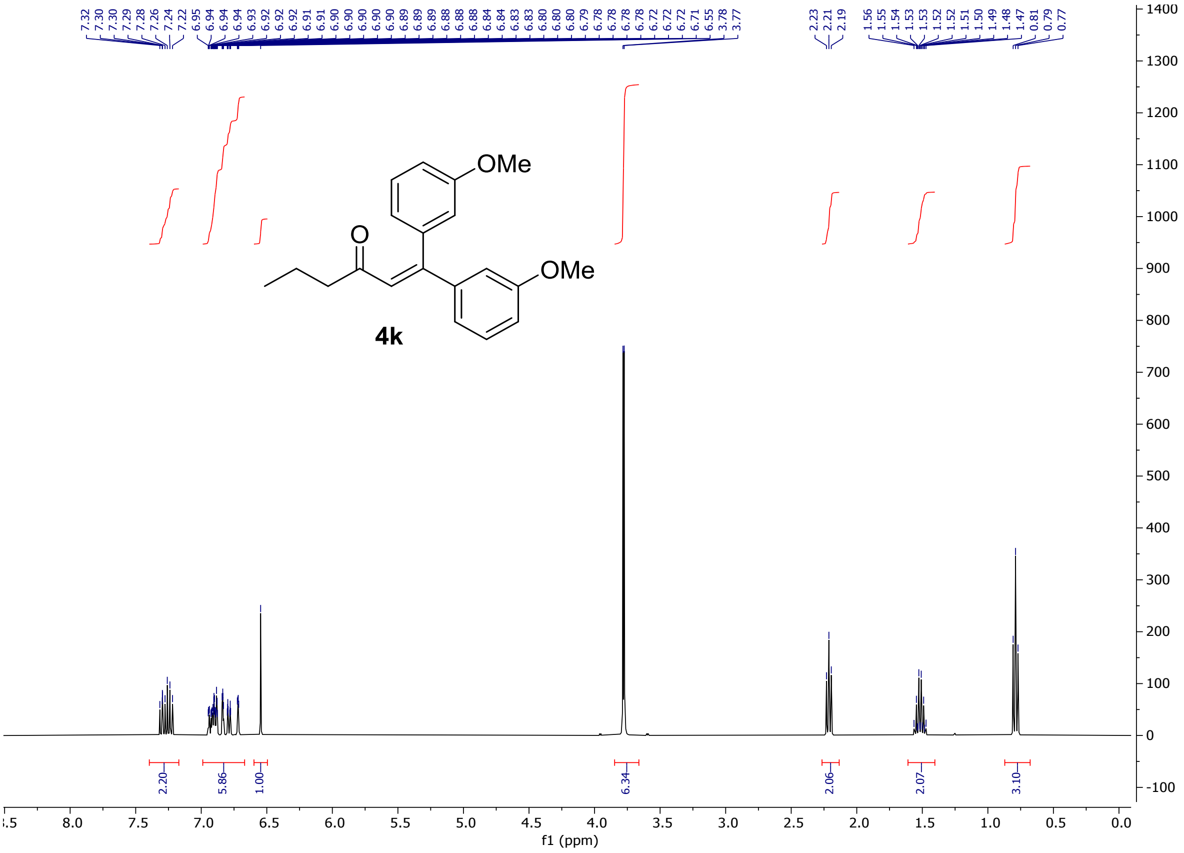
7.32 7.30 7.30 7.29 7.28 7.26 7.24 7.22 6.95 6.94 6.94 6.94 6.93 6.92 6.92 6.92 6.91 6.91 6.90 6.90 6.90 6.90 6.90 6.89 6.89 6.89 6.88 6.88 6.88 6.84 6.84 6.83 6.83 6.80 6.80 6.80 6.79 6.78 6.78 6.78 6.78 6.78 6.72 6.72 6.72 6.72 6.71 6.55 3.78 3.77
2.23 2.21 2.19
1.56 1.55 1.54 1.53 1.53 1.52 1.52 1.51 1.50 1.49 1.48 1.47 0.81 0.79 0.77
OMe
OMe
O
4k
f1 (ppm)

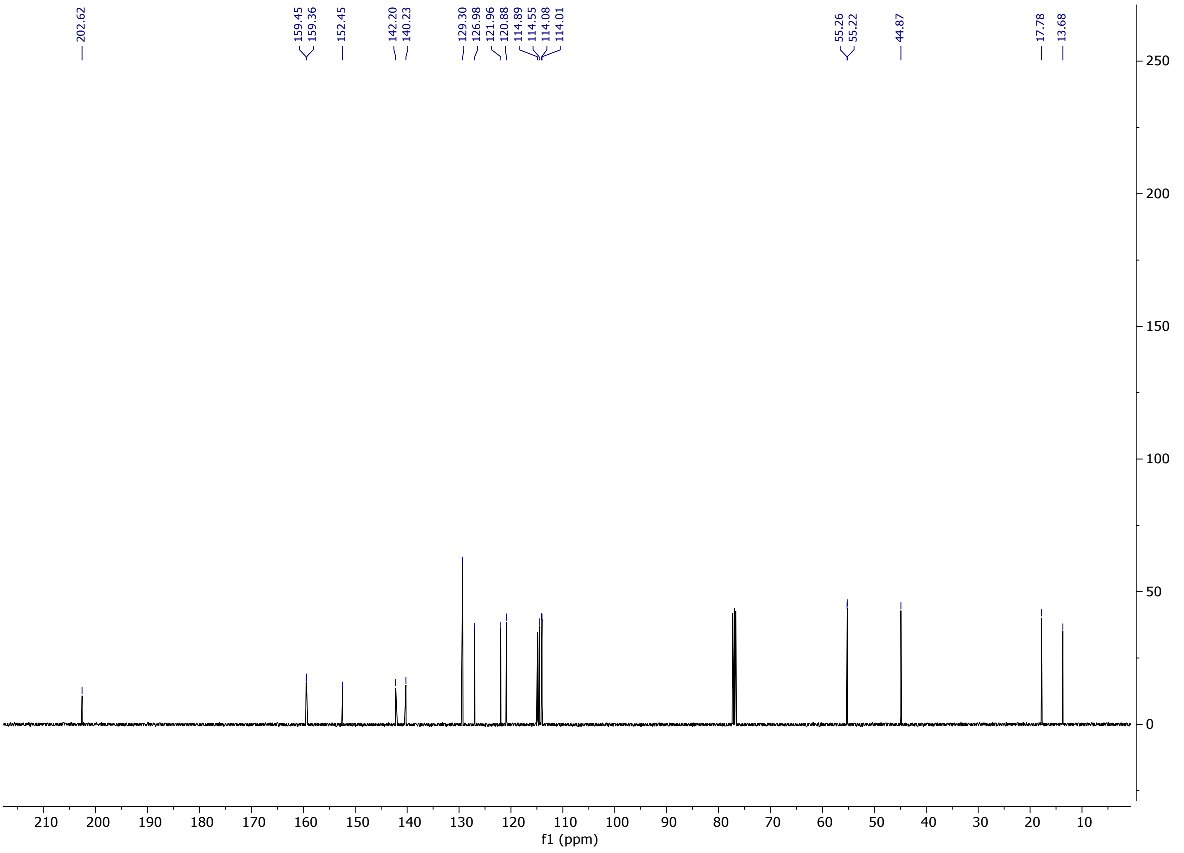
202.62
159.45 159.36 152.45 142.20 140.23 129.30 126.98 121.96 120.88 114.89 114.55 114.08 114.01
55.26 55.22 44.87 17.78 13.68
f1 (ppm)

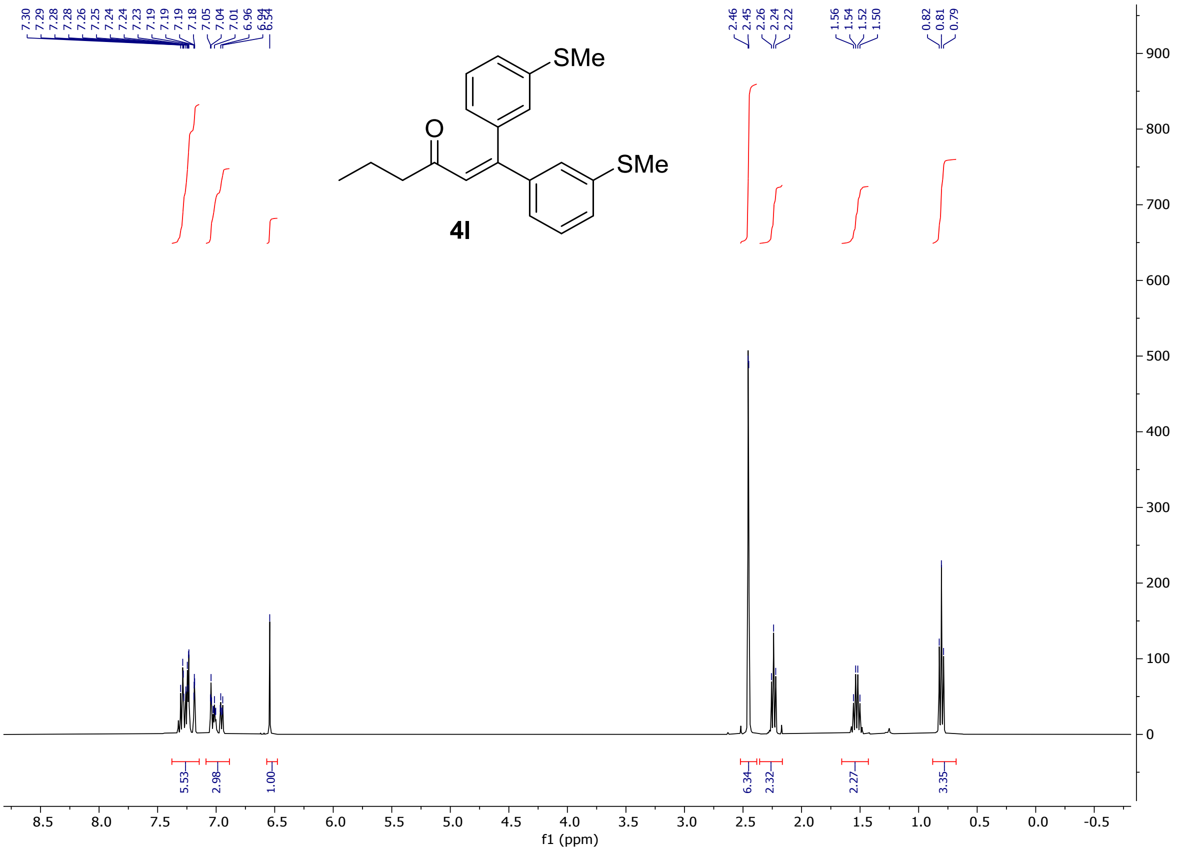

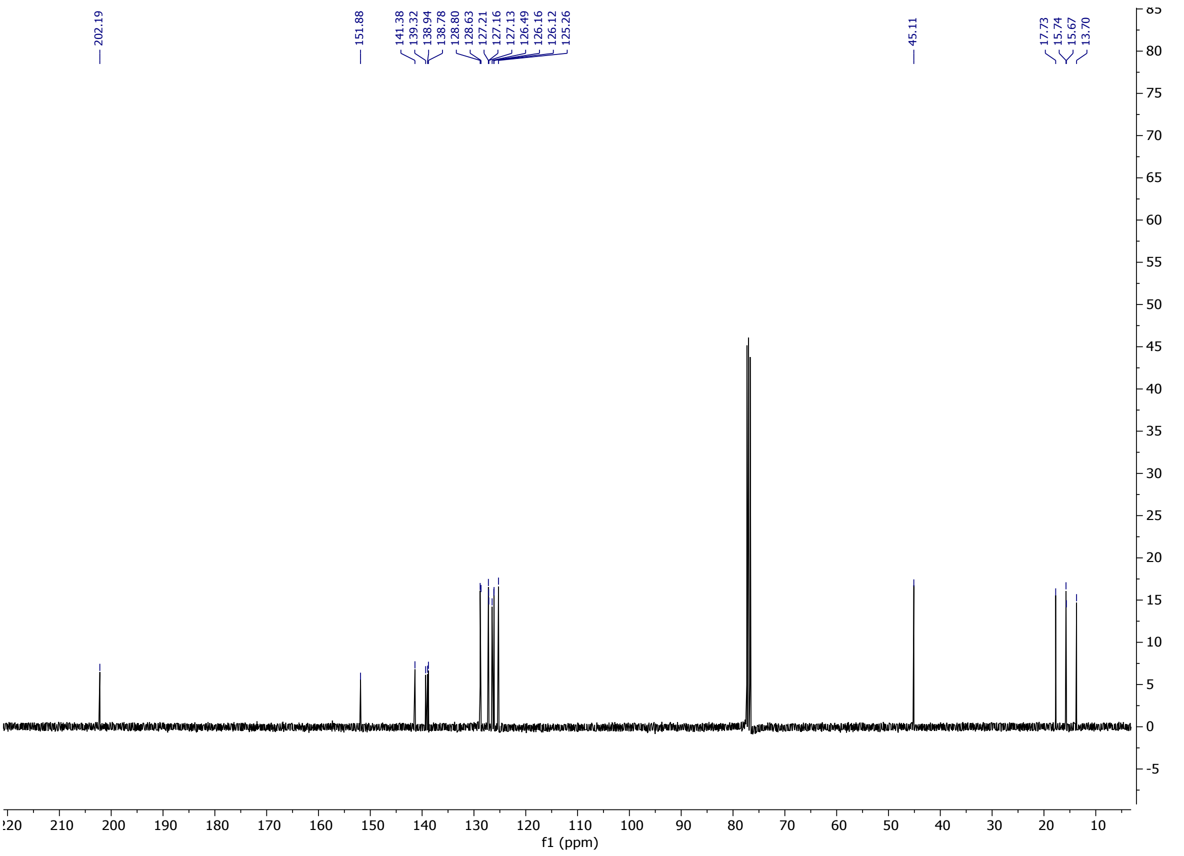

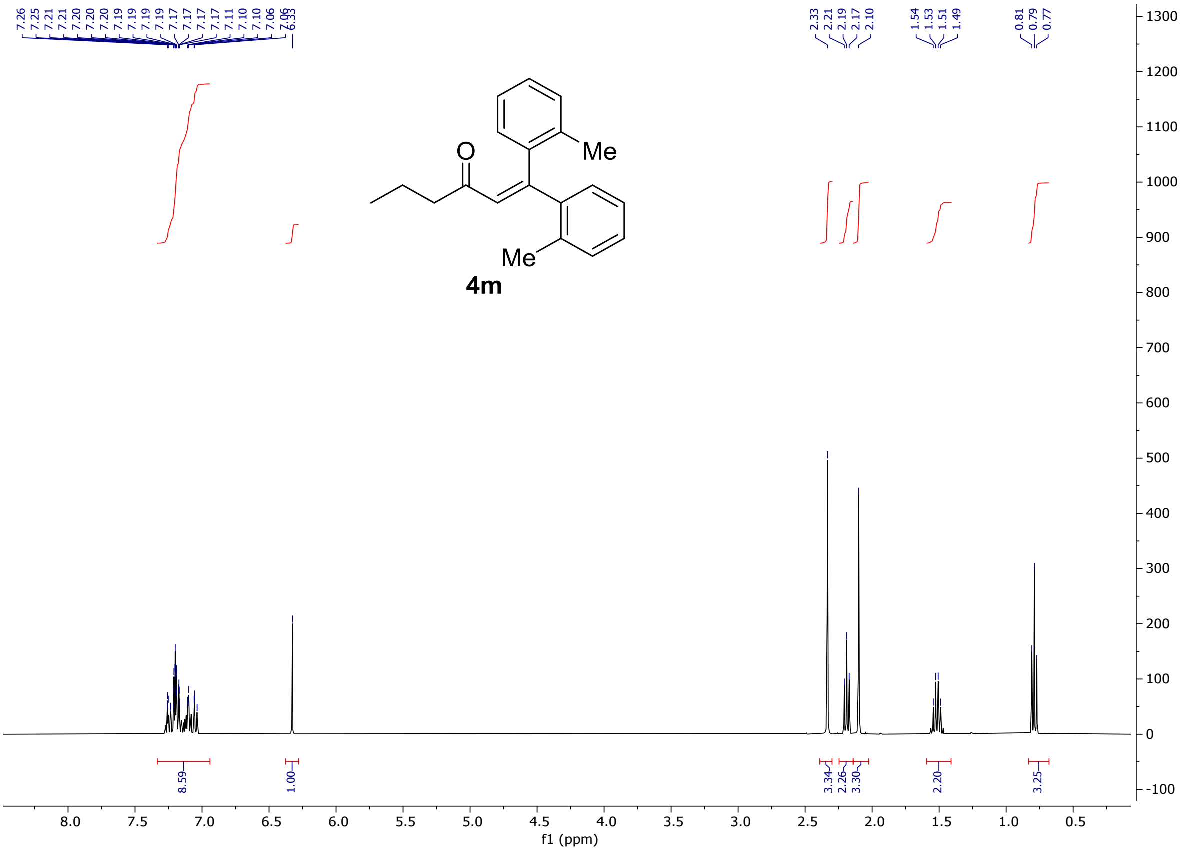
7.26
7.25
7.21
7.21
7.20
7.20
7.20
7.19
7.19
7.19
7.19
7.17
7.17
7.17
7.17
7.11
7.10
7.10
7.06
6.33
2.33
2.21
2.19
2.17
2.10
1.54
1.53
1.51
1.49
0.81
0.79
0.77
O
Me
Me
4m
8.59
1.00
3.34
2.26
3.30
2.20
3.25
f1 (ppm)

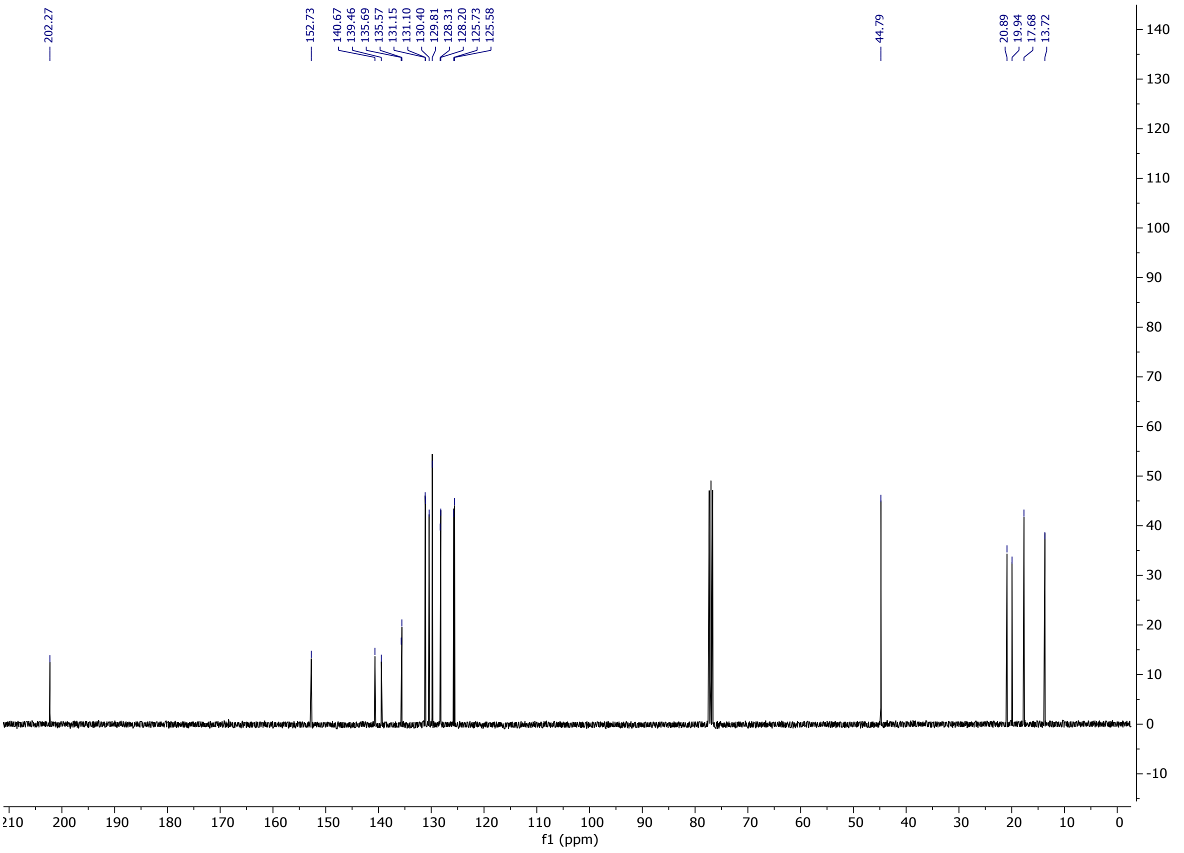
202.27
152.73
140.67
139.46
135.69
135.57
131.15
131.10
130.40
129.81
128.31
128.20
125.73
125.58
44.79
20.89
19.94
17.68
13.72
f1 (ppm)

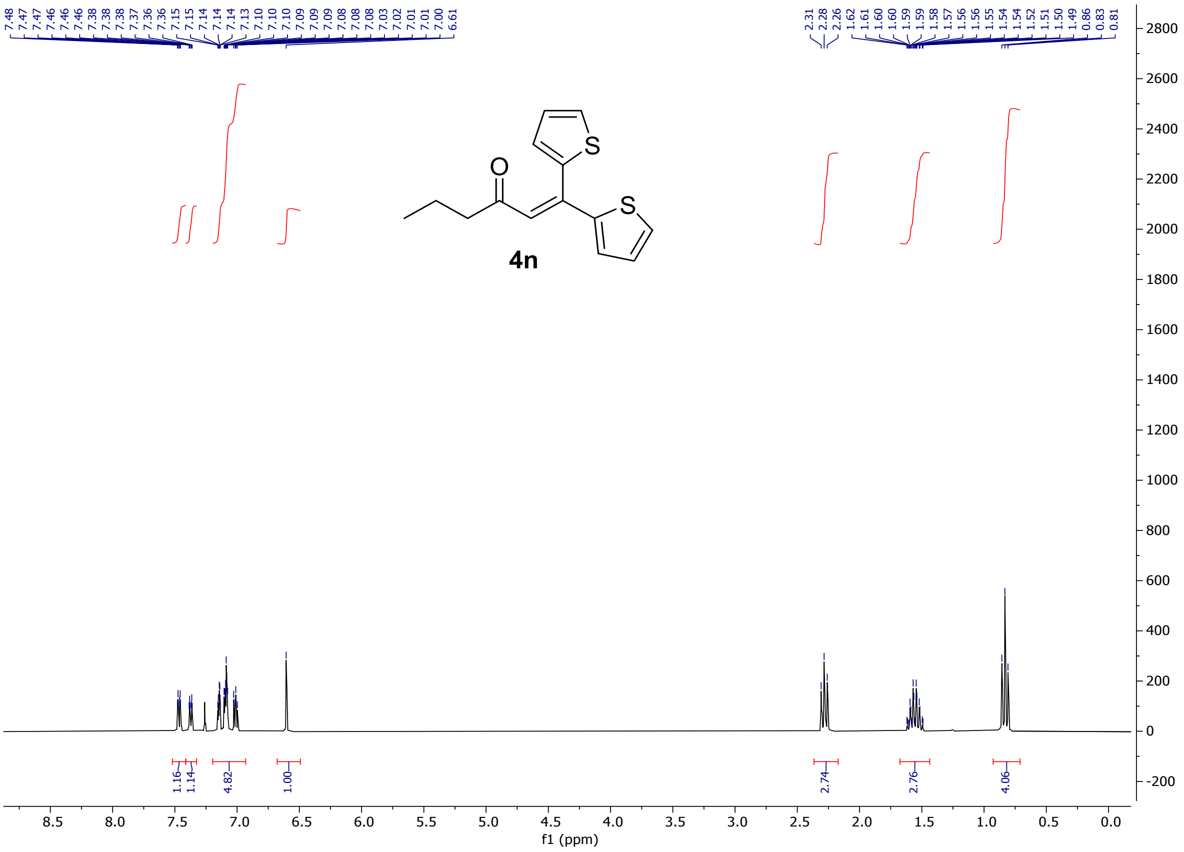
7.48 7.47 7.46 7.46 7.46 7.38 7.38 7.38 7.37 7.36 7.36 7.15 7.15 7.14 7.14 7.14 7.13 7.10 7.10 7.10 7.09 7.09 7.09 7.08 7.08 7.08 7.03 7.02 7.01 7.00 6.61
2.31 2.28 2.26 1.62 1.61 1.60 1.60 1.59 1.59 1.58 1.57 1.56 1.56 1.55 1.54 1.54 1.52 1.51 1.50 1.49 0.86 0.83 0.81
O
S
S
4n
1.16 1.14 4.82 1.00 2.74 2.76 4.06
f1 (ppm)

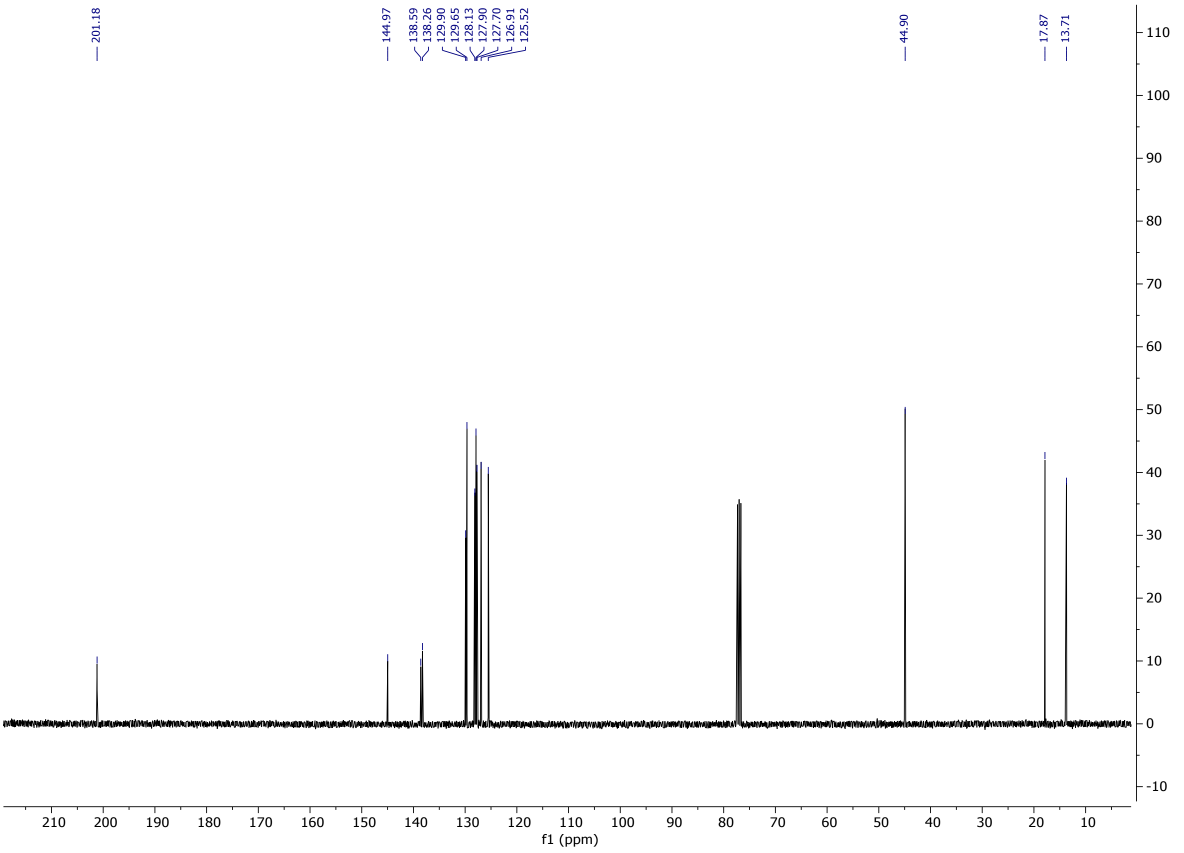
201.18
144.97 138.59 138.26 129.90 129.65 128.13 127.90 127.70 126.91 125.52
44.90
17.87 13.71
f1 (ppm)

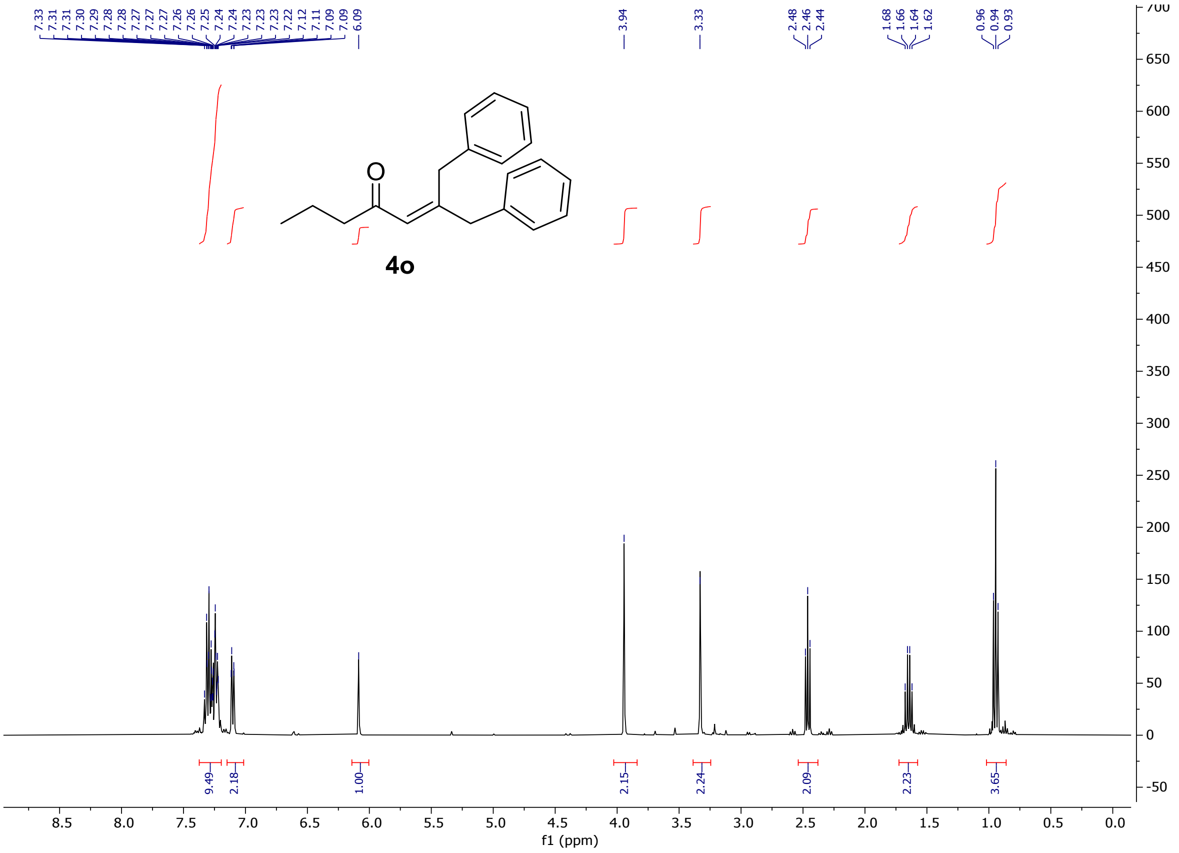
7.33
7.31
7.31
7.30
7.29
7.28
7.28
7.27
7.27
7.27
7.26
7.25
7.24
7.24
7.23
7.23
7.23
7.22
7.12
7.11
7.09
7.09
6.09
3.94
3.33
2.48
2.46
2.44
1.68
1.66
1.64
1.62
0.96
0.94
0.93
O
4o
9.49
2.18
1.00
2.15
2.24
2.09
2.23
3.65
f1 (ppm)

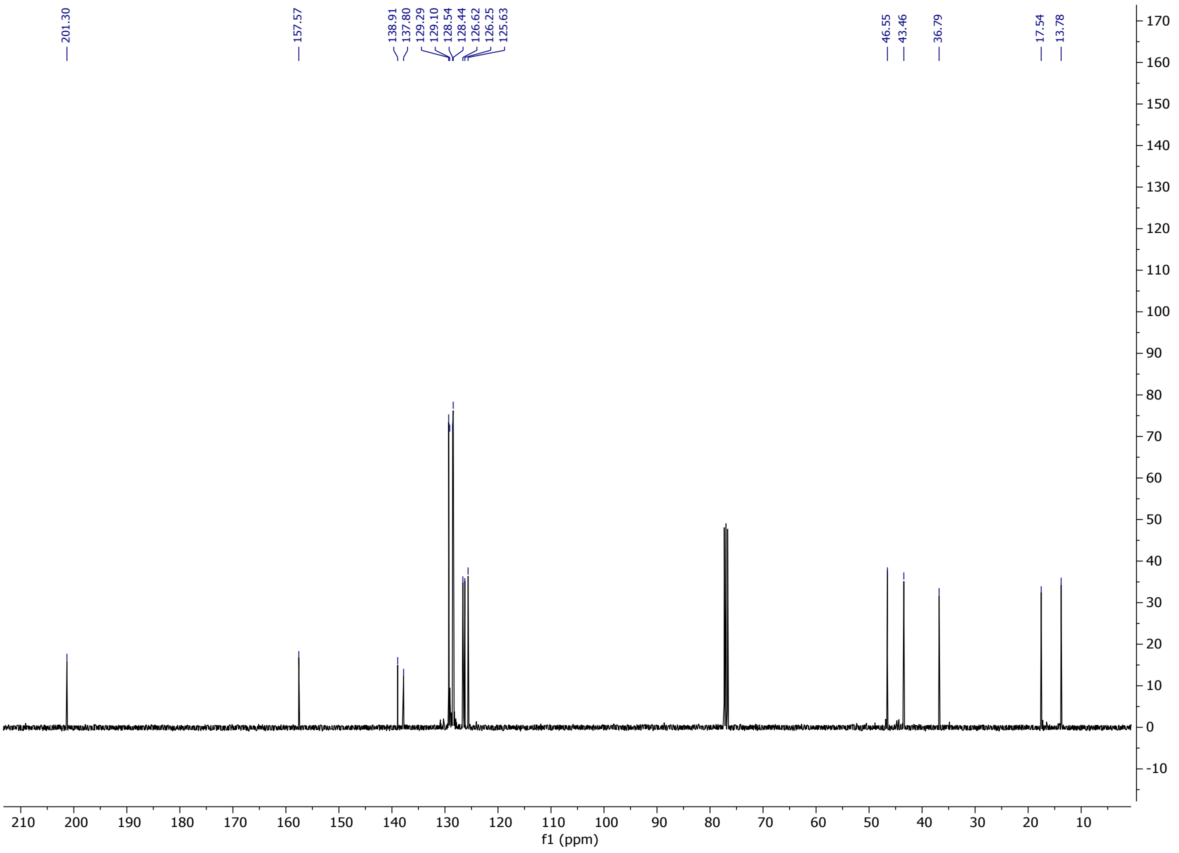
201.30
157.57
138.91
137.80
129.29
129.10
128.54
128.44
126.62
126.25
125.63
46.55
43.46
36.79
17.54
13.78
f1 (ppm)

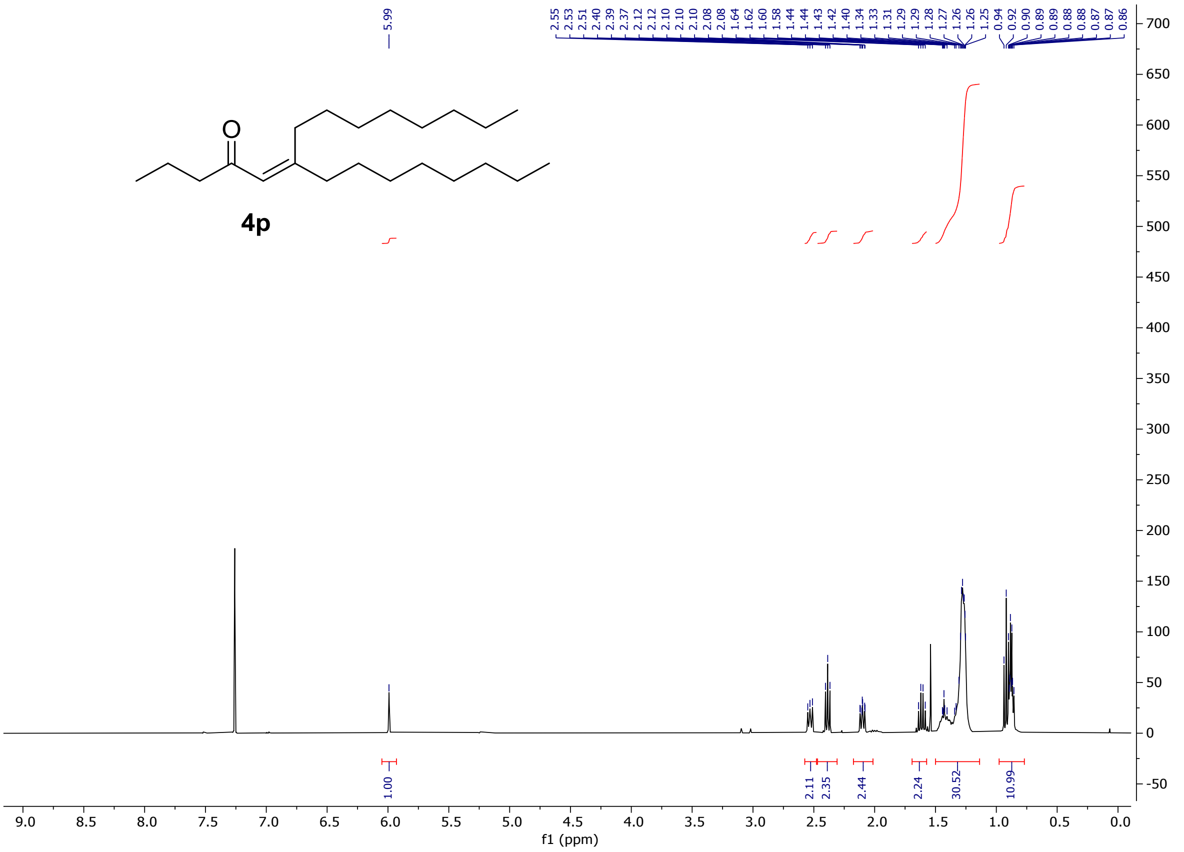

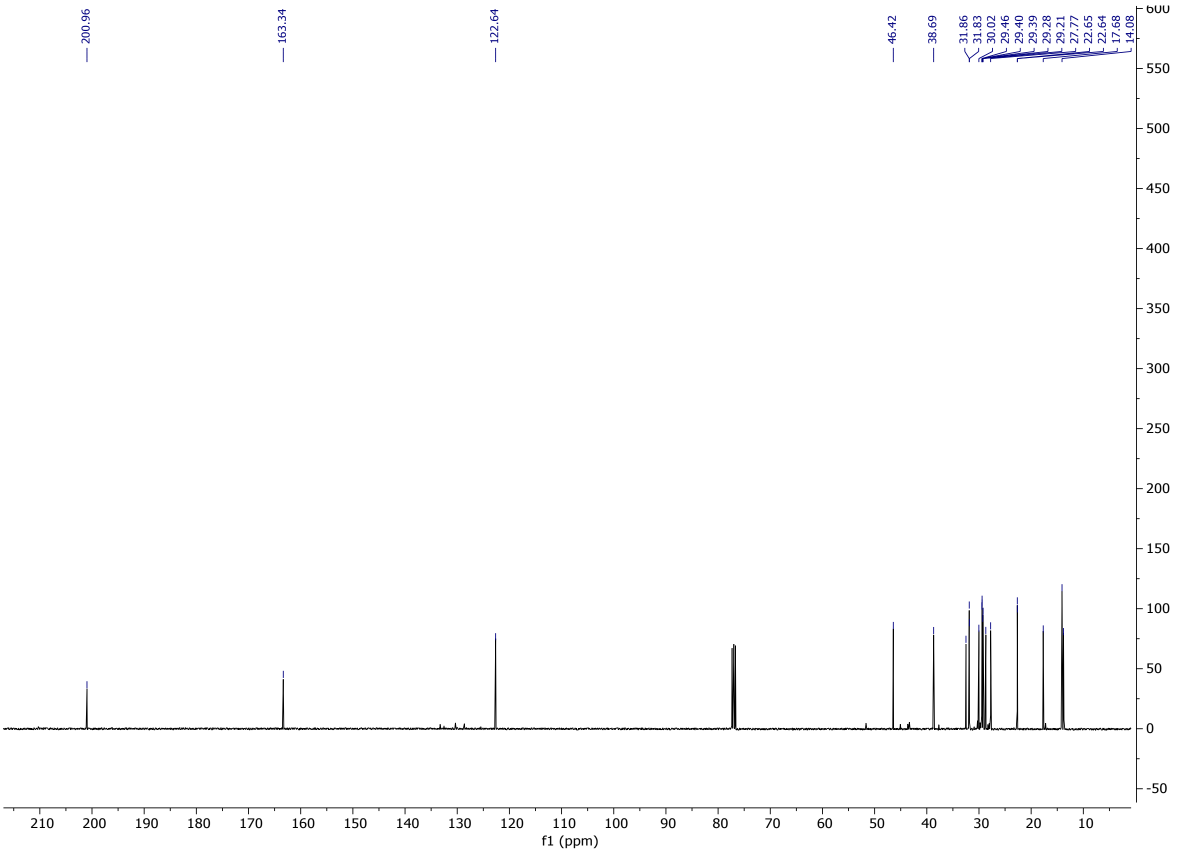

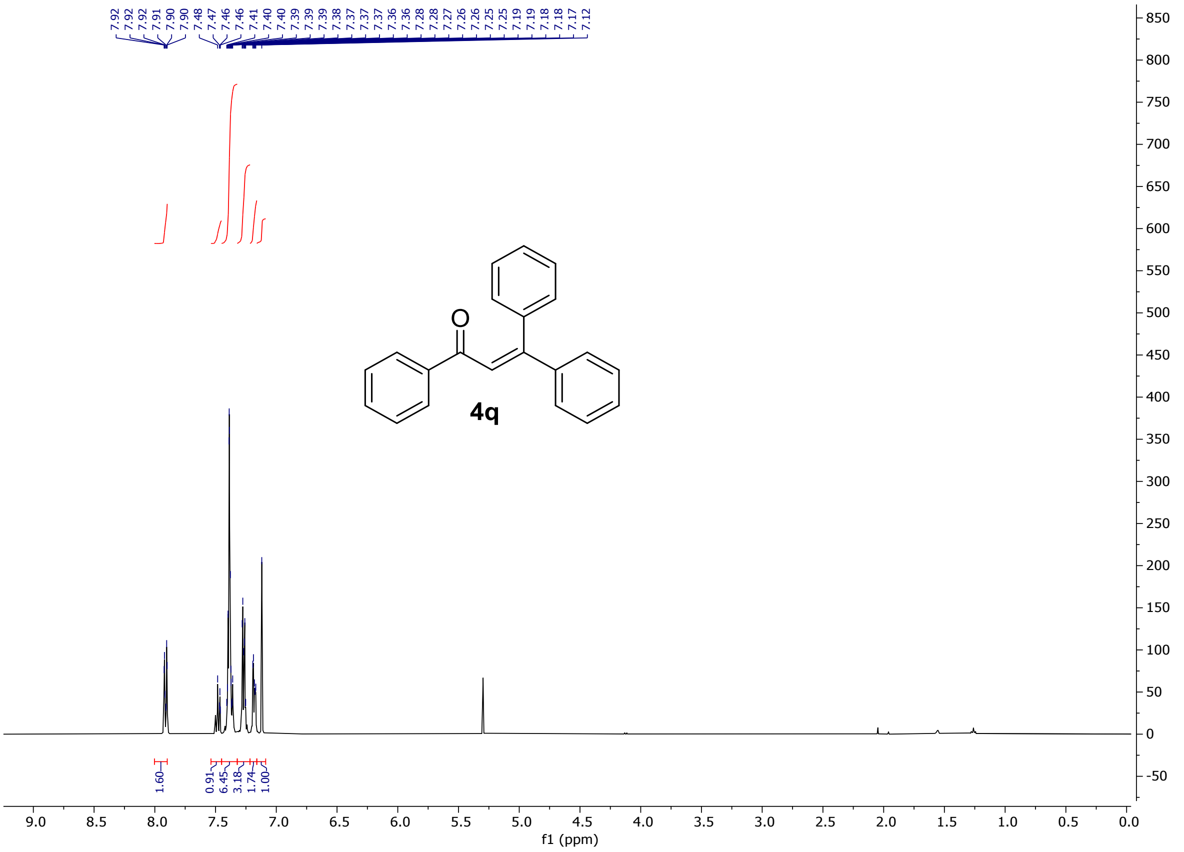
7.92 7.92 7.92 7.91 7.90 7.90 7.48 7.47 7.46 7.46 7.41 7.40 7.40 7.39 7.39 7.39 7.38 7.37 7.37 7.37 7.36 7.36 7.28 7.28 7.28 7.27 7.26 7.26 7.25 7.25 7.19 7.19 7.18 7.18 7.17 7.12
O
4q
1.60
0.91
6.45
3.18
1.74
1.00
f1 (ppm)

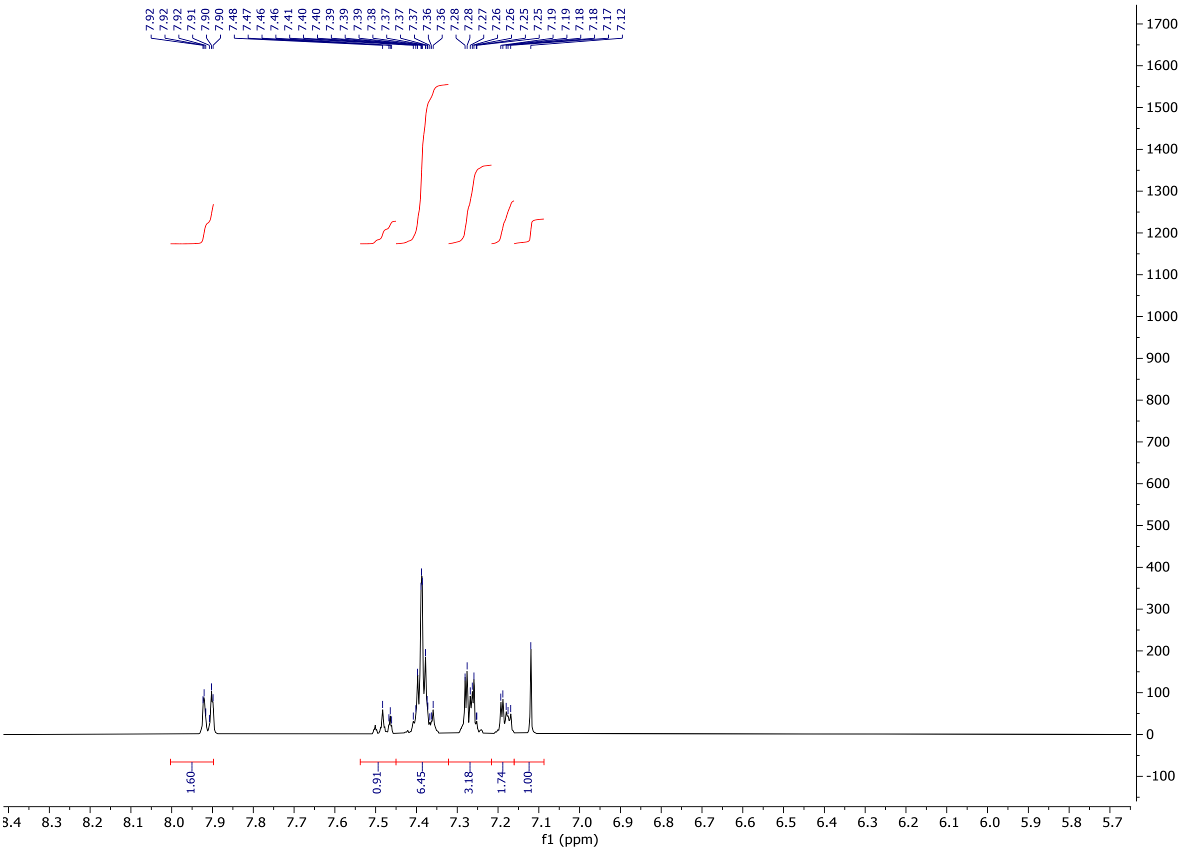
7.92 7.92 7.92 7.91 7.90 7.90 7.48 7.47 7.46 7.41 7.40 7.40 7.39 7.39 7.39 7.38 7.37 7.37 7.37 7.36 7.36 7.28 7.28 7.28 7.27 7.26 7.26 7.25 7.25 7.19 7.19 7.18 7.18 7.17 7.12
1.60
0.91
6.45
3.18
1.74
1.00
f1 (ppm)

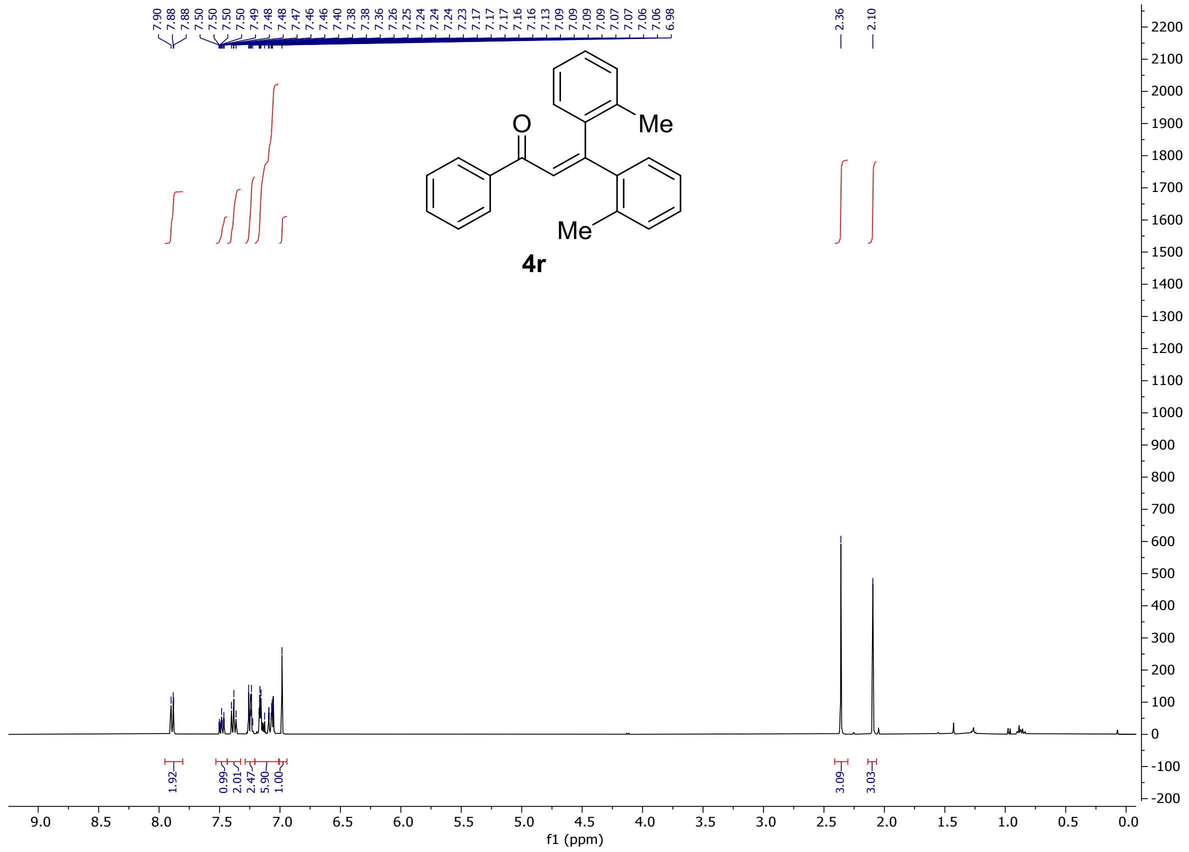
Me
Me
O
4r

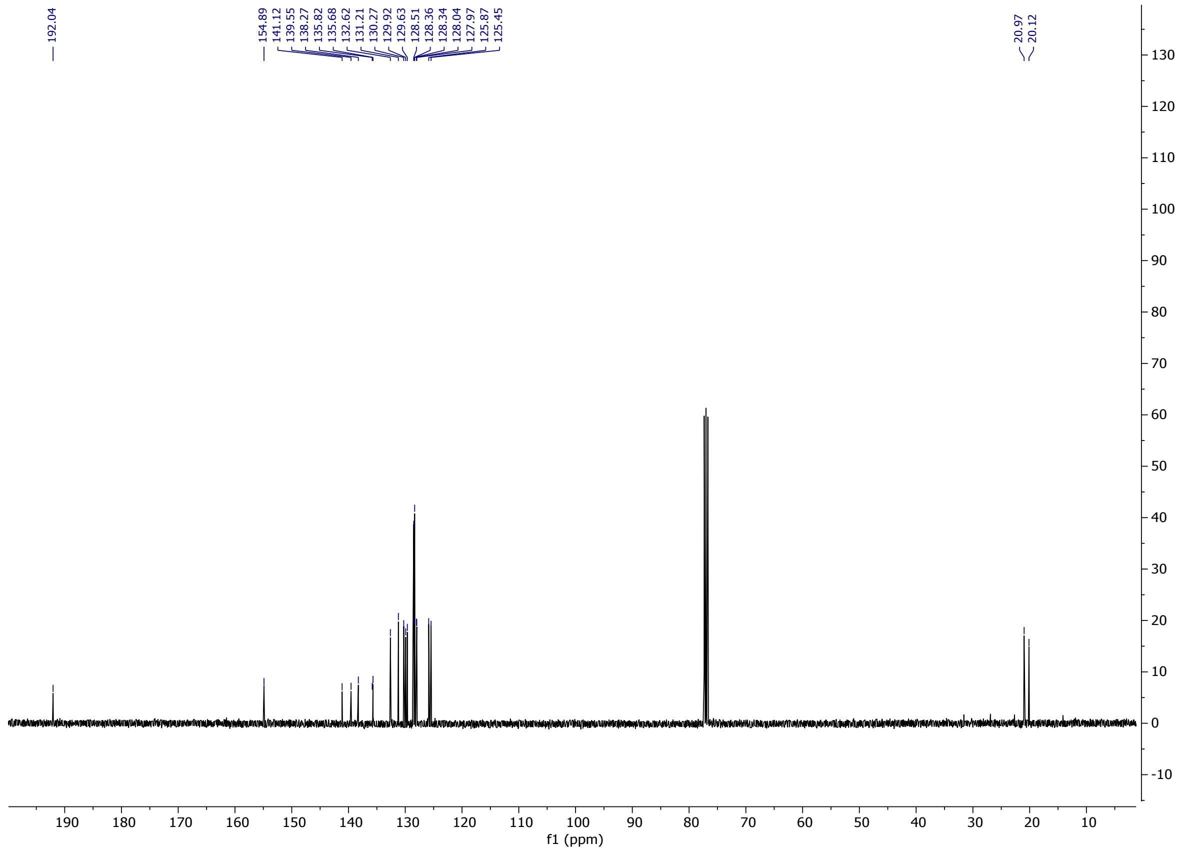

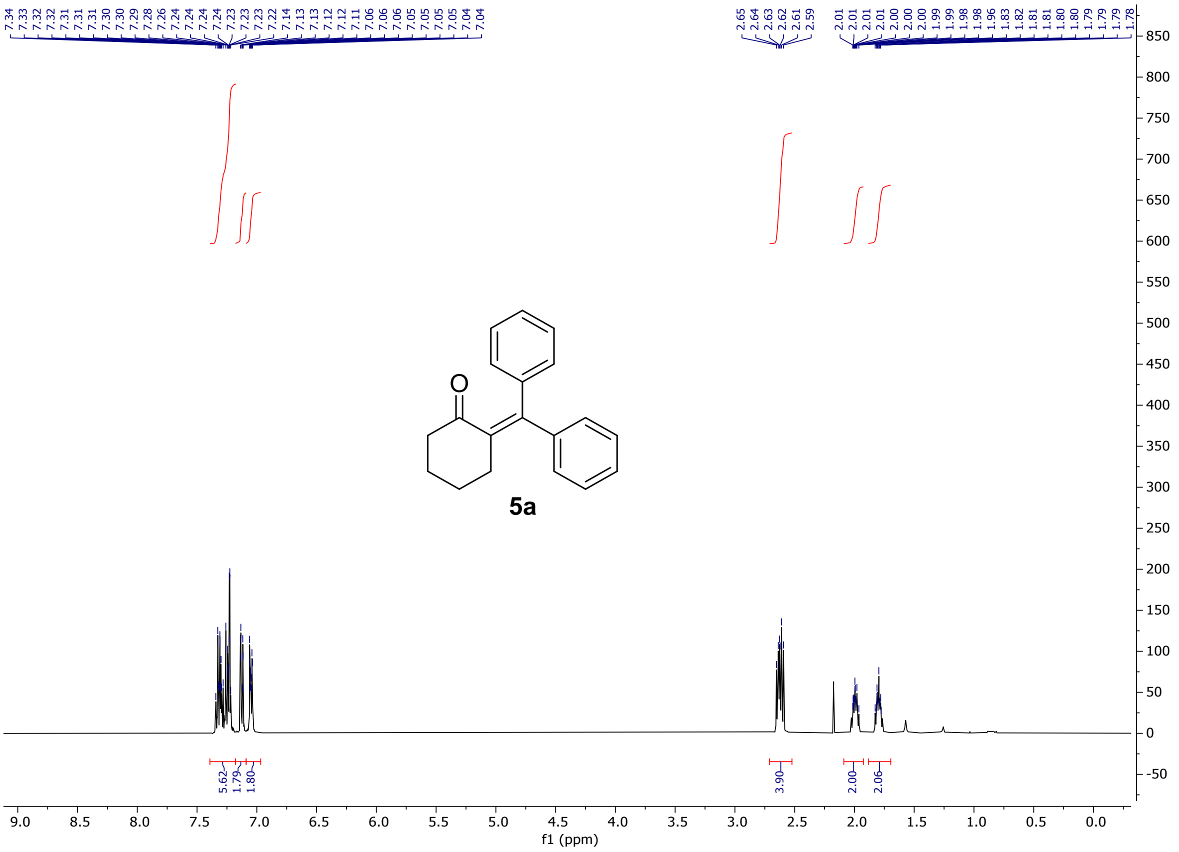
7.34 7.33 7.32 7.32 7.31 7.31 7.31 7.30 7.30 7.29 7.28 7.26 7.24 7.24 7.24 7.24 7.24 7.23 7.23 7.23 7.22 7.14 7.13 7.13 7.12 7.12 7.11 7.06 7.06 7.06 7.05 7.05 7.05 7.05 7.04 7.04
2.65 2.64 2.63 2.62 2.61 2.59
2.01 2.01 2.01 2.01 2.00 2.00 1.99 1.99 1.98 1.98 1.96 1.83 1.82 1.81 1.81 1.80 1.80 1.79 1.79 1.78
O
5a
5.62 1.79 1.80
3.90
2.00 2.06
f1 (ppm)

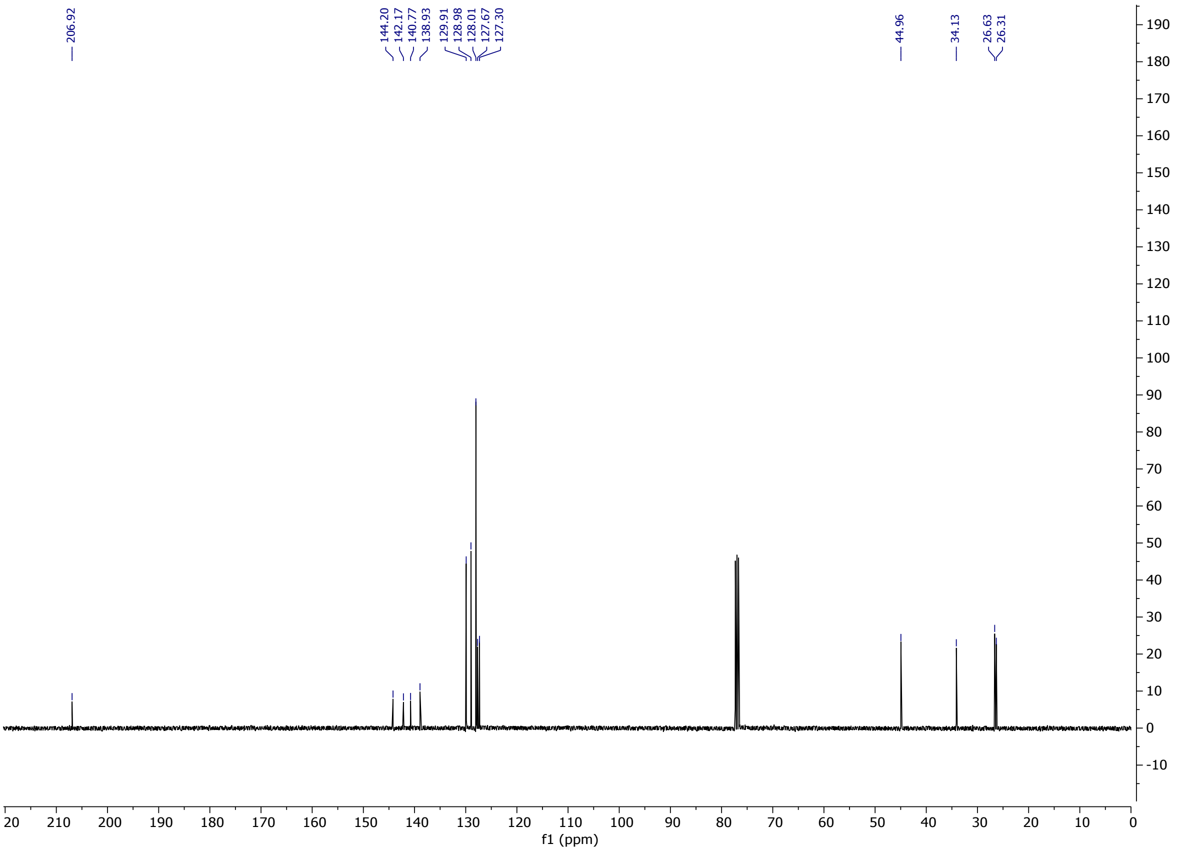
206.92
144.20 142.17 140.77 138.93 129.91 128.98 128.01 127.67 127.30
44.96
34.13
26.63 26.31
f1 (ppm)

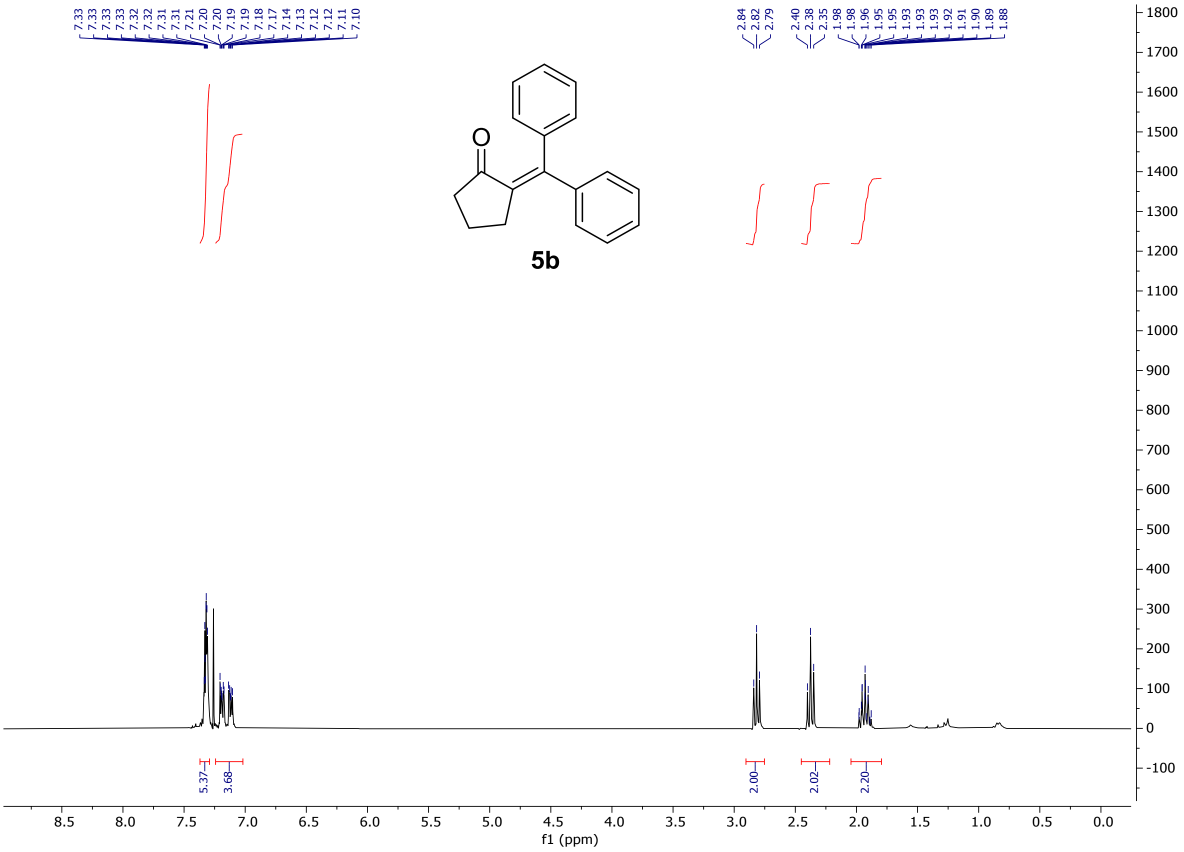
7.33
7.33
7.33
7.33
7.32
7.32
7.31
7.31
7.21
7.20
7.20
7.19
7.19
7.18
7.17
7.14
7.13
7.12
7.12
7.11
7.10
2.84
2.82
2.79
2.40
2.38
2.35
1.98
1.98
1.96
1.95
1.95
1.93
1.93
1.92
1.91
1.90
1.89
1.88
O
5b
5.37
3.68
2.00
2.02
2.20
f1 (ppm)

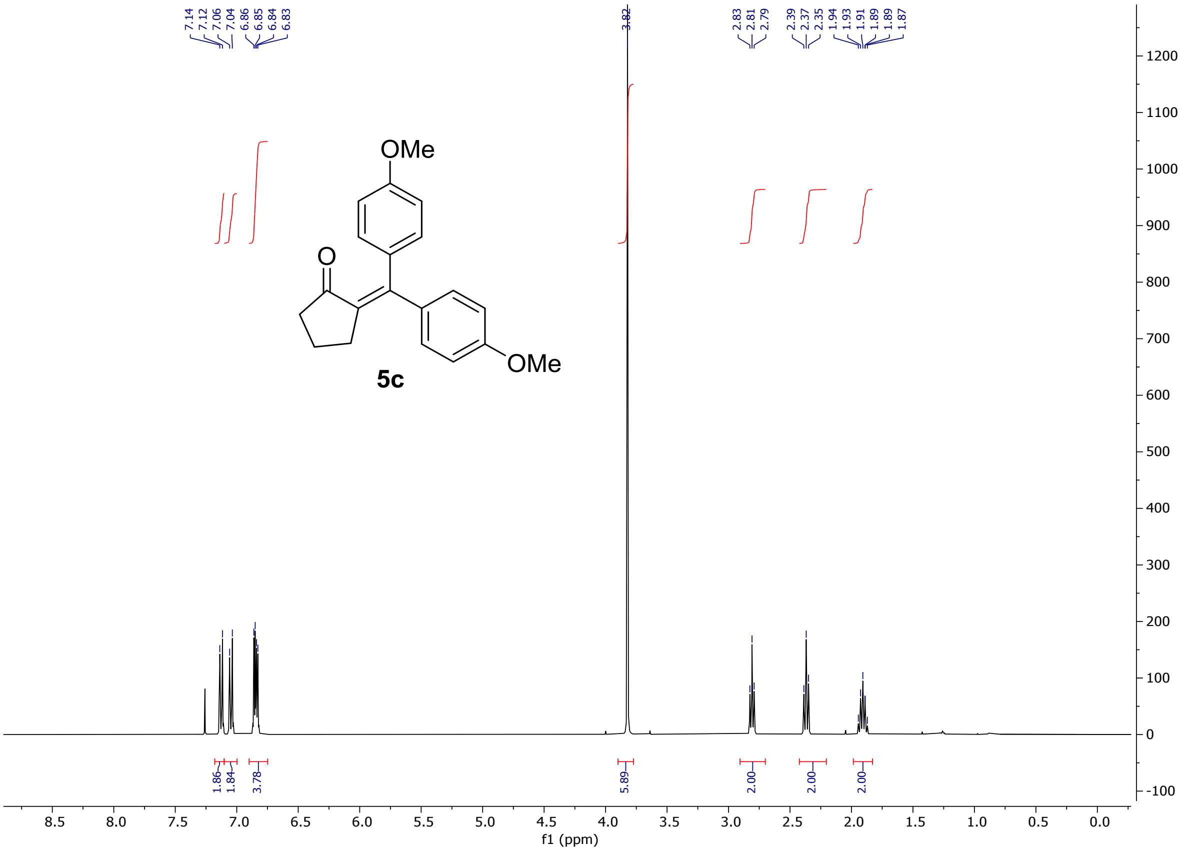
7.14
7.12
7.06
7.04
6.86
6.85
6.84
6.83
3.82
2.83
2.81
2.79
2.39
2.37
2.35
1.94
1.93
1.91
1.89
1.89
1.87
OMe
O
OMe
5c
1.86
1.84
3.78
5.89
2.00
2.00
2.00
f1 (ppm)

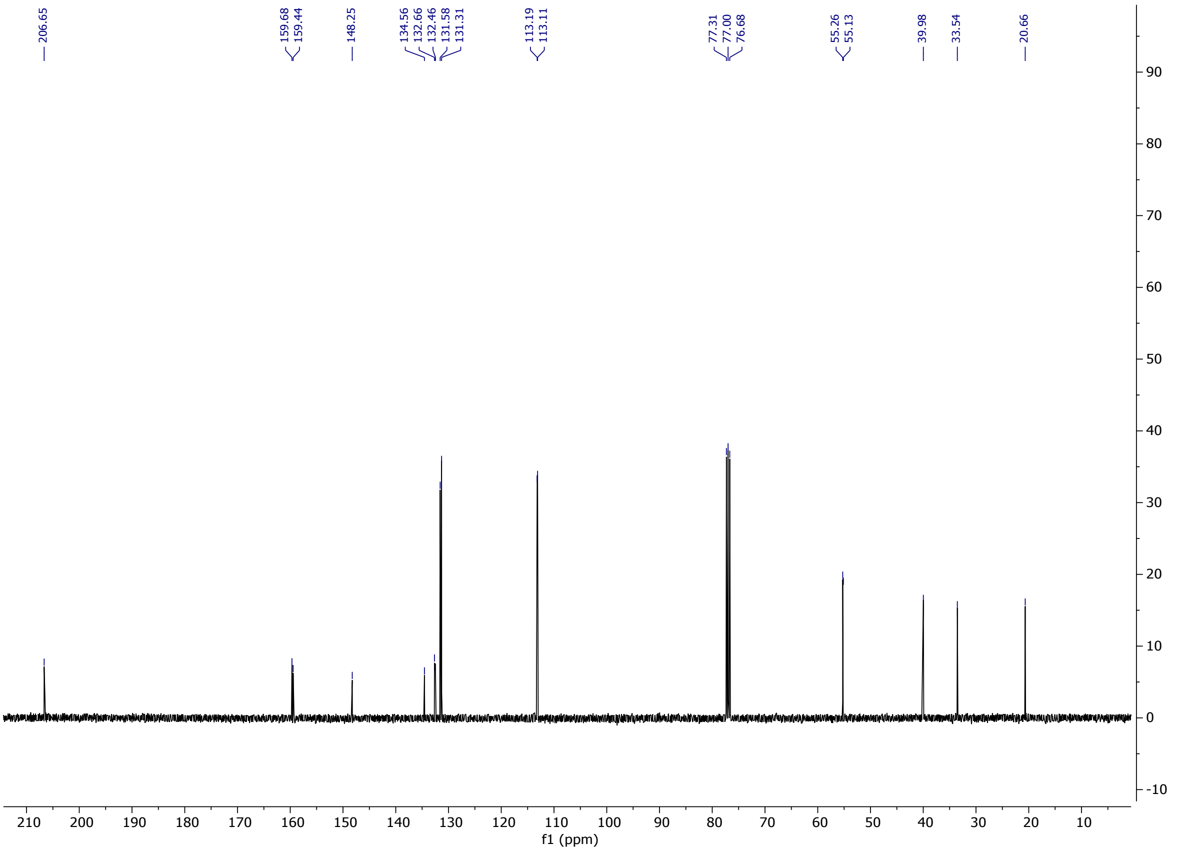
206.65
159.68
159.44
148.25
134.56
132.66
132.46
131.58
131.31
113.19
113.11
77.31
77.00
76.68
55.26
55.13
39.98
33.54
20.66
f1 (ppm)

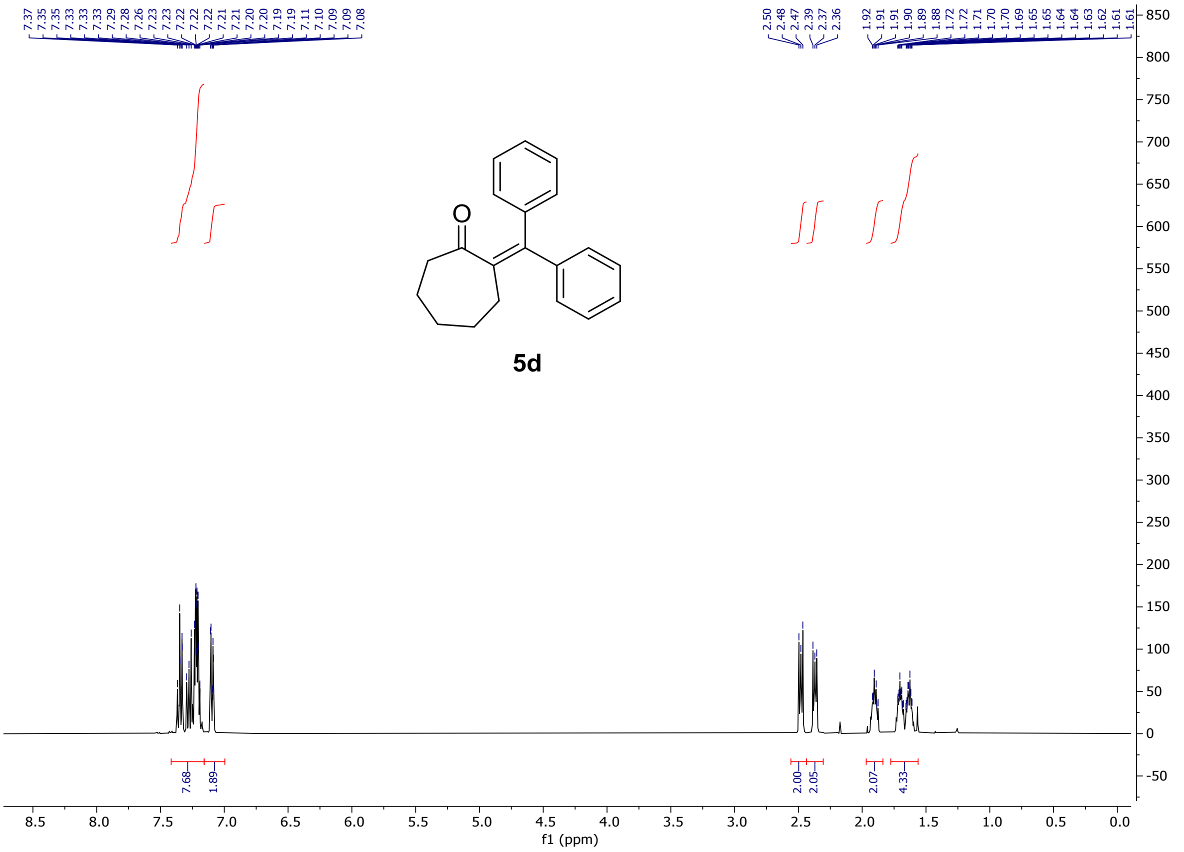
7.37 7.35 7.35 7.33 7.33 7.33 7.29 7.28 7.26 7.23 7.23 7.22 7.22 7.22 7.22 7.21 7.21 7.20 7.20 7.19 7.19 7.11 7.10 7.09 7.09 7.08
2.50 2.48 2.47 2.39 2.37 2.36
1.92 1.91 1.91 1.90 1.89 1.88 1.72 1.72 1.71 1.70 1.69 1.65 1.65 1.64 1.64 1.63 1.62 1.61 1.61
O
5d
7.68 1.89 2.00 2.05 2.07 4.33
f1 (ppm)

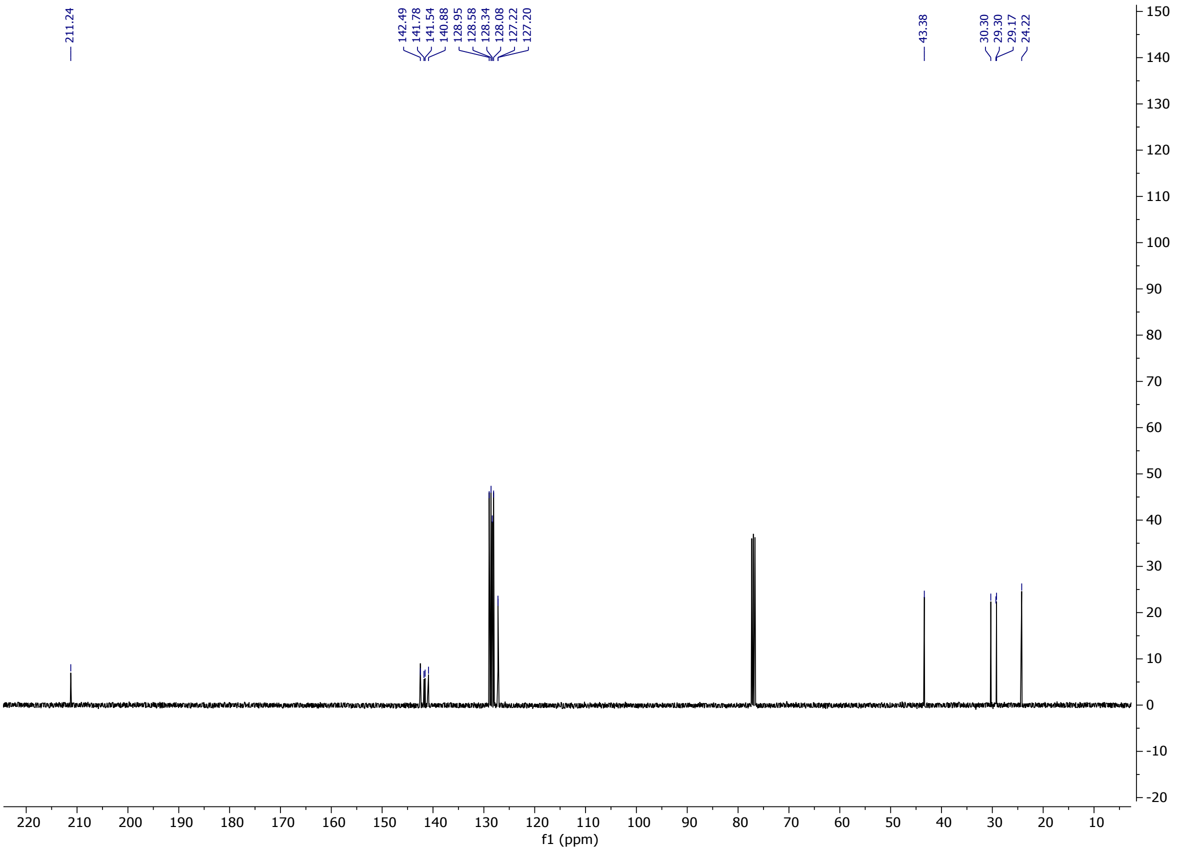
211.24
142.49 141.78 141.54 140.88 128.95 128.58 128.34 128.08 127.22 127.20
43.38
30.30 29.30 29.17 24.22
f1 (ppm)

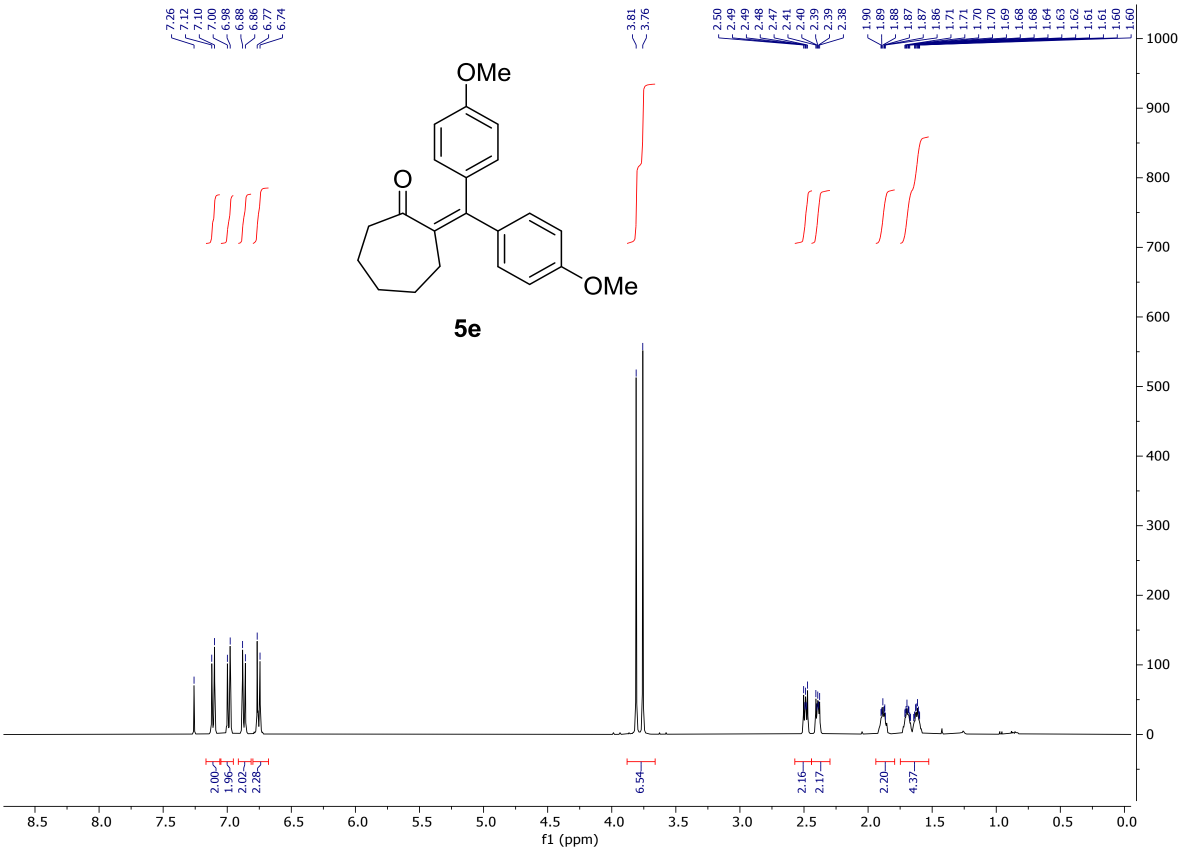
7.26 7.12 7.10 7.00 6.98 6.88 6.86 6.77 6.74
3.81 3.76
2.50 2.49 2.49 2.48 2.47 2.41 2.40 2.39 2.39 2.38
1.90 1.89 1.88 1.87 1.87 1.86 1.71 1.71 1.70 1.69 1.68 1.68 1.64 1.63 1.62 1.61 1.61 1.60 1.60
OMe
O
OMe
5e
2.00 1.96 2.02 2.28
6.54
2.16 2.17
2.20 4.37
f1 (ppm)

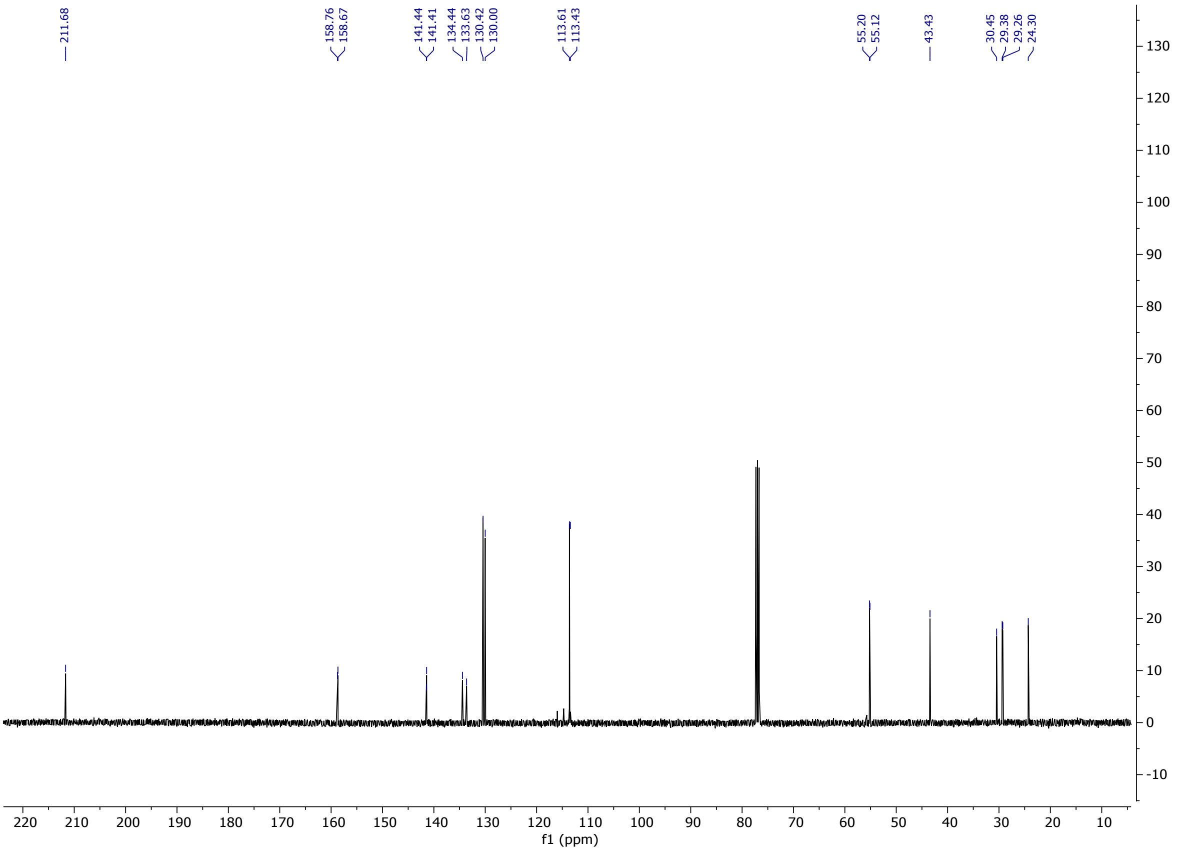
211.68
158.76 158.67
141.44 141.41 134.44 133.63 130.42 130.00
113.61 113.43
55.20 55.12
43.43
30.45 29.38 29.26 24.30
f1 (ppm)

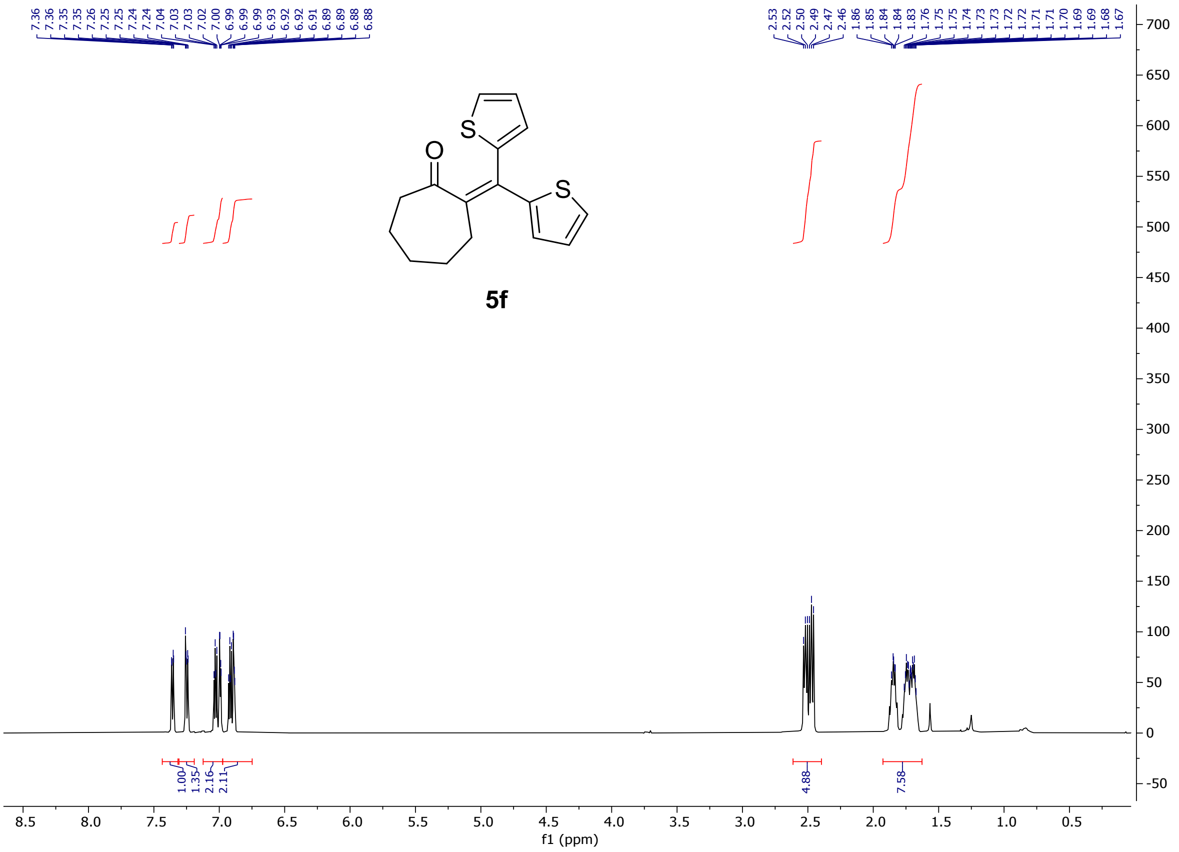
7.36 7.36 7.35 7.35 7.26 7.25 7.25 7.24 7.24 7.04 7.03 7.03 7.02 7.00 6.99 6.99 6.99 6.93 6.92 6.92 6.91 6.89 6.89 6.88 6.88
2.53 2.52 2.50 2.49 2.47 2.46 1.86 1.85 1.84 1.84 1.83 1.76 1.75 1.75 1.74 1.73 1.73 1.72 1.72 1.71 1.71 1.70 1.69 1.69 1.68 1.67
O
S
S
5f
1.00
1.35
2.16
2.11
4.88
7.58
f1 (ppm)

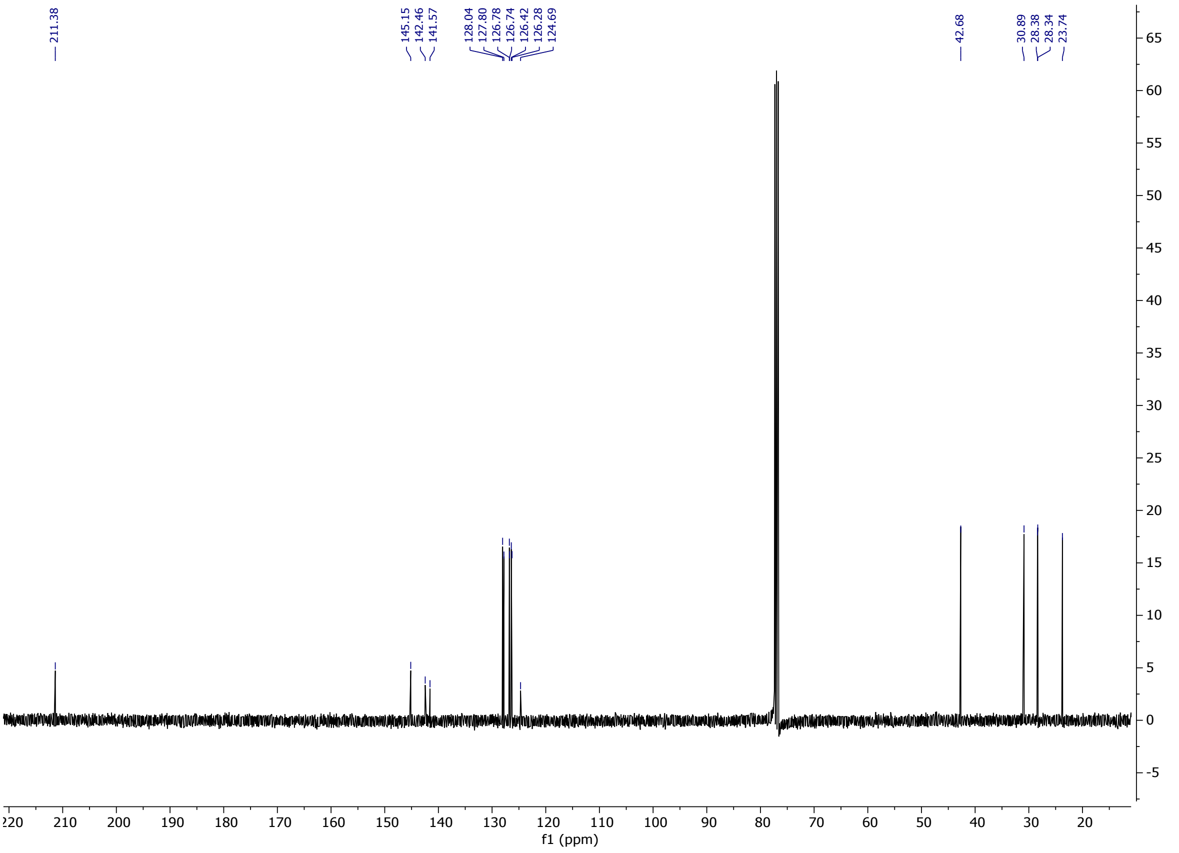
211.38
145.15 142.46 141.57
128.04 127.80 126.78 126.74 126.42 126.28 124.69
42.68
30.89 28.38 28.34 23.74
f1 (ppm)

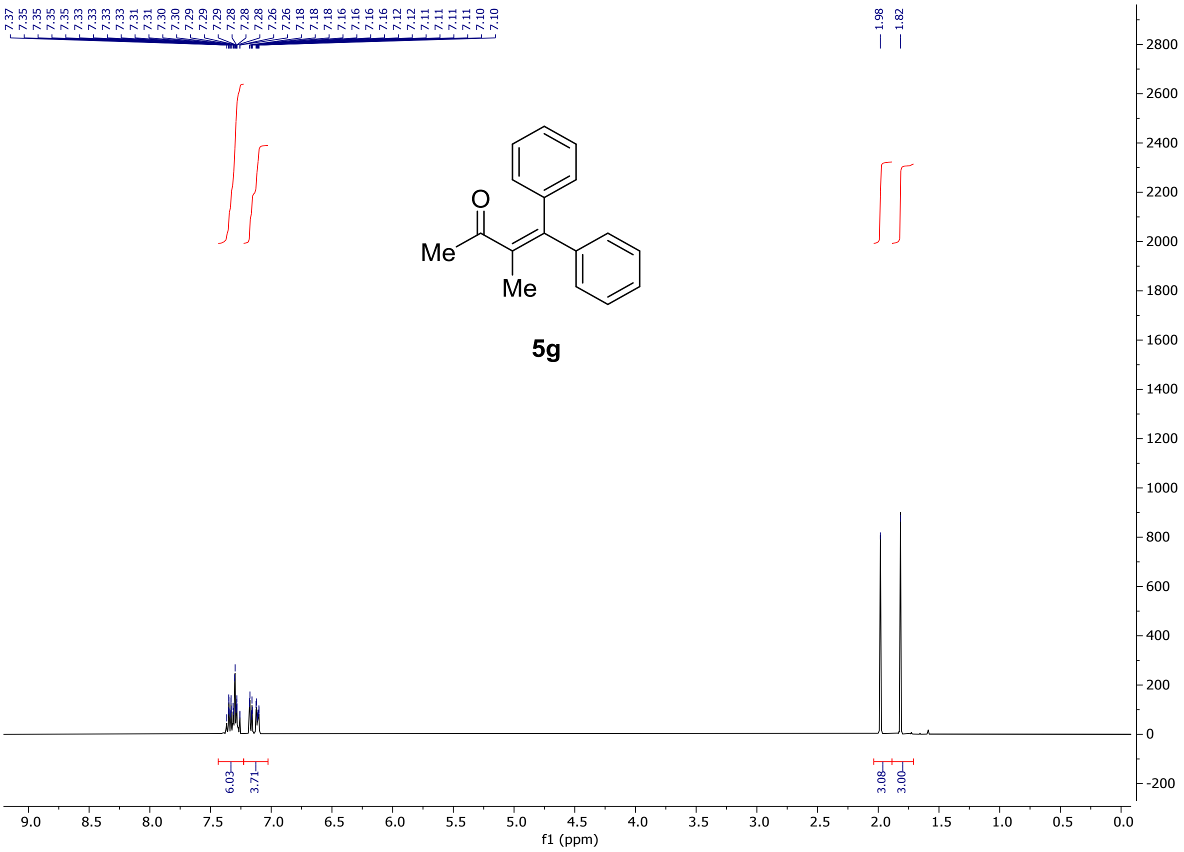
7.37 7.35 7.35 7.35 7.35 7.33 7.33 7.33 7.33 7.31 7.31 7.30 7.30 7.29 7.29 7.29 7.28 7.28 7.28 7.26 7.26 7.18 7.18 7.18 7.16 7.16 7.16 7.12 7.12 7.11 7.11 7.11 7.11 7.10 7.10
1.98
1.82
O
Me
Me
5g
6.03
3.71
3.08
3.00
f1 (ppm)

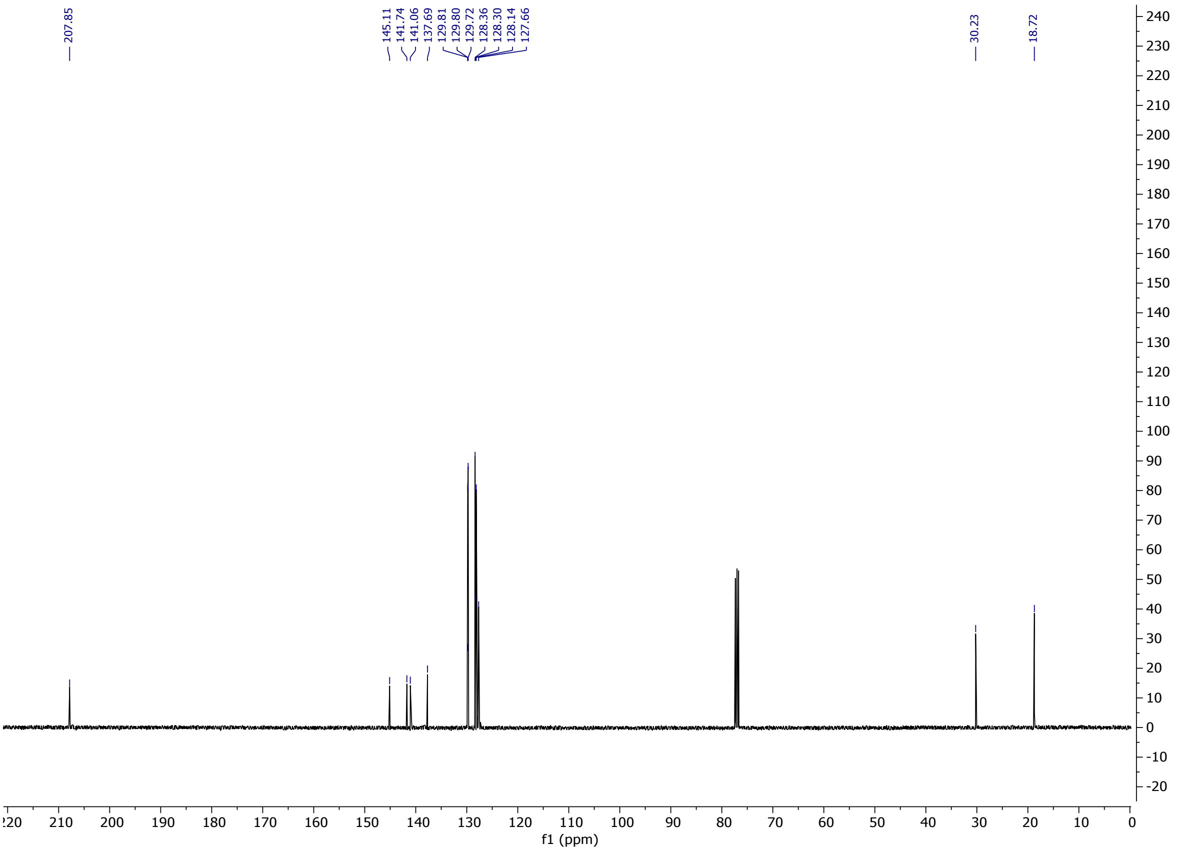
207.85
145.11 141.74 141.06 137.69 129.81 129.80 129.72 128.36 128.30 128.14 127.66
30.23
18.72
f1 (ppm)

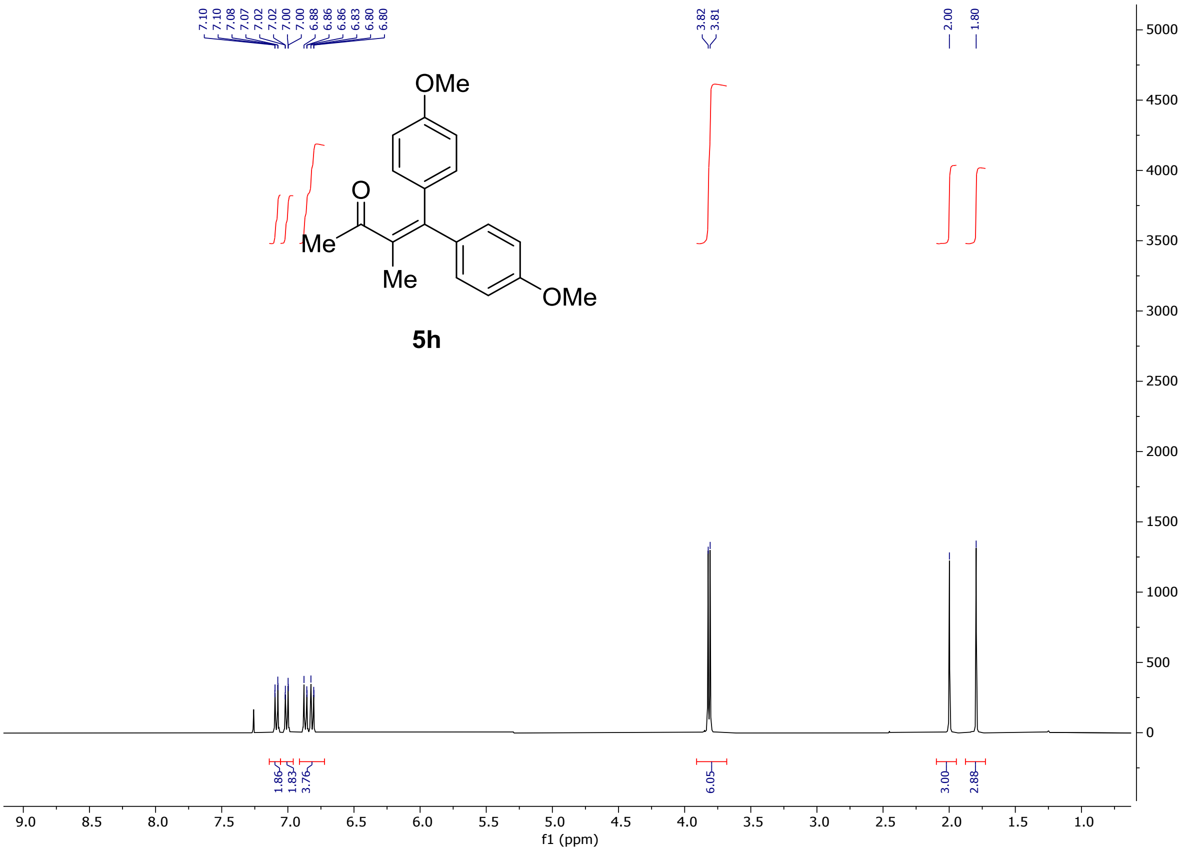
7.10
7.10
7.08
7.07
7.02
7.02
7.00
7.00
6.88
6.86
6.86
6.83
6.80
6.80
3.82
3.81
2.00
1.80
OMe
O
Me
Me
OMe
5h
1.86
1.83
3.76
6.05
3.00
2.88
f1 (ppm)

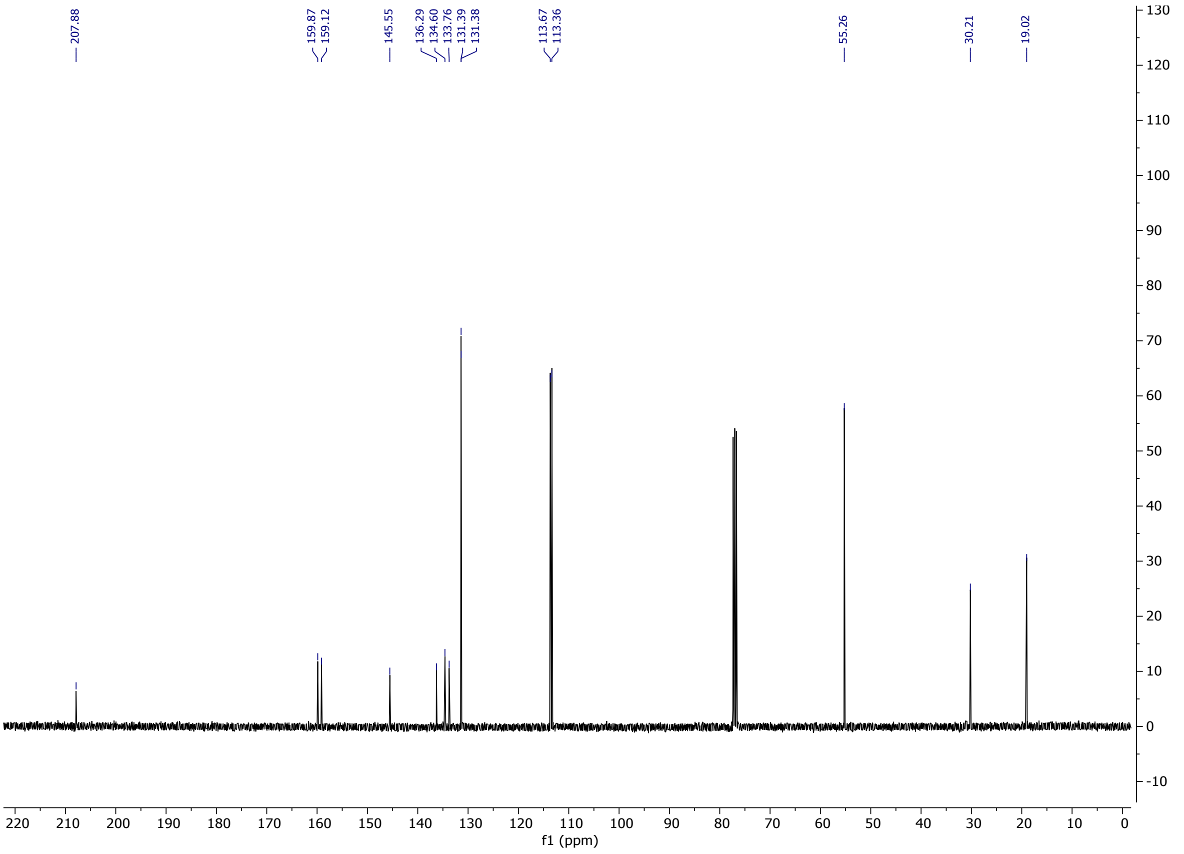
207.88
159.87
159.12
145.55
136.29
134.60
133.76
131.39
131.38
113.67
113.36
55.26
30.21
19.02
f1 (ppm)

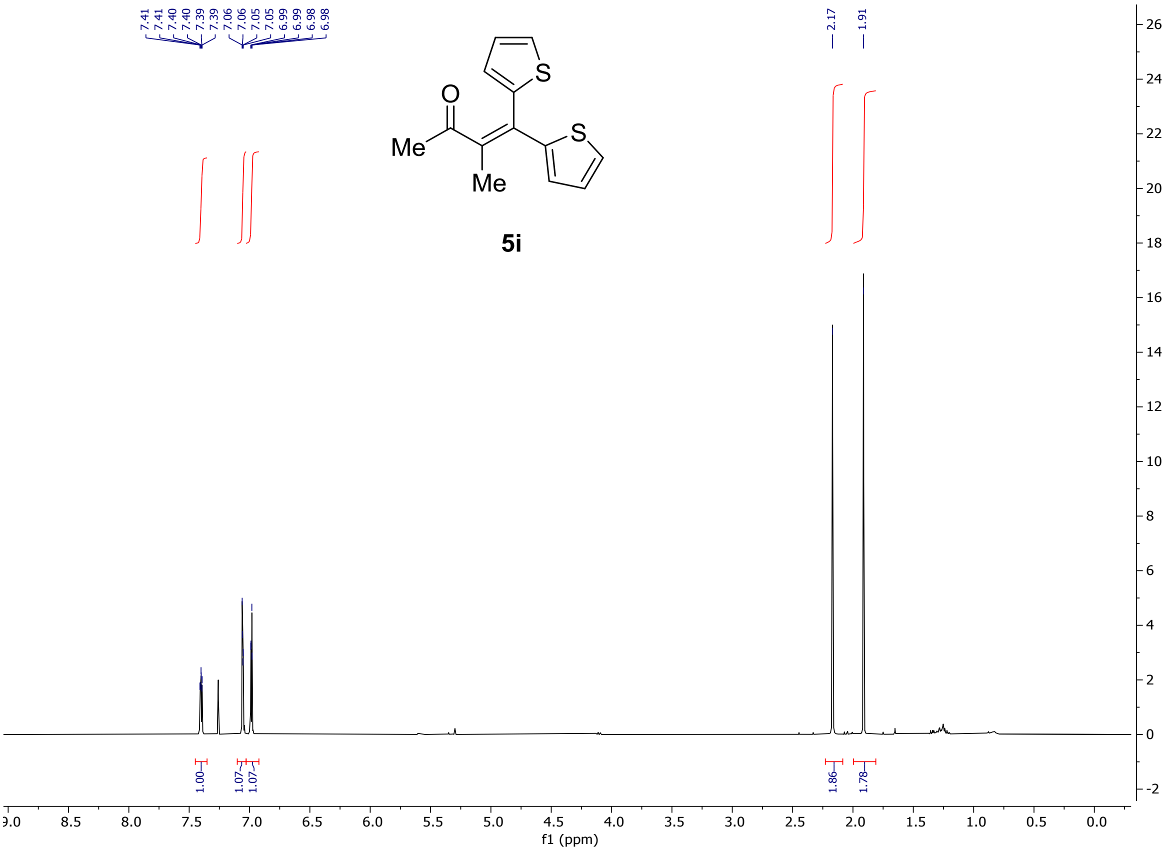
O
Me
Me
S
S
5i
7.41 7.41 7.40 7.40 7.39 7.39 7.06 7.06 7.05 7.05 6.99 6.99 6.98 6.98
2.17
1.91
1.00
1.07
1.07
1.86
1.78
f1 (ppm)

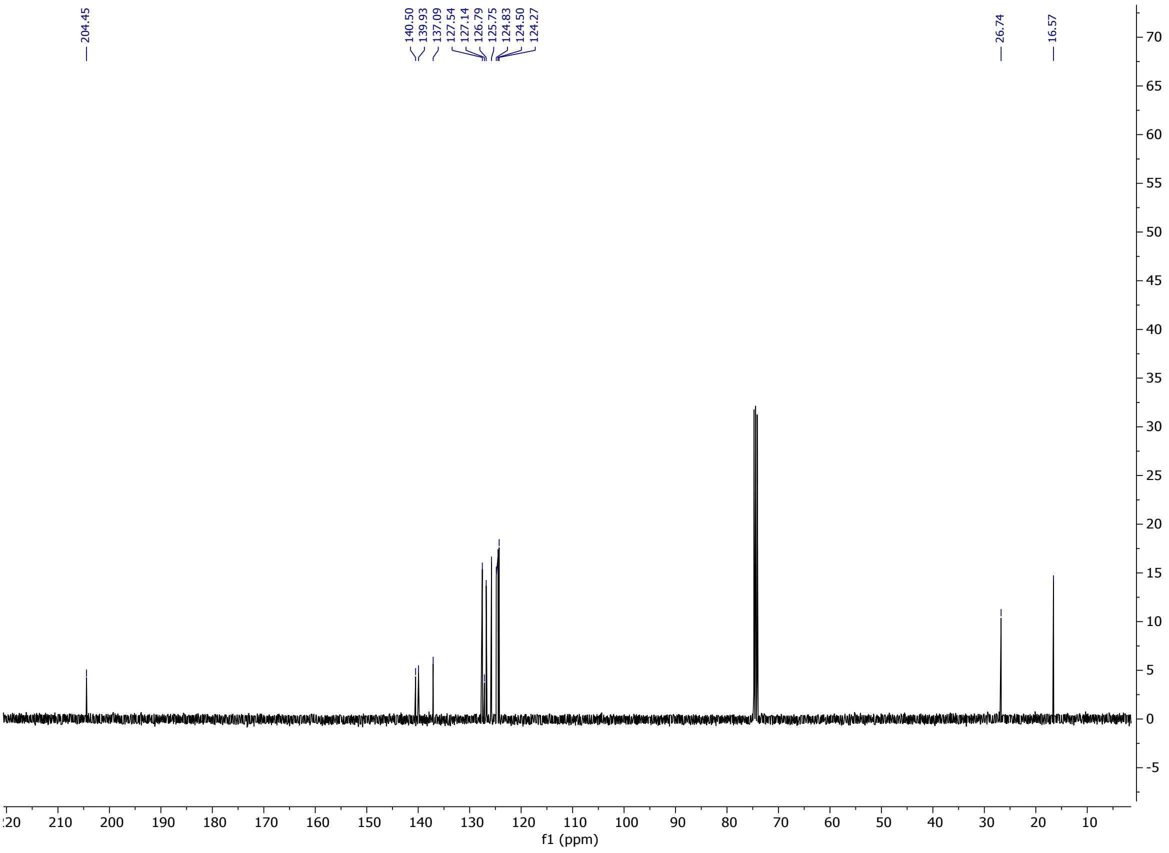
204.45
140.50 139.93 137.09 127.54 127.14 126.79 125.75 124.83 124.50 124.27
26.74
16.57
f1 (ppm)

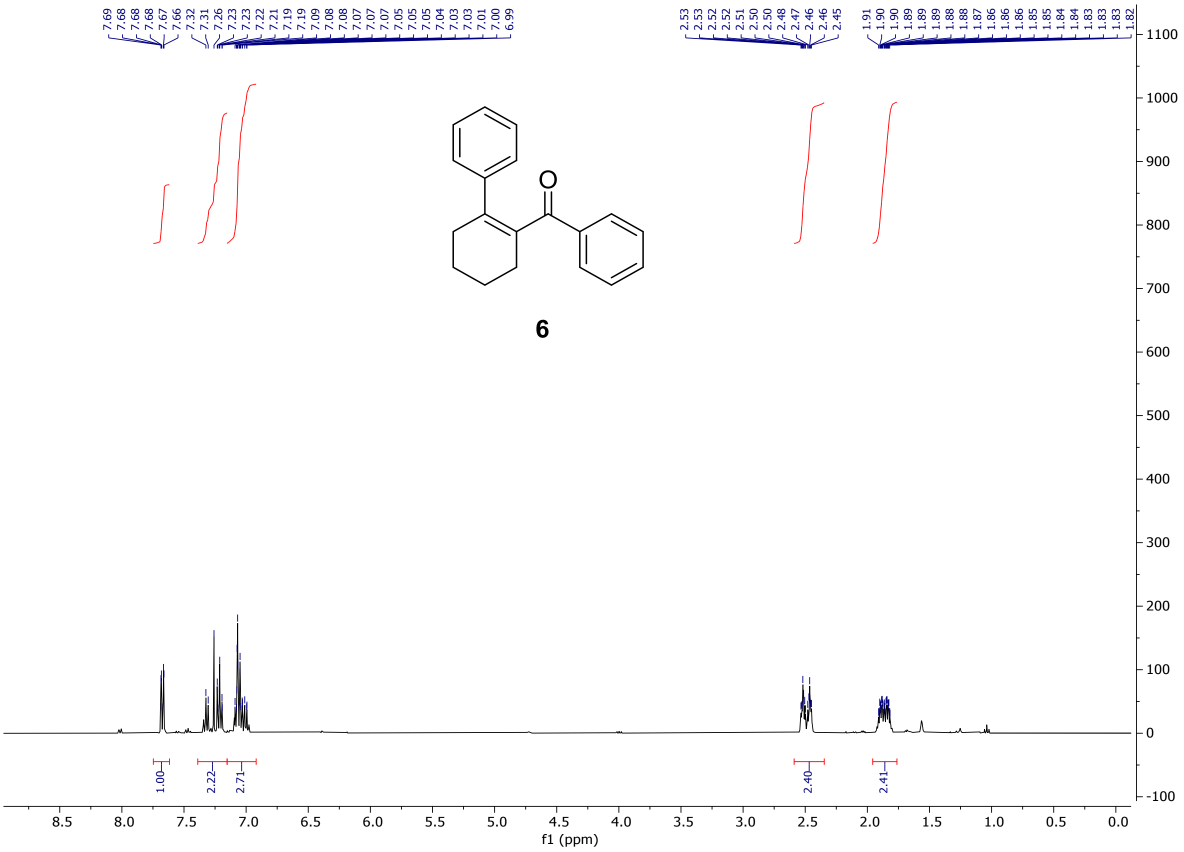
7.69 7.68 7.68 7.68 7.67 7.66 7.32 7.31 7.26 7.23 7.23 7.22 7.21 7.19 7.19 7.09 7.08 7.08 7.07 7.07 7.05 7.05 7.05 7.04 7.03 7.03 7.01 7.00 6.99
2.53 2.53 2.52 2.51 2.50 2.50 2.48 2.47 2.46 2.46 2.45
1.91 1.90 1.90 1.89 1.89 1.88 1.88 1.87 1.86 1.86 1.85 1.85 1.84 1.84 1.83 1.83 1.83 1.82
O
6
1.00
2.22
2.71
2.40
2.41
f1 (ppm)

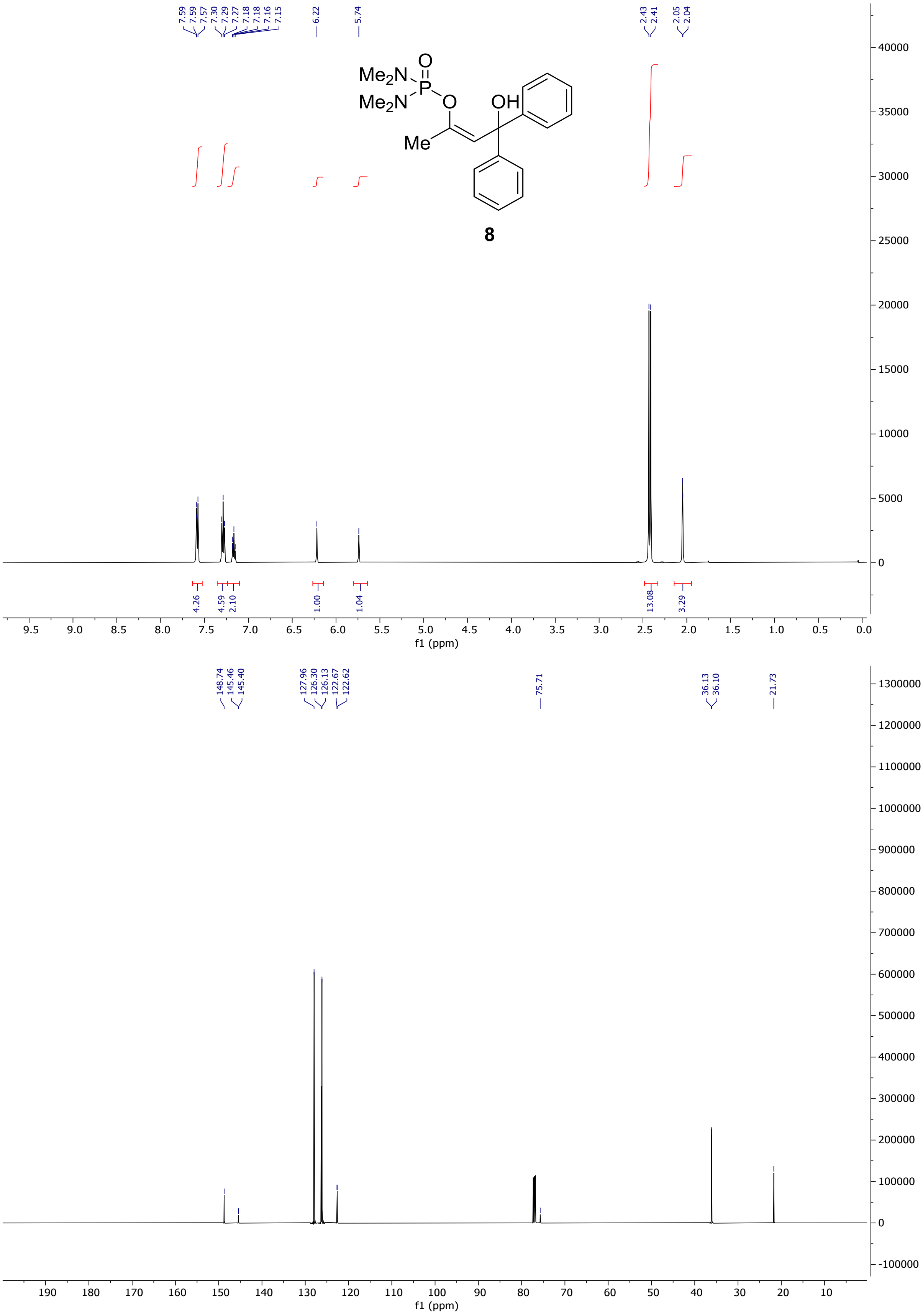
7.59
7.59
7.57
7.30
7.29
7.27
7.18
7.18
7.16
7.15
6.22
5.74
2.43
2.41
2.05
2.04
Me2N
Me2N
P
O
O
OH
Me
8
4.26
4.59
2.10
1.00
1.04
13.08
3.29
f1 (ppm)
148.74
145.46
145.40
127.96
126.30
126.13
122.67
122.62
75.71
36.13
36.10
21.73
f1 (ppm)
